# Concentration- and chromosome-organization-dependent regulator unbinding from DNA for transcription regulation in living cells

**DOI:** 10.1038/ncomms8445

**Published:** 2015-07-06

**Authors:** Tai-Yen Chen, Ace George Santiago, Won Jung, Łukasz Krzemiński, Feng Yang, Danya J. Martell, John D. Helmann, Peng Chen

**Affiliations:** 1 Department of Chemistry and Chemical Biology, Cornell University, Ithaca, New York 14853, USA; 2 Department of Microbiology, Cornell University, Ithaca, New York 14853, USA

## Abstract

Binding and unbinding of transcription regulators at operator sites constitute a primary mechanism for gene regulation. While many cellular factors are known to regulate their binding, little is known on how cells can modulate their unbinding for regulation. Using nanometer-precision single-molecule tracking, we study the unbinding kinetics from DNA of two metal-sensing transcription regulators in living *Escherichia coli* cells. We find that they show unusual concentration-dependent unbinding kinetics from chromosomal recognition sites in both their apo and holo forms. Unexpectedly, their unbinding kinetics further varies with the extent of chromosome condensation, and more surprisingly, varies in opposite ways for their apo-repressor versus holo-activator forms. These findings suggest likely broadly relevant mechanisms for facile switching between transcription activation and deactivation *in vivo* and in coordinating transcription regulation of resistance genes with the cell cycle.

## Abstract (web summary)

Binding and unbinding of transcription regulators at operator sites regulates gene expression. By single-molecule tracking of metal-sensing regulators, here the authors show that the unbinding kinetics depends on regulator concentration and chromosome condensation, and varies with their metal-binding states.

## Graphical abstract

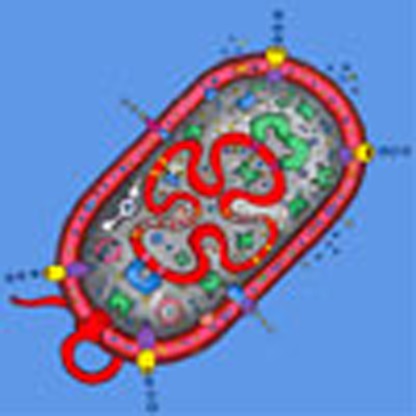

Binding and unbinding of transcription regulators at operator sites constitutes a primary mechanism for regulating gene expression, and they are often rate-determining for regulatory responses^1^,^2^,^3^. For binding to an operator site, its rate is readily modulated by many cellular factors such as regulators’ cellular concentration and the chromosome organization^4^,^5^,^6^,^7^,^8^,^9^,^10^,^11^. For unbinding from an operator site, it is unclear whether these factors can modulate its rate for regulation. At least, regulator unbinding from an operator site on DNA is presumed to be a unimolecular reaction and thus independent of regulators’ cellular concentration. Surprisingly, recent *in vitro* studies revealed that CueR, a Cu^+^-sensing efflux regulator, can undergo assisted dissociation and direct substitution processes on its cognate DNA; both lead to its concentration-dependent unbinding rate from a recognition site on DNA and may facilitate CueR in deactivating transcription^12^. This unusual concentration-dependent unbinding was also reported recently for nonspecific chromosomal organization proteins^13^, DNA polymerase^14^ and a single-strand-DNA-binding protein^15^, all functionally unrelated to CueR. Moreover, using force to apply tension, which changes DNA conformation, can modulate protein unbinding kinetics from DNA^16^,^17^.

Despite the above discoveries *in vitro*, it remains unknown whether concentration- and DNA-conformation-dependent protein unbinding from DNA is relevant *in vivo*. Here using stroboscopic single-molecule tracking (SMT)^2^ to image protein−DNA interactions in real time and at nanometer spatial resolution, we study the quantitative DNA interaction kinetics of CueR and its Zn^2+^-sensing homologue ZntR in living *Escherichia coli* (*E. coli*) cells. CueR and ZntR, both belonging to the MerR-family regulators, bind tightly to their respective dyad-symmetric recognition sequences within σ^70^-dependent suboptimal promoters, either repressing or, with Cu^+^ or Zn^2+^ bound (10^−21^ M affinity and 10^−15^ M affinity, respectively^18^,^19^), activating the transcription of efflux genes to defend against metal stress^19^,^20^,^21^. We find that in living *E. coli* cells, CueR and ZntR show striking concentration-dependent unbinding kinetics from chromosomal recognition sites in both their apo and holo forms. Unexpectedly, their unbinding kinetics further varies with the extent of chromosome condensation, and more surprisingly, varies in opposite ways for apo-repressor versus holo-activator forms. These findings suggest novel mechanisms for facilitating transcription deactivation and activation *in vivo* and in coordinating transcription regulation of resistance genes with the cell cycle, which are likely broadly relevant for gene regulation.

## Results

### Concentration-dependent regulator residence time

**Figure 1:**
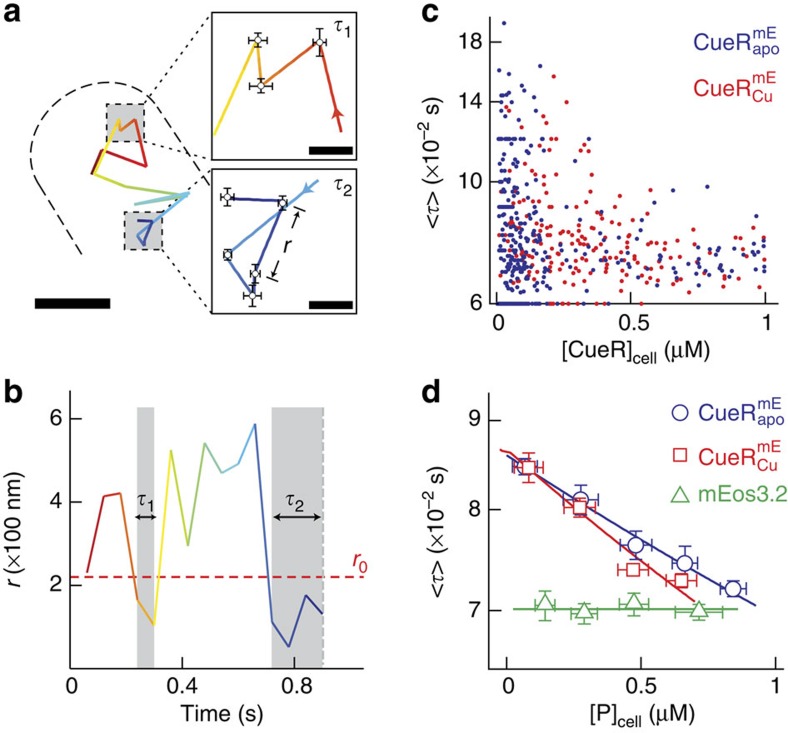
Protein-concentration-dependent residence time of CueR and ZntR on chromosome from stroboscopic single-molecule tracking in living *E. coli* cells. (**a**) Position trajectory of a 
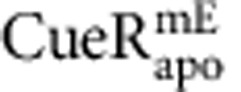
 molecule in a living cell. Dash line is the cell boundary. Zoom-in insets: locations (that is, residence sites) associated with the two residence times in **b**. Displacement *r* per time-lapse is the distance the molecule travelled between two consecutive images as shown in the *τ*_2_ inset. Scale bars in **a** and in the insets are 500 nm and 80 nm, respectively. (**b**) Displacement *r* per time-lapse (*T*_tl_=60 ms) versus time trajectory for the molecule in **a**. *τ*_1_ and *τ*_2,_ whose lengths are denoted by grey shades and double-headed arrows, are two microscopic residence times thresholded by *r*_0_=220 nm (horizontal red dashed line). (**c**) Correlation of average residence time 〈*τ*〉 and total protein concentration in each cell for 
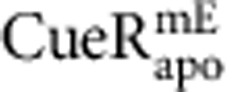
 and 
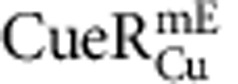
 from ∼450 and 250 cells containing a total of ∼12,000 and 10,000 molecules, respectively. (**d**) Dependence of 〈*τ*〉 on cellular protein concentration for 
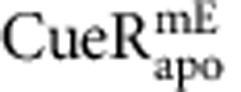
 , 
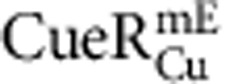
 or free mEos3.2 as a control. 〈*τ*〉 of individual cells from **c** are grouped every ∼150 nM along the *x*-axis by their cellular protein concentrations and averaged within each group. Note 1 nM corresponds approximately to one protein molecule per cell volume (about 1.5 fL). The solid lines are empirical fits with 〈*τ*〉=(*a*[P]_cell_+*b*)^−1^ (Supplementary Note 8), except for mEos3.2, for which the line is a horizontal eye guide. *x*, *y* error bars are s.d. and s.e.m., respectively. Relevant data for 
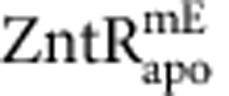
 and 
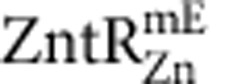
 are in Supplementary Figs 16 and 19.

To visualize CueR or ZntR in living cells, we made its functional fusion with the photoconvertible fluorescent protein mEos3.2 (refs 22, 23; that is, CueR^mE^ or ZntR^mE^) at its chromosomal locus as well as in a plasmid for varying its cellular concentration (Methods and Supplementary Note 2). We used time-lapse stroboscopic imaging^2^,^24^,^25^,^26^,^27^,^28^,^29^ to track the positions over time of single photoconverted mEos3.2-tagged proteins in a cell at tens of nanometer precision until their mEos3.2 tags photobleached (Fig. 1a; Methods and Supplementary Note 5.2). For each tracked protein molecule, we obtained its time trajectory of displacement *r* per time-lapse (that is, the distance the protein molecule travelled between two consecutive images) (Fig. 1a,b). This SMT approach, along with single-cell total fluorescence counting, also enabled us to quantify the copy number of CueR or ZntR in each cell (Methods and Supplementary Note 5.3). By sorting the cells into groups of similar cellular protein concentrations, we could analyse protein-concentration-dependent processes without being limited by the large cell-to-cell heterogeneity in protein expression levels (Supplementary Note 5.3).

We first examined 
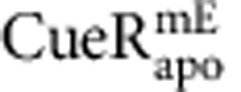
 and 
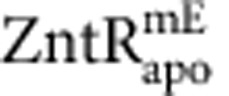
, whose metal-binding cysteines were mutated to make them permanently locked in the apo-repressor forms (that is, C112S for CueR^18^ and C115S for ZntR^30^). The displacement-versus-time trajectory of a single 
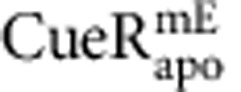
 (or 
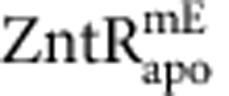
) in a cell shows clear transitions between large and small *r* values; the small *r* values are expected to be dominated by protein binding to chromosome, thus being nearly stationary (Fig. 1b). Thresholding the displacement-versus-time trajectory with an upper displacement limit *r*_0_ (for example, *r*_0_=220 nm; see later for justification of this value) selects out those small displacements and gives the estimates of the individual time durations (that is, the microscopic residence time *τ*) of a single protein molecule at a chromosomal binding site. Each microscopic residence time *τ* starts when *r* drops below *r*_0_ and ends when *r* jumps above *r*_0_ (for example, *τ*_1_ in Fig. 1b) or when the mEos3.2-tag photobleaches/blinks (for example, *τ*_2_ in Fig. 1b). By measuring many individual *τ*’s from a large number of single-molecule displacement trajectories, we obtained the average residence time 〈*τ*〉 for each cell, whose cellular concentration of 
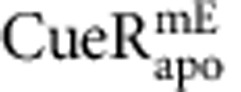
 (or 
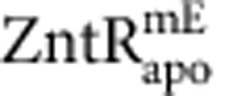
) was also determined (Fig. 1c).

Strikingly, the average residence time 〈*τ*〉 decreases with increasing cellular concentration of 
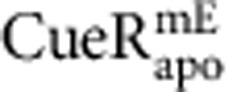
 or 
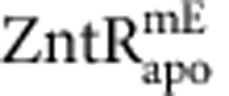
 (Fig. 1c,d). This trend is independent of the value of the thresholding *r*_0_ and persists after correcting for the contribution of mEos3.2 photobleaching/blinking kinetics (Supplementary Note 8). Free mEos3.2, which does not bind to DNA and exhibits much shorter apparent 〈*τ*〉, does not show this trend in the cell (Fig. 1d).

We next examined un-mutated CueR^mE^ and ZntR^mE^ in cells grown with 100 μM Cu^2+^ or Zn^2+^ in the medium (that is, 
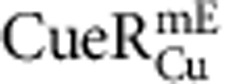
 and 
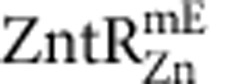
). These metal concentrations cause maximal induction of the *cueR* or *zntR* regulons in the cell^30^,^31^, and the regulators are largely metallated. Their 〈*τ*〉’s show differences from those of 
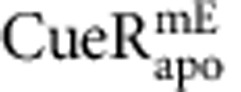
 and 
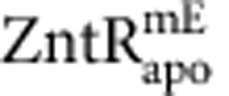
, indicating that we indeed observe the behaviour of holo-regulators (Fig. 1c,d). Importantly, their average residence times on chromosome still exhibit a decreasing trend with increasing cellular protein concentrations (Fig. 1c,d). Therefore, for both apo and holo forms of CueR or ZntR, higher cellular protein concentrations shorten their average residence times on chromosome, suggesting concentration-enhanced protein unbinding kinetics from DNA.

### Concentration-enhanced regulator unbinding

**Figure 2:**
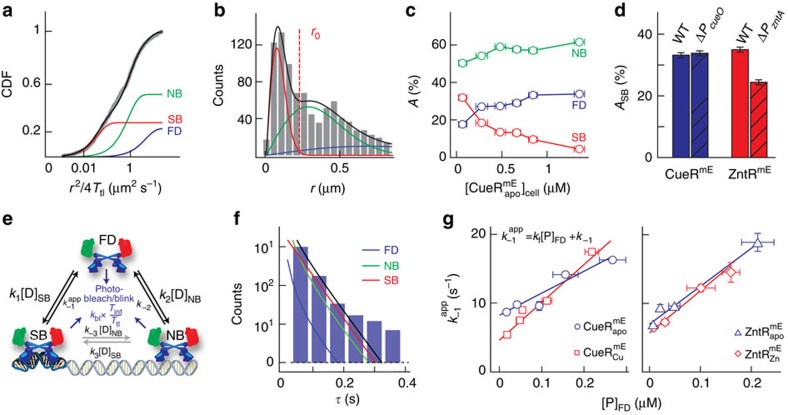
Protein-concentration-dependent unbinding of CueR and ZntR from recognition sites. (**a**) Cumulative-distribution-function (CDF) of displacement *r* (plotted against 
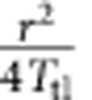
) of 
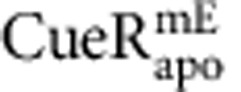
 at 
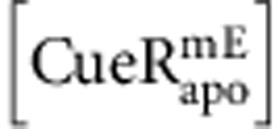
_cell_=180±34 nM; fitting (black line) with three diffusion states gives effective diffusion constants (and fractional populations) (equation (1)): *D*_FD_=3.7±0.2 μm^2^ s^−1^ (23.2±0.3%), *D*_NB_=0.70±0.03 μm^2^ s^−1^ (49.2±0.4%) and *D*_SB_=0.036±0.009 μm^2^ s^−1^ (27.6±0.5%), which are also plotted individually. (**b**) Histogram of displacement *r* and the corresponding resolved FD, NB and SB states as in **a**. The solid lines are the overall probability density function (PDF) (black; equation (2)) and the resolved three components of *r* (red, green and blue as in **a**), all multiplied by a scaling factor to account for the actual number of measured displacements. The vertical red dash line denotes *r*_0_=220 nm, as in Fig. 1b. (**c**) Protein-concentration-dependent fractional populations (*A*’s, in percentage) of FD, NB and SB states for 
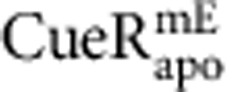
. For example, *A*_SB_ is equivalent to (number of proteins bound at recognition sites)/(total number of proteins in the cell). Data for ZntR are summarized in Supplementary Tables 4 and 5. (**d**) Fractional populations of the SB state (*A*_SB_) for CueR^mE^ and ZntR^mE^ in wild-type (WT) or promoter knockout strains at cellular protein concentrations of ∼100 nM. (**e**) Kinetic model for regulator−DNA interactions in a cell. The direct interconversions between NB and SB states are assumed to be negligible, but indicated (grey arrows). Parameters are defined in detail in Methods and Supplementary Fig. 29. (**f**) Histogram in log scale of residence time *τ* for 
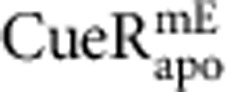
 at 
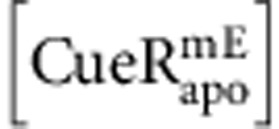
_cell_=180 nM; fitting with a three-state kinetic model (black line, equation (6)) gives the apparent unbinding rate constant (
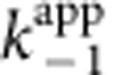
) from recognition sites. Contribution from each state is also plotted. (**g**) Dependences of 
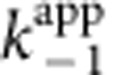
 on free protein concentration (=*A*_FD_[P]_cell_) in cells for 
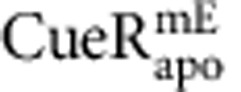
 and 
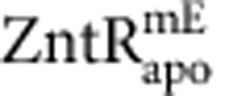
 and for 
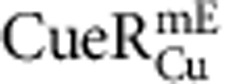
 and 
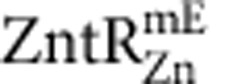
. Lines are fits with 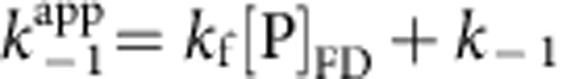 (equation (8)). All error bars are s.d.

To quantify the variable motions of individual CueR or ZntR molecules in a cell, we determined their cumulative distribution functions (CDFs) of displacement *r* (Fig. 2a)^32^. Regardless of the metallation state of CueR or ZntR, global analysis of CDFs across all cellular protein concentrations resolves minimally three diffusion states with effective diffusion constants of ∼3.7 μm^2^ s^−1^, 0.7 μm^2^ s^−1^ and 0.04 μm^2^ s^−1^ (referred to as *D*_FD_, *D*_NB_ and *D*_SB_, respectively; Methods and Supplementary Note 11). No subcellular localization or protein aggregation was observed, and hence these two are not the reasons for the presence of the resolved three diffusion states (Supplementary Note 13). We assigned these three diffusion states as proteins that are: freely diffusing (FD) in the cytoplasm; nonspecifically bound (NB) to and moving on chromosome (the contributions from nonspecific interactions with the plasmids in the cell is <7% and thus negligible; Supplementary Note 20.5); and specifically bound (SB) to chromosomal recognition sites, whose slow motions reflect the chromosome dynamics (and measurement uncertainties). Both experimental evidences and simulations (details in Supplementary Notes 12 and 15) support these assignments: (1) CueR or ZntR can bind to DNA specifically and nonspecifically^12^,^21^,^33^,^34^. (2) The three effective *D*’s are consistent with reported values for proteins freely diffusing in the cytoplasm, nonspecifically bound or specifically bound to chromosome^2^,^25^,^28^,^32^,^35^, when the cell confinement effect^24^ and the time-lapse effect of imaging^32^ are both taken into account. Note that the magnitude of the effective diffusion constant *D*_FD_ here is smaller than that measured for free diffusing protein *in vitro*^36^; this apparent difference results from the cell confinement effect (Supplementary Note 15.2 and Supplementary Figs 35 and 36). (3) Control experiments on tracking free mEos3.2 in cells with variable time lapses show similar effective diffusion constants as the FD state (Supplementary Fig. 23). (4) Simulations of molecular diffusion in confined cell geometries. (5) Short-time movement analysis for the SB state agrees with the literature reported chromosome diffusion constant (Supplementary Fig. 26).

Moreover, the resolved CDF also gives the fractional populations (that is, percentages) of the CueR (or ZntR) in the FD, NB and SB states among all CueR (or ZntR) protein molecules in the cell. With increasing cellular protein concentrations, the fractional populations of *D*_FD_ and *D*_NB_ increase, while that of *D*_SB_ decreases (Fig. 2c); these trends are consistent with that at higher protein concentrations, each protein molecule will spend more time freely diffusing or nonspecifically bound to the chromosome than specifically bound at recognition sites, as more protein molecules compete for the limited number of recognition sites. Deleting the *cueO* promoter (Δ*P*_*cueO*_), one of the many CueR operator sites, expectedly did not cause much perturbation, including CueR^mE^’s fractional population at the SB state (Fig. 2d and Supplementary Note 18.3). In contrast, deleting the *zntA* promoter (Δ*P*_*zntA*_), the only known ZntR operator site in *E. coli*, did decrease, but surprisingly did not abolish, ZntR^mE^’s SB state (Fig. 2d and Supplementary Note 18.3). This observation motivated us to identify many additional possible ZntR recognition sites in the *E. coli* genome (Supplementary Note 22).

The resolved CDF of *r* concurrently gives the corresponding resolved probability density function (PDF) of *r* (Fig. 2b), in which the three resolved peaks correspond to the FD, NB and SB states. The resolution of these three states in this PDF of *r* immediately justifies the *r*_0_ value that thresholds the displacement-versus-time trajectories—For *r* smaller than *r*_0_≈220 nm, >99% of SB states of CueR (or ZntR) are included, and the thresholded residence times *τ* are thus dominated by contributions from proteins specifically bound at chromosomal recognition sites (and those nonspecifically bound to chromosome at higher cellular protein concentrations), with very little contribution from freely diffusing proteins in the cytoplasm. To quantitatively deconvolute all possible contributions to *τ*, we formulated a minimal mechanistic model comprising the FD, NB and SB states (Fig. 2e and Supplementary Note 14). This model enabled us to analyse the distribution of *τ* to obtain 
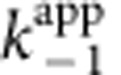
, the apparent unbinding rate constant from recognition sites (Fig. 2f), as well as the fractional populations of different states to obtain other kinetic constants for protein binding and unbinding at recognition sites and nonspecific DNA sites (Methods, Table 1, and Supplementary Tables 7 and 8).

Strikingly, 
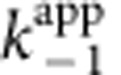
, the apparent unbinding rate constant from recognition sites, increases linearly with increasing cellular concentrations of the free (or total) 
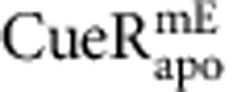
 or 
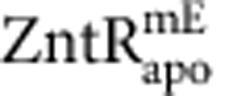
 (Fig. 2g), in contrast to unimolecular reaction kinetics of protein unbinding from DNA where the unbinding rate constant is independent of free protein concentration. Linear fitting of 
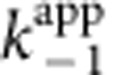
 versus free protein concentration gives the slope *k*_f_ and intercept *k*_−1_, the facilitated (second order) and spontaneous (first order) unbinding rate constants from recognition sites, respectively. For 
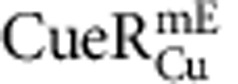
 or 
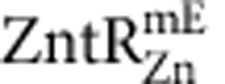
, the slopes are even steeper (Fig. 2g). Therefore, both apo and holo forms of CueR and ZntR have concentration-dependent unbinding kinetics from chromosomal recognition sites, where the dependence likely results from the assisted dissociation or direct substitution mechanism that we discovered for CueR−DNA interactions *in vitro*^12^ (see Discussion and Supplementary Fig. 41b and Supplementary Note 18.2)^15^,^37^,^38^,^39^,^40^.

### Different extents of chromosome condensation among cells

We next examined the spatial distribution in a cell of the residence sites associated with the residence times of CueR and ZntR, which are dominated by their binding to chromosomal recognition sites and nonspecific sites (Fig. 1a insets). This distribution can reflect the chromosome organization^41^ because: (1) The recognition sites of CueR and ZntR spread across the chromosome randomly (Supplementary Note 22.3), (2) their nonspecific binding sites are expected to scatter across the chromosome randomly as well (Supplementary Note 20.6) and (3) contributions from nonspecific binding to the plasmids in the cell are negligible (<7%; Supplementary Note 20.5).

**Figure 3:**
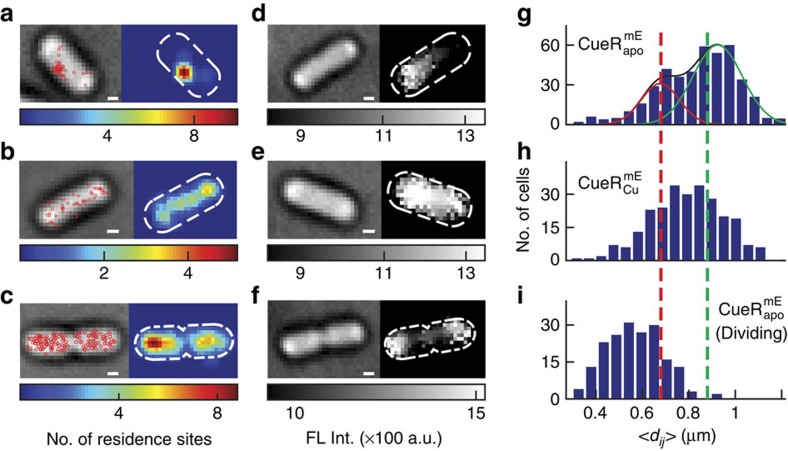
Different extents of chromosome condensation among individual cells. (**a**–**c**) Scatter (red circles, overlaid on cell transmission image; left) and two-dimensional histogram (right) plots of protein residence sites on chromosome for nondividing (**a**,**b**) and dividing (**c**) cells expressing 
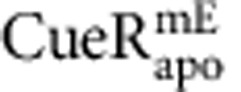
. The residence sites in **a** localize to a region; those in **b** spread over the cell. The dividing cell in **c** shows two localized regions of residence sites. All scale bars: 400 nm. (**d**–**f**) Transmission (left) and fluorescence (right) images of Hoechst-dye-stained chromosomes in nondividing (**d**,**e**) and dividing cells (**f**). The chromosome in **d** is compact; that in **e** spreads over the cell. The dividing cell in **f** has two highly compact chromosomes. (**g**–**i**) Distributions of average pairwise distance 〈*d*_*ij*_〉 between residence sites among nondividing cells expressing 
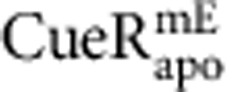
 (**g**), 
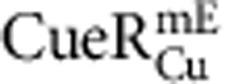
 (**h**) or among dividing cells expressing 
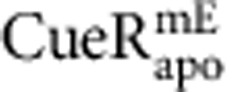
 (**i**). 〈*d*_*ij*_〉 of dividing cells are significantly shorter than those of nondividing cells. Lines in **g** are Gaussian fits. Vertical dash lines at 〈*d*_*ij*_〉=0.68 and 0.88 μm are for dividing and grouping the cells.

In some cells, these residence sites localize to a small region (Fig. 3a), reflecting that in these cells the chromosomes are highly condensed. In contrast, in some other cells, the residence sites spread over the cell (Fig. 3b), reflecting that the cells’ chromosomes are less condensed. We further directly imaged the Hoechst-dye-stained chromosomes in the cells. Consistently, some cells have compact chromosomes (Fig. 3d), while others have their chromosomes spread over the entire cell volume (Fig. 3e), reflecting again the different extents of chromosome condensation among the individual cells.

To quantify the extent of chromosome condensation for each cell, we computed the average pairwise distance 〈*d*_*ij*_〉 between the residence sites as a measure. We validated this measure by comparing with results from direct imaging of dye-stained chromosomes (Supplementary Note 20.2). The distributions of 〈*d*_*ij*_〉 among cells are broad (Fig. 3g,h), reflecting that the extent of chromosome condensation differs significantly from cell to cell. For cells expressing 
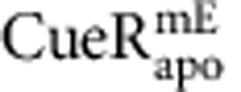
, for example, two subpopulations can be resolved, corresponding to those with more or less condensed chromosomes (that is, smaller or larger 〈*d*_*ij*_〉, respectively; Fig. 3g).

### Chromosome-organization-dependent regulator unbinding

**Figure 4:**
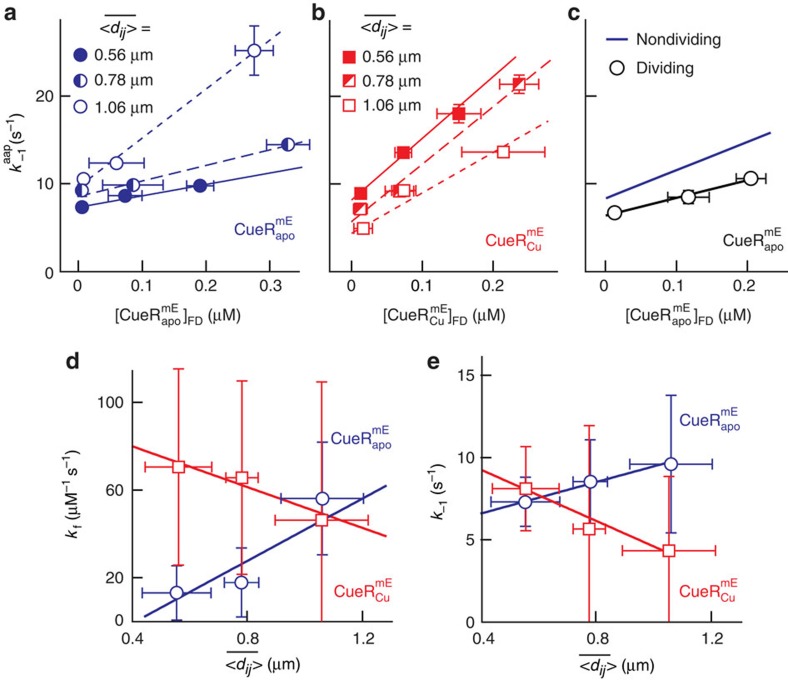
Chromosome-organization-dependent unbinding of CueR and ZntR from recognition sites. (**a**,**b**) Protein-concentration-dependent 
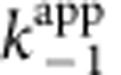
 of 
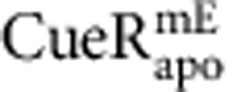
 and 
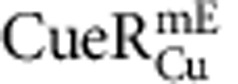
 in nondividing cells with different average 〈*d*_*ij*_〉, 
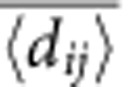
. Lines are linear fits with 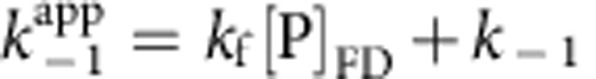, as in Fig. 2g. (**c**) Same as **a** but for dividing cells, in comparison with 
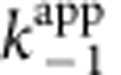
 for nondividing cells from Fig. 2g. (**d**,**e**) 
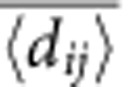
-dependent facilitated unbinding and spontaneous unbinding rate constants *k*_f_ and *k*_−1_ of 
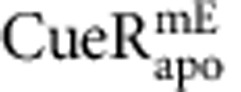
 and 
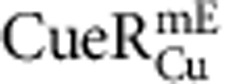
 in nondividing cells. Lines are linear fits (Supplementary Note 21.3). Data for ZntR are in Supplementary Note 21. All error bars are s.d.

We sorted the cells into three groups based on their 〈*d*_*ij*_〉 to examine how the unbinding of CueR and ZntR from recognition sites may relate to chromosome organization. Within each group, we determined the dependence of 
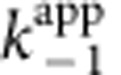
, the apparent unbinding rate constant from recognition sites, on the cellular protein concentration. Remarkably, for 
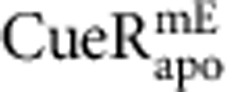
 and 
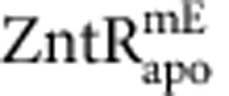
, while their 
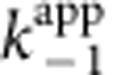
 preserve the protein-concentration dependence in each group, their 
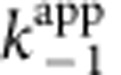
 in cells with more condensed chromosome (that is, smaller 〈*d*_*ij*_〉) are up to three times smaller than those in cells with less condensed chromosome (that is, larger 〈*d*_*ij*_〉; Fig. 4a and Supplementary Fig. 50c). As a control, we treated the cells expressing 
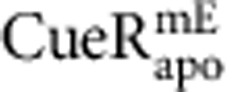
 and 
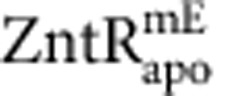
 with the drug chloramphenicol, which is known to cause chromosome compaction^42^. Consistently, this chloramphenicol treatment leads to a decrease of 
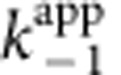
 across all accessible cellular protein concentrations (Supplementary Note 21.5).

More surprisingly, an opposite trend applies to the metallated holo-proteins with regard to the dependences of their unbinding kinetics on the extent of chromosome condensation. For 
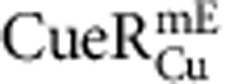
 and 
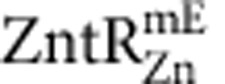
, their 
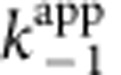
 are larger in cells with more condensed chromosomes (that is, smaller 〈*d*_*ij*_〉) at any cellular protein concentration (Fig. 4b and Supplementary Fig. 50f).

We further obtained *k*_f_ and *k*_−1_, the second-order facilitated and first-order spontaneous unbinding rate constants from recognition sites, for cells with different extents of chromosome condensation (Fig. 4a,b). Correspondingly, *k*_f_ and *k*_−1_ both show opposite dependences on 〈*d*_*ij*_〉 between the apo and holo forms of CueR or ZntR; and they can change by up to nine times with 〈*d*_*ij*_〉 ranging from ∼0.5 to 1.2 μm (Fig. 4d,e, and Supplementary Note 21.3). Altogether, these results suggest that chromosome organization modulates both the facilitated and spontaneous unbinding of CueR and ZntR from recognition sites, but in opposite directions depending on the regulators’ metallation state.

## Discussion

The concentration- and chromosome-organization-dependent unbinding of CueR and ZntR from their recognition sites open up new possibilities for regulating transcription of their associated metal resistance genes. The concentration-dependent unbinding of CueR and ZntR, together with their spontaneous unbinding from DNA, may facilitate the switching between the transcriptionally activated state (bound holo-protein) and the repressed state (bound apo-protein), which both require protein binding to the same operator site (Supplementary Note 18.2). Transcription deactivation by these regulators will likely involve the unbinding of a promoter-bound holo-activator from DNA followed by binding of an apo-repressor, rather than the dissociation of the tightly bound metal from the promoter-bound holo-protein^18^,^19^, which is expected to be slow. For 
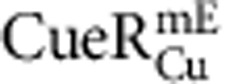
, just the basal concentration range of ∼17–240 nM (Supplementary Fig. 17) can already change its unbinding rate from a recognition site from ∼4.7 to 7.9 s^−1^, a ∼70% increase. On the other hand, transcription activation will likely involve the unbinding of a promoter-bound apo-repressor followed by binding of a holo-activator, rather than the direct metallation of the promoter-bound apo-repressor, which faces competition for the metal by many other apo-repressors in the cell^43^,^44^. For 
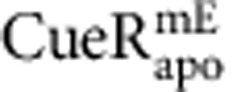
, the basal concentration range of ∼14–185 nM can change its unbinding from a recognition site from ∼8.3 to 9.8 s^−1^.

**Figure 5:**
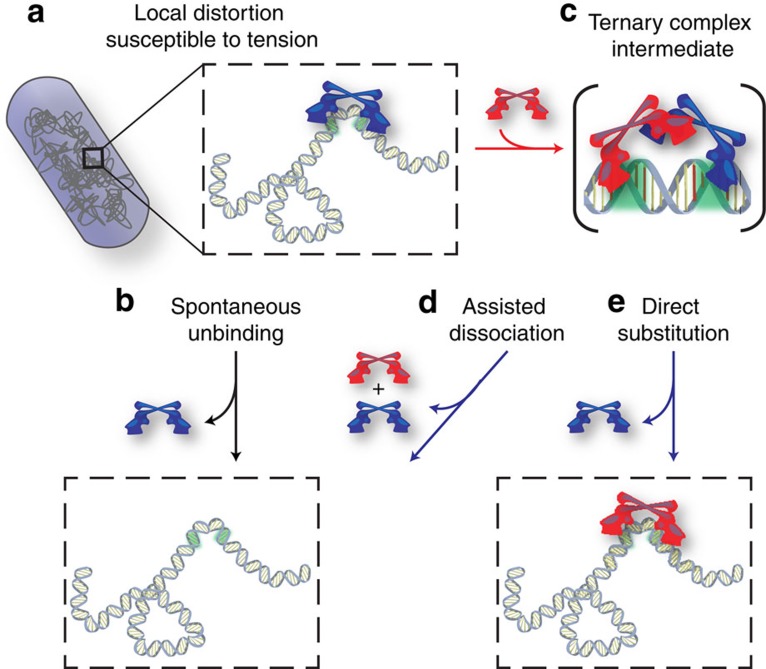
Schematic mechanism for concentration- and chromosome-organization-dependent unbinding from recognition sites. On binding to a recognition site in the chromosome, the homodimeric protein CueR or ZntR distorts the DNA structure (**a**), making the complex susceptible to chromosome organization and associated tension in DNA. Green colour on chromosome denotes the two halves of the dyad-symmetric recognition sequence. The bound regulator (blue) can unbind spontaneously (**b**). Alternatively, another regulator (red) from the cytoplasm can bind to one half of the recognition sequence, which is vacated by the incumbent one, forming a ternary complex as an intermediate (**c**), which can then proceed to result in assisted dissociation (**d**) or direct substitution (**e**), giving rise to concentration-dependent unbinding.

Mechanistically, concentration-dependent unbinding of CueR or ZntR from chromosomal recognition sites likely results from their assisted dissociation or direct substitution process, which we discovered previously in studying CueR interactions with a specific DNA *in vitro* (Supplementary Note 18.2)^12^. In the assisted dissociation process, a protein from the surrounding solution (for example, the cytoplasm) helps carry away the incumbent protein bound at the recognition site (Fig. 5d); in the direct substitution process, the incoming protein directly replaces the incumbent one (Fig. 5e); both processes depend on the protein concentration in the surrounding. Both of these processes likely involve a ternary complex as a common intermediate, in which two protein molecules each partially bind to the recognition site (Fig. 5c), as we proposed previously^37^. This ternary complex is possible because of the homodimeric nature of CueR and ZntR: they can form bivalent contacts with DNA in which their two DNA-binding domains bind to the two halves of their dyad-symmetric recognition sequences (Fig. 5a). Under thermal fluctuation, one of the DNA-binding domains of a protein molecule could transiently detach, allowing another protein from solution to bind to the vacant half dyad sequence, leading to a ternary complex (Fig. 5c). This ternary complex could then proceed in two possible pathways: both proteins fall off, resulting in an assisted dissociation (Fig. 5d), or one of the two proteins falls off, where 50% of the chance would result in a direct substitution (Fig. 5e). Although no evidence exists for higher-order oligomeric CueR or ZntR complexes on regular DNA, our previous *in vitro* study has revealed a related ternary complex in CueR interactions with an engineered DNA Holliday junction^37^,^45^. Relatedly, ternary complexes involving multivalent contacts with DNA were also proposed to rationalize the concentration-dependent unbinding from DNA of nonspecific chromosomal organization proteins^38^,^39^ that are dimeric and of a single-strand-DNA-binding protein^15^ that has multiple DNA-binding domains.

Regarding the correlation between the unbinding kinetics of CueR (or ZntR) and chromosome organization, as well as the opposite trends between their apo-repressor versus holo-activator forms, we postulate that they may help the cell modulate transcription of metal resistance genes during the growth cycle. The unbinding of apo- and holo-CueR (or ZntR) from recognition sites is important for activation and deactivation of transcription, respectively. Under optimal growth conditions without metal stress, cells divide frequently. The dividing cells tend to have highly condensed chromosomes^5^,^7^, which we verified by examining the spatial distribution of residence sites of CueR (or ZntR) on chromosome and by direct chromosome staining (Fig. 3c,f,i). Their highly condensed chromosomes should lead to slower unbinding of apo-CueR and apo-ZntR (that is, repressors) and faster unbinding of holo-CueR and holo-ZntR (that is, activators); both lead to less (now unneeded) activation of metal resistance genes, beneficial for saving energy for cell division. On the other hand, under metal stress conditions, cells barely divide and their chromosomes are less condensed (Fig. 3h versus Fig. 3i). Here apo-CueR or apo-ZntR unbinds faster from promoters, leading to more facile transcription activation to defend against metal stress. Moreover, holo-CueR or holo-ZntR unbinds slower here, which would keep activating transcription longer. Supporting this postulate, we found that the apparent unbinding rate constants (
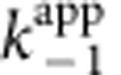
) of 
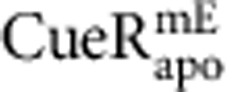
 and 
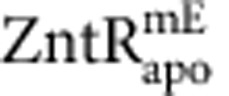
 in dividing cells are indeed significantly slower than those in nondividing cells across all cellular protein concentrations (Fig. 4c and Supplementary Fig. 52c).

Mechanistically, the chromosome-organization-dependent unbinding of CueR and ZntR from recognition sites likely stems from their ability to impose DNA structural distortions. The holo forms of these regulators bend and unwind DNA at the recognition sites, as shown by structural studies of related MerR-family regulators in complex with DNA^46^,^47^. These protein-imposed DNA distortions should lead to a susceptibility of the protein−DNA complexes to mechanical tensions in DNA (Fig. 5a), and thus to chromosome organization^16^,^17^ in which the extent of condensation can exert variable tensions along the chromosome^48^,^49^ (Supplementary Note 21.6). This susceptibility should then give rise to dependences on chromosome organization for both spontaneous unbinding (Fig. 5b) and facilitated unbinding (Fig. 5d,e) of CueR and ZntR from their recognition sites. Moreover, it is known that apo and holo forms of MerR-family regulators distort DNA structure differently^47^,^50^,^51^,^52^,^53^ (the detailed structural differences are not yet defined though). These differences could be the reason that apo- and holo-CueR or ZntR respond to chromosome organization in opposite trends.

Many DNA-interacting proteins form multivalent contacts with DNA or impose structural distortions on DNA^54^. For the former, these proteins should thus be capable of forming ternary complexes at recognition sites on chromosome, making assisted dissociation or direct substitution processes possible. For the latter, their normal complexes should be susceptible to DNA tension and thus to chromosome organization. Therefore, the concentration- and chromosome-organization-dependent unbinding from recognition sites discovered here could be broadly relevant for protein−DNA interactions and gene regulation in cells.

## Methods

### Materials and sample preparation

All strains were derived from the *E. coli* BW25113 strain (Supplementary Note 2). CueR^mE^ or ZntR^mE^ was generated by fusing to the C terminus of CueR or ZntR the monomeric, irreversibly photoconvertible fluorescent protein mEos3.2 (that is, mE)^22^,^23^. The corresponding genes were integrated at the chromosomal loci via λ-RED homologous recombination^55^. An L-arabinose-inducible plasmid pBAD24 containing CueR^mE^ or ZntR^mE^ was further introduced to allow for variable protein expression. On the basis of the CueR and ZntR structures^18^, their C-termini are distant from their DNA-binding domains, and thus the fusion tag is expected to not interfere with DNA binding. Cell growth assays of mEos3.2-tagged strains in comparison with the wild-type and knockout strains show that both fusion proteins are functional (Supplementary Note 3). The intactness of the fusion proteins were examined via standard SDS–PAGE or western blot; for the latter, the fusion proteins were further tagged at the C-termini with the FLAG epitope (Supplementary Note 3). CueR^mE^ is essentially intact in cells (estimated to have <8% cleaved mEos3.2, comparable to our measurement errors). ZntR^mE^ shows significant degradation in cells, consistent with prior literature^33^. This degradation results in a time-dependent cellular concentration of mEos3.2-tagged ZntR during our imaging experiments, and our results thus reflect an averaged protein unbinding kinetics over the corresponding protein concentration range. Nevertheless, this degradation does not affect ZntR’s apparent unbinding rate constant 
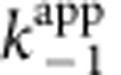
 from recognition sites, but its *k*_f_, the facilitated unbinding rate constant from recognition sites, is likely underestimated and its *k*_−1_, the spontaneous unbinding rate constant from recognition sites, is likely overestimated (Supplementary Note 17).


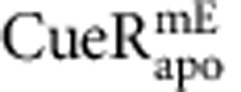
 or 
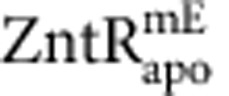
 was generated by mutating one of its Cu- or Zn-binding cysteines to serine (that is, C112S for CueR^18^ and C115S for ZntR^30^) to make it permanently apo and constitutively functional as a repressor. The corresponding genes were cloned in a pBAD24 plasmid, which was introduced into the Δ*cueR* or Δ*zntR* knockout strain, respectively. The Δ*P*_*cueO*_ or Δ*P*_*zntA*_ promoter knockout was done via λ-RED recombination in the Δ*cueR* or Δ*zntR* strain, respectively, into which the pBAD24 plasmid containing CueR^mE^ or ZntR^mE^ was introduced.

*E. coli* cells expressing mEos3.2-tagged CueR or ZntR were imaged at room temperature on an agarose pad sandwiched between a coverslip and a slide in a sealed chamber (details in Supplementary Note 4). The cells were first grown in LB medium overnight at 37°C. The cultures were then diluted 1:100 in (metal depleted) M9 medium supplemented with amino acids and vitamins and grown at 37 °C until OD_600_ reached 0.3, at which point L-arabinose was added to induce expression for 5–30 min. The cells were then recovered by centrifugation, washed, re-suspended in M9 medium containing glucose, MEM amino acids and MEM vitamins, and incubated for 60 min before being placed on a 3% agarose gel. Note that for metal stress conditions, CuSO_4_ or ZnSO_4_ solution was also added into the cell solution to a final concentration of 100 μM at this stage. Gold particles (100 nm) were drop-casted on the coverslip as position marks for drift correction. The cells are viable under our imaging conditions, with a doubling time of ∼250 min, similar to cells not exposed to the imaging lasers (Supplementary Note 4.2).

Based on the bright-field transmission images, single cells with an aspect ratio of ∼2.5 and having no visible division septum were analysed; they are referred to as nondividing cells. Under our experimental conditions, these single cells were in either B or C phase of the cell cycle, containing one or a partially replicated chromosome. Dividing cells (that is, cells showing a clear division septum, for example, Fig. 3c,f) were also analysed and they were in the D phase of the cell cycle containing two copies of chromosome (Supplementary Note 4.4).

### SMT and cell protein quantification

Individual cells were studied using SMT via time-lapse stroboscopic imaging and single-cell quantification of protein concentration (SCQPC). Time-lapse stroboscopic imaging^2^,^24^,^25^,^26^,^27^,^28^,^29^ was adapted to track the motions of single photoconverted mEos3.2-tagged proteins in a cell for high temporal (that is, 4 ms) and spatial (that is, ∼20 nm) resolution, using an Olympus IX71 microscope and inclined epi-illumination (Supplementary Note 5). Single mEos3.2 photoconversion was achieved by controlling the power density and illumination time of a 405-nm laser. Short 561-nm excitation laser pulses (pulse duration *T*_int_=4 ms) were synchronized with camera exposures so that even a fast-moving protein with a typical diffusion constant of 3–11 μm^2^ s^−1^ in bacterial cytoplasm would not move beyond a diffraction-limited focus during *T*_int_. This stroboscopic imaging yielded a diffraction-limited fluorescence spot for a protein in an image, where the protein’s centroid could be localized to tens of nanometer precision through two-dimensional Gaussian fitting. By capturing images in a time-lapse manner (lapse time *T*_tl_=30 to 400 ms; all data presented in the main text are at *T*_tl_=60 ms), we could track the motion of each protein until its mEos3.2 tag photobleached (for example, Fig. 1a) and obtain the corresponding time trajectory of displacement *r* between adjacent images (for example, Fig. 1b). Individual molecules were probed using the 561-nm laser pulses for 30 imaging frames after each photoconversion. This photoconversion-imaging-and-bleaching cycle was then repeated for 500 times followed by the SCQPC step to quantify the rest of proteins that were not tracked.

In SCQPC (Supplementary Note 5.3), the remaining number of mEos3.2-tagged protein molecules in each cell was determined in two steps: (1) Photoconverting all of them to the red form and imaging the total red fluorescence intensity of the cell. (2) Dividing the total fluorescence intensity by the average fluorescence intensity of a single mEos3.2 (which was derived from the earlier SMT for the same single cell). Note that in step (1), depending on the amount of remaining mEos3.2, the EM gain of the camera was adjusted accordingly to ensure the fluorescence signal was within the linear regime of camera response. The fluorescence signal was then corrected by the EM gain to obtain the total fluorescence intensity for quantification. An important feature here was to use the single mEos3.2 fluorescence intensity determined in the same cell to eliminate the need to correct for power density differences of the imaging laser across the illumination area. The photoconversion efficiency (0.42)^56^,^57^ of mEos3.2 and the protein oligomeric state (CueR and ZntR are stable homodimers) were also included in determining the total copy number of CueR or ZntR in each cell. The protein copy number was converted to protein concentration by using the cell volume determined from its transmission image: the cell boundary was fitted with the model of a cylinder with two hemispherical caps as reported^58^ (Supplementary Fig. 13a). The quantification of each cell’s CueR or ZntR concentration allowed us to sort the individual cells into groups of similar cellular protein concentrations.

The overall imaging time for SMT and SCQPC processes for each cell is ∼30 min. This imaging time is much shorter than the average cell doubling time (∼250 min) under our conditions and the cell morphology stays the same throughout the measurements.

### Analysis of single-molecule diffusive motions

The effective diffusion constants and fractional population of different states were extracted by analysing the CDF of displacement. Experimentally, the CDF of displacement *r* per time-lapse was constructed using only the first displacement of each position trajectory to prevent long trajectories from biasing the sampling^32^. After constructing CDFs across a range of cellular protein concentrations, we globally fitted them with a three-diffusion-state CDF (*C*_3_(*r*), equation (1) and Supplementary Equation 11, and Fig. 2a), which is a linear combination of three terms:




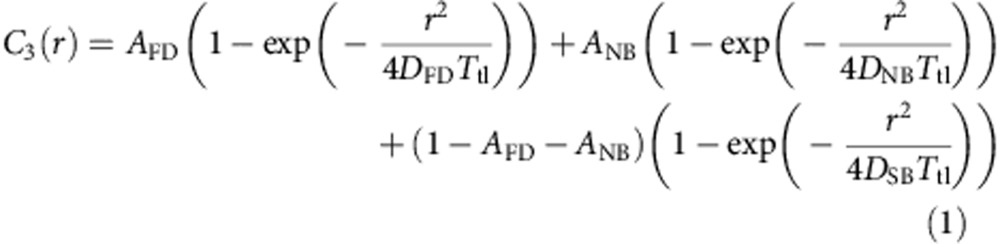




The corresponding three-state PDF of *r*, *P*_3_(*r*) is:




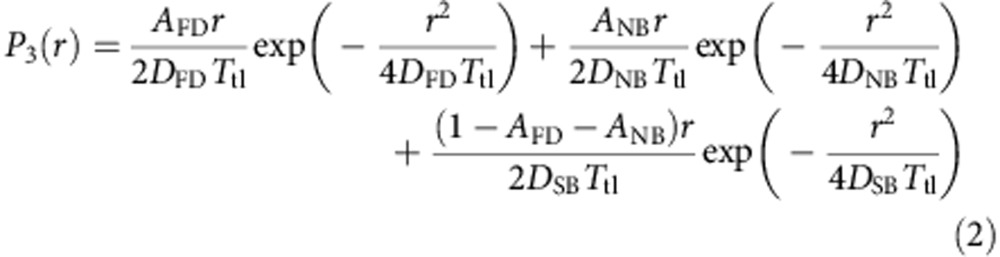




The approach of using a linear combination of diffusion terms in the CDF (or the corresponding PDF, Fig. 2b) of *r* was also applied previously by others to analyse SMT results and resolve multiple diffusion states of a protein in *E. coli* or mammalian cells^1^,^2^,^24^,^27^,^32^,^35^,^59^,^60^,^61^. This linear combination approach assumes an approximation of a quasi-static system, that is, interconversion between the diffusion states is slower than the experimental time resolution (see justification of this approximation for CueR or ZntR in Supplementary Note 18.4).

In the global fit, the effective diffusion constants (that is, *D*_FD_, *D*_NB_ and *D*_SB_) were expected to be concentration independent and thus were shared across the cellular protein concentrations, while their fractional populations (that is, *A*_FD_, *A*_NB_ and *A*_SB_=1–*A*_FD_–*A*_NB_) were allowed to differ (Supplementary Note 11.2). This global fitting was critical for the reliability of determining the minimal number of diffusion states, and their fitted diffusion constants and fractional populations, as compared with fitting the CDFs individually. Exemplary fitted fractional populations versus cellular protein concentrations are shown in Fig. 2c. The extraction of the number of diffusion states and their effective diffusion constants was validated through multistate diffusion simulations within the confined cell geometry, followed by the quantitative analysis of the simulated results in parallel to analysis of the experimental results (Supplementary Note 15).

### Determination of 
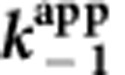
 from chromosomal recognition sites

A three-state kinetic model (Fig. 2e) was used to analyse the distributions of the microscopic residence time *τ*, thresholded from the displacement-versus-time trajectory to extract the apparent unbinding rate constant 
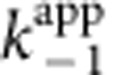
of mEos3.2-tagged regulators from chromosomal recognition sites (Supplementary Note 14). The three states are the freely diffusing CueR or ZntR proteins in the cytoplasm, nonspecifically bound to DNA and specifically bound to chromosomal recognition sites. This kinetic model includes the reversible binding/unbinding of a regulator to chromosomal recognition sites and nonspecific sites, as well as the photobleaching/blinking of the mEos3.2 tag, but the direct interconversion between the SB and the NB states is assumed to be sufficiently slow to be kinetically negligible (justification and validation of this approximation in Supplementary Note 14.5).

With a given displacement threshold *r*_0_, the effective diffusion constants (*D*’s), the unbinding rate constant (*k*_−2_) from the NB state, the apparent unbinding rate constant (
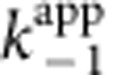
) from the SB state and the photobleaching/blinking rate constant *k*_bl_, we derived the respective probability distribution functions *φ*(*τ*) of the thresholded residence time *τ* for the FD, NB and SB states (that is, *φ*_FD_(*τ*), *φ*_NB_(*τ*) and *φ*_SB_(*τ*), respectively; Supplementary Note 14.1):




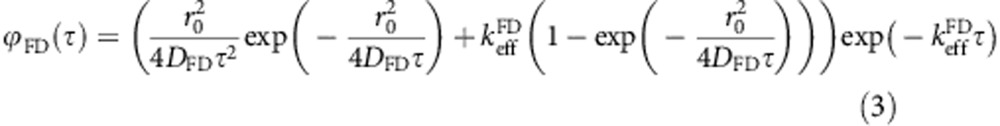







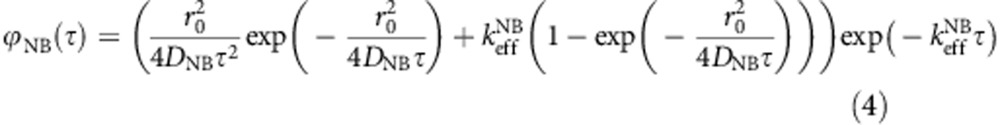







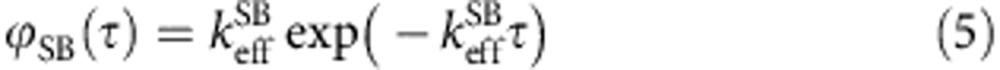




where 
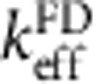
, 
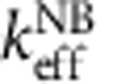
 and 
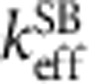
 are the rate constants that account for the unbinding of a mEos3.2-tagged regulator from chromosome and/or mEos3.2 photobleaching/blinking. 
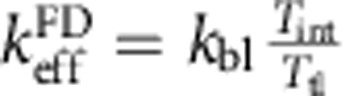
, 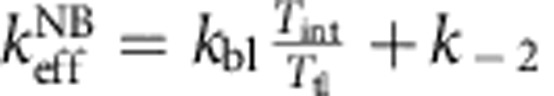 and 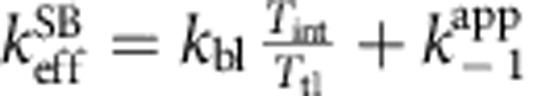. With the fractional populations of FD, NB and SB states (that is, *A*_FD_, *A*_NB_ and *A*_SB_) extracted from the global CDF analysis, at any cellular protein concentration, the overall probability distribution function of *τ*, *φ*(*τ*)_all_, is:




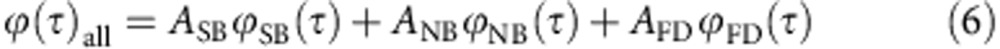




Here we independently determined *k*_bl_ by analysing the distribution of length of the tracking trajectories (Supplementary Note 10). The unbinding rate constant *k*_−2_ from nonspecific sites was extracted by fitting the residence time distribution with equation (7) at the highest cellular protein concentration (for example, 1,375 nM for 
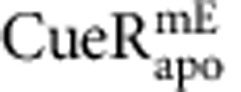
), where *A*_SB_ is ≤5% and *A*_SB_*φ*_SB_(*τ*) in equation (6) can be neglected and *k*_−2_ became the only floating parameter:




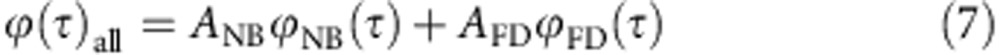




For any other cellular protein concentration with *A*_SB_>5%, the residence time distribution was fitted with equation (6), with predetermined *D*’s, *A*’s, *k*_bl_ and *k*_−2_, and the only floating parameter was 
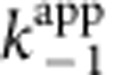
 (for example, Fig. 2f). As both CueR and ZntR have many recognition sites and nonspecific binding sites in the *E. coli* chromosome (Supplementary Note 22), all determined rate constants here represent the average properties over all the possible sites.

The dependence of 
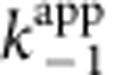
 on the cellular concentration of the freely diffusing proteins (that is, [P]_FD_) was then fitted with the linear function (for example, Fig. 2g):




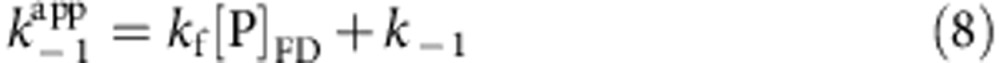




where *k*_f_ is a second-order rate constant for facilitated unbinding and *k*_−1_ is the first-order rate constant for spontaneous unbinding from the recognition sites. And, [P]_FD_=[P]_cell_*A*_FD_, where [P]_cell_ is the total cellular concentration of CueR or ZntR.

The entire procedure of extracting the unbinding rate constants from residence time distributions was also validated by simulations of three-state diffusion processes in confined cell geometry with variable interconversion rates (Supplementary Note 15.3). The results were further corroborated by hidden Markov model analysis of single-particle tracking trajectories using the vbSPT (variational Bayes Single Particle Tracking) software^62^ (Supplementary Note 16).

### Population analysis of different states in the cell

The same three-state kinetic model also allowed us to analyse the relative populations of FD, NB and SB states of CueR or ZntR across all cellular protein concentrations. This analysis uses a quasi-equilibrium approximation, which assumes that a CueR or ZntR molecule can sample these three states rapidly relative to its cellular lifetime and thus all binding and unbinding are at equilibrium (justification and validation in Supplementary Note 14.3). Under this approximation, relative concentrations of the proteins at these three states can be related to the kinetic parameters in the model as in equations (9, 10, 11) (Supplementary Note 14.3). Here [P]_FD_, [PD]_NB_ and [PD]_SB_ are the cellular concentrations of freely diffusing CueR or ZntR proteins, proteins nonspecifically bound to DNA and proteins specifically bound chromosomal recognition sites, respectively. And, [P]_FD_=[P]_cell_*A*_FD_, [PD]_NB_=[P]_cell_*A*_NB_ and [PD]_SB_=[P]_cell_*A*_SB_.




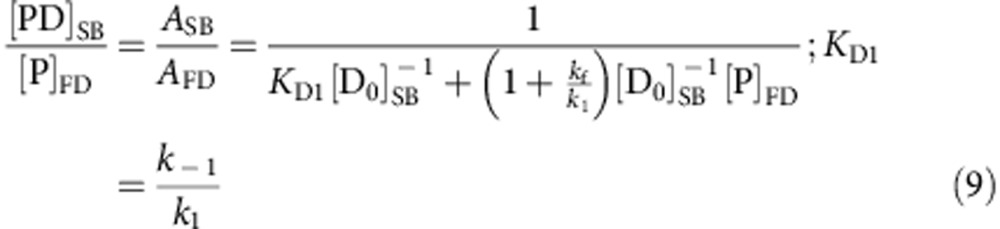







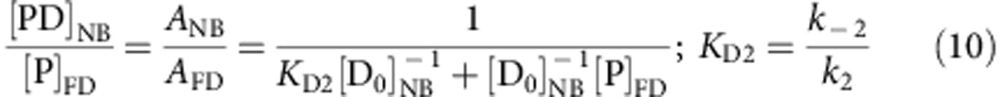







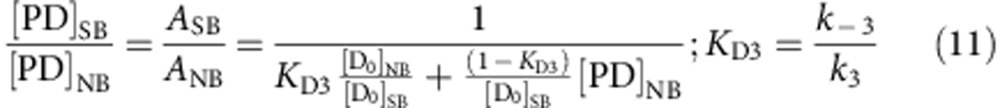




Here *k*_1_ and *k*_2_ are the binding rate constants to the recognition sites and nonspecific sites, respectively. *k*_3_ and *k*_−3_ are the interconversion rate constants between the SB and NB states, which are approximated to be sufficiently small to be kinetically negligible as mentioned above (validations in Supplementary Note 14.5). [D]_SB_, [D_0_]_SB_, [D]_NB_ and [D_0_]_NB_ are the effective cellular concentrations of vacant chromosomal recognition sites, total chromosomal recognition sites, vacant nonspecific binding sites and total nonspecific binding sites, respectively. Using *k*_−2_, *k*_−1_ and *k*_f_ from the residence time analysis described earlier, we can fit *A*_SB_/*A*_FD_ versus [P]_FD_, *A*_NB_/*A*_FD_ versus [P]_FD_, *A*_SB_/*A*_NB_ versus [PD]_NB_ with equations (9, 10, 11) as in Supplementary Fig. 30 to obtain the dissociation constants of SB (*K*_D1_) and NB (*K*_D2_) states together with *k*_2_, [D_0_]_NB_, *k*_1_ and [D_0_]_SB_ (Table 1 and Supplementary Tables 7 and 8). The determined dissociation constants (*K*_D_’s) at the recognition sites and nonspecific sites (tens and hundreds of nM, respectively) are consistent with literature^12^,^21^,^33^, further supporting the validity of our analyses. An interesting note here: for both CueR and ZntR, their affinity differences between recognition and nonspecific sites mainly come from kinetic differences in binding rather than unbinding (Supplementary Note 18.2).

## Additional information

**How to cite this article:** Chen, T.-Y. *et al*. Concentration- and chromosome-organization-dependent regulator unbinding from DNA for transcription regulation in living cells. *Nat. Commun.* 6:7445 doi: 10.1038/ncomms8445 (2015).

## Supplementary Material

Supplementary Information Supplementary Figures 1-56, Supplementary Tables 1-13, Supplementary Notes 1-22 and Supplementary References

## Figures and Tables

**Table 1:** Kinetic and thermodynamic parameters for CueR−DNA interactions in *E. coli* cells*.

**Parameters**	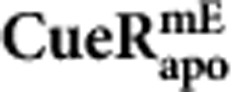	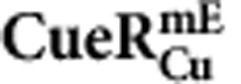	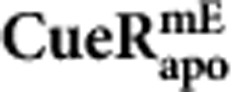 **(dividing cells)**
*k*_1_ (μM^−1^ s^−1^)	214±46	54±96	219±77
*k*_−1_ (s^−1^)	8.2±0.9	4.6±0.8	6.4±1.1
*k*_f_ (μM^−1^ s^−1^)	31.9±6.9	55.0±8.5	20.1±8.7
*K*_D1_(*k*_−1_/*k*_1_) (μM)	0.037±0.028	0.038±0.058	0.029±0.011
*k*_2_ (μM^−1^ s^−1^)	3.6±1.9	4.9±3.8	1.6±6.1
*k*_−2_ (s^−1^)	2.5±0.1	4.1±0.1	2.6±0.2
*K*_D2_(=*k*_−2_/*k*_2_) (μM)	0.69±0.38	0.83±0.65	1.6±6.3
*K*_D3_(=*k*_−3_/*k*_3_)	0.06±0.03	0.04±0.05	0.02±0.08
*N*_NB_	2,605±1,381	2,588±1,976	8,941±34,406
*N*_SB_	130±17	121±89	153±36

^*^See Fig. 2e for definition of kinetic parameters. *N*_NB_ and *N*_SB_ are the effective numbers of specific recognition sites and nonspecific binding sites on the chromosome. All error bars are s.d. Relevant data for ZntR variants and other control strains are in Supplementary Tables 7 and 8.
