# Supplementary material for: Concentration- and chromosome-organization-dependent regulator unbinding from DNA for transcription regulation in living cells
Source: Nat Commun. 2015 Jul 6;6:7445. doi: 10.1038/ncomms8445 (PMC4507017; doi:10.1038/ncomms8445)
Supplement: Supplementary Information — Supplementary Figures 1-56, Supplementary Tables 1-13, Supplementary Notes 1-22 and Supplementary References [file ncomms8445-s1.pdf]

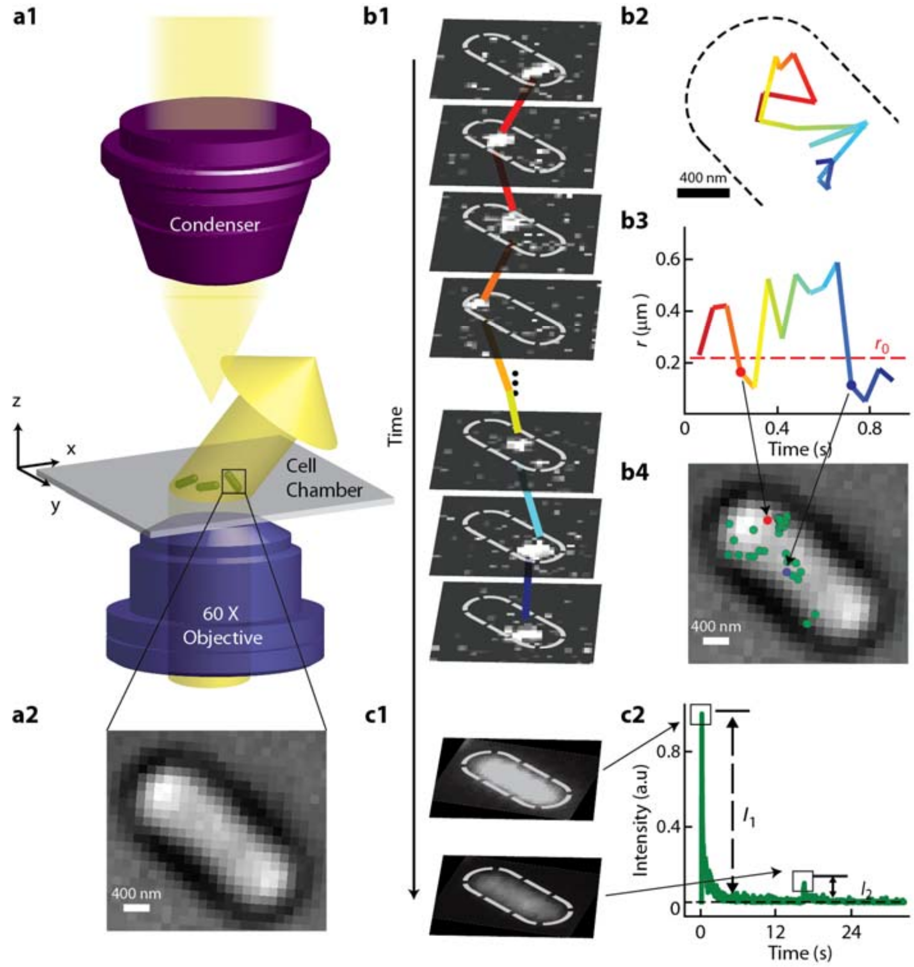

Supplementary Figure 1. Schematic overview of single-molecule tracking to quantify regulator unbinding kinetics from chromosome in living *E. coli* cells and data analysis procedures. (**a1-a2**) Experimental scheme using single-molecule fluorescence microscopy with lasers under inclined epi-illumination and a cell chamber (**a1**) to image diffusion and binding/unbinding of proteins to/from chromosome in individual living *E. coli* cells. The boundary of each cell is determined from its transmission image (**a2**). See Section 5 for details. (**b1-b4**) Single-molecule tracking (SMT) using time-lapse stroboscopic imaging of single proteins tagged with photoconverted fluorescent proteins (**b1**). The centroid of the fluorescent protein in each time-lapse image can be localized to nanometer accuracy using two-dimensional Gaussian fitting of the fluorescence image. Connecting all centroids across images results in a position trajectory (**b2**), which can then be used to compute the corresponding displacement trajectory (**b3**). Microscopic residence times can then be determined with displacement threshold r_0 (**b3**). See Sections 5 and 6 for details. Overlaying all residence sites associated with the residence times on top of the cell transmission image maps out the spatial organization of chromosome, which is further quantified by the average pairwise distance between the residence sites (**b4**). See Section 20 for details. (**c1-c2**) The protein concentration of each cell is determined via single-cell quantification of protein concentration (SCQPC), in addition to the single-molecule counting during SMT. SCQPC is performed right after the SMT step, where the whole-cell fluorescence intensity of tagged proteins (**c1**) is measured repeatedly until all remaining mEos3.2 molecules are photobleached. The overall intensity (I_1+I_2; (**c2**)) is then divided by the average fluorescence intensity of a single tag molecule in the same cell to obtain the number of protein molecules that were not tracked individually. See Section 5 for details.

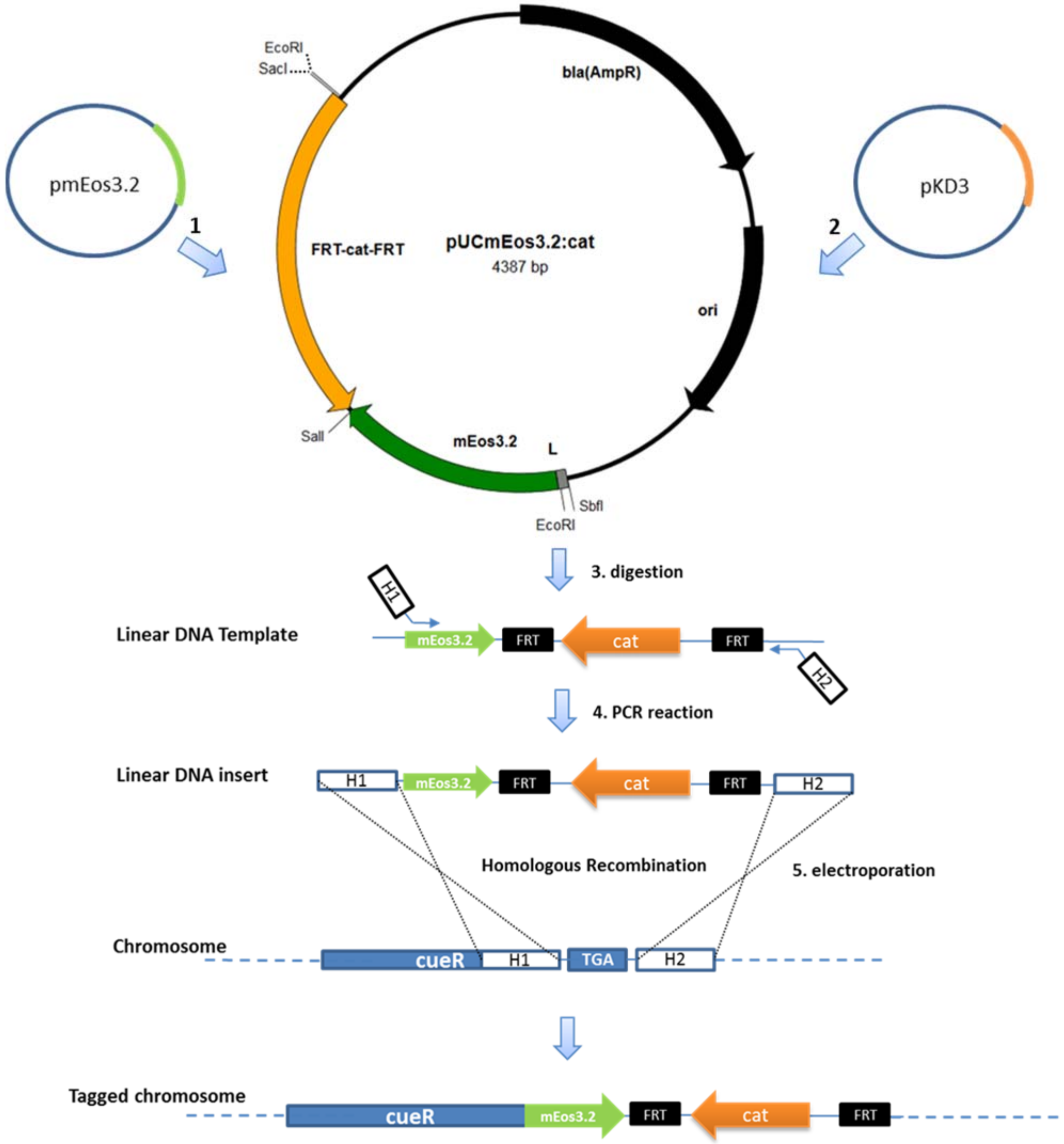

Supplementary Figure 2. Major procedures of using λ-Red technique[1-3] to tag chromosomal genes in *E. coli*. (1) The *mEos3.2* gene was first amplified from plasmid pmEos3.2 and inserted into a pUC19 vector giving pUCmEos3.2. (2) This was followed by the insertion of the *cat* gene (chloramphenicol acetyltransferase conferring chloramphenicol resistance) cassette amplified from a pKD3 plasmid giving pUCmEos3.2:cat. (3) The plasmid pUCmEos3.2:cat was digested to obtain a linear DNA cassette containing *mEos3.2* and *cat* genes. (4) Using this template, a PCR reaction was performed to obtain the linear double stranded DNA insert 5′-H1-mEos3.2:cat-H2-3′. The H1 in the linear DNA insert is a 40-bp DNA, which is homologous to the last 40 bp of the target gene (e.g., *cueR*) excluding the stop codon (TGA in *cueR*), while H2 is a 41-bp DNA, which is homologous to the next 41 bp right after the stop codon of the target gene. We excluded in the linear DNA insert design the stop codon of the target gene so that *mEos3.2* will be expressed as a C-terminal fusion protein. (5) The linear DNA insert was electroporated into *E. coli* cells harboring the pKD46 plasmid that expresses the recombinase enzymes (*exo*, β, γ). Once the linear DNA insert entered the cell, these enzymes integrate the foreign linear DNA into the target site in the chromosome, ensuring the target gene being tagged at the C-terminal with *mEos3.2*, alongside with the *cat* gene. The *cat* gene is flanked by the Flippase Recognition Target (FRT) sequence (i.e., FRT-cat-FRT), which is a necessary element for the removal, if desired, of the *cat* cassette in the chromosome using Flippase (FLP) enzyme-mediated removal technique [1]. The 30-bp linker (L) adjacent to the mEos3.2 gene in the pUCmEos3.2:cat map could be used for making fusion proteins in which mEos3.2 is attached to the C-terminal of the protein of interest connected by the 10-amino acid linker (AGSAAGSGEF) encoded by L; this linker is not used for all constructs made in the current study. The plasmid pUCmEos3.2:cat map was made using an online program accessible at http://www.bioinformatics.org/savvy/.

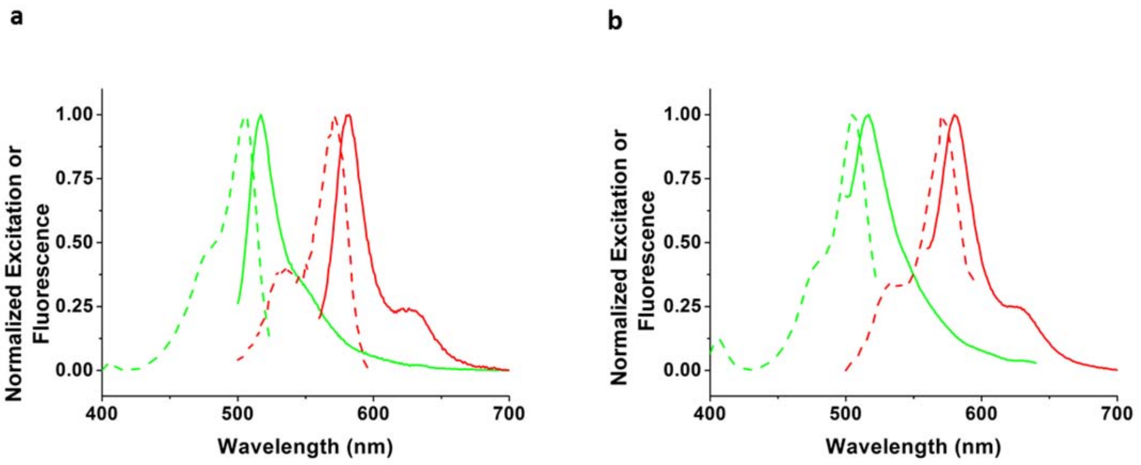

Supplementary Figure 3. Ensemble emission (solid lines) and excitation (dashed lines) spectra of CueR^{mE} (**a**) and ZntR^{mE} (**b**) in both the green (green lines) and red forms (red spectra) of mEos3.2 in living *E. coli* cells. All fluorescence intensities are normalized to 1 at the peak.

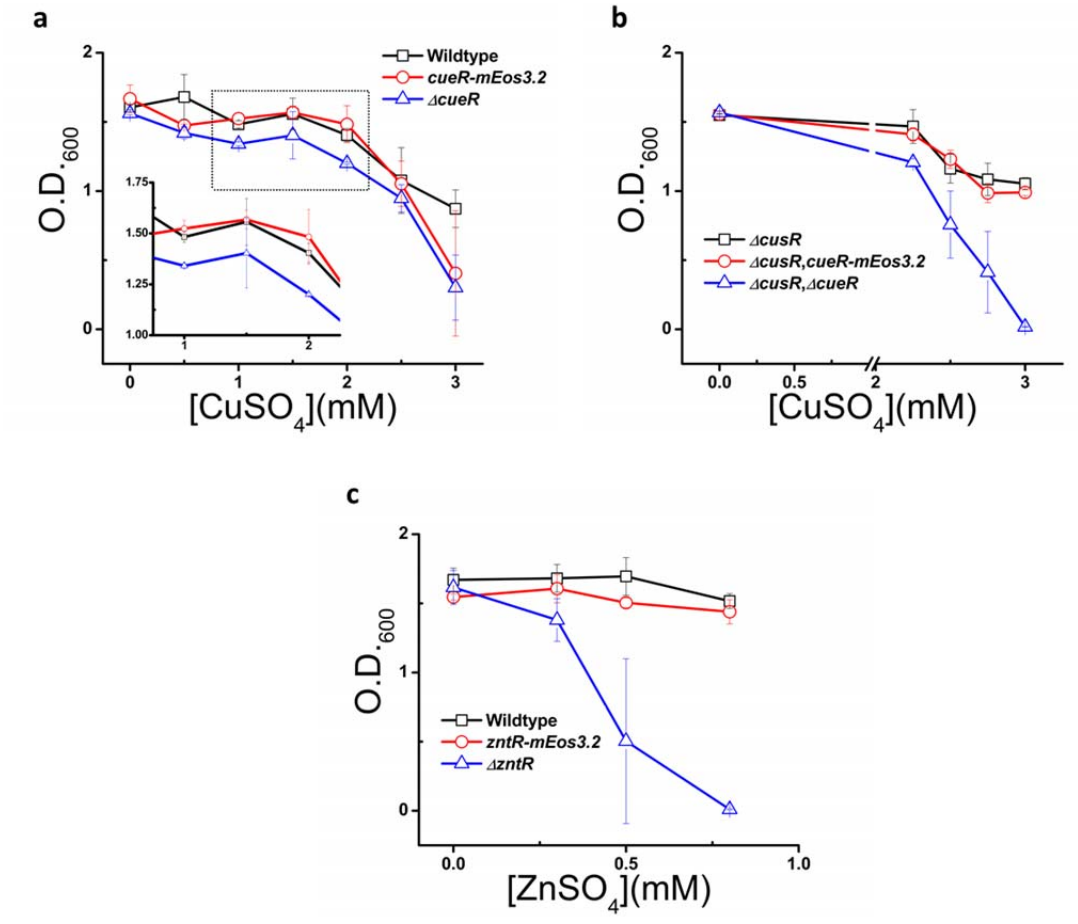

Supplementary Figure 4. Metal tolerance cell growth assays to test the functionality of mEos3.2-tagged CueR (**a, b**) and ZntR (**c**). For **a**, the inset shows the plot within the rectangular dotted box. Data were collected in triplicates. Error bars are standard deviations.

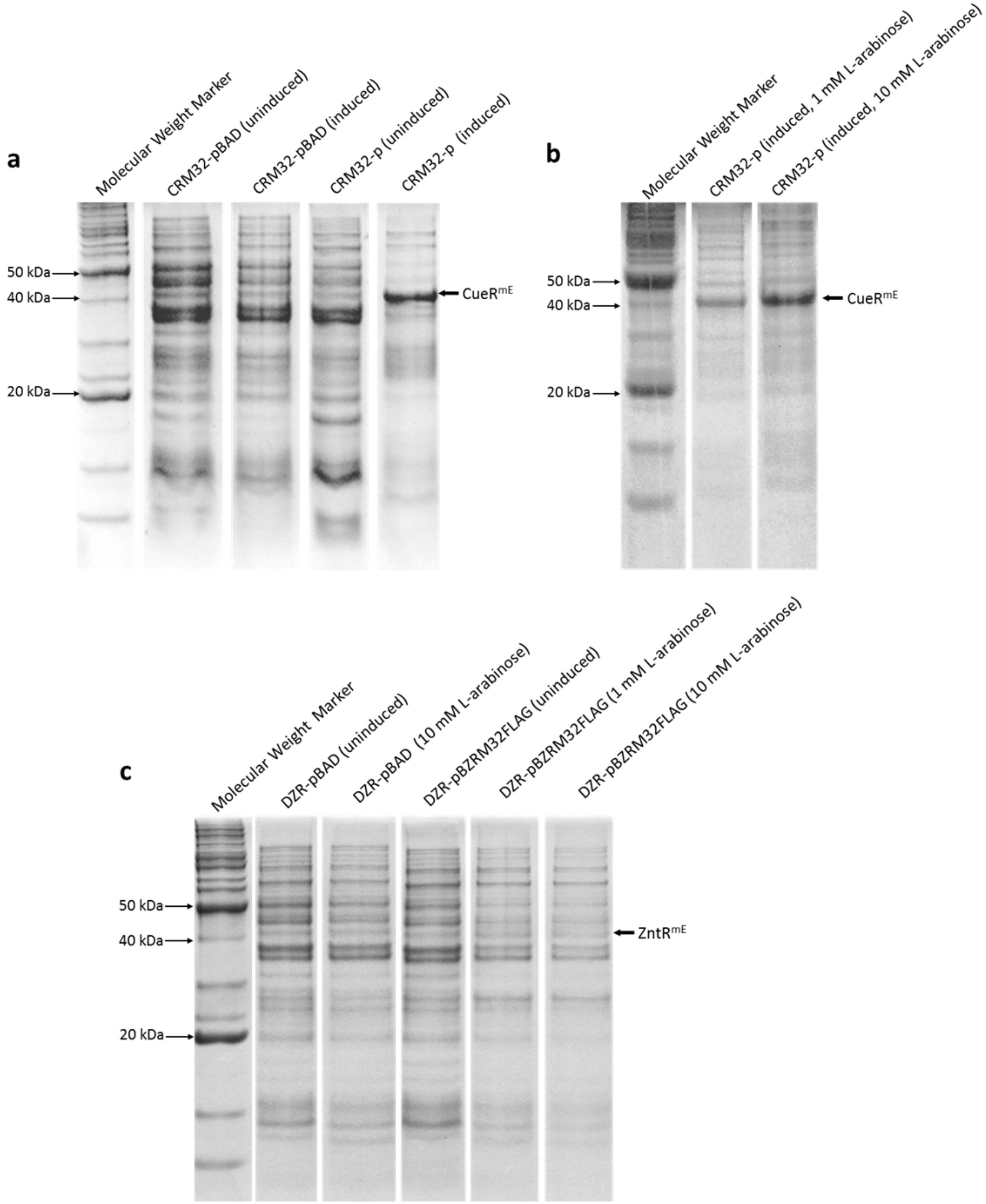

Supplementary Figure 5. Coomassie Blue stained SDS-PAGE showing the overexpression of CueR^{mE} from pBAD24 plasmid (strain CRM32-p) upon induction with 1 mM L-arabinose together with the negative control strain, CRM32-pBAD, which carries the empty pBAD24 plasmid (a). Comparison of the expression profiles of CueR^{mE} under different L-arabinose concentrations (b). Corresponding SDS-PAGE data for the strain expressing ZntR^{mE}-FLAG from pBAD24 (DZR-pBZRM32FLAG) together with the negative control DZR-pBAD, which carries the empty pBAD24 plasmid.

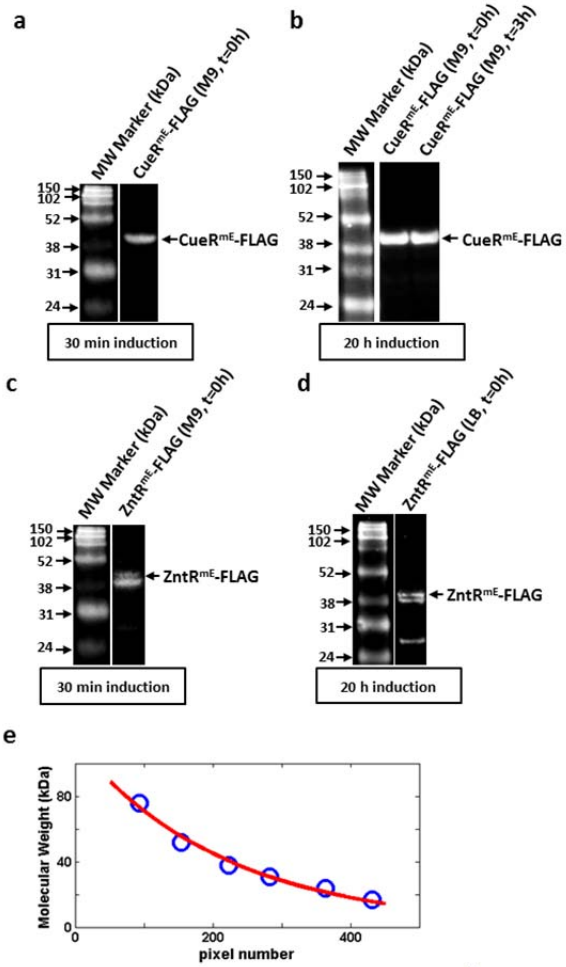

Supplementary Figure 6. Western blot analysis of $CueR^{mE}$-FLAG (**a, b**) and $ZntR^{mE}$-FLAG (**c, d**) expression in cells with different expression induction times and different time delay after stopping induction. $t = 0$ h or $t = 3$ h refers to the time delay after induction was stopped. (**e**) Molecular weight calibration curve from the gel image of MW markers in (d). The blue circles are points from the MW markers. The solid red line is a double-exponential fit.

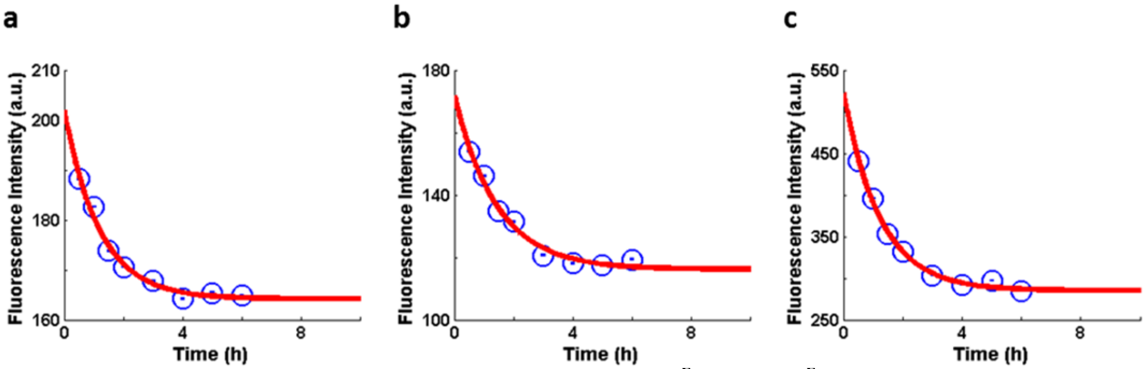

Supplementary Figure 7. Fluorescence vs. time for cells expressing $CueR^{mE}$ (a), $ZntR^{mE}$ (b), and mEos3.2 (c). Cells were grown in M9 to $OD_{600} = 0.3$ and induced for 18 h. Induction was stopped by re-suspending the cells in M9 medium containing glucose. The green fluorescence of the cells was measured at different time points after stopping induction, excited at $\lambda = 465$ nm and detected at $\lambda = 556$ nm, which is the emission maximum of the green form of mEos3.2. The decay rate for each plot was calculated from a single-exponential fit to the data, giving, for $CueR^{mE}$, $ZntR^{mE}$, and mEos3.2, 0.84 ± 0.16 h^{-1}, 0.70 ± 0.15 h^{-1}, and 0.81 ± 0.09 h^{-1}, respectively.

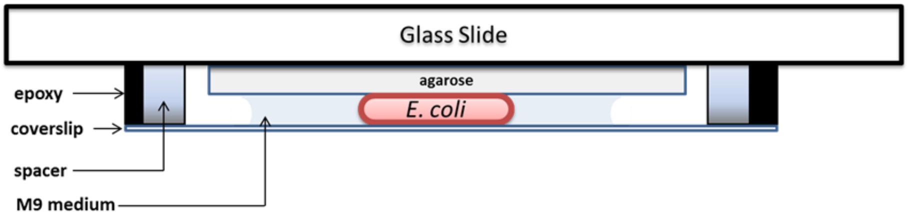

Supplementary Figure 8. Assembly of the sample cell used for fluorescence imaging. *E. coli* cells are immobilized on 3% agarose gel pad, facing the coverslip. The agarose gel pad is sandwiched between a coverslip and glass slide joined together by epoxy.

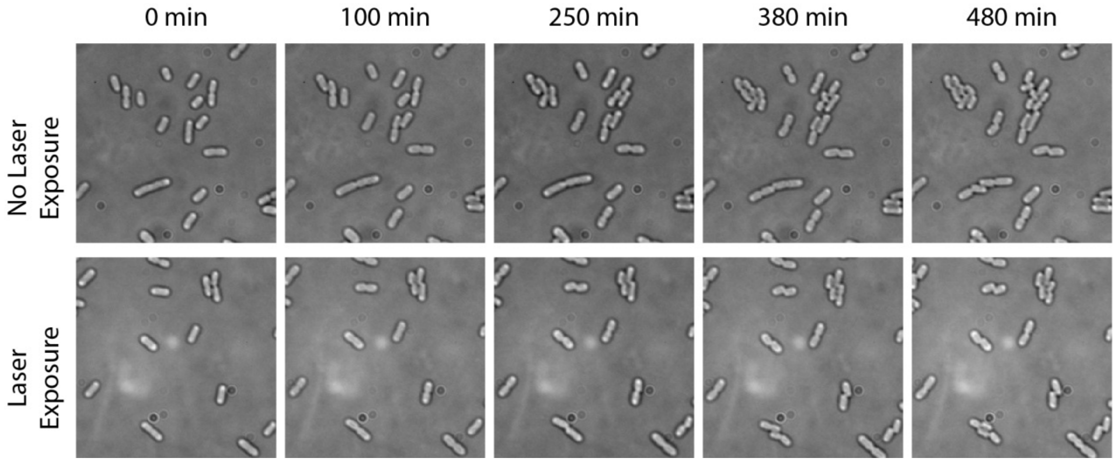

Supplementary Figure 9. Cell viability under our imaging conditions. The growth and division of *E. coli* cells (the strain that expresses $\text{CueR}^{\text{mE}}_{\text{apo}}$; DCR-pACRM32 in

Supplementary Table **3**) are monitored on the microscope at 20°C without (top row) or with (bottom row) laser exposure.

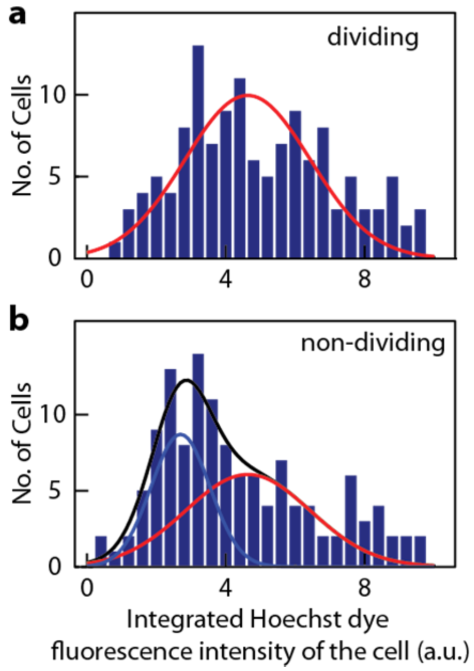

Supplementary Figure 10. Histograms of integrated fluorescence intensity of the Hoechst staining dye over the cells expressing CueR$^{\mathrm{mE}}_{\mathrm{apo}}$. (**a**) For dividing cells, for which the fluorescence intensity is integrated over both halves of a dividing cell and thus reflects the contribution from two copies of chromosome. (**b**) For non-dividing cells. The blue and red curves are Gaussian fittings, and the red curve fit is shared in a global fit between the two plots. The resolved two populations in (**b**) correspond to cells having one (blue curve) or two (or less than two for partially replicated chromosomes; red curve) copies of chromosomes.

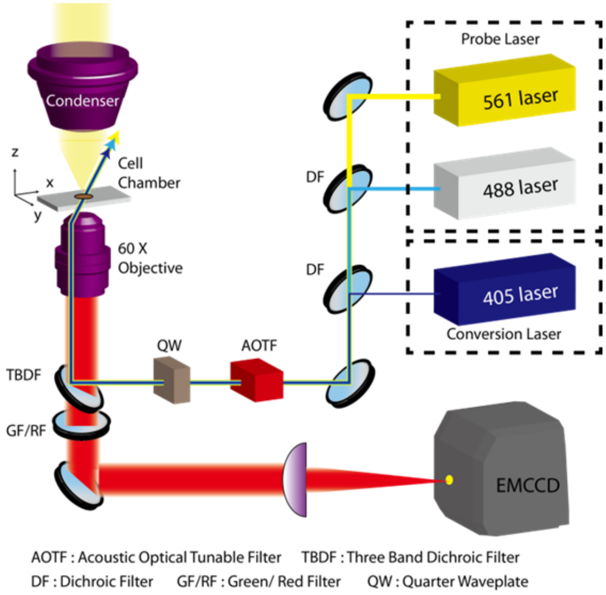

Supplementary Figure 11. Schematic diagram of microscope setup. An AOTF is synchronized with EMCCD camera and shutters lasers to generate short laser pulses for single-molecule stroboscopic imaging and single-cell quantification of protein concentration. For single-molecule stroboscopic imaging, the 405 nm laser photoconverts one mEos3.2 from green to red fluorescence form, which is tracked with short 561 nm laser pulses per tracking cycle. For single-cell quantification of protein concentration, the 405 nm laser photoconverts all mEos3.2 to red form and the 561 nm laser probes the fluorescence intensity of

the whole cell. The 488 nm laser can also be used to directly quantify protein concentration of each cell via the green form of mEos3.2, but it is complicated by the cell autofluorescence.

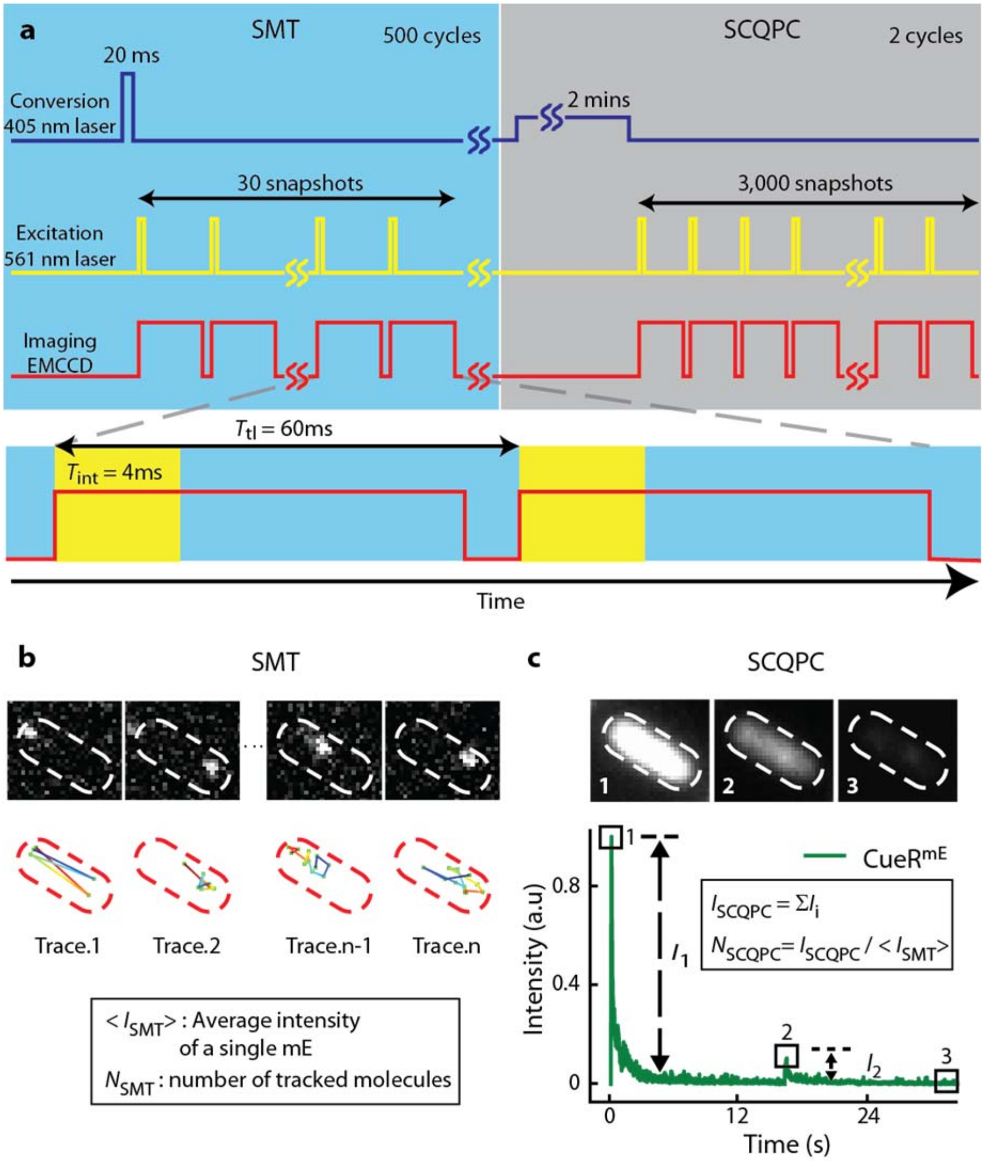

Supplementary Figure 12. Single-molecule tracking (SMT) with time-lapse stroboscopic imaging and single-cell quantification of protein concentration (SCQPC). (**a**) Laser and camera timing diagram for SMT and SCQPC. For SMT, each imaging cycle includes a 20-ms 405 nm laser pulse photoconverting one (or none) mEos3.2 to its red form followed by 30 imaging snapshots probed by the EMCCD-synchronized 561 nm laser pulses (pulse duration T_{int} = 4 ms) and with time lapse T_{tl} = 60 ms. This imaging cycle is then repeated for 500 times for each measurement. For SCQPC, once the SMT step is completed, a weak 405 nm laser illuminates the cell for two minutes to photoconvert the rest of green-mEos3.2 proteins to red-mEos3.2 followed by 561 nm laser imaging for 3,000 frames at the same laser power density and laser exposure time as done in the SMT step. The SCQPC step can be repeated multiple times until all the proteins inside the cell are photoconverted. (**b**) Top: exemplary single-molecule fluorescence images during the SMT step. Middle: exemplary position trajectories of each tracked mEos3.2-tagged protein molecule. The average fluorescence intensity of a single mEos3.2 ($\langle I_{SMT} \rangle$) in each image is extracted from SMT images. Concurrently we have the number of the tracked mEos3.2-tagged molecules N_{SMT}. (**c**) Top: exemplary whole cell red fluorescence images during the SCQPC step. Lower: exemplary whole cell red fluorescence intensity in each image vs. time for a cell that expresses CueR^{mE}. $\langle I_{SMT} \rangle$ determined from the SMT step (**b**) is used to quantify the number of remaining mEos3.2 by dividing the fluorescence intensity of the whole cell with the average intensity of single mEos3.2 ($I_{SCQPC}/\langle I_{SMT} \rangle$). The total number of mEos3.2 detected is equal to the sum of N_{SMT} and N_{SCQPC}, which is further corrected by the protein oligomerization state and the photoconversion efficiency of mEos3.2 to obtain the copy number of mEos3.2-tagged CueR or ZntR in each cell.

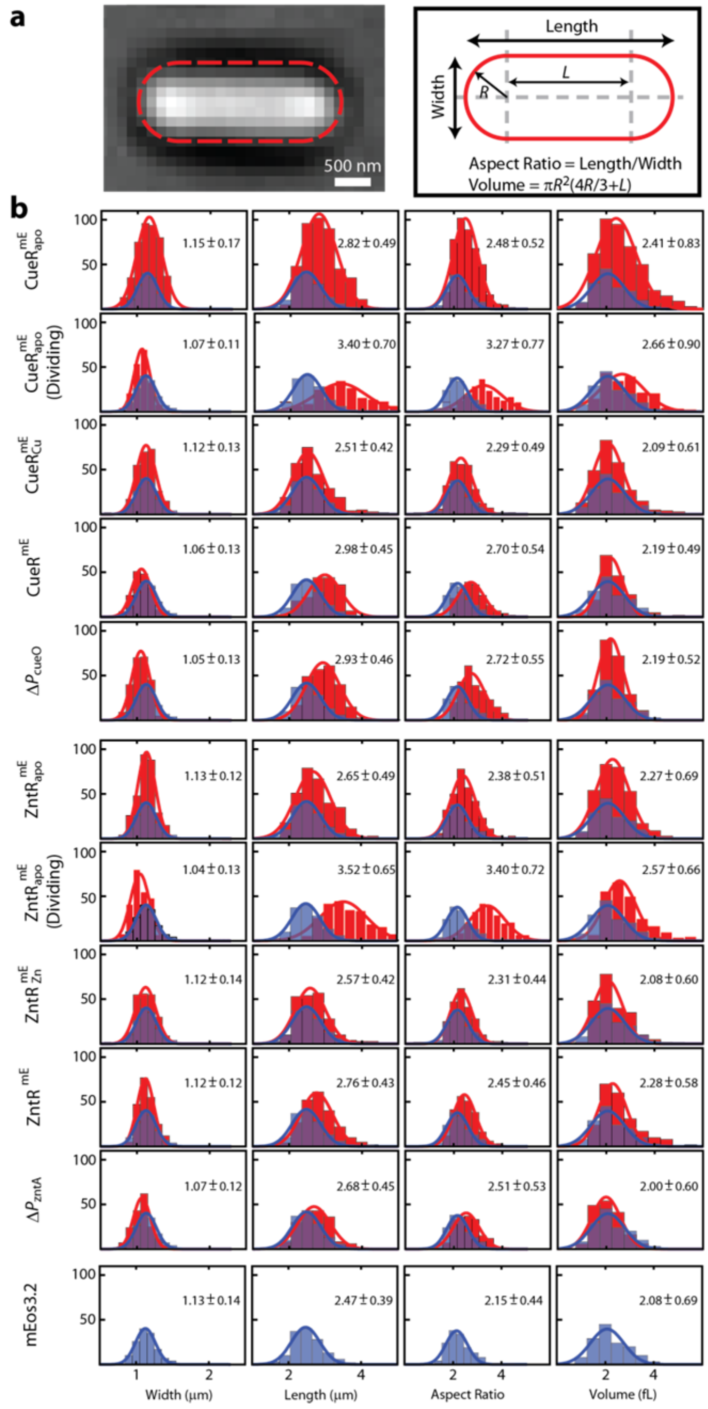

Supplementary Figure 13. Distribution of cell geometric parameters across all experimental conditions. (**a**) Transmission image (left) of each cell is fitted with the model (right) of a cylinder with two hemispherical caps to get the quantitative information on the cell width, length, aspect ratio and volume. (**b**) Histograms of cell geometric parameters for the cell strains under different experimental conditions (red) versus the cells that express merely free mEos3.2 (blue). The cells were imaged in metal-depleted medium except for those expressing $\mathbf{CueR^{mE}_{Cu}}$ or $\mathbf{ZntR^{mE}_{Zn}}$ which were imaged in the presence of 100 μM $[Cu^{2+}]/[Zn^{2+}]$. Each histogram is fitted with a Gaussian distribution function; the average and distribution (center position ± standard deviation) of geometric parameter is denoted in each histogram panel.

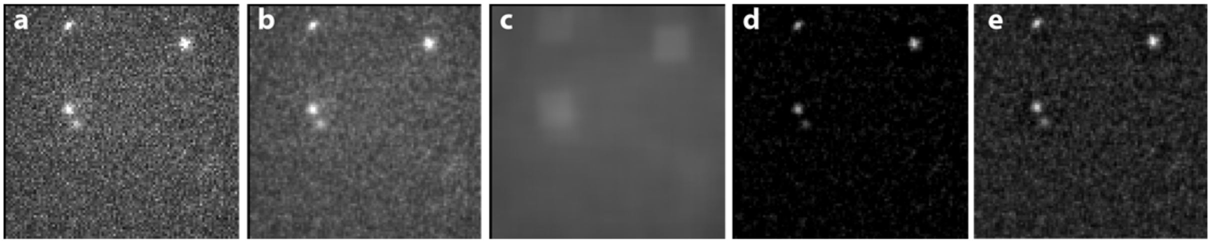

Supplementary Figure 14. Exemplary fluorescence image processing procedure for spot localization of single CueR^{mE} molecules. (**a**) Original fluorescence image. (**b**) Low-pass Gaussian-smoothed image of (**a**). (**c**) Non-uniform background estimated from (**b**) using boxcar kernel. (**d**) Final image for spot localization generated by subtracting (**c**) from (**b**). (**e**) The background-corrected image for 2-D Gaussian fitting of the fluorescence PSF of individual fluorescent molecules, generated by subtracting (**c**) from (**a**).

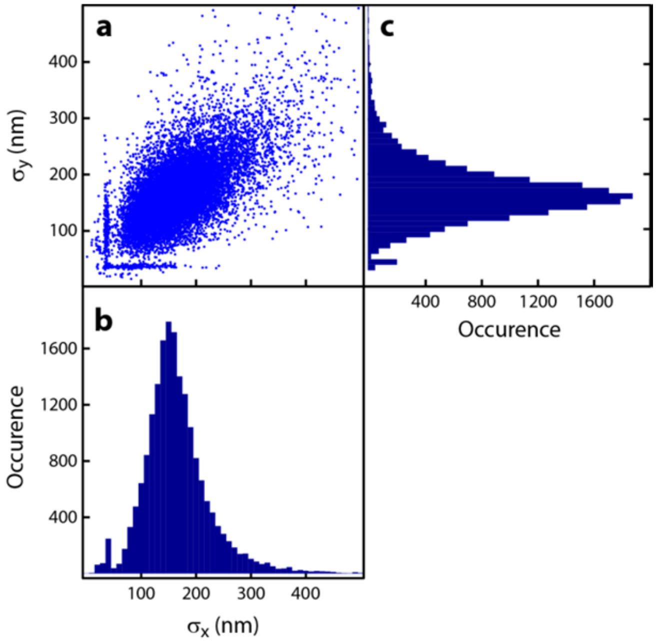

Supplementary Figure 15. Distribution of standard deviation of the Gaussian function fit. (**a**) Scatter plot of σ_x and σ_y from the 2D Gaussian fitting of the PSF of single mEos3.2 molecules in fixed *E. coli* cells. (**b, c**) Distributions of σ_x and σ_y from (**a**).

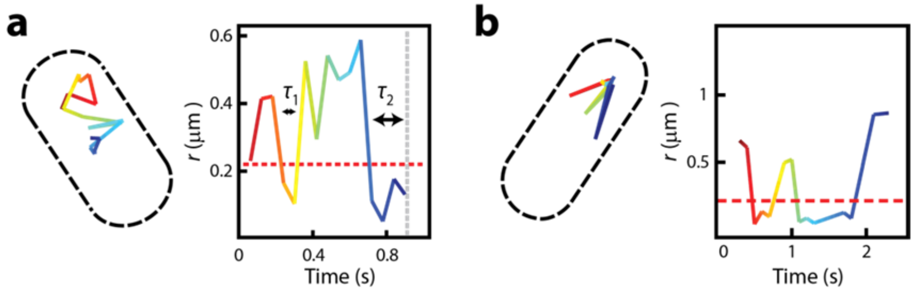

Supplementary Figure 16. Exemplary position and displacement trajectories. (**a**) Position (left) and corresponding displacement trajectory (right) of a single CueR$^{mE}_{apo}$ molecule. The horizontal red dash line indicates the threshold r_0 = 220 nm. Two residence times τ are denoted here. The vertical gray dash line indicates a photobleaching event. These two panels are the same as part of Fig. 1a and b in the main text. (**b**) Position and displacement trajectories of a single ZntR$^{mE}_{apo}$ molecule.

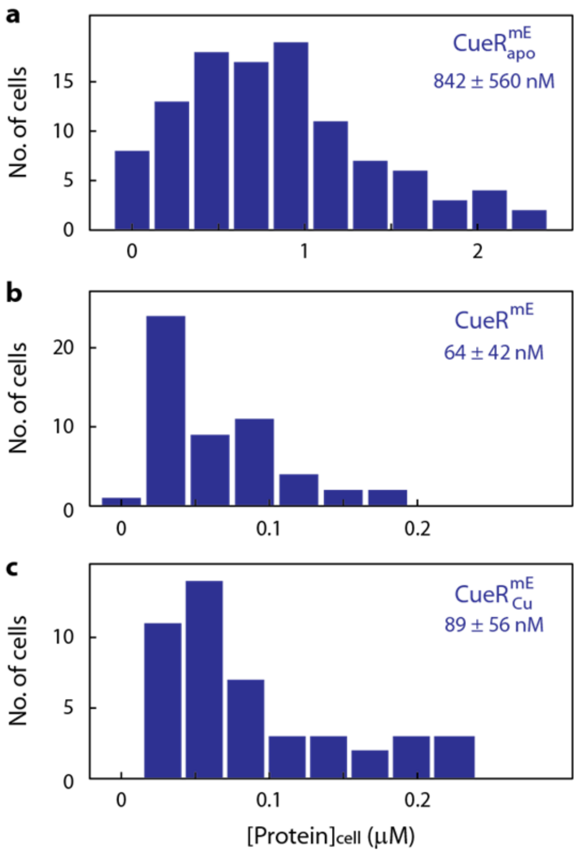

Supplementary Figure 17. Distribution of protein concentrations among individual cells at different growth conditions, using CueR constructs as examples. (**a**) Distribution of total cellular $CueR^{mE}_{apo}$ concentration among cells from a single growth after 30 minute induction with L-arabinose in metal-depleted M9 medium with vitamins and amino acids. (**b**) Distribution of cellular $CueR^{mE}$ concentration of single cells at basal expression from the chromosome without copper stress in the medium. (**c**) Same plot as (b) but with copper stress (100 μM) in the medium. The average concentrations ± standard deviations are denoted in each panel.

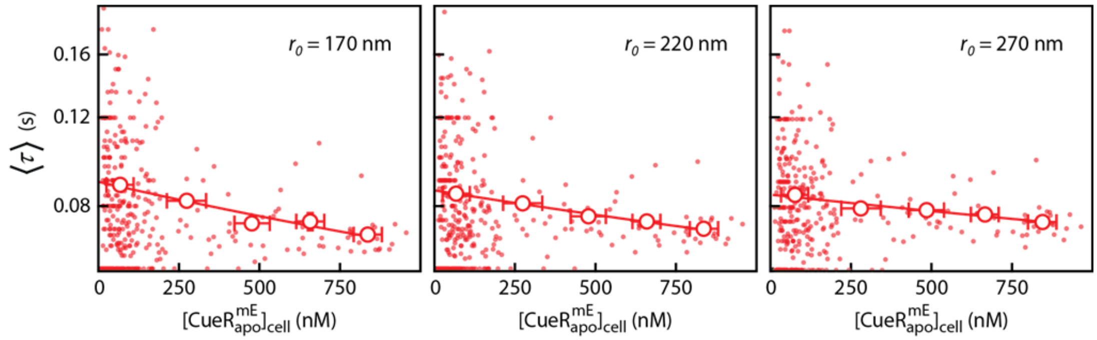

Supplementary Figure 18. Cellular protein-concentration-dependent average residence time $\langle\tau\rangle$ of $CueR^{mE}_{apo}$ with different r_o thresholds. Here r_0 = 170, 220, and 270 nm, respectively, plotted in log-linear scale. Each red dot represents the average residence time measured for one cell. We further bin the cells into groups of every 200 nM cellular protein concentration, and determined the dependence of $\langle\tau\rangle$ on the total cellular protein concentration (red open circle). The error bars of concentration and $\langle\tau\rangle$ of each binned data point are estimated from the standard deviation and standard error of the mean respectively. The solid lines are fits with Supplementary Equation **5**.

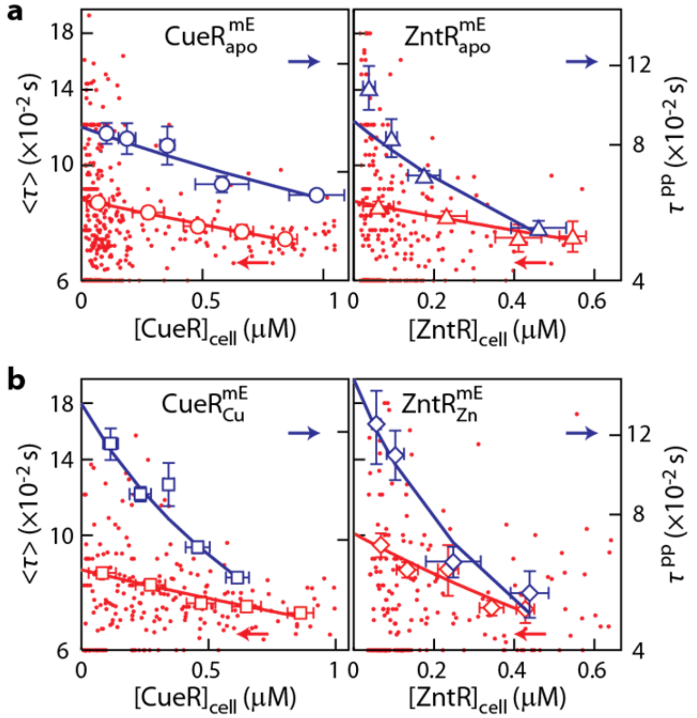

Supplementary Figure 19. (**a**) Correlation of $\langle\tau\rangle$ and protein concentration in each cell for $\mathrm{CueR}^{\mathrm{mE}}_{\mathrm{apo}}$ and $\mathrm{ZntR}^{\mathrm{mE}}_{\mathrm{apo}}$ (red dots) from a total of ~250 cells each. The individual cells are then grouped every 200 nM by their cellular protein concentrations and averaged within each group to obtain the dependence of $\langle\tau\rangle$ on cellular protein concentration (red circles and triangles for $\mathrm{CueR}^{\mathrm{mE}}_{\mathrm{apo}}$ and $\mathrm{ZntR}^{\mathrm{mE}}_{\mathrm{apo}}$, respectively). Further correction with the photophysics of mEos3.2 gives the corresponding τ^{pp} (blue circles and triangles for $\mathrm{CueR}^{\mathrm{mE}}_{\mathrm{apo}}$ and $\mathrm{ZntR}^{\mathrm{mE}}_{\mathrm{apo}}$, respectively). (**b**) Same as (**a**) but for $\mathrm{CueR}^{\mathrm{mE}}_{\mathbf{Cu}}$ and $\mathrm{ZntR}^{\mathrm{mE}}_{\mathbf{Zn}}$, respectively. *y* error bars are s.e.m.; *x* error bars are s.d.

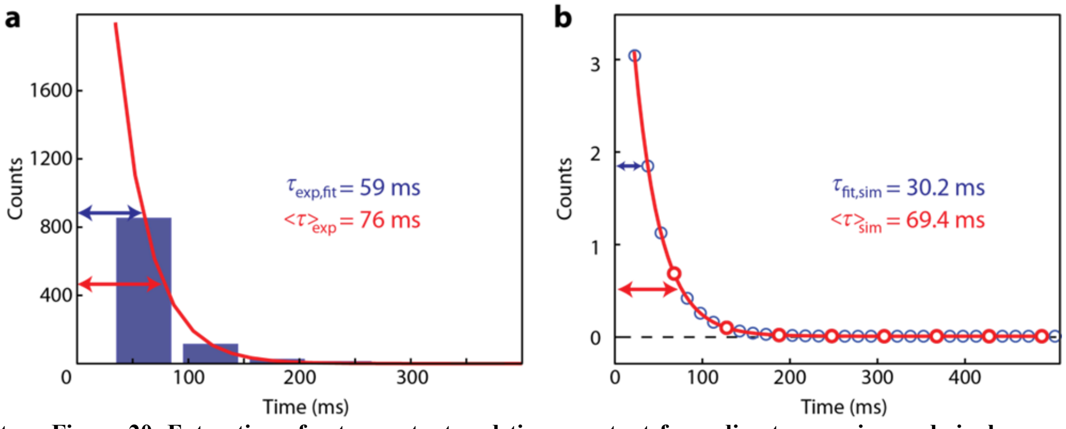

Supplementary Figure 20. Extraction of rate constant and time constant from direct averaging and single exponential fitting of distribution of τ, assuming single-step kinetic processes. (**a**) Residence time distribution of $\mathbf{CueR^{mE}_{apo}}$ effectively follows a single exponential function. The residence time estimated from direct averaging, $\langle\tau\rangle_{\mathrm{exp}}$, is always longer than the experimental time resolution of 60 ms, whereas the exponential fitting of the distribution, $\tau_{\mathrm{exp,fit}}$, gives the time constant (its inverse is the rate constant), which could be shorter than the experimental time resolution of 60 ms. (**b**) Simulated τ distribution data that follows a single exponential function with time constant of 30 ms and plotted every 15 ms in blue open circles. The simulated data is then sampled every 60 ms to mimic our experiment condition (red open circle). This sampled data are then used to calculate the straight average τ, $\langle\tau\rangle_{\mathrm{sim}}$, or fitted with the single exponential function (red curve) to extract the decay time constant, $\tau_{\mathrm{fit,sim}}$. Even though the decay time constant of 30 ms is twice faster than the time resolution, the $\tau_{\mathrm{fit,sim}}$ successfully extracted the correct decay time constant as simulated.

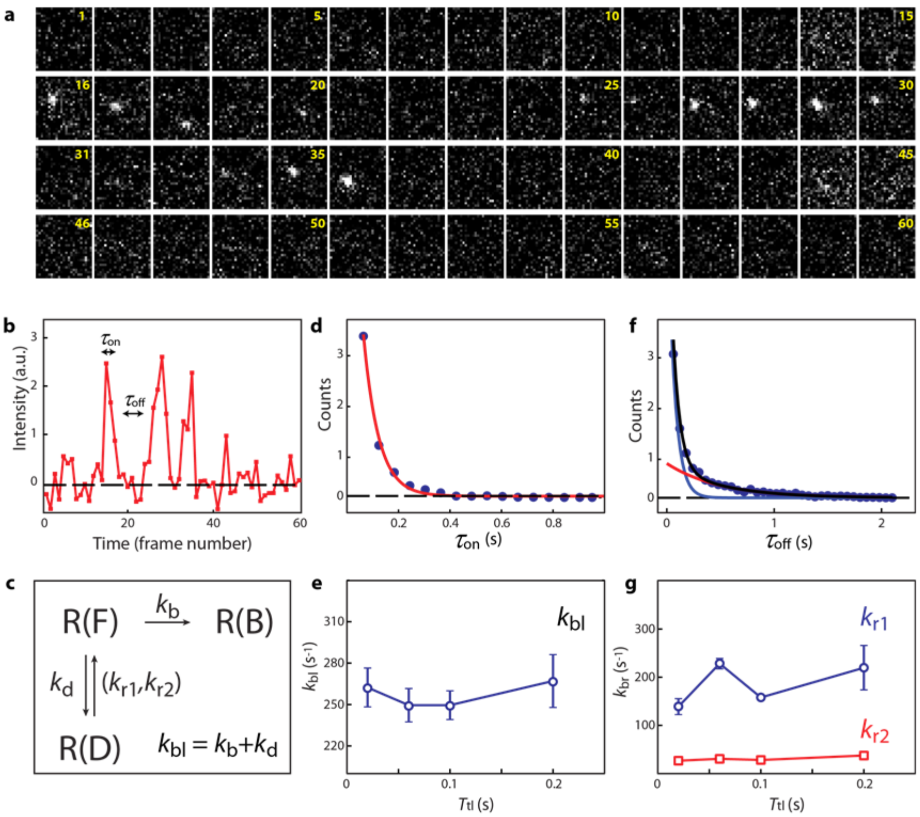

Supplementary Figure 21. Photoblinking/bleaching kinetics of mEos3.2. (**a**) Exemplary single-molecule tracking fluorescence images of a photoconverted mEos3.2 of CueR^{mE} in an *E. coli* cell. (**b**) Corresponding fluorescence intensity trajectory from (**a**). The intensity trajectory shows a clear on-off behavior due to photoblinking and eventually becomes permanently off due to photobleaching. (**c**) General kinetic model for photophysics of mEos2 as described by Lee *et al.* [36], and adapted for mEos3.2 here. Once the mEos3.2 is excited at the red fluorescent from R(F), it can go to the bleached R(B) or blinked R(D) states with rate constants k_b and k_d respectively. Once the mEos3.2 is at the R(D), it can recover from R(D) to R(F) with recovery rate constants k_{r1} and k_{r2}. The distribution of τ_{on} reports the photoblinking/bleaching rate k_{bl}, $k_{bl} = k_b + k_d$, of mEos3.2. The distribution of τ_{off} reports the recovery rate $k_{r1}$and k_{r2} of mEos3.2. (**d**) The distribution of τ_{on} (dots). The solid line is a fit with Supplementary Equation 6) with T_{int} = 4 ms, T_{tl} = 60 ms, and $k_{bl} = 250 \pm 12$ s^{-1}. (**e**) k_{bl} determined at different time-lapse T_{tl} = 20, 60, 100, and 200 ms, showing no dependence on T_{tl}. (**f**) The distribution of τ_{off} (dots). The solid line is a fit with a double exponential decay corrected with T_{int} = 4 ms, T_{tl} = 60 ms, and k_{r1}(blue curve) and k_{r2}(red curve) are 228 ± 12 and 30 ± 3 s^{-1} respectively. (**g**) k_{r1} and k_{r2} determined at different time-lapse T_{tl} = 20, 60, 100, and 200 ms, showing no clear dependence on T_{tl}. All error bars are standard deviations.

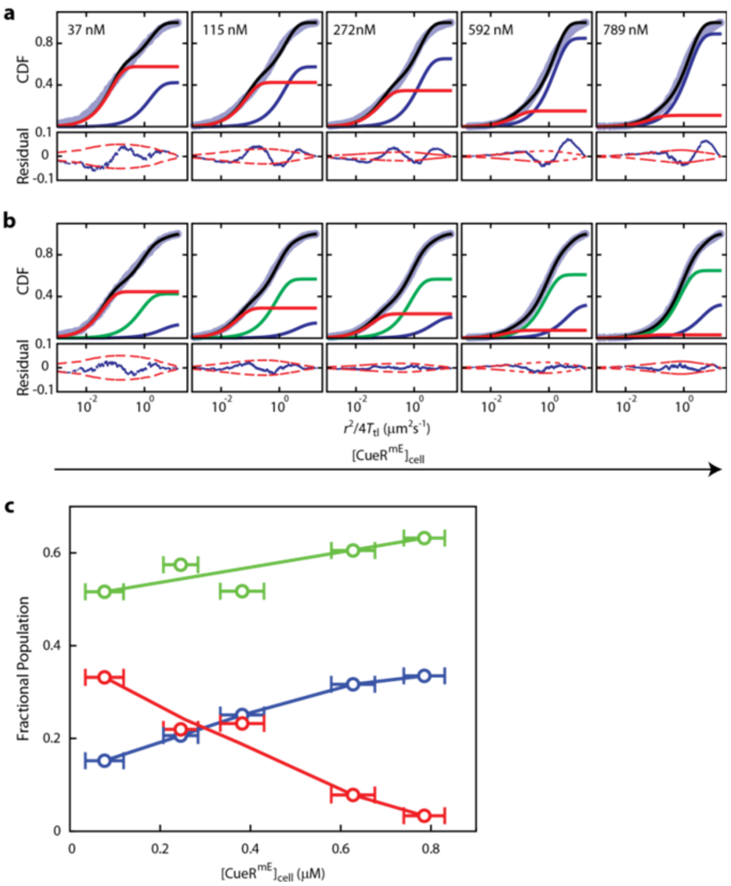

Supplementary Figure 22. Exemplary protein-concentration-dependent fitting results of CDFs of displacement *r* (plotted against $r^2/4T_{tl}$) for CueR^{mE}. CDFs fitted with (**a**) two and (**b**) three diffusion-state models. The blue circles represent the experimental CDF data, the black curve is the overall fit of two or three exponential components, which are individually plotted as blue and red in (**a**) and blue, green, and red in (**b**). Each CDF at a cellular CueR^{mE} concentration was obtained from tracking > 1000 molecules in more than 35 cells. The red dashed lines in the residual panels represent the 95% lower and upper confidence bounds, which are estimated from the Greenwood's formula [43]. (**c**) Corresponding fractional populations of the three diffusion states in (**b**) at different cellular protein concentrations where A_1, A_2, and A_3 are plotted as blue, green, and red open circles. Error bars of fractional populations are smaller than the symbol size. Error bars of protein concentrations are s.d. Solid lines are eye guides.

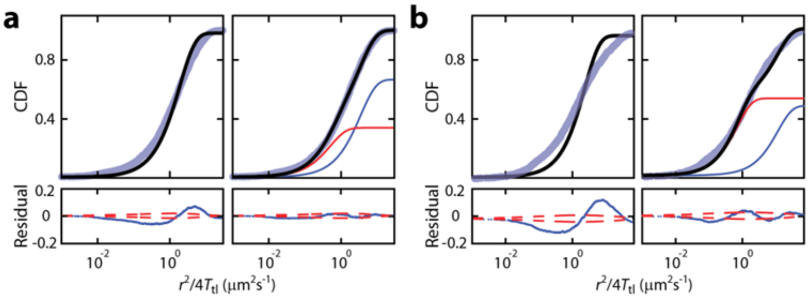

Supplementary Figure 23. CDF fitting results of $r^2/4T_{tl}$ for free mEos3.2 in *E. coli* cells with one (left) and two (right) diffusion-state models at two different T_{tl}. (**a**) T_{tl} = 60 ms. (**b**) T_{tl} = 15 ms. The blue circles represent the experimental CDF data, the black curve is the overall fit of one or two exponential components which are individually plotted as blue and red.

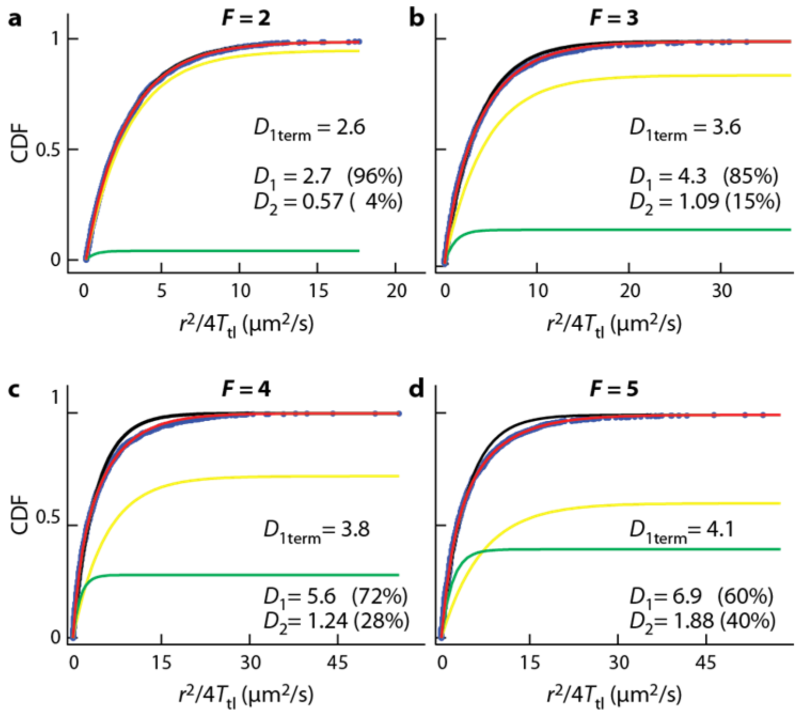

Supplementary Figure 24. The effect of aspect ratio *F* (= cell-length/cell-width) on the CDF analysis of *r*. Cell width was kept at 1.15 µm, and D_{input} = 11 µm^2/s. The blue dots are simulated data, and the black line is a CDF fit with a single component ($D_{1\text{term}}$ = the extracted D). The red line is a CDF fit with two components (D_1 and D_2, with the percentages being their respective amplitudes), and the yellow line is the fitted major component (D_1), and green one is the fitted minor component (D_2). As the aspect ratio increases, the second, artificial minor component is increasingly needed to fit the CDF satisfactorily.

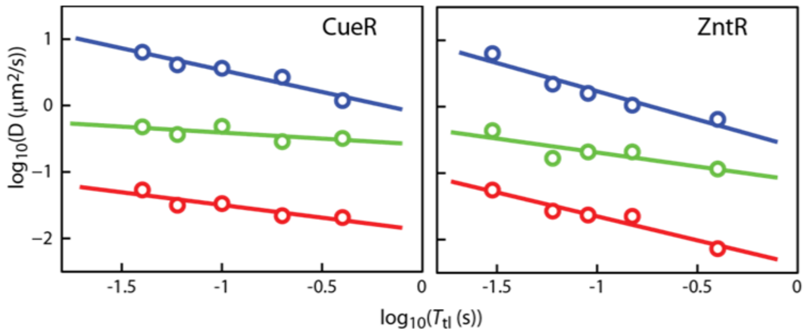

Supplementary Figure 25. Time-lapse dependent effective diffusion constants for CueR^{mE} and ZntR^{mE} in living *E. coli* cells. Diffusion constants for FD (D_1), NB (D_2), and SB (D_3) at different time-lapse are plotted as blue, green, and red circles respectively. Solid lines are fitted results of FD, NB, SB states with Supplementary Equation 12) and plotted in log scale.

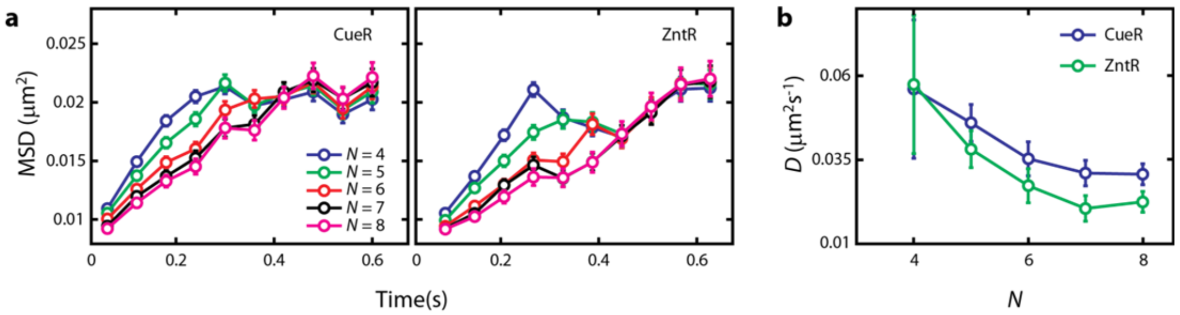

Supplementary Figure 26. Short-time movement analysis of CueR and ZntR in living *E. coli* cells. (**a**) MSD versus time lapse plot at using different residence time in frame number N, N = 4 to 8, for CueR (left) and ZntR (right). The data from all CueR or ZntR variants are used here for large statistics, except those promoter knockout controls. Error bars are s.e.m. (**b**) Diffusion rate constants extracted from first five points of each curve in (**a**) at different N for CueR and ZntR. Error bars are s.d.

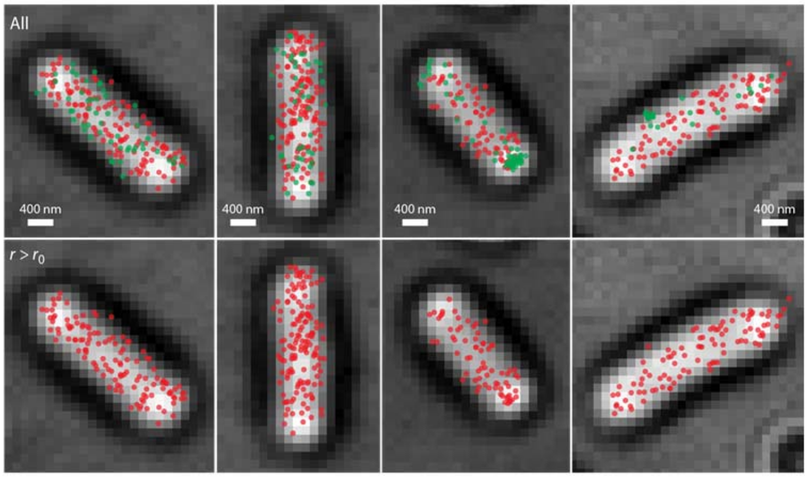

Supplementary Figure 27. Spatial distribution of tracked $\mathbf{CueR^{mE}_{apo}}$ molecules overlaid on top of corresponding cell transmission images. Top row: The spatial distribution of the first localizations of all trajectories of tracked molecules. Localizations during binding to DNA (i.e., residence sites) are marked in green. Bottom row: The spatial distribution of the first localizations from $r > r_0$ thresholding.

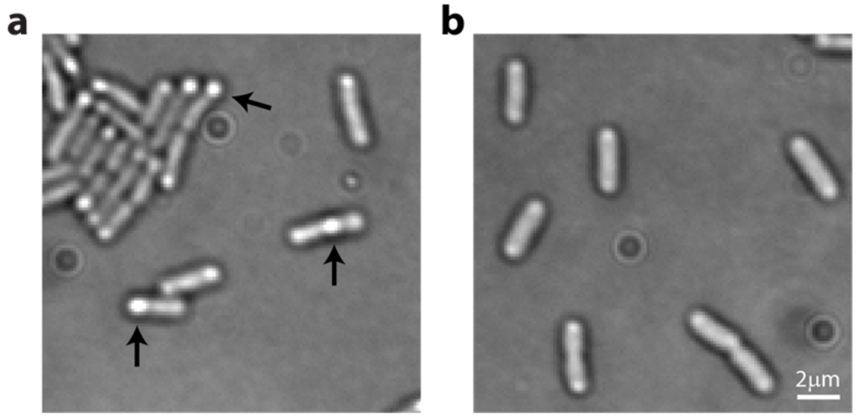

Supplementary Figure 28. Transmission images of cells expressing CueR tagged with a cleaved mEos3.2 fragment or complete mEos3.2. (**a**) Transmission image of cells expressing CueR tagged with a cleaved mEos3.2 fragment, where the expression was induced with IPTG for 2.5 hours in LB at 20°C. These cells show bright localized spots from inclusion bodies. (**b**) Transmission image of cells expressing $CueR^{mE}_{apo}$, after induction with L-arabinose in M9 at 37°C for 30 min, shows homogeneous morphology, and indicates no aggregation or inclusion bodies formed in the cells.

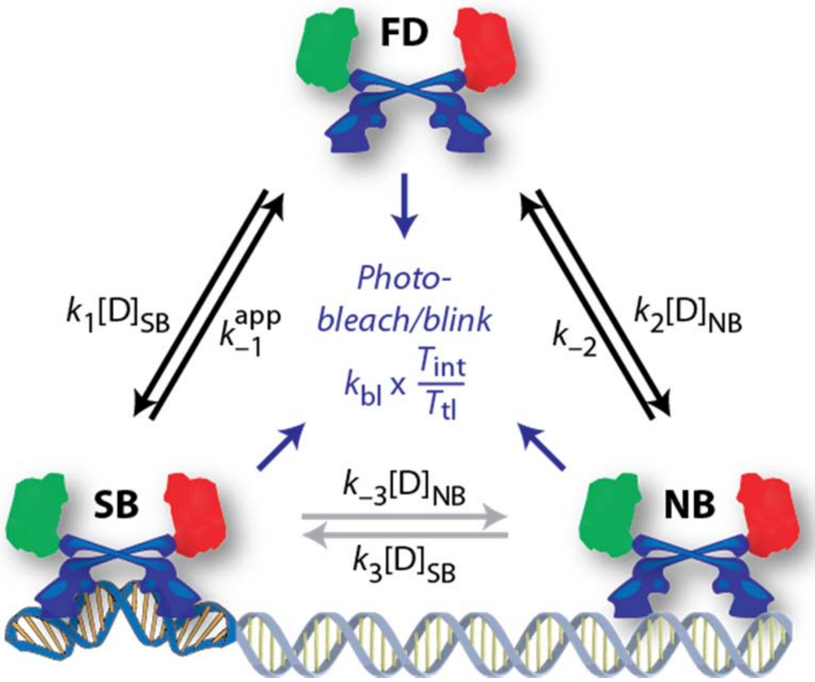

Supplementary Figure 29. Kinetic model for regulator-DNA interactions in a cell. The regulator can be freely diffusing (FD) in the cytoplasm, nonspecifically bound (NB) to DNA, or specifically bound (SB) to chromosomal recognition sites. A FD regulator can convert to a NB one reversibly via binding to nonspecific binding sites on chromosome (i.e., $k_2[D]_{NB}$) and spontaneous unbinding (i.e., k_{-2}). A FD regulator can also convert to a SB one reversibly via binding to specific recognition sites on chromosome (i.e., $k_1[D]_{SB}$) and unbinding from the sites with an apparent rate constant k_{-1}^{app}, $k_{-1}^{app} = k_{-1} + k_f[P]_{FD}$, which includes both spontaneous unbinding (i.e., k_{-1}) and facilitated unbinding (i.e., $k_f[P]_{FD}$). At any state, the mEos3.2 tag can undergo photobleaching/blinking with the rate constant corrected by the laser exposure and lapse time (i.e., $k_{bl}T_{int}/T_{tl}$). The direct interconversion between NB and SB states for a regulator is assumed to be sufficiently slow to be negligible in the overall kinetics, but they are indicated on the scheme (grey arrows) with their apparent rate constants $k_{-3}[D]_{NB}$ and $k_3[D]_{SB}$. This is the same figure as Fig. 2d in the main text.

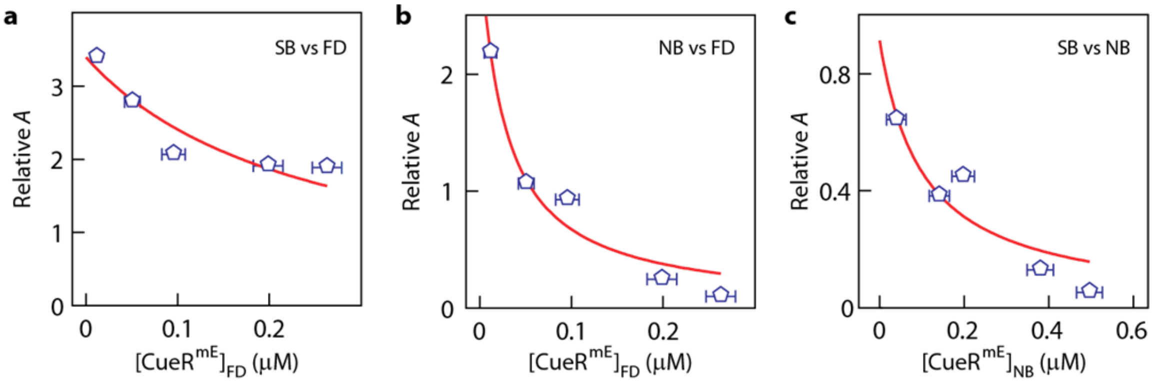

Supplementary Figure 30. Relative population analysis of different states of $CueR^{mE}$ in cells. Fractional population data of (a) $[PD]_{SB}/[P]_{FD}$ vs. $[P]_{FD}$, (b) $[PD]_{NB}/[P]_{FD}$ vs. $[P]_{FD}$, and (c) $[PD]_{SB}/[PD]_{NB}$ vs. $[PD]_{NB}$ generated from Supplementary Figure 22c for $CueR^{mE}$. Experimental data and the fitting results are plotted as blue open circles and red curves, respectively. Error bars are s.d.

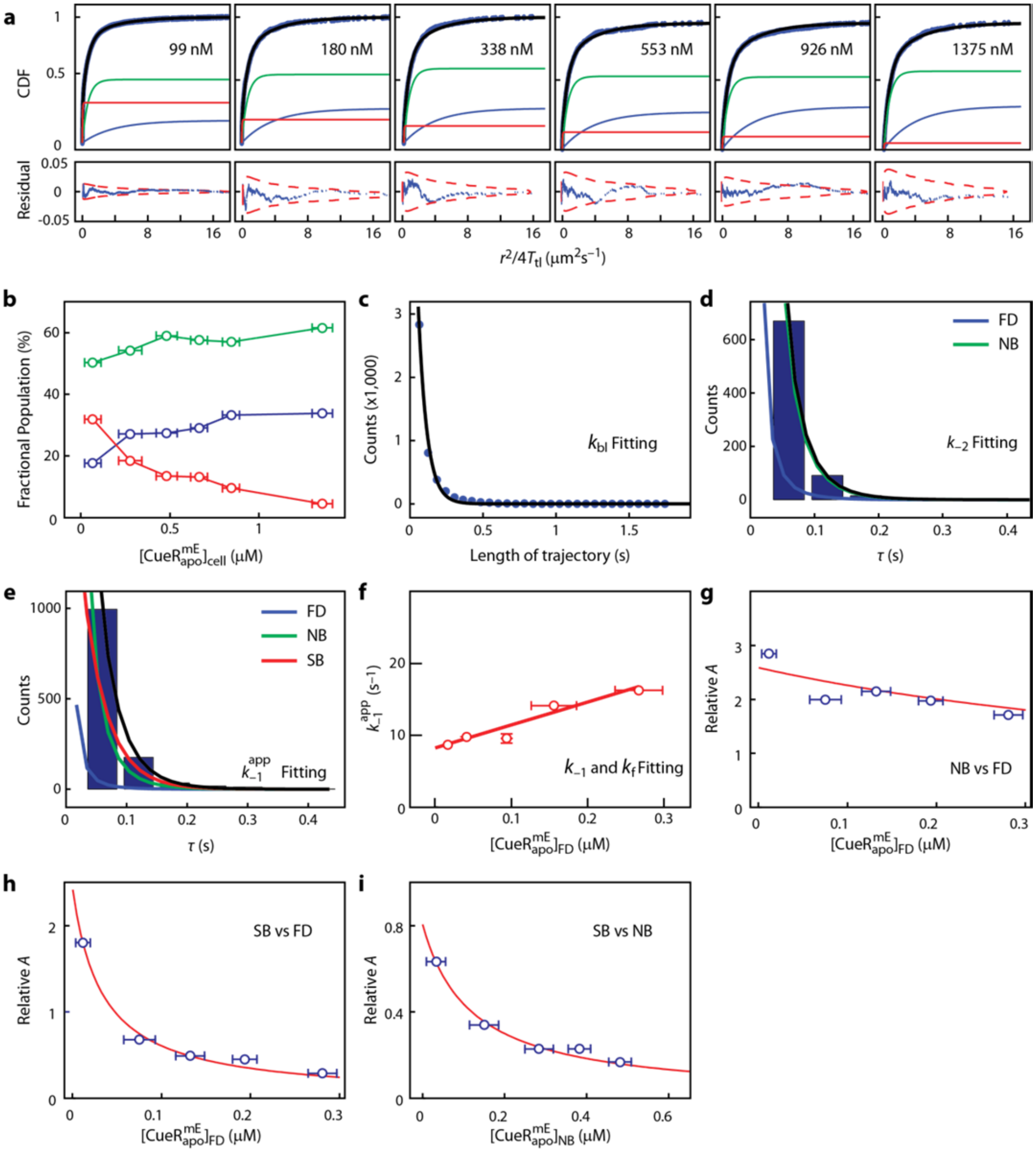

Supplementary Figure 31. Exemplary analysis and extraction of kinetic and thermodynamic parameters for CueR$^{mE}_{apo}$ in interactions with DNA in *E. coli* cells. (**a**) D_{FD}, D_{NB}, and D_{SB} and their associated fractional populations (A's) are extracted by fitting CDFs of r across a range of protein concentrations. In each panel, the black curve is the overall fit including FD, NB, and SB states, which are individually plotted as blue, green and red curves, respectively. (**b**) Fitting CDFs with shared diffusion constants also reports the fractional populations of FD (A_{FD}, blue circle), NB (A_{NB}, green circle), and SB (A_{SB}, red circle) at each protein concentration. At the highest protein concentration, A_{SB} is only ~3%. (**c**) Extract k_{bl} from the distribution of τ_{on} in the fluorescence intensity trajectories. (**d**) At the highest protein concentration condition in (**b**), A_{SB} is less than 5% and the residence time distribution at this concentration is dominated from FD and NB. With D_{FD}, D_{NB}, A_{FD}, and A_{NB} from (**a**) and (**b**), and k_{bl} from (**c**), fitting the residence time with Supplementary Equation 22 gives k_{-2}. (**e**) With known diffusion constants, fractional populations, k_{bl}, and k_{-2}, fitting the residence time distribution for A_{SB} > 5% conditions with Supplementary Equation 21 gives k^{app}_{-1}. (**f**) Linearly fit the k^{app}_{-1} vs. $[P]_{FD}$ plot to get k_{-1} and k_f. (**g**) Determine K_{D2} (= k_{-2}/k_2) and $[D_0]_{NB}$ from $[PD]_{NB}$ / $[P]_{FD}$ fit. With known k_{-2}, we get k_2. (**h**) Determine K_{D1} (= k_{-1}/k_1) and $[D_0]_{SB}$ from $[PD]_{SB}$ / $[P]_{FD}$ fit. With known k_{-1} and k_f, we get k_1. (**i**) Determine K_{D3} (= k_{-3}/k_3) from $[PD]_{SB}/[PD]_{NB}$ fit with known $[D_0]_{SB}$ *and* $[D_0]_{NB}$. All error bars are s.d.

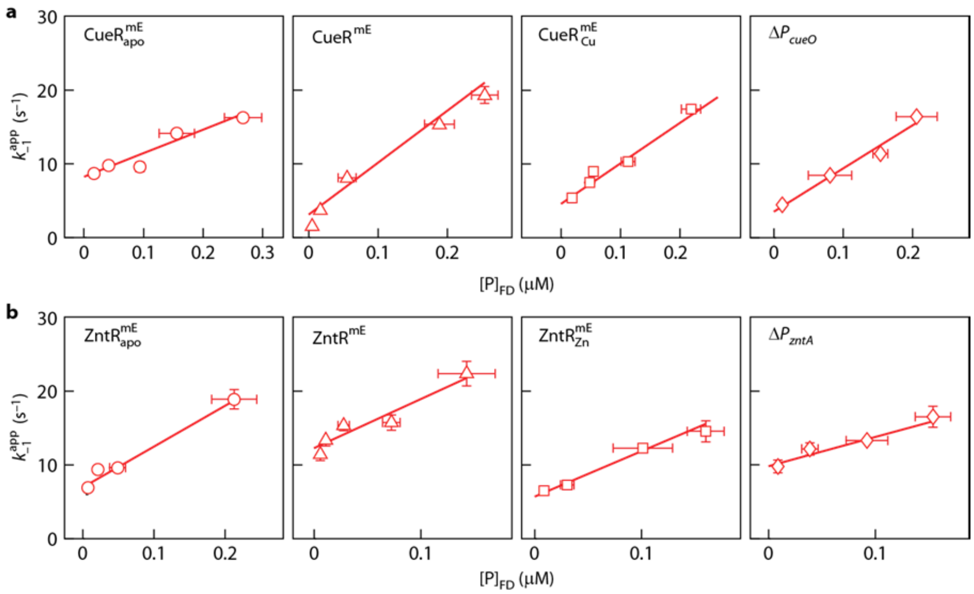

Supplementary Figure 32. Apparent unbinding rate constant from recognition sites k^{app}_{-1} vs. free protein concentration $[P]_{FD}$ for all CueR or ZntR variants with k_{-2} extracted from the highest cellular protein concentration condition in each variant. (a) k^{app}_{-1} vs. $[P]_{FD}$ for $CueR^{mE}_{apo}$, $CueR^{mE}$, $CueR^{mE}_{Cu}$, and ΔP_{cueO}. (**b**) k^{app}_{-1} vs. $[P]_{FD}$ for $ZntR^{mE}_{apo}$, $ZntR^{mE}$, $ZntR^{mE}_{Zn}$, and ΔP_{zntA}. All experiments were imaged in metal-depleted medium except for $CueR^{mE}_{Cu}$/$ZntR^{mE}_{Zn}$ for which the cells were imaged in the presence of 100 μM Cu^{2+}/Zn^{2+} in the medium. All error bars are s.d.

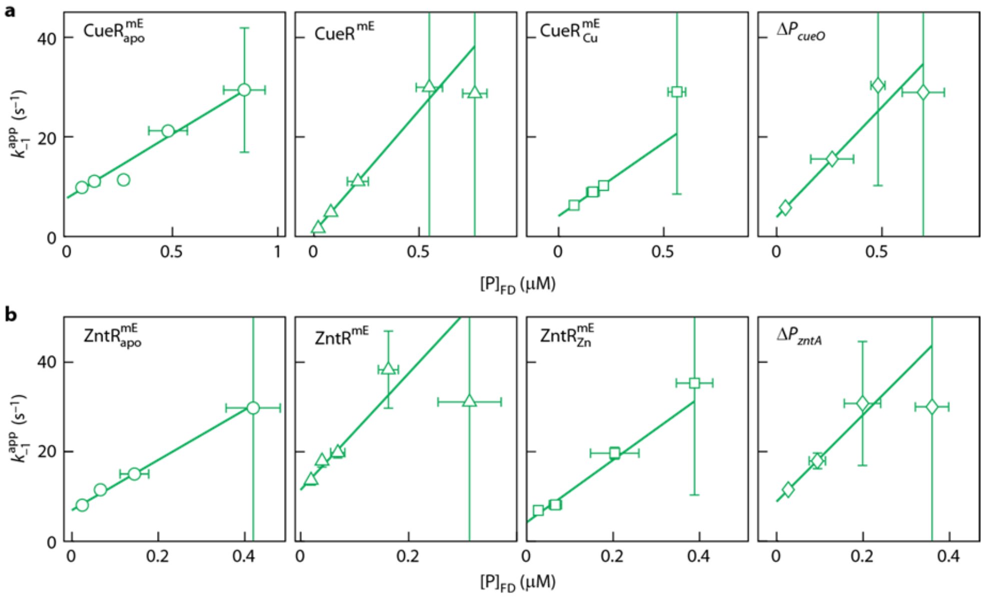

Supplementary Figure 33. k^{app}_{-1} vs. $[P]_{FD}$ plots of all CueR or ZntR variants with $k_{-2} = 0$ for all CueR variants (**a**) and all ZntR variants (**b**).

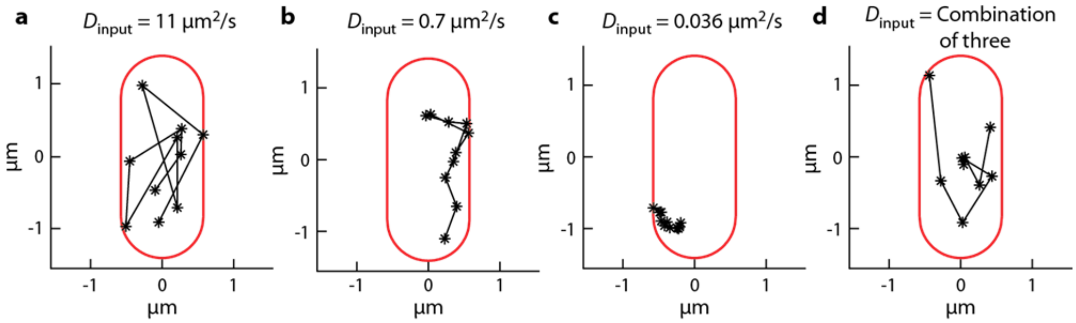

Supplementary Figure 34. Examples of simulated position trajectories. (a)-(c) Trajectories from single diffusive state simulations. The input diffusion constants here correspond to the intrinsic diffusion constants for the FD (a), NB (**b**), and SB state (**c**), respectively. The input values of diffusion constants in the simulations are 11, 0.7, and 0.036 μm^2/s, respectively. (**d**) A simulated trajectory including three diffusion states. Same intrinsic *D*'s as in (a)-(c) are used as input, and the values of interconversion rates are taken from our $\mathrm{CueR}^{\mathrm{mE}}_{\mathrm{apo}}$ data at the lowest concentration (i.e., $[\mathrm{CueR}^{\mathrm{mE}}_{\mathrm{apo}}]_{\mathrm{cell}}$ = 99 nM; γ_{12} (i.e., $k_2[\mathrm{D}]_{\mathrm{NB}}$) = 8.4 s^{-1}, γ_{21} (i.e., k_{-2}) = 2.6 s^{-1}, γ_{13} (i.e., $k_1[\mathrm{D}]_{\mathrm{SB}}$) = 15.3 s^{-1}, γ_{31} (i.e., k_{-1}^{app}) = 8.8 s^{-1} and γ_{23} (i.e., $k_3[\mathrm{D}]_{\mathrm{SB}}$) = γ_{32} (i.e., $k_{-3}[\mathrm{D}]_{\mathrm{NB}}$) = 0 s^{-1} and k_{bl} = 252 s^{-1}. Note the 1, 2, and 3 for γ's are for NB, SB, and FD state, respectively. Supplementary Table 6). Cell geometry: width = 1.15 μm, length = 2.82 μm.

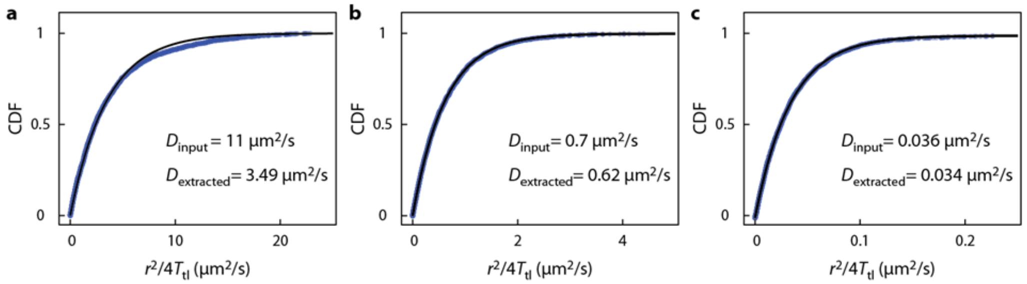

Supplementary Figure 35. CDF analysis of the displacement *r* of the simulated trajectories of single diffusion state. The cell width and length in simulation are 1.15 μm, and 2.82 μm, respectively. The input values of diffusion constant in simulated trajectory are 11 (**a**), 0.7 (**b**), and 0.036 μm^2/s (**c**). The blue points are simulation results (i.e., data) and the solid black lines are fits with a single diffusion component $\boldsymbol{CDF(r) = 1 - \exp(-\frac{r^2}{4DT_{\mathrm{tl}}})}$.

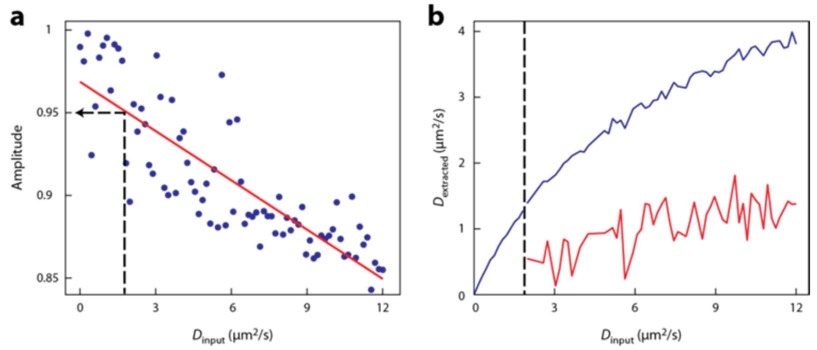

Supplementary Figure 36. CDF fitting results of 1-state diffusion simulation results. The cell width and length in simulations are 1.15 and 2.82 μm, respectively. (**a**) Dependence of the amplitude of the major, faster fitted diffusion component from the 2-component fitting of CDF of *r*. The red line is a linear fit to the results (dots). The black dashed lines mark the amplitude = 0.95 when D_{input} = 1.88 μm^2/s. (**b**) The extracted *D* ($D_{\mathrm{extracted}}$) from CDF fitting versus the D_{input} of the simulation. The vertical black dash line marks D_{input} = 1.88 μm^2/s. When $D_{\mathrm{input}} \leq$ 1.88 μm^2/s, the results from the single-component fitting of the CDF were used. When D_{input} > 1.88 μm^2/s, the results from the 2-compoment fitting of the CDF were used: a major/faster component D_{major} (blue) and a minor/slower component D_{minor} (red); D_{major} reflects the apparent diffusion constant in a confined cell geometry, whereas D_{minor} is an artifact from the CDF fitting.

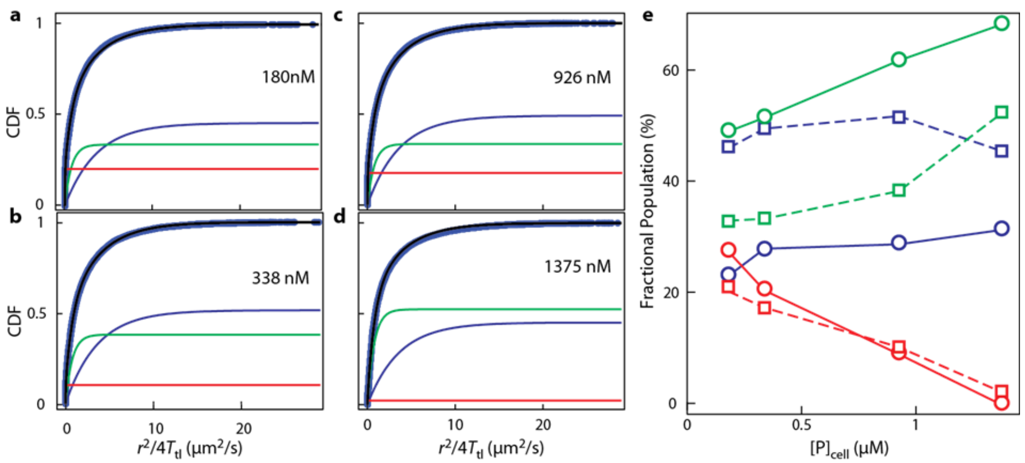

Supplementary Figure 37. Analysis of the CDF of *r* from the simulations of three (and two) interconverting diffusion states. (a-d) CDF fitting. The blue circles are results from the simulations. The black lines are the CDF fit using three diffusion components with apparent diffusion constants (and amplitudes) D_1 (A_1), D_2 (A_2), and D_3 (A_3); the three fitted components are also individually plotted as blue, green, and red lines. The input values of interconversion rate constants were taken from the experimental results on $\mathrm{CueR}^{\mathrm{mE}}_{\mathrm{apo}}$ at cellular protein concentrations of 180 nM (**a**), 338 nM (**b**), and 926 nm (**c**) and summarized in Supplementary Table 6. The cell width and length in simulation are 1.15 μm and 2.82 μm, respectively. Input diffusion constants were taken as the expected intrinsic diffusion constants from the experimental apparent diffusion constants on the basis of the correlation in Supplementary Figure 36b, and are given in Supplementary Table 6. (**d**) is from a 2-state simulation, mimicking the case at the highest cellular protein concentration (1375 nM; Supplementary Table 6) where the SB state is negligible. This simulation is performed to extract the nonspecific unbinding rate constant, $k_{-2,}$ as we did on the experimental data. The CDF fit here is still 3-component fit to demonstrate the SB state is indeed negligible, i.e., $A_3 < 5\%$. (**e**) Amplitude of each state is plotted against the corresponding cellular protein concentrations. The solid lines with circles are simulation inputs, taken from results on $\mathrm{CueR}^{\mathrm{mE}}_{\mathrm{apo}}$, and the dash lines with squares are those from the CDF analysis of the simulations. Blue is for FD (A_1), green is for NB (A_2), and red is for SB (A_3). The A_3 decreases as cellular protein concentration increases.

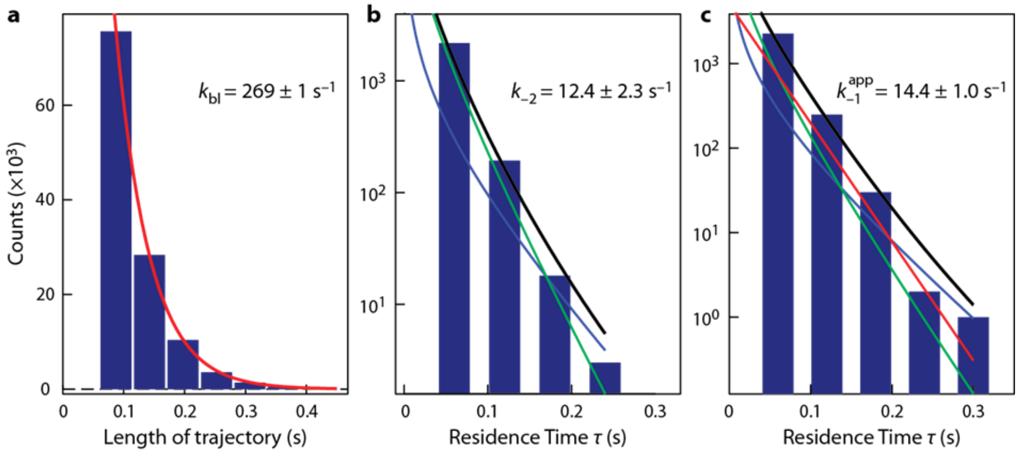

Supplementary Figure 38. Extraction of rate constants from analyzing the 3-state and 2-state diffusion simulations. (**a**) The distribution of the trajectory length of 3-diffusion-state simulation gives the photobleaching/blinking rate constant k_{bl} of mEos3.2. The red line is a fit with Supplementary Equation 6) (i.e., $\boldsymbol{C}_{\mathbf{bl}}\mathbf{exp}(-\boldsymbol{k}_{\mathbf{bl}}\frac{\boldsymbol{T}_{\mathbf{int}}}{\boldsymbol{T}_{\mathbf{tl}}}\boldsymbol{t})$), giving $k_{\mathrm{bl}} = 269 \pm 1\ \mathrm{s}^{-1}$ with $T_{\mathrm{int}} = 4$ ms and $T_{\mathrm{tl}} = 60$ ms. (**b**) Residence time distribution from 2-diffusion-state simulations, including the FD and NB states. The black line is a fit with Supplementary Equation 22, including the FD and NB state contributions to the residence times, which are also plotted individually as blue and green lines, respectively. The fit gives γ_{21}(i.e., k_{-2}) = $12.4 \pm 2.3\ \mathrm{s}^{-1}$. (**c**) Exemplary residence time distribution from a 3-diffusion-state simulation as in Supplementary Figure 37c. Using the extracted apparent diffusion constants and fractional populations Supplementary Figure 37c, k_{bl} from (**a**), and γ_{21}(i.e., k_{-2}) from (**b**), fitting the residence time distribution with Supplementary Equation 21 (black line) gives γ_{31}(i.e., $\boldsymbol{k}_{-\mathbf{1}}^{\mathbf{app}}$). The residence time distribution fit includes contributions from FD, NB, and SB states, which are individually plotted as blue, green and red curves, respectively.

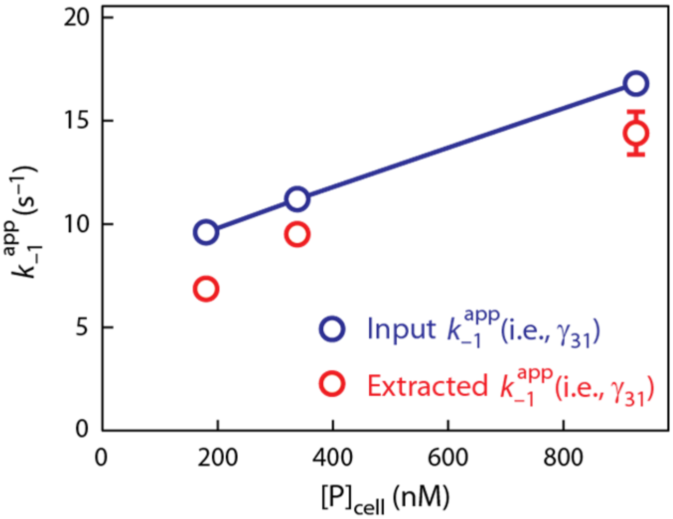

Supplementary Figure 39. The extracted $\boldsymbol{k_{-1}^{app}}$ (red symbols) from analyzing the 3-state diffusion simulation versus the input γ_{31} in Supplementary Table 6 (blue dots) in the simulations, both plotted against the corresponding cellular protein concentrations. The trend of $\boldsymbol{k_{-1}^{app}}$ increasing with increasing cellular protein concentration is clear. Error bars are s.d.

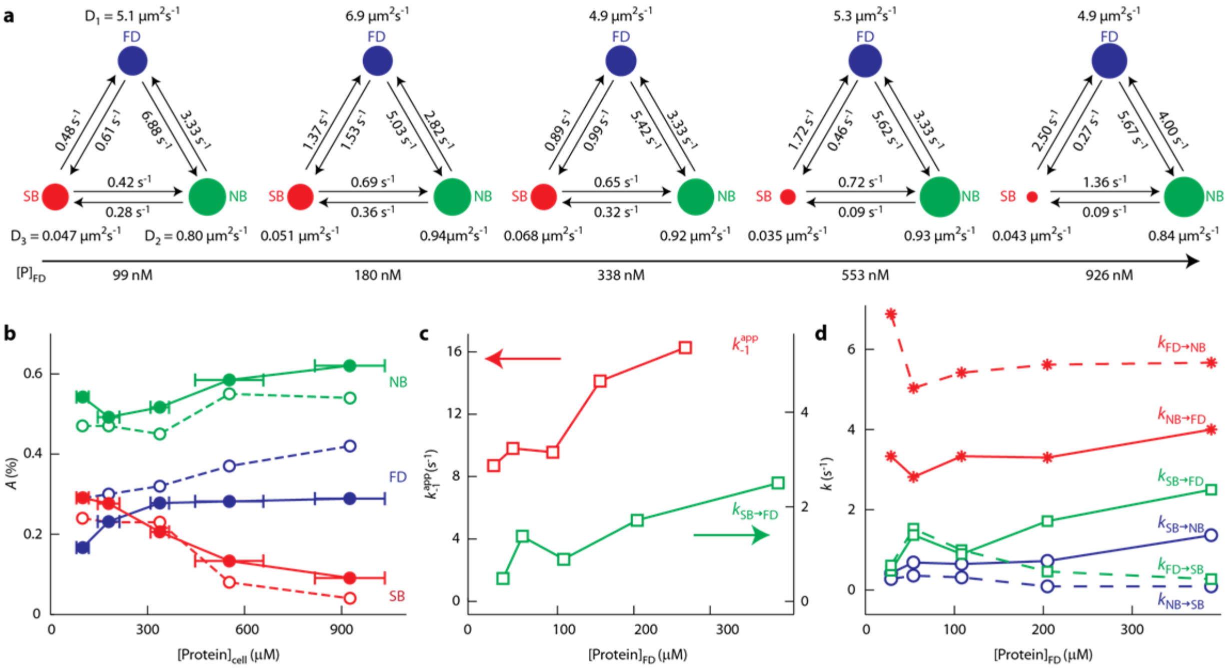

Supplementary Figure 40. Hidden Markov analysis of CueR$^{\mathbf{mE}}_{\mathbf{apo}}$ tracking via vbSPT. (**a**) Diffusion constants (D_1 for FD, D_2 for NB, and D_3 for SB) and interconversion rates of CueR$^{mE}_{apo}$ across a range of protein concentrations extracted from vbSPT with a 3-state model. FD, NB, and SB states were represented as blue, green, and red circles respectively. (**b**) Comparison of protein-concentration dependent fractional population A of CueR$^{mE}_{apo}$ obtained from our CDF analysis (solid line solid circles; error bars are s.d.; Section 14.4) and from vbSPT (dash line open circles). (**c**) Protein-concentration dependence of $\boldsymbol{k_{-1}^{app}}$ from our residence time analysis (red square, Section 14.4) and vbSPT (green square). Both analyses show that $\boldsymbol{k_{-1}^{app}}$ increases with free-diffusing protein concentrations (= [P]$_{cell}A_{FD}$). (**d**) Protein-concentration dependent interconversion kinetics of CueR$^{mE}_{apo}$ extracted from vbSPT. The curve of $k_{SB\rightarrow FD}$ is the same as the one in (**c**).

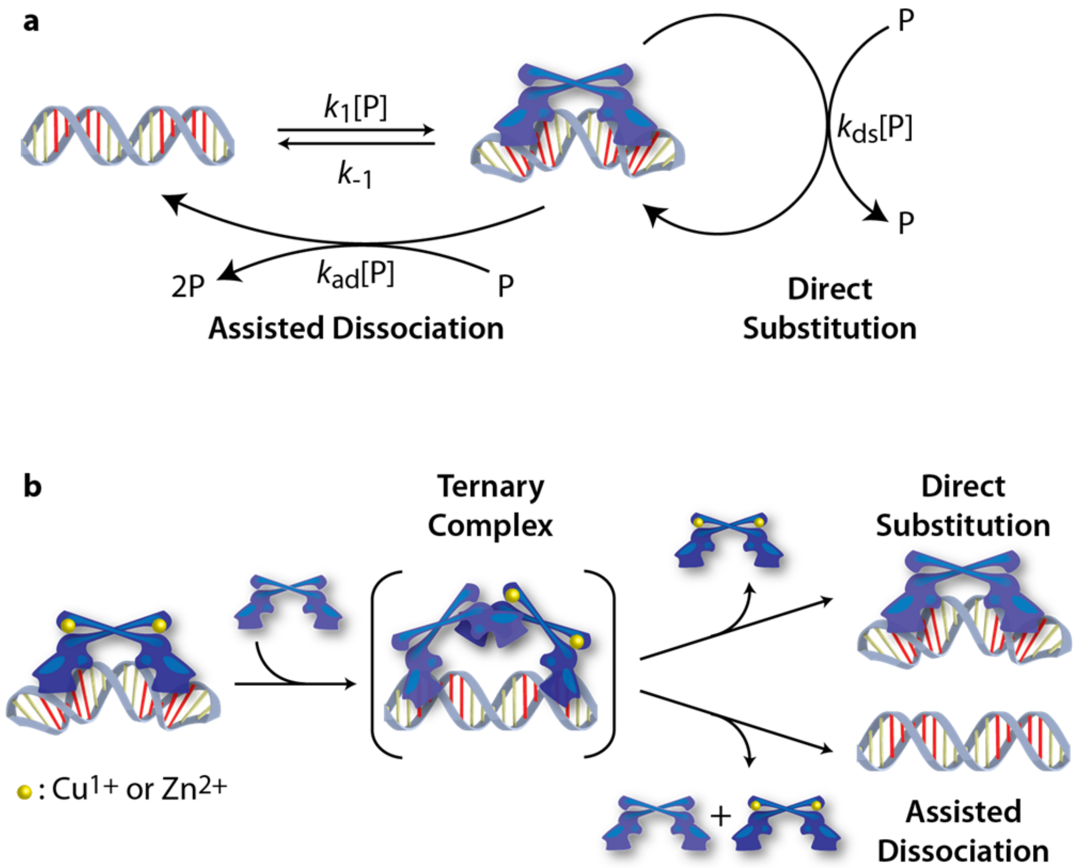

Supplementary Figure 41. **(a)** Part of the kinetic mechanism of CueR in interacting with specific DNA sequences containing recognition sites from our previous *in vitro* work [50] (red color bars on DNA marks the dyad symmetric sequence recognized by CueR). *In vitro*, the specifically bound CueR at the recognition site may also undergo a conformational change to a different binding mode, which is not plotted in this mechanism here. **(b)** Proposed molecular mechanism [66] for direct substitution and assisted dissociation pathways for protein unbinding from DNA recognition sites, which involves the formation of a ternary $CueR_2$-DNA complex. Note CueR and ZntR are homodimers.

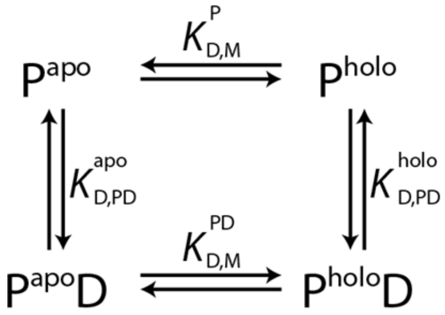

Supplementary Figure 42. General coupling scheme for metal binding to the DNA-bound and free metal-responsive regulator.

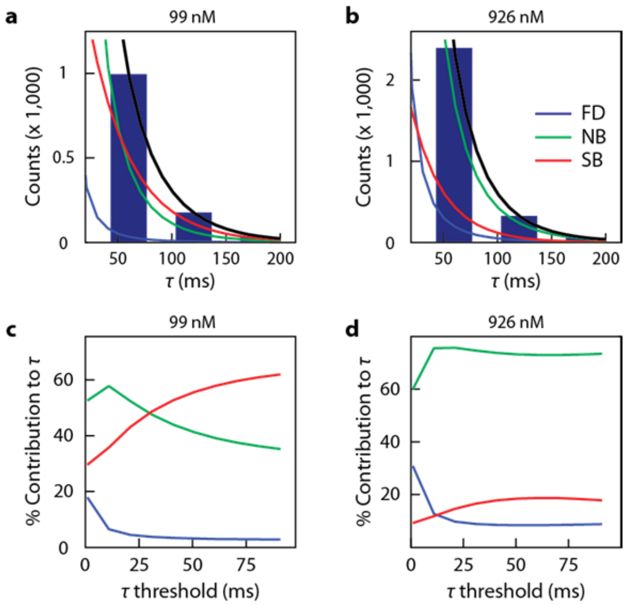

Supplementary Figure 43. Relative contributions of FD, NB, and SB states to the microscopic residence time τ distribution of CueR$^{\mathrm{mE}}_{\mathrm{apo}}$. (**a**) Distribution of τ at a low CueR$^{\mathrm{mE}}_{\mathrm{apo}}$ cellular concentration of 99 nM and fit with Supplementary Equation 21 (black line), as well as the deconvoluted contributions from the FD (blue), NB (green), and SB (red) states. (**b**) Same as (**a**)**,** but for data at a high CueR$^{\mathrm{mE}}_{\mathrm{apo}}$ cellular concentration of 926 nM. (**c**) Percent contributions of FD, NB, and SB states to the distribution of τ as a function of τ threshold for (**a**). (**d**) Same as (**c**), but for (**b**). The percent population of each state is estimated by dividing the area under each state by the area sum of three states after specified τ threshold. For example, for FD state contribution, it is $\int_{\mathbf{threshold}}^{\infty} A_{\mathbf{FD}}\varphi_{\mathbf{FD}}(\tau)\mathbf{d}\tau / \int_{\mathbf{threshold}}^{\infty} \varphi(\tau)_{\mathbf{all}}\mathbf{d}\tau$, where the functions are defined in Supplementary Equation 21).

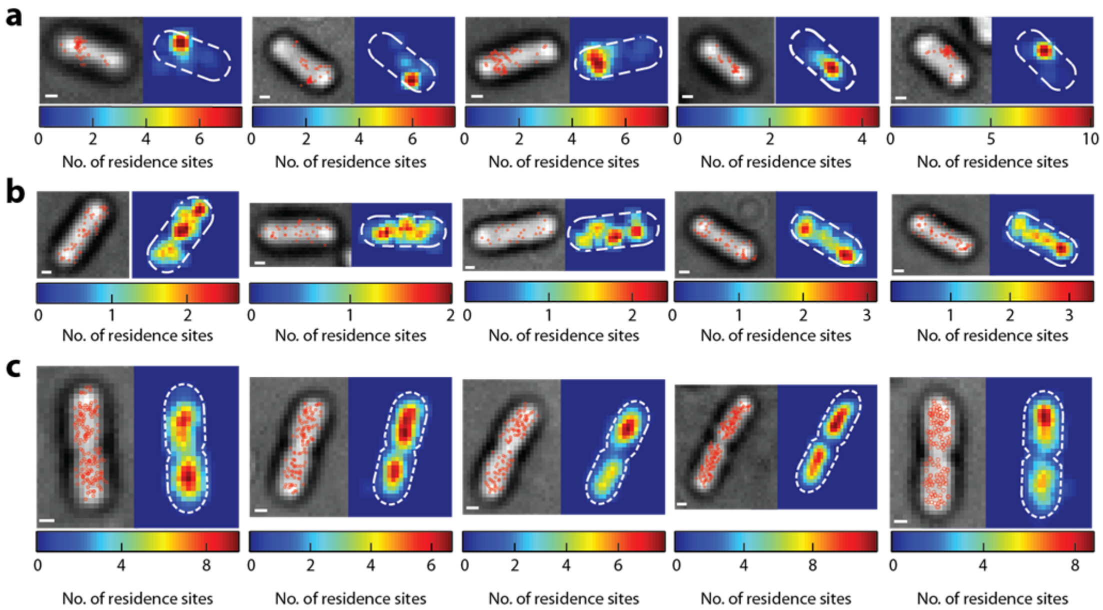

Supplementary Figure 44. Spatial distribution of residence sites of cells expressing CueR$^{\mathrm{mE}}_{\mathrm{apo}}$. (**a**) Exemplary nondividing cells with highly condensed chromosome organization. Each panel shows the locations of residence sites (red open circles) overlaid on the cell bright field transmission image (left) and the corresponding 2D histogram of the residence sites (right) where the white dash line marks the cell boundary. All scale bars = 400 nm. (**b**) Exemplary nondividing cells with less condensed chromosome that spreads over the cell. (**c**) Exemplary dividing cells, for which two copies of chromosomes are clearly observed, one in each half cell envelop and the division septum is clear in the transmission image.

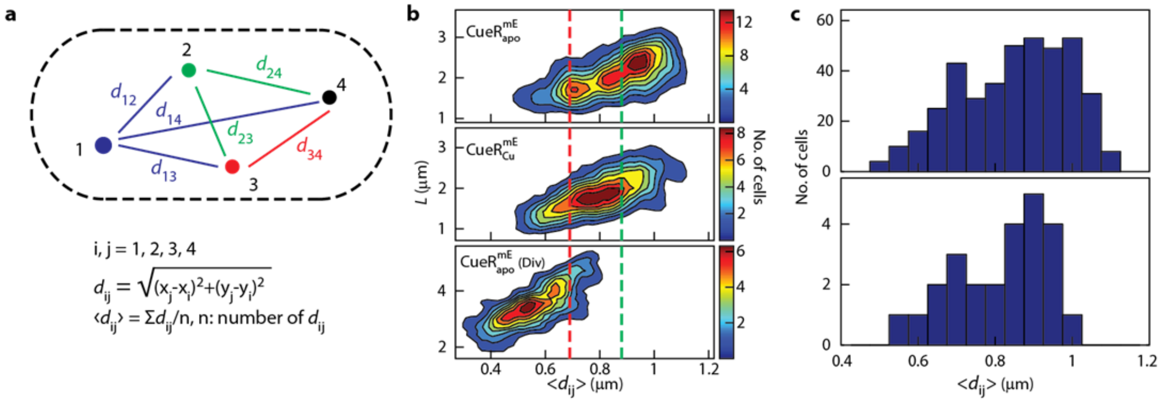

Supplementary Figure 45. (a) Schematic for the computation of $\langle d_{ij}\rangle$ from residence sites. Here residence sites are represented as solid circles. The Euclidean distance between residence sites i and j are first computed to generate d_{ij} as depicted in the scheme. The $\langle d_{ij}\rangle$ is the mean d_{ij}. (b) Two-dimensional histograms of cell length (L) and $\langle d_{ij}\rangle$ for nondividing cells expressing $\mathrm{CueR}^{\mathrm{mE}}_{\mathrm{apo}}$, $\mathrm{CueR}^{\mathrm{mE}}_{\mathrm{Cu}}$, or for dividing cells expressing $\mathrm{CueR}^{\mathrm{mE}}_{\mathrm{apo}}$. Vertical dash lines are at $\langle d_{ij}\rangle$ = 0.68 and 0.88 µm. **(c)** Distribution of $\langle d_{ij}\rangle$ for nondividing cells expressing $\mathrm{CueR}^{\mathrm{mE}}_{\mathrm{apo}}$: top, all cells with different cellular protein concentrations; bottom, those with $[\mathrm{P}]_{\mathrm{cell}}$ ~ 1150 nM.

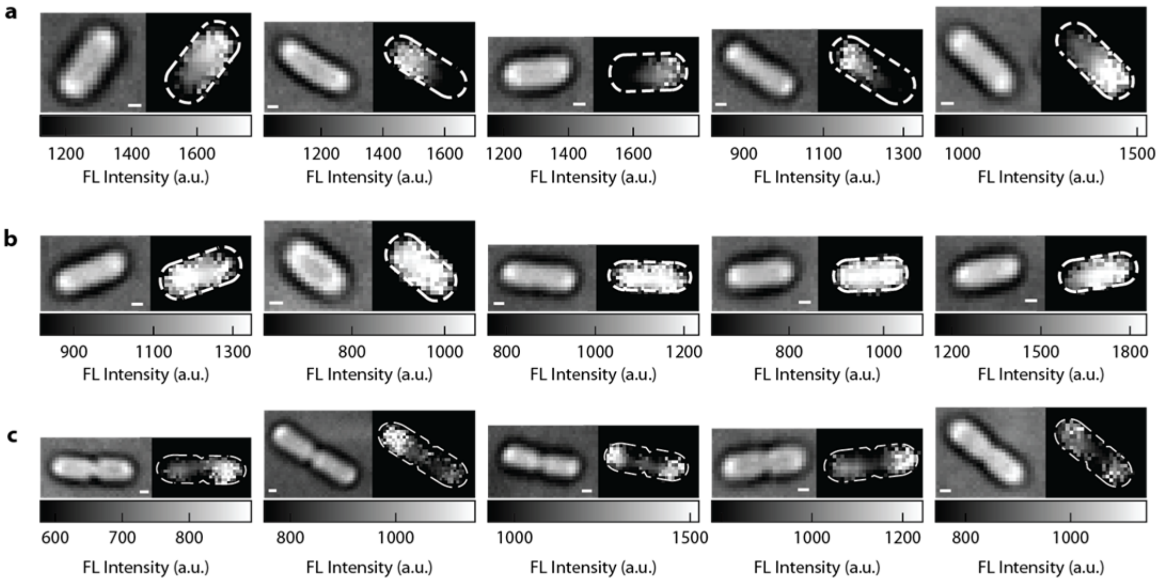

Supplementary Figure 46. Spatial organization of chromosome from Hoechst-dye-stained cells expressing $\mathrm{CueR}^{\mathrm{mE}}_{\mathrm{apo}}$. (**a**) Exemplary panels of non-dividing cells with highly condensed chromosome organization. Each panel shows the cell transmission image (left) and its fluorescence image (right) from the Hoechst dye stain. The white dash line in the fluorescence image outlines the cell boundary from the transmission image. All scale bars = 400 nm. (**b**) Same as (**a**), but for nondividing cells with less condensed chromosomes. (**c**) Same as (**a-b**), but for dividing cells. Here two chromosomes are clearly observed, one on each side of the division septum. Images are taken under 405 nm laser excitation, emission from Hoechst dye stain is then passed through an emission filter (460 ± 25 nm, Chroma AT460/50m**)** before being detected by an EMCCD camera; experimental details are in Section 4.3.

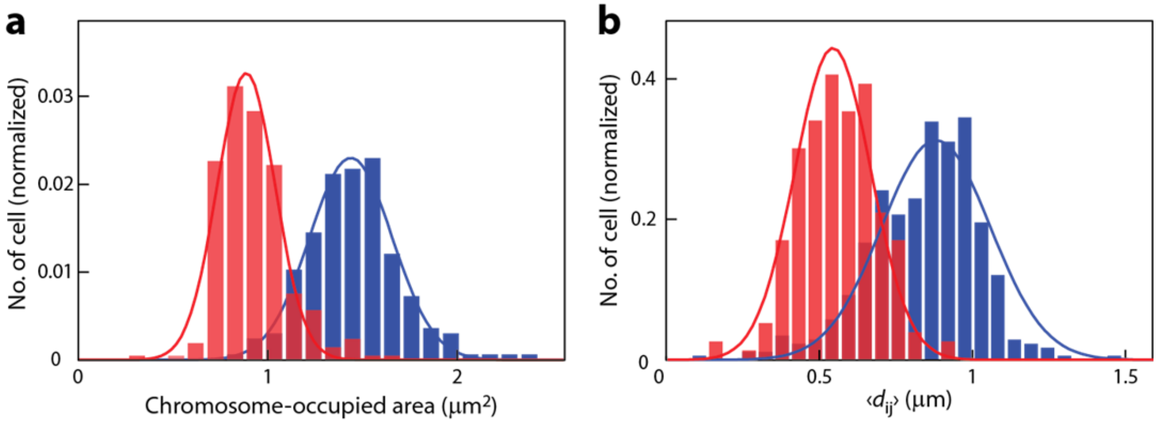

Supplementary Figure 47. Extent of chromosome condensation measured by Hoechst-dye-staining versus ⟨d_{ij}⟩ for nondividing and dividing cells that express $\mathrm{CueR}^{\mathrm{mE}}_{\mathrm{apo}}$. (**a**) Distributions of the chromosome-occupied area for nondividing (blue bar) and dividing (red bar) cells from imaging Hoechst-dye-staining. *y* axis represents the number of cells normalized to the total number. (**b**) Distribution of ⟨d_{ij}⟩ of nondividing (blue bar) and dividing (red bar) cells. The data for nondividing cells are the same as that in Supplementary Figure 45c top panel. *y* axis represents the number of cells normalized to the total number. Each distribution in (**a**) is fitted with a Gaussian distribution function (red and blue curves for dividing and nondividing cell, respectively). The Gaussian fitted curves from (**a**) are then overlaid on top of the ⟨d_{ij}⟩ populations in (**b**) after scaling. The scaling factor is estimated by fitting both distributions in (**b**) with Gaussian curves from (**a**) simultaneously.

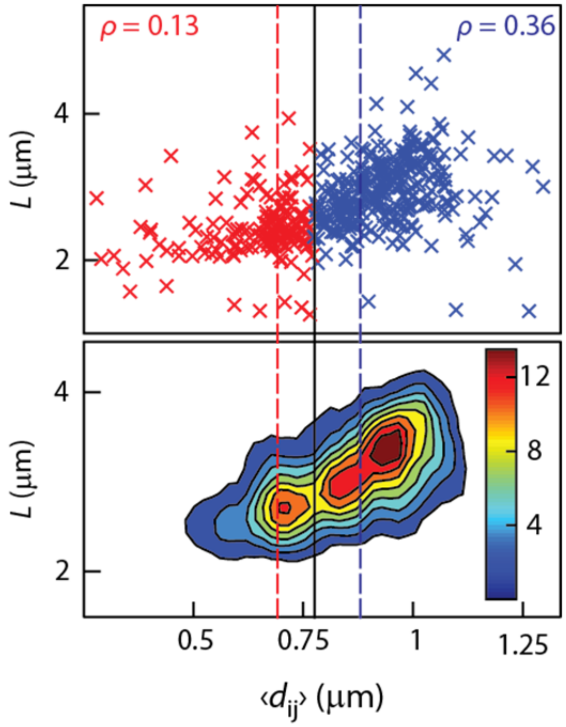

Supplementary Figure 48. Correlation between each cell's length (*L*) versus its average pairwise distance ⟨d_{ij}⟩ in scatter plot (upper) and in 2D histogram plot (lower) for nondividing cells that express $\mathrm{CueR}^{\mathrm{mE}}_{\mathrm{apo}}$. This 2D histogram (same as the top panel in Supplementary Figure 45b) resolves two distinct subpopulations, one with smaller ⟨d_{ij}⟩ and the other larger. The black vertical line at ⟨d_{ij}⟩ = 0.77 μm separate these two subpopulations. The two subpopulations are further colored in red and blue in the scatter plot, and for each population, a Pearson's correlation coefficient (ρ) is calculated between *L* and ⟨d_{ij}⟩.

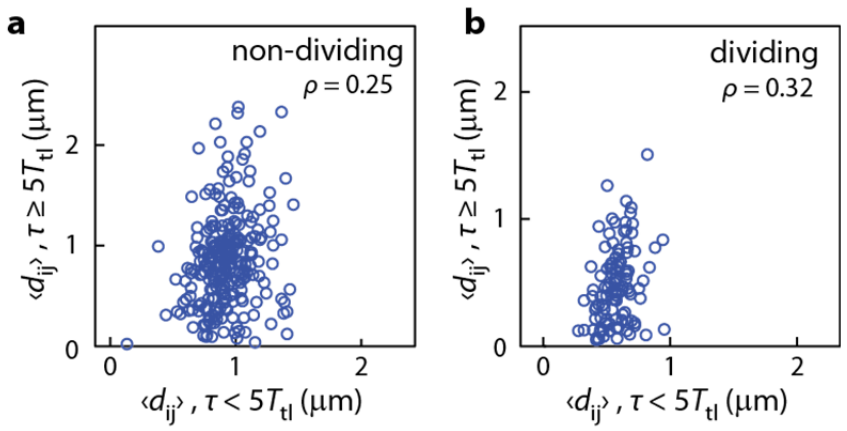

Supplementary Figure 49. Scatter correlation plots for $\langle d_{ij} \rangle$ from residence sites with residence time $\tau \geq 5T_{tl}$ and from those with $\tau < 5T_{tl}$. (a) For nondividing cells and **(b)** for dividing cells expressing $\mathrm{CueR}^{\mathrm{mE}}_{\mathrm{apo}}$. The cross-correlation coefficients here are 0.25 and 0.32, respectively.

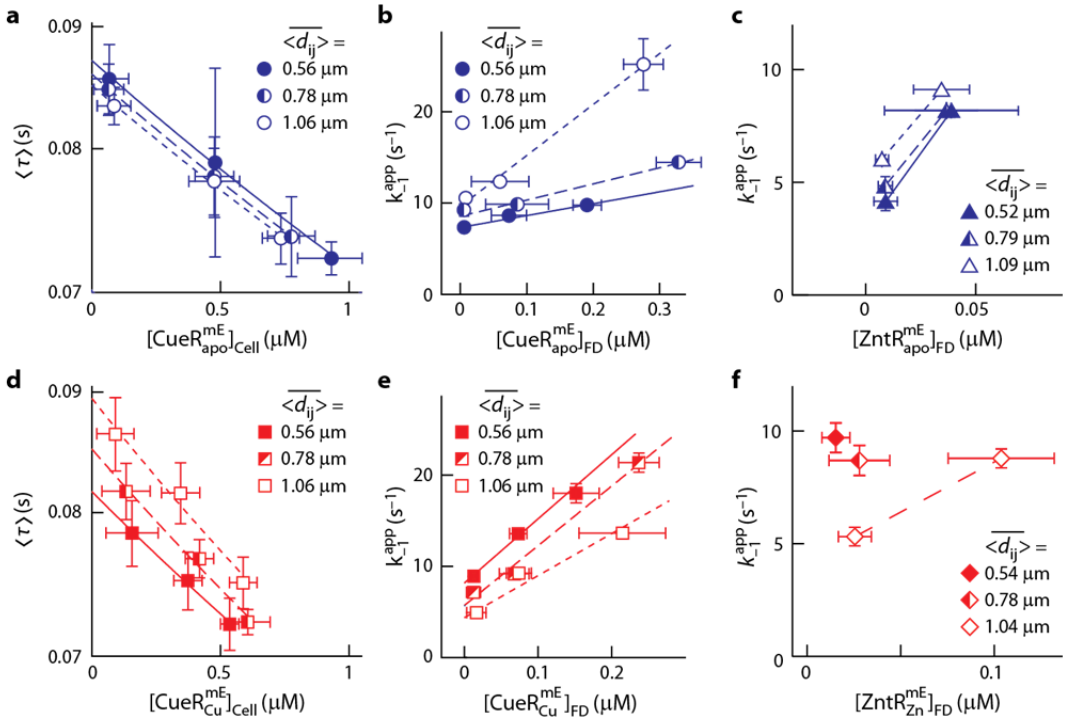

Supplementary Figure 50. Chromosome-organization-dependent k_{-1}^{app} of CueR or ZntR in nondividing cells. (**a**, **b**) Cellular protein-concentration-dependent average residence time $\langle \tau \rangle$ (**a**) and the corresponding k_{-1}^{app} (**b**) of $\mathrm{CueR}^{\mathrm{mE}}_{\mathrm{apo}}$ for cells with different chromosome organizations (i.e., different $\overline{\langle \boldsymbol{d}_{\mathrm{ij}} \rangle}$). (**c**) Same as (**b**) but for $\mathrm{ZntR}^{\mathrm{mE}}_{\mathrm{apo}}$. (**d**, **e**) Same as (**a**, **b**) but for $\mathrm{CueR}^{\mathrm{mE}}_{\mathbf{Cu}}$. (**f**) Same as (**c**) but for $\mathrm{ZntR}^{\mathrm{mE}}_{\mathbf{Zn}}$. Note that panel (**b**) and (**e**) are the same as Fig. 4a and b in the main text. Please note that ZntR variants have lower expression levels than CueR variants and thus less number of SMT results in total; as a result, after sub-dividing the cells into groups of different chromosome condensations, there are less data points in the dependence of k_{-1}^{app} vs. cell protein concentration for ZntR variants (Supplementary Figure 50c and f). All error bars are s.d.

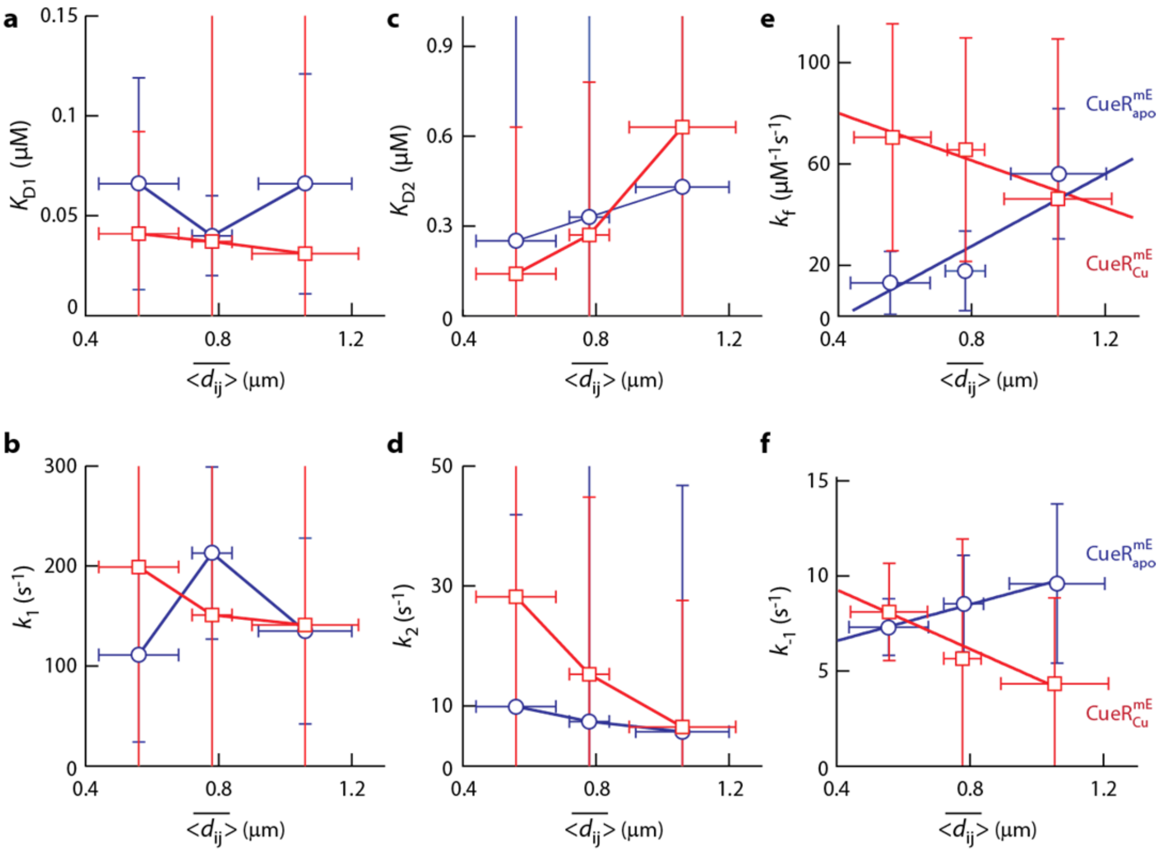

Supplementary Figure 51. $\overline{\langle d_{ij} \rangle}$ dependent (**a**) K_{D1}, (**b**) k_1, (**c**) K_{D2}, (**d**) k_2, (**e**) k_f and (**f**) k_{-1} for $CueR^{mE}_{apo}$ (blue circles) or $CueR^{mE}_{\mathbf{Cu}}$ (red squares) in non-dividing cells. Note that panels (**e**) and (**f**) are the same as part of Fig. 4d and e in the main text. All error bars are s.d.

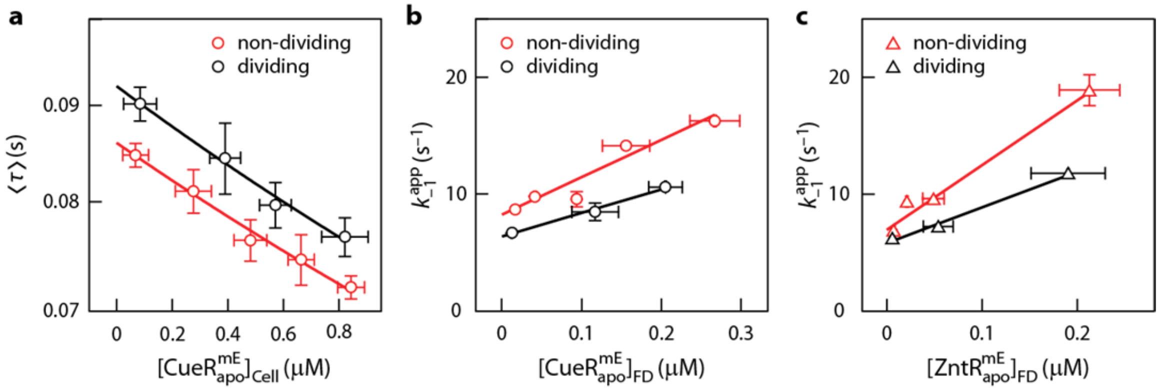

Supplementary Figure 52. Comparison of apo protein unbinding kinetics in dividing vs. nondividing cells. (**a**) Protein-concentration-dependent average residence time $\langle \tau \rangle$ for nondividing and dividing cells that express $CueR^{mE}_{apo}$. (**b**) Comparison of protein-concentration-dependent k^{app}_{-1} between nondividing and dividing cells that express $CueR^{mE}_{apo}$. (**c**) Same as (**b**), but for $ZntR^{mE}_{apo}$. All error bars are s.d.

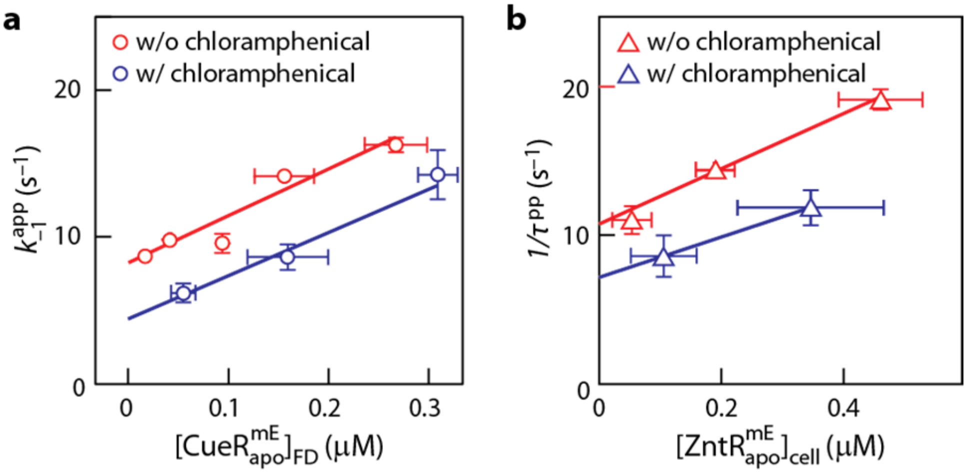

Supplementary Figure 53. Comparison of protein-concentration-dependent unbinding kinetics for cells expressing CueR$^{mE}_{apo}$ and ZntR$^{mE}_{apo}$ with and without chloramphenicol treatment. (**a**) k_{-1}^{app} of CueR$^{mE}_{apo}$ and (**b**) $1/\tau^{pp}$ of ZntR$^{mE}_{apo}$ at different cellular protein concentrations. All error bars are s.d.

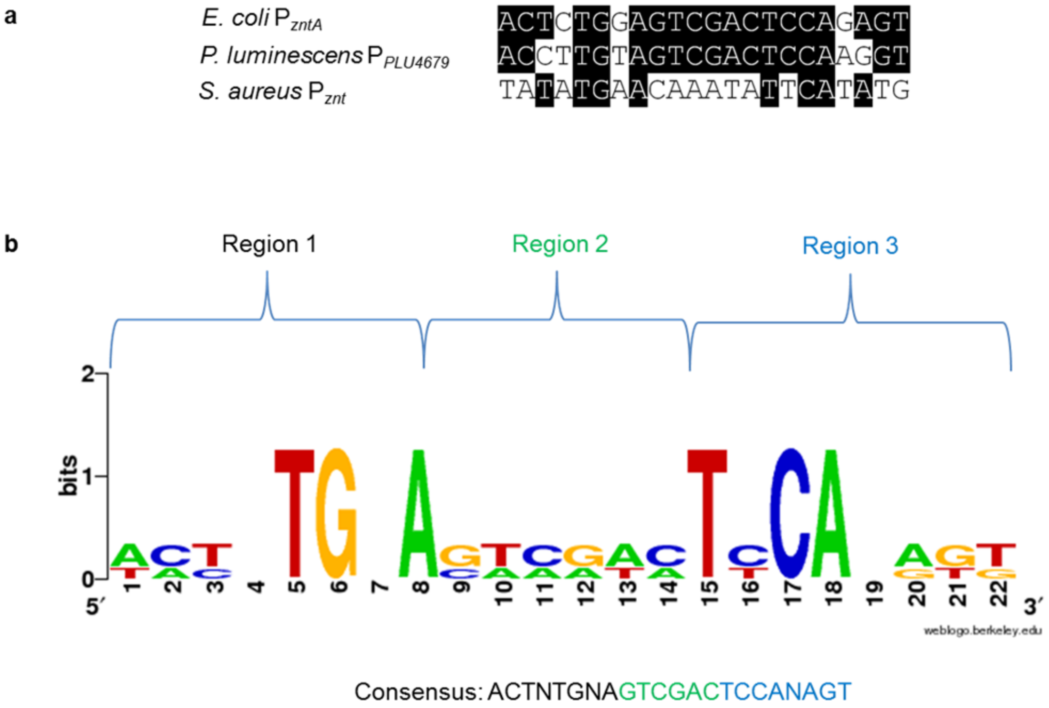

Supplementary Figure 54. Generating a consensus ZntR recognition sequence. (**a**) Alignment of known and putative promoter recognition sequences for ZntR from different organisms. **(b)** Sequence logo of the three aligned ZntR binding sites in (a), generated using the Weblogo software [111], showing the highly conserved bases (T(5), G(6), A(8), T(15), C(17), A(18)). Based on these alignments, the ZntR binding site sequence was partitioned into 3 regions: Region 1 (bases 1-8, first region containing highly conserved bases, T(5)G(6)A(8)), Region 2 (bases 9-14, variable region), Region 3 (bases 15-22, second region containing highly conserved bases, T(15)C(17)A(18)).

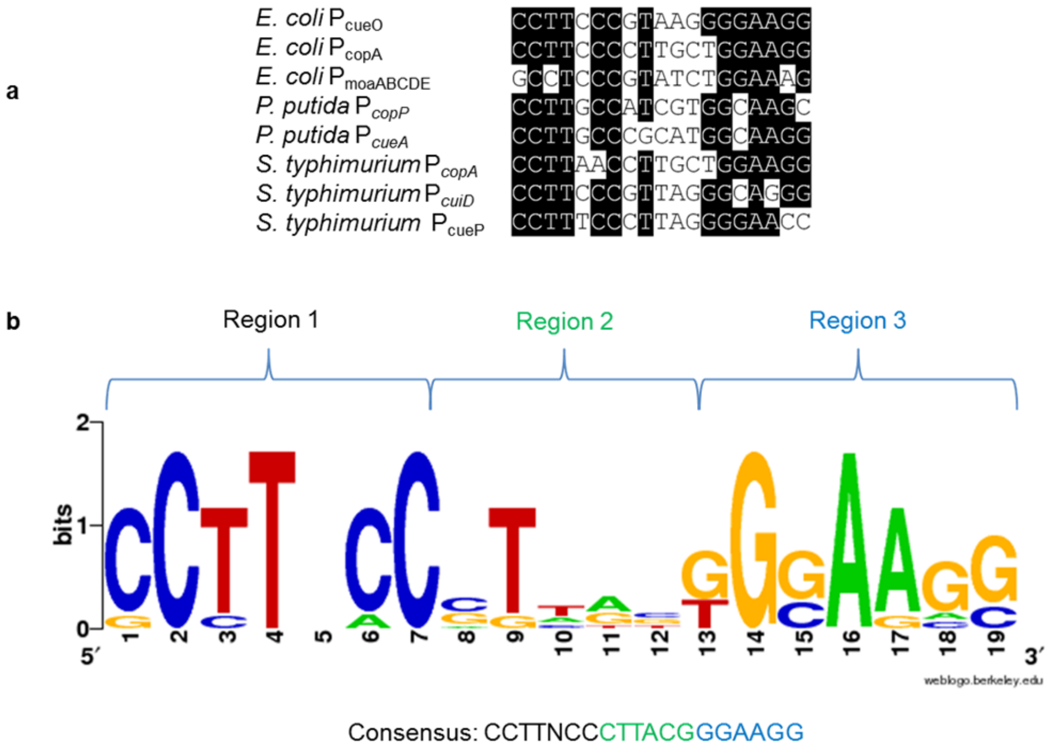

Supplementary Figure 55. Generating a consensus CueR recognition sequence. (a) Alignment of known and putative promoter recognition sequences for CueR from different organisms. (b) Sequence logo of the aligned CueR binding sites in (**a**) showing the highly conserved bases (C(2), T(4), C(7), G(14), A(16)). Based on these alignments, the CueR binding site sequence was partitioned into 3 regions: Region 1 (bases 1-7, first conserved region), Region 2 (bases 8-12, variable region), and Region 3 (bases 13-19, second conserved region).

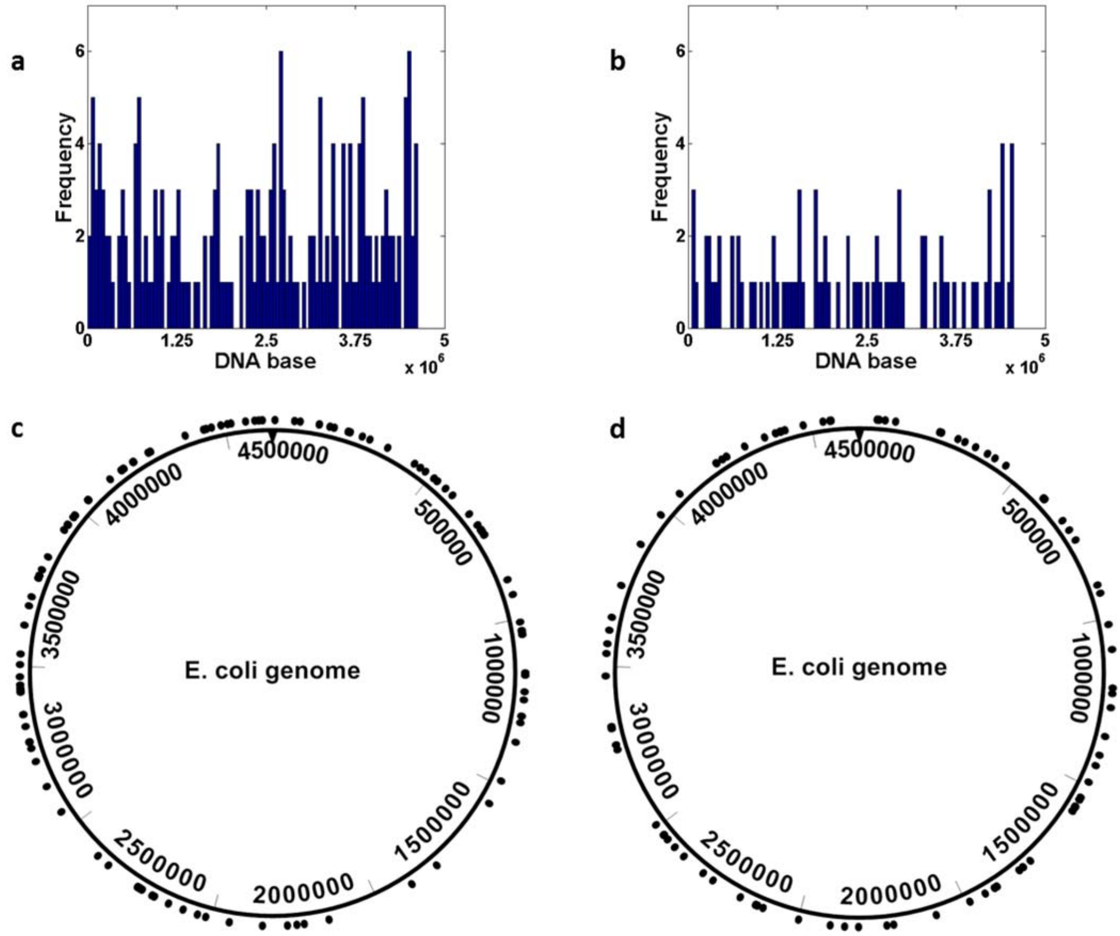

Supplementary Figure 56. Mapping potential CueR and ZntR recognition sites in the *E. coli* genome. Histograms of **(a)** CueR and **(b)** ZntR box-like sequences in the chromosome show no significant clustering in any particular region and that these sites are scattered throughout the entire length of the genome. A total of 197 recognition sites were identified for CueR, and 88 recognition sites for ZntR. Bin size of the histogram: 100 bases. The circular genome maps of **(c)** CueR and **(d)** ZntR recognition sites are also shown. The black ticks outside the circle correspond to the locations of the potential CueR or ZntR recognition sites in the genome.

Supplementary Table 1. Primers used for cloning in this study

Primer Name	Sequence (5'-3')
1. PstI-L-mEos3.2-f	AGTCAGCTGCAGGCTGGCTCCGCTGCTGGTTCTGGCGAATTCAGTGCGATTAAGCCAGAC
2. SalI-mEos3.2-r	AGTCAGGTCGACTTATCGTCTGGCATTGTCAG
3. pKDsal-f	AGTCAGGTCGACGTGTAGGCTGGAGCTGCTTC
4. pKDsac-r	AGTCAGGAGCTCCATATGAATATCCTCCTTAG
5. H1CueR-f	TATCGAAAATCTCTCCGGCTGCTGTCATCATCGGGCAGGGATGAGTGCGATTAAGCCAGA
6. H2CueR-r	TAGAAGGGATAACCCTACATATCCGAGCCGTCTCGTCTTAACGACGGCCAGTGAATTCGA (or AGAAGGGATAACCCTACATATCCGAGCCGTCTCGTCTTAACATATGAATATCCTCCTTAG)
7. H1ZntR-f	AGCTCTTGAACAAGGGGCGAGTGGCGTTAAGAGTGGTTGTATGAGTGCGATTAAGCCAGA
8. H2ZntR-r	GTTATTTAACGGCGCGAGTGTAATCCTGCCAGTGCAAAAAACGACGGCCAGTGAATTCGA (or TTATTTAACGGCGCGAGTGTAATCCTGCCAGTGCAAAAAACATATGAATATCCTCCTTAG)
9. PstI-mEos3.2-r	AGTCAGCTGCAGTTATCGTCTGGCATTGTCAG
10. EcoRI-CueR-f	AGTCAGGAATTCACCATGAACATCAGCGATGTAGC
11. EcoRI-ZntR-f	AGTCAGGAATTCACCATGTATCGCATTGGTGAGCT
12. CueRC112S-f	CACTGGCGAATGCCAGCCCTGGCGATGACAGC
13. CueRC112S-r	GCTGTCATCGCCAGGGCTGGCATTCGCCAGTG
14. ZntRC115S-f	CGCCTTAACGATGCCTGTTCAGGGACTGCTCATAGC
15. ZntRC115S-r	GCTATGAGCAGTCCCTGAACAGGCATCGTTAAGGCG
16. H1pCueO-f	GACGACCATTATTGTCGATTAAATTGTGTCTGCGGCTTGAGTGTAGGCTGGAGCTGCTTC
17. H2pCueO-r	TTCTTAAGCAGGCGAAACAAAATCAAACGTTGAGCATAGTCATATGAATATCCTCCTTAG
18. H1pZntA-f	TCACTTCCTGATCGTCCGCTCGCTGTATCTCTGATAAAACGTGTAGGCTGGAGCTGCTTC
19. H2pZntA-r	GACATGGCATCCTCCGGTTAAGTTTTTCTCATTAACCGACATATGAATATCCTCCTTAG
20. pCueO-f	CGGCTTGACCTTCCCGTAAG
21. pCueO-r	AATCGCCATCCCCATCTG
22. pZntA-f	CTGGAGTCGACTCCAGAGT
23. pZntA-r	CAACGTCACCAGACGGTCT
24. EcoRI-mEos3.2-f	AGTCAGGAATTCACCATGAGTGCGATTAAGCCAGA
25. PstI-mEos3.2-flag-r	TCAGCTGCAGTTATTTATCATCATCATCTTTATAATCAGGACGTCGTCTGGCATTGTCAG
26. H1dCueR-f	TGAAACCCTTTAACAAAGCACAGGAGGCGTTGCGCGAACGGTGTAGGCTGGAGCTGCTTC
27. H2dCueR-r	AGAAGGGATAACCCTACATATCCGAGCCGTCTCGTCTTAACATATGAATATCCTCCTTAG
28. PstI-CueR-r	AGTCAG CTGCAGTCACCCTGCCCGATGATGACA

Supplementary Table 2. Plasmids used or constructed in this study

Plasmid Name	Vector Backbone	Gene Insert	Resistance
1. pmEos3.2	pEGFP-N1	*mEos3.2*	Kanamycin (Kan)
2. pUCmEos3.2	pUC19	*mEos3.2*	Ampicillin (Amp)
3. pKD3	pANTSγ	*cat*	Amp and chloramphenicol (Cam)
4. pUCmEos3.2:cat	pUC19	*mEos3.2:cat*	Amp, Cam
5. pBCueR-mEos3.2	pBAD24	*cueR-mEos3.2*	Amp
6. pBZntR-mEos3.2	pBAD24	*zntR-mEos3.2*	Amp
7. pBCR(C112S)-mEos3.2	pBAD24	*cueR(C112S)-mEos3.2*	Amp
8. pBZR(C115S)-mEos3.2	pBAD24	*zntR(C115S)-mEos3.2*	Amp
9. pBmEos3.2	pBAD24	*mEos3.2*	Amp
10. pBZntR-mEos3.2-FLAG	pBAD24	*zntR-mEos3.2-FLAG*	Amp
11. pBCueR-mEos3.2-FLAG	pBAD24	*cueR-mEos3.2-FLAG*	Amp
12. pBmEos3.2-FLAG	pBAD24	*mEos3.2-FLAG*	Amp
13. pKD46	pINT-ts	*γ,β,exo enzymes*	Amp

Supplementary Table 3. Strains used or constructed in the current study

Strain Name	Plasmid	Chromosomal Gene Tag/Modification	Resistance
1. CRM32	none	*cueR-mEos3.2*	Cam
2. ZRM32	none	*zntR-mEos3.2*	Cam
3. CRM32-p	pBCueR-mEos3.2	*cueR-mEos3.2*	Amp, Cam
4. ZRM32-p	pBZntR-mEos3.2	*zntR-mEos3.2*	Amp, Cam
5. JW0476-1	None	*ΔcueR*	Kan
6. JW3254-5	None	*ΔzntR*	Kan
7. DCR-pACRM32	pBCR(C112S)-mEos3.2	*ΔcueR*	Amp, Kan
8. DZR-pAZRM32	pBZR(C115S)-mEos3.2	*ΔzntR*	Amp, Kan
9. JW0476-1DPCueO	None	*ΔcueR*, ΔP_{cueO}	Kan, Cam
10. JW3254-5DPZntA	None	*ΔzntR*, ΔP_{zntA}	Kan, Cam
11. JW0476-1λ	pKD46	*ΔcueR*	Kan, Amp
12. JW3254-5λ	pKD46	*ΔzntR*	Kan, Amp
13.DCRPCO-pBCRM32	pBCueR-mEos3.2	*ΔcueR*, ΔP_{cueO}	Kan, Cam, Amp
14. DZRPZA-pBZRM32	pBZntR-mEos3.2	*ΔzntR*, ΔP_{zntA}	Kan, Cam, Amp
15. BW25113	pKD46	*none (experimental wild-type)*	Amp
16. BWPM32	pBmEos3.2	*none*	Amp
17. CRM32-pBAD	pBAD24	*cueR-mEos3.2*	Amp, Cam
18. DZR-pBZRM32FLAG	pBZntR-mEos3.2-FLAG	*ΔzntR*	Amp, Kan
19. DCR-pBCRM32FLAG	pBCueR-mEos3.2-FLAG	*ΔcueR*	Amp, Kan
20. DZR-pBAD	pBAD24	*ΔzntR*	Amp, Kan
21. JW0560-1	none	*ΔcusR*	Kan
22. JW0560-1λ	pKD46	*ΔcusR*	Kan, Amp
23. JW0560-1DCR	none	*ΔcusR, ΔcueR*	Kan, Cam
24. DCSR-CRM32	none	*ΔcusR, cueR-mEos3.2*	Kan, Cam
25. DCR-pBM32FLAG	pBmEos3.2-FLAG	*ΔcueR*	Kan, Amp

Supplementary Table 4. Fitted effective diffusion constants of CueR and ZntR variants in live *E. coli* cells [a]

	D_{FD} ($\mu m^2 s^{-1}$)	D_{NB} ($\mu m^2 s^{-1}$)	D_{SB} ($\mu m^2 s^{-1}$)
$CueR^{mE}_{apo}$	3.7 ± 0.2	0.70 ± 0.03	0.036 ± 0.009
$CueR^{mE}_{apo}$ (Dividing)	4.2 ± 0.4	0.70 ± 0.02	0.047 ± 0.009
$CueR^{mE}_{Cu}$	3.7 ± 0.3	0.67 ± 0.04	0.036 ± 0.004
$CueR^{mE}$	4.2 ± 0.3	0.76 ± 0.04	0.046 ± 0.015
ΔP_{cueO}	3.4 ± 0.1	0.64 ± 0.02	0.044 ± 0.004
$ZntR^{mE}_{apo}$	4.0 ± 0.2	0.74 ± 0.04	0.036 ± 0.003
$ZntR^{mE}_{apo}$ (Dividing)	4.2 ± 0.2	0.76 ± 0.03	0.034 ± 0.009
$ZntR^{mE}_{Zn}$	3.7 ± 0.1	0.73 ± 0.04	0.036 ± 0.006
$ZntR^{mE}$	3.5 ± 0.1	0.76 ± 0.04	0.046 ± 0.014
ΔP_{zntA}	3.6 ± 0.1	0.75 ± 0.04	0.051 ± 0.015

[a]: All experiments are carried out in (metal-depleted) M9 medium except for $CueR^{mE}_{Cu}$/$ZntR^{mE}_{Zn}$ for which the cells were grown in the presence of 100 µM Cu^{2+}/Zn^{2+} in the medium (see Section 4.1 for details).

Supplementary Table 5. Fitted fractional populations of CueR and ZntR variants in live *E. coli* cells [a]

	$[P]_{cell}$ (nM)			A_{FD} (%)			A_{NB} (%)			A_{SB} (%)		
$CueR^{mE}_{apo}$	99	±	19	16.7	±	0.2	54.2	±	0.1	29.1	±	0.2
	180	±	34	23.2	±	0.3	49.2	±	0.4	27.6	±	0.5
	338	±	28	27.8	±	0.3	51.7	±	0.4	20.6	±	0.6
	553	±	105	28.2	±	0.4	58.5	±	0.5	13.3	±	0.6
	926	±	108	28.9	±	0.3	62.0	±	0.3	9.1	±	0.4
	1375	±	40	30.1	±	0.4	65.5	±	0.6	3.7	±	0.7
$CueR^{mE}_{apo}$ (Dividing)	81	±	54	15.2	±	0.2	53.4	±	0.1	31.4	±	0.3
	461	±	115	25.4	±	0.3	59.1	±	0.4	15.5	±	0.7
	821	±	84	25.0	±	0.3	66.6	±	0.4	8.4	±	0.8
	1227	±	86	23.3	±	0.4	72.3	±	0.5	4.5	±	0.8
$CueR^{mE}_{Cu}$	115	±	29	16.3	±	0.3	48.1	±	0.4	35.6	±	0.5
	231	±	42	20.9	±	0.2	49.8	±	0.2	29.3	±	0.3
	342	±	20	15.9	±	0.3	46.6	±	0.4	37.5	±	0.5
	456	±	47	24.7	±	0.3	61.1	±	0.2	14.2	±	0.3
	612	±	45	35.8	±	0.3	56.1	±	0.3	8.1	±	0.4
	877	±	101	31.0	±	0.3	65.1	±	0.3	3.9	±	0.4
$CueR^{mE}$	37	±	14	13.0	±	0.6	44.1	±	0.9	42.9	±	1.1
	115	±	29	14.6	±	0.3	55.6	±	0.4	29.8	±	0.4
	272	±	64	20.3	±	0.2	56.5	±	0.3	23.2	±	0.3
	592	±	68	31.8	±	0.3	60.8	±	0.3	7.4	±	0.4
	789	±	60	32.2	±	0.3	64.7	±	0.3	3.1	±	0.4
ΔP_{cueO}	64	±	19	19.0	±	0.3	47.2	±	0.4	33.8	±	0.5
	327	±	126	24.8	±	0.4	55.9	±	0.5	19.3	±	0.6
	560	±	39	27.4	±	0.5	58.5	±	0.4	14.0	±	0.6
	779	±	112	26.4	±	0.5	63.0	±	0.5	10.6	±	0.7
	798	±	19	25.3	±	0.6	68.9	±	0.7	5.8	±	1.0
$ZntR^{mE}_{apo}$	38	±	15	19.6	±	0.2	43.7	±	0.2	36.7	±	0.3
	94	±	14	22.4	±	0.2	47.6	±	0.2	30.0	±	0.4
	175	±	40	28.0	±	0.2	54.8	±	0.2	17.3	±	0.4
	432	±	50	45.1	±	0.5	51.0	±	0.6	4.0	±	1.1
$ZntR^{mE}_{apo}$ (Dividing)	49	±	30	12.1	±	0.2	32.5	±	0.2	55.4	±	0.3
	255	±	74	21.2	±	0.3	61.1	±	0.3	17.7	±	0.5
	511	±	104	37.3	±	0.3	51.2	±	0.3	11.5	±	0.5
	814	±	22	44.5	±	0.3	51.0	±	0.3	4.4	±	0.6
$ZntR^{mE}_{Zn}$	55	±	11	15.8	±	0.3	33.2	±	0.4	51.0	±	0.7
	102	±	21	30.0	±	0.3	35.5	±	0.3	34.5	±	0.5
	243	±	67	41.7	±	0.4	42.2	±	0.4	16.2	±	0.7
	430	±	46	37.3	±	0.4	53.1	±	0.4	9.6	±	0.9
	601	±	53	44.9	±	0.4	48.6	±	0.5	6.4	±	0.9
$ZntR^{mE}$	60	±	8	18.5	±	0.3	48.3	±	0.3	33.2	±	0.5
	106	±	20	26.1	±	0.2	38.9	±	0.2	35.0	±	0.5
	203	±	23	35.7	±	0.3	44.4	±	0.3	19.9	±	0.6

	351 ± 65	40.8 ± 0.3	48.4 ± 0.3	10.8 ± 0.6
	494 ± 24	51.1 ± 0.4	44.7 ± 0.4	4.2 ± 0.7
ΔP_{zntA}	46 ± 18	19.3 ± 0.2	39.8 ± 0.2	40.9 ± 0.3
	125 ± 25	30.9 ± 0.3	44.7 ± 0.3	24.4 ± 0.6
	228 ± 48	40.5 ± 0.3	46.8 ± 0.3	12.8 ± 0.5
	381 ± 41	40.4 ± 0.3	53.9 ± 0.4	5.7 ± 0.7
	693 ± 111	48.9 ± 0.3	50.0 ± 0.3	1.4 ± 0.6

[a]: All experiments were carried out in (metal-depleted) M9 medium except for $\mathrm{CueR}^{\mathrm{mE}}_{\mathrm{Cu}}/\mathrm{ZntR}^{\mathrm{mE}}_{\mathrm{Zn}}$ for which the cells were grown in the presence of 100 μM Cu^{2+}/Zn^{2+} in the medium (see Section 4.1 for details).

Supplementary Table 6. Input parameters[a] used in 3-state (or 2-state) simulations and the parameters extracted from analyzing the CDF of r and the residence time distributions from the simulations. Note the number 1, 2, and 3 for γ's correspond to NB, SB, and FD states, respectively

Parameters	Input in simulation	Results from analyzing the simulated trajectories
D_1 ($\mu m^2 s^{-1}$)	11	3.55[b] (Expected: 3.7 ± 0.2)[a]
D_2 ($\mu m^2 s^{-1}$)	0.70	0.74 [b] (Expected: 0.70 ± 0.03)[a]
D_3 ($\mu m^2 s^{-1}$)	0.036	0.037 [b] (Expected: 0.036 ± 0.009)[a]
k_{bl} (s^{-1})	252	269 ± 1 [c]
3-state simulation corresponding to [$CueR^{mE}_{apo}$] = 180 nM (Input A_1 = 23.2%, A_2 = 49.2%, A_3 = 27.6%) [d]		
γ_{12} ($k_2[D]_{NB}$) (s^{-1})	5.3	
γ_{21} (k_{-2}) (s^{-1})	2.5	
γ_{13} ($k_1[D]_{SB}$) (s^{-1})	11.4	
γ_{31} (k^{app}_{-1}) (s^{-1})	9.6	6.9 ± 0.3 [d]
3-state simulation corresponding to [$CueR^{mE}_{apo}$] = 338 nM (Input A_1 = 27.8%, A_2 = 51.7%, A_3 = 20.6%) [d]		
γ_{12} ($k_2[D]_{NB}$) (s^{-1})	4.7	
γ_{21} (k_{-2}) (s^{-1})	2.5	
γ_{13} ($k_1[D]_{SB}$) (s^{-1})	8.3	
γ_{31} (k^{app}_{-1}) (s^{-1})	11.2	9.5 ± 0.2[d]
3-state simulation corresponding to [$CueR^{mE}_{apo}$] = 926 nM (Input A_1 = 28.9%, A_2 = 62.0%, A_3 = 9.1%) [d]		
γ_{12} ($k_2[D]_{NB}$) (s^{-1})	5.4	
γ_{21} (k_{-2}) (s^{-1})	2.5	
γ_{13} ($k_1[D]_{SB}$) (s^{-1})	5.3	
γ_{31} (k^{app}_{-1}) (s^{-1})	16.8	14.4 ± 1.0 [d]
2-state simulation corresponding to [$CueR^{mE}_{apo}$] = 1375 nM (Input A_1 = 31.5%, A_2 = 68.5%) [d]		
γ_{12} ($k_2[D]_{NB}$) (s^{-1})	5.4	
γ_{21} (k_{-2}) (s^{-1})	2.5	12.4 ± 2.3 [e]

[a] Input parameters and expected values are taken from Supplementary Table 4 and Supplementary Table 7. [b] From analyzing the CDFs of r from the simulations; the fitting error bars are <0.001. [c] From analyzing the length distribution of simulated trajectories. [d] From analyzing the distribution of the residence times from the 3-state simulations. [e] From analyzing the distribution of the residence times from the 2-state simulations (i.e., without the SB state). [d] The input fractional amplitudes are determined by the input rate constants k's, and are consistent with those in Supplementary Table 4.

Supplementary Table 7. Kinetic and thermodynamic parameters for CueR−DNA interactions in *E. coli* cells [a]

Kinetic Parameters	$CueR^{mE}_{apo}$	$CueR^{mE}_{apo}$ (Dividing cells)	$CueR^{mE}_{Cu}$	$CueR^{mE}$	$CueR^{mE}$ + ΔP_{cueO}
k_1 ($\mu M^{-1}s^{-1}$)	214 ± 46	219 ± 77	54 ± 96	43 ± 72	10 ± 52
k_{-1} (s^{-1})	8.2 ± 0.9	6.4 ± 1.1	4.6 ± 0.8	3.1 ± 1.2	3.5 ± 2.1
k_f ($\mu M^{-1}s^{-1}$)	31.9 ± 6.9	20.1 ± 8.7	55.0 ± 8.5	70.6 ± 17.7	58.6 ± 15.5
K_{D1}(= k_{-1}/k_1) (μM)	0.037 ± 0.028	0.029 ± 0.011	0.038 ± 0.058	0.027 ± 0.028	0.047 ± 0.139
k_2 ($\mu M^{-1}s^{-1}$)	3.6 ± 1.9	1.6 ± 6.1	4.9 ± 3.8	25.0 ± 10.6	7.6 ± 16.4
k_{-2} (s^{-1})	2.5 ± 0.1	2.6 ± 0.2	4.1 ± 0.1	6.1 ± 0.1	9.4 ± 0.1
K_{D2}(= k_{-2}/k_2) (μM)	0.69 ± 0.38	1.6 ± 6.3	0.83 ± 0.65	0.24 ± 0.10	1.24 ± 2.66
K_{D3}(= k_{-3}/k_3)	0.06 ± 0.03	0.02 ± 0.08	0.04 ± 0.05	0.11 ± 0.09	0.03 ± 0.07
N_{NB}	2605 ± 1381	8941 ± 34406	2588 ± 1976	1093 ± 439	4312 ± 9251
N_{SB}	130 ± 17	153 ± 36	121 ± 89	111 ± 69	141 ± 26

[a]: All strains were imaged in (metal-depleted) m9 medium except for $CueR^{mE}_{Cu}$, for which the cells were imaged in the presence of 100 μM Cu^{2+}.

Supplementary Table 8. Kinetic and thermodynamic parameters for ZntR−DNA interactions in *E. coli* cells [a]

Kinetic Parameters	$ZntR^{mE}_{apo}$	$ZntR^{mE}_{apo}$ (Dividing cells)	$ZntR^{mE}_{Zn}$	$ZntR^{mE}$	$ZntR^{mE}$ + ΔP_{zntA}
k_1 ($\mu M^{-1}s^{-1}$)	379 ± 161	1143 ± 472	1593 ± 264	728 ± 329	1310 ± 562
k_{-1} (s^{-1})	7.0 ± 0.8	5.8 ± 2.0	5.7 ± 0.8	12.3 ± 1.3	9.8 ± 1.4
k_f ($\mu M^{-1}s^{-1}$)	55.4 ± 15.6	30.6 ± 21.6	61.6 ± 12.8	66.6 ± 28.3	39.5 ± 16.6
K_{D1}(= k_{-1}/k_1)(μM)	0.018 ± 0.008	0.005 ± 0.003	0.004 ± 0.001	0.017 ± 0.008	0.007 ± 0.003
k_2 ($\mu M^{-1}s^{-1}$)	31.6 ± 5.1	34.5 ± 32.6	32.8 ± 44.5	46.4 ± 21.3	55.7 ± 65.5
k_{-2} (s^{-1})	6.0 ± 0.2	7.8 ± 0.5	11.5 ± 1.0	9.1 ± 0.2	10.3 ± 0.1
K_{D2}(= k_{-2}/k_2) (μM)	0.19 ± 0.03	0.23 ± 0.21	0.35 ± 0.47	0.20 ± 0.09	0.19 ± 0.22
K_{D3}(= k_{-3}/k_3)	0.07 ± 0.03	0.02 ± 0.02	0.02 ± 0.02	0.07 ± 0.05	0.04 ± 0.05
N_{NB}	580 ± 87	1181 ± 1060	759 ± 997	500 ± 214	444 ± 502
N_{SB}	67 ± 19	89 ± 12	55 ± 3	68 ± 17	42 ± 10

[a]: All cells were imaged in (metal-depleted) M9 medium except for $ZntR^{mE}_{Zn}$, for which the cells were imaged in the presence of 100 μM Zn^{2+}. Parameters were extracted without correcting for the ZntR degradation in the cell.

Supplementary Table 9. Summary of relative metal binding affinities (i.e., dissociation constants) of DNA-bound and free CueR using different literature values of DNA binding affinities of apo- and holo-CueR

Experiments [a]	Dissociation constant of apo-protein from DNA, $K_{D,PD}^{apo}$ (nM)	Dissociation constant of holo-protein from DNA, $K_{D,PD}^{holo}$ (nM)	Ratio of dissociation constants for metal binding, $\frac{K_{D,M}^{PD}}{K_{D,M}^{P}}$
Single-molecule imaging[50]	39 ± 16	52 ± 36	1.33 ± 1.07
Ensemble anisotropy titration[67]	6 ± 2	1.9 ± 0.8	0.32 ± 0.17
Gel shift assay[49]	17 ± 2	25 ± 7	1.47 ± 0.45

[a] Different experiments were used in the literature to measure the dissociation constant of protein-DNA complexes.

Supplementary Table 10. Fitted kinetic and thermodynamic parameters of CueR variants for sorted cell groups with different extents of chromosome condensations

Parameters	$CueR_{apo}^{mE}$			$CueR_{Cu}^{mE}$		
$\overline{\langle d_{ij} \rangle}$ (μm)	0.56 ± 0.12	0.78 ± 0.06	1.06 ± 0.14	0.56 ± 0.12	0.78 ± 0.06	1.06 ± 0.16
k_1 (μM^{-1}s^{-1})	111 ± 87	213 ± 86	135 ± 93	199 ± 244	151 ± 561	141 ± 762
k_{-1} (s^{-1})	7.3 ± 1.5	8.5 ± 2.5	9.6 ± 4.2	8.1 ± 2.6	5.6 ± 6.3	4.3 ± 4.5
k_f (μM^{-1}s^{-1})	13.1 ± 12.4	17.8 ± 15.7	56.1 ± 25.7	70.4 ± 44.8	65.5 ± 44.1	46.1 ± 63.0
K_{D1}(μM)	0.066 ± 0.053	0.040 ± 0.020	0.066 ± 0.055	0.041 ± 0.051	0.037 ± 0.145	0.031 ± 0.168
k_2 (μM^{-1}s^{-1})	9.9 ± 32	7.4 ± 49.5	5.7 ± 41.1	28.2 ± 96	15.3 ± 29.5	6.5 ± 21.1
k_{-2} (s^{-1})[a]	2.5 ± 0.1	2.5 ± 0.1	2.5 ± 0.1	4.1 ± 0.1	4.1 ± 0.1	4.1 ± 0.1
K_{D2} (μM)	0.25 ± 0.81	0.33 ± 2.23	0.43 ± 3.1	0.14 ± 0.49	0.27 ± 0.51	0.63 ± 2.02
K_{D3}	0.20 ± 0.73	0.26 ± 0.39	0.05 ± 0.76	0.21 ± 0.68	0.07 ± 0.26	0.04 ± 0.2
N_{NB}	1934 ± 6079	1418 ± 9279	1693 ± 12000	509 ± 1580	1022 ± 1904	1421 ± 4491
N_{SB}	294 ± 61	153 ± 32	223 ± 65	139 ± 95	85 ± 221	98 ± 246

[a] k_{-2} is obtained by fitting the distribution of residence time from all non-dividing cells from Supplementary Table 7.

Supplementary Table 11. Fitted kinetic parameters of ZntR variants for sorted cell groups with different extents of chromosome condensations

Parameters	$ZntR_{apo}^{mE}$			$ZntR_{Zn}^{mE}$		
$\overline{\langle d_{ij} \rangle}$ (μm)	0.52 ± 0.14	0.79 ± 0.06	1.09 ± 0.18	0.54 ± 0.13	0.78 ± 0.06	1.04 ± 0.13
k_{-1} (s^{-1})	2.9 ± -	3.8 ± -	5.2 ± -	- ± -	- ± -	4.18 ± -
k_f (μM^{-1}s^{-1})	135.5 ± -	120.3 ± -	113.9 ± -	- ± -	- ± -	44.4 ± -

Table S12. Selected hits from BLAST search of *E. coli* K-12 genome for potential ZntR recognition sites [a]

Sequence hits	Location in the genome	Number of Mismatches in regions		
		R1	R3	R1+R3
1. ACTCTGGAGTCGACTCCAGAGT	at the promoter of *zntA*	0	0	0
2. TCTCTGTAAAAGATTCCACAGT	within the gene *yaiT*	1	0	1
3. GCTATGCAGAAATTTGCACAGT	within the *ato operon*	1	0	1
4. ACTTTCTAAGGCCGTGCAAAGT	within the gene of *nsrR*	1	0	1

[a] Red letters correspond to mismatch with respect to the consensus sequence.

Supplementary Table 13. Selected hits from BLAST search of *E. coli* K-12 genome for potential CueR recognition sites [a]

Sequence	Description	Number of Mismatch		
		R1	R3	R1+R3
1. CCTTCCCGTAAGGGGAAGG	at the promoter region of *cueO*	0	0	0
2. CCTTCCCCTTGCTGGAAGG	at the promoter region of *copA*	0	0	0
3. GCCTCCCGTATCTGGAAAG	at the promoter region of *moaABCDE* operon	2	1	3
4. CCTTCCGGGGAAGGGAAGG	within the gene of *aat*	1	0	1
5. CCCTCCCGAGTGCGGAAGG	At the promoter region of *yafC*	1	0	1
6. CCTTCCCCTGAATGGGAGG	Within the gene of *pheL*	0	1	1
7. CCTGCCCGGTGCCGGAAGG	Within the gene of *rrlC*	1	0	1
8. CCTGCCCGGTGCCGGAAGG	Within the gene of *rrlA*	1	0	1
9. CCTGCCCGGTGCCGGAAGG	Within the gene of *rrlB*	1	0	1
10. CCTGCCCGGTGCCGGAAGG	Within the gene of *rrlE*	1	0	1
11. CCTGCCCGGTGCCGGAAGG	Within the gene of *rrlH*	1	0	1
12. CCTTACCGCCGCTGGAAGG	Within the gene of *garD*	1	0	1

[a] Red letters correspond to mismatch with respect to the consensus sequence.

Supplementary Notes

1. Schematic overview of experimental strategy

The schematic overview of experimental strategy is illustrated in Supplementary Figure 1.

2. Construction of plasmids and strains

2.1 Tagging E. coli chromosomal genes with mEos3.2 (i.e., mE) using λ-Red Technique

2.1.1 Making the plasmid template containing mEos3.2:cat cassette (Steps 1 and 2 in Supplementary Figure 2)

The *mEos3.2* gene was cloned from the plasmid pmEos3.2 [4] (Supplementary Figure 2) using primers PstI-L-mEos3.2-f and SalI-mEos3.2-r (Supplementary Table 1 summarizes all primers used) and AccuprimerPfx DNA Polymerase (Life Technologies, cat. #: 12344024). The PCR product (purified using Wizard SV Gel and PCR Clean-Up System, Promega, A9281) was digested with PstI-HF (NEB, R3140S) and SalI-HF (NEB, R3138S) and inserted into a similarly digested pUC19 vector (Lucigen) using Quick Ligase enzyme (NEB, M2200S), giving pUCmEos3.2 (Step 1 in Supplementary Figure 2; Supplementary Table 2 summarizes all plasmids used/constructed in this study), which was purified using Qiaprep Spin Miniprep (Qiagen, 27106) then transformed into and propagated in E. cloni 10G cells (Lucigen, 60106-1). The chloramphenicol resistance cassette (*cat*), flanked by FRT sequences on both sides (FRT-cat-FRT), was cloned from plasmid pKD3 (Keio Collection, CGSC #7631) using primers pKDsal-f and pKDsac-r (Step 2 in Supplementary Figure 2, Supplementary Table 1). The FRT sequences are necessary elements for the Flippase (FLP)-mediated removal, if needed, of the *cat* gene after chromosomal integration of the linear DNA insert [1]. (Note: the FLP enzyme was not used in this study and all FRT-cat-FRT insertions were maintained in the genome for antibiotic selection.) The product was digested with SalI-HF and SacI-HF (R3156S) and then ligated into a similarly digested pUCmEos3.2 vector to give the plasmid pUCmEos3.2:cat (Supplementary Table 2), which was transformed into and propagated in E. cloni 10G cells. All plasmid insertions were screened by colony PCR and confirmed by DNA sequencing.

2.1.2 C-Terminal tagging of chromosomal cueR or zntR gene with mEos3.2 via λ-Red Homologous recombination

<u>Making the linear DNA template (Step 3 in Supplementary Figure 2)</u>. The plasmid pUCmEos3.2:cat was digested with EcoRI-HF (NEB, R3101S) restriction enzyme and gel-purified to obtain the linear *mEos3.2:cat* DNA cassette template. (Note: If linker is desired in the final linear DNA template, use SbfI-HF (NEB, R3642S) and SacI-HF (R3156S) double digestion instead (Supplementary Figure 2)). This linearization step was introduced to prevent the plasmid pUCmEos3.2:cat from contaminating the linear DNA insert; possible contamination would make the drug selection of the transformed cells difficult.

<u>Making linear DNA inserts (Step 4 in Supplementary Figure 2)</u>. For the linear DNA insert to tag chromosomal *cueR* gene, primers H1CueR-f and H2CueR-r were used to copy *mEos3.2:cat* from the linear *mEos3.2:cat* DNA cassette template to obtain a DNA fragment containing *mEos3.2:cat* with flanking homology regions (i.e., H1 and H2 in Supplementary Figure 2, oriented in the sense/coding (5′-3′) strand with respect to the *mEos3.2* gene; H1 is the last 40 bp of the *cueR* gene excluding the stop codon while H2 is the next 41 bp after the *cueR* stop codon). Similarly, for tagging *zntR*, the linear DNA insert with flanking homology regions was obtained via PCR using primers H1ZntR-f and H2ZntR-r and the *mEos3.2:cat* as the template (H1 is the last 40 bp of *zntR* gene excluding the stop codon while the H2 is the next 41 bp after the *zntR* stop codon). The linear DNA insert was purified through a PCR Clean-up System (Promega) and eluted with nuclease-free water.

Homologous Recombination via λ-Red (Step 5 in Supplementary Figure 2). Electrocompetent cells were prepared by first culturing for 18 hours in 30°C *E. coli* BW25113 cells (CGSC# 7739 Keio Collection, Yale; genotype: (F- *Δ(araD-araB)567*, *ΔlacZ4787*(::rrnB-3), *λ*$^{-}$, *rph-1*, *Δ(rhaD-rhaB)568*, *hsdR514*) harboring the temperature-sensitive pKD46 plasmid in LB (Sigma-Aldrich, cat.#: L3022-6X1KG) with ampicillin (100 µg/mL, USBiological). Note that beyond 30°C, the pKD46 plasmid can no longer be replicated and will be lost in subsequent generations. From this culture, a 1:100 dilution was prepared in SOB medium (2% w/v Bacto Tryptone (Sigma-Aldrich, cat.#: T9410), 0.5 % w/v Bacto Yeast Extract (Sigma-Aldrich, cat.#: Y1625), 10 mM NaCl (Macron, 7581-12), 2.5 mM KCl (Fisher Scientific, P217-500), 10 mM $MgCl_2$ (Mallinckrodt, 5958-04), and 10 mM $MgSO_4$ (Fisher Scientific, M63-500) all in nanopure sterile water) containing ampicillin (100 µg/mL) and 20 mM L-arabinose (Sigma-Aldrich, cat. #: A3256). This SOB culture was incubated at 30°C with shaking at 250 rpm until OD_{600} = 0.6. The cells were centrifuged and washed thrice with cold 10% glycerol (Macron, 5092-02) in nanopure sterile water. The cells were diluted to a final volume of 25 µL in 10% glycerol in nanopure sterile water.

The linear DNA insert (100 ng in nuclease-free water) was electroporated (2.5 kV, using MicroPulser Electroporator cat. #: 1652100, Bio-Rad) into BW25113 cells expressing the recombinase enzymes (*exo*, β, γ) from pKD46 (electroporation cuvette: Bio-Rad 0.2 cm gap, cat. #1652086). The electroporated cells were diluted in 1 mL SOC medium (SOB medium containing 20 mM glucose (Sigma-Aldrich, cat. #: G7528)) and then incubated at 37°C with shaking at 250 rpm for 4 hours. The cells were plated onto LB-agar containing chloramphenicol (10 µg/mL, USBiological) and incubated at 37°C for 18 h. About 10-20 colonies (1 mm diameter each) grew on the plate after incubation, and 8 colonies were chosen for screening. Successful integration of the *mEos3.2:cat* at the chromosomal target site was confirmed by colony PCR screening and sequence analysis of PCR fragments amplified from the genomic extract (Wizard Genomic DNA Purification Kit, Promega, cat. #: A1120).

To eliminate the temperature-sensitive pKD46 plasmid after homologous recombination, the selected colony was streaked onto chloramphenicol-containing LB-agar plate and incubated at 42°C for 18 h. Only ampicillin-sensitive (i.e., the drug resistance carried by pKD46) and chloramphenicol-resistant (due to the *cat* gene inserted in the genome) colonies were propagated and used for imaging experiments. The resulting recombinant strains are shown in Supplementary Table 3, which also summarizes all of the constructed strains used in this study. All chromosomal insertions were confirmed by colony PCR and DNA sequencing. The resulting strain containing *cueR-mEos3.2* in the genome is called CRM32 while *zntR-mEos3.2* is called ZRM32.

2.2 Making the cueR-mEos3.2 and the zntR-mEos3.2 gene fusions in the arabinose-inducible plasmid pBAD24

Using the genomic extract of strain CRM32 whose chromosomal *cueR* gene was tagged with *mEos3.2* (all strains used or constructed in the study are summarized in Supplementary Table 3), we cloned out the *cueR-mEos3.2* gene fragment using primers EcoRI-CueR-f and PstI-mEos3.2-r (Supplementary Table 1). The resulting PCR fragment was digested with EcoRI-HF and PstI-HF, and then inserted into a similarly digested pBAD24 plasmid (CGSC#: 12523), giving pBCueR-mEos3.2 (Supplementary Table 2). The pBAD24 plasmid was chosen as the parent vector because it contains suitable restriction sites that allow us to insert fusion genes whose expression will be under the control of a tightly-regulated arabinose operon promoter [5]. All insertions were confirmed by colony PCR and DNA sequencing. The restriction sites and primers here were chosen and designed in such a way that the $CueR^{mE}$ protein, once expressed from the plasmid, results in exactly the same protein as the one expressed from the chromosomal *cueR-mEos3.2* gene without extra amino acids at either N- or C-terminal of the fusion protein. *The same*

attention was given to any plasmid-expressed fusion genes in this study, so they all translate into the same proteins as those expressed from the chromosome.

Similarly, *zntR-mEos3.2* fragment was amplified from the genomic extract of strain ZRM32 (Supplementary Table 3) using primers EcoRI-ZntR-f and PstI-mEos3.2-r (Supplementary Table 1). The resulting PCR fragment was digested with EcoRI-HF and PstI-HF, and then ligated into a similarly digested pBAD24, giving pBZntR-mEos3.2 (Supplementary Table 2).

Plasmids pBCueR-mEos3.2 and pBZntR-mEos3.2 were then separately transformed into strains CRM32 and ZRM32, respectively, resulting in strains CRM32-p and ZRM32-p (Supplementary Table 3). These two strains now each contain a chromosomal copy of the *cueR/zntR-mEos3.2* gene and a corresponding exact copy in an inducible plasmid.

We further made C-terminally FLAG-tagged *zntR-mEos3.2*, *cueR-mEos3.2, and mEos3.2* genes (i.e., *zntR-mEos3.2-FLAG*, *cueR-mEos3.2-FLAG*, and *mEos3.2-FLAG* respectively) for detecting $ZntR^{mE}$ and $CueR^{mE}$ expression levels and possible cleavage products in Western blot.

The *zntR-mEos3.2-FLAG* gene fragment was made by using primers EcoRI-ZntR-f and PstI-mEos3.2-flag-r (Supplementary Table 1) with the genomic extract of strain ZRM32 as template. The fragment was digested with EcoRI-HF and PstI-HF and then inserted into a similarly digested pBAD24, giving plasmid pBZntR-mEos3.2-FLAG, which was then introduced into strain JW3254-5 (CGSC#: 10455), whose chromosomal *zntR* was knocked out (Supplementary Table 3), giving strain DZR-pBZRM32FLAG (Supplementary Table 3).

The *cueR-mEos3.2-FLAG* gene fragment was made by using primers EcoRI-CueR-f and PstI-mEos3.2-flag-r (Supplementary Table 1) with the genomic extract of strain CRM32 as template. The fragment was digested with EcoRI-HF and PstI-HF and then inserted into a similarly digested pBAD24, giving plasmid pBCueR-mEos3.2-FLAG, which was then introduced into strain JW0476-1 (CGSC#: 8628), whose chromosomal *cueR* was knocked out (Supplementary Table 3), giving strain DCR-pBCRM32FLAG (Supplementary Table 3).

The *mEos3.2-FLAG* gene fragment was made by using primers EcoRI-mEos3.2-f and PstI-mEos3.2-flag-r (Table S1) and template genomic extract of strain ZRM32 containing the *mEos3.2* gene. The fragment was digested with EcoRI-HF and PstI-HF and then inserted into a similarly digested pBAD24, giving plasmid pBmEos3.2-FLAG, which was then introduced into strain JW0476-1, whose chromosomal *cueR* was knocked out (Supplementary Table 3), giving strain DCR-pBM32FLAG (Supplementary Table 3).

2.3 Making the apo mutants $CueR^{mE}_{apo}$ and $ZntR^{mE}_{apo}$ in pBAD24

To make CueR and ZntR permanently apo and constitutively repressors, we mutated one of the metal binding cysteines to serine using site-directed mutagenesis protocol (QuikChange, Stratagene). For CueR, this mutation is C112S[6], and for ZntR, the mutation is C115S[7].

To make the pBAD24 plasmid expressing $CueR^{mE}_{apo}$, primers CueRC112S-f and CueRC112S-r were used together with pBCueR-mEos3.2 plasmid as the template (Supplementary Table 1 and Supplementary Table 2). After PCR amplification using AccuprimePfx DNA Polymerase, Dpn1 (NEB, R0176S) was added to the mixture to digest the methylated, nonmutated parental plasmid, leaving only the mutated plasmid pBCR(C112S)-mEos3.2 (Supplementary Table 2). The digestion reaction was allowed to proceed

for 4 h at 37°C. The plasmid pBCR(C112S)-mEos3.2 was then transformed into E. cloni 10G chemically competent cells for propagation and miniprep.

Similarly, to make the pBAD24 plasmid expressing $ZntR_{apo}^{mE}$, primers ZntRC115S-f and ZntRC115S-r were used together with pBZntR-mEos3.2 as the template (Supplementary Table 1 and Supplementary Table 2). PCR reaction gave the mutant plasmid pBZR(C115S)-mEos3.2 (Supplementary Table 2), which was transformed into E. cloni 10G chemically competent cells for propagation and miniprep.

The mutations were subsequently confirmed by DNA sequencing. pBCR(C112S)-mEos3.2 and pBZR(C115S)-mEos3.2 were then transformed into strains JW0476-1 (a Δ*cueR* strain) and JW3254-5 (a Δ*zntR* strain), respectively, resulting in strains DCR-pACRM32 and DZR-pAZRM32 (Supplementary Table 3).

2.4 *Making promoter knockouts, ΔP_{cueO} and ΔP_{zntA}, in the E. coli chromosome*

We used λ-Red homologous recombination to knock out a known DNA recognition site of CueR in Δ*cueR* strain (JW0476-1) or of ZntR in Δ*zntR* strain (JW3254-5), into which we later introduced a pBAD24 plasmid containing *cueR-mEos3.2* or *zntR-mEos3.2* genes, respectively.

To make the promoter knockouts, the plasmid pKD46, which contains the ampicillin resistance gene and three λ-phage enzyme genes (*exo*, β, γ) that work together during DNA homologous recombination, was first introduced via electroporation into the Δ*cueR* strain JW0476-1 and Δ*zntR* strain JW3254-5, resulting in strains JW0476-1λ and JW3254-5λ (Supplementary Table 3). Next, electrocompetent cells of these strains expressing the recombinase enzymes from pKD46 were prepared similarly as in Section 2.1.2 above. To knockout the *cueO* promoter (i.e., P_{cueO}), the linear DNA insert targeting the *cueO* promoter region in the chromosome was made using primers H1pCueO-f and H2pCueO-r together with a template having a chloramphenicol resistance gene cassette obtained from the plasmid pKD3; for the *zntA* promoter knockout, the corresponding primers used were H1pZntA-f and H2pZntA-r. The linear DNA insert with homology regions flanking the *cueO* (or *zntA*) promoter was introduced via electroporation into the recombinogenic strain JW0476-1λ (or strain JW3254-5λ for making ΔP_{zntA} construct). The cells were recovered in 1 mL SOC medium, incubated at 37°C and shaking at 250 rpm for 4 h, and finally plated onto LB-agar plate containing both chloramphenicol (10 μg/mL) and kanamycin (15 μg/mL), resulting in the strains JW0476-1DPCueO and JW3254-5DPZntA (Supplementary Table 3). Deletions were further confirmed by colony PCR using primers pCueO-f and pCueO-r for ΔP_{cueO}, and pZntA-f and pZntA-r for ΔP_{zntA} (Supplementary Table 1).

Plasmids pBCueR-mEos3.2 and pBZntR-mEos3.2 were transformed into strains JW0476-1DPCueO and JW3254-5DPZntA, respectively, yielding strains DCRPCO-pBCRM32 and DZRPZA-pBZRM32 (Supplementary Table 3).

2.5 *Making the plasmid expressing mEos3.2 alone (control)*

The *mEos3.2* gene was amplified from the *mEos3.2:cat* template using primers EcorI-mEos3.2-f and PstI-mEos3.2-r. The PCR product was digested with EcoRI-HF and PstI-HF and ligated into a similarly digested pBAD24 vector, yielding pBmEos3.2 (Supplementary Table 2), which was subsequently transformed into *E. coli* BW25113, yielding the strain BWPM32. Correct insertion was confirmed by colony PCR and DNA sequencing.

2.6 *Making the ΔcusRΔcueR double knockout and ΔcusR,cueR-mEos3.2 strains*

To make the double knockout strain *ΔcusRΔcueR* (JW0560-1DCR) and the *ΔcusR*,*cueR-mEos3.2* strain (DCSR-CRM32), we used the base *ΔcusR* strain JW0560-1, into which we first introducted the plasmid pKD46, resulting in the recombinogenic strain JW0560-1λ (Supplementary Table 3). Next, electrocompetent cells of these strains expressing the recombinase enzymes from pKD46 were prepared similarly as in Section 2.1.2.

To knockout *cueR*, the linear DNA insert targeting the *cueR* gene in the chromosome was made using primers H1dCueR-f and H2dCueR-r together with a template having a chloramphenicol resistance gene cassette obtained from the plasmid pKD3. To tag the chromosomal *cueR* with *mEos3.2,* the corresponding primers used were H1CueR-f and H2CueR-r to generate the linear DNA insert. The two linear DNAs were each introduced via electroporation into the recombinogenic strain JW0560-1λ. The cells were recovered in 1 mL SOC medium, incubated at 37°C and shaking at 250 rpm for 4 h, and finally plated onto LB-agar plate containing both chloramphenicol (10 µg/mL) and kanamycin (15 µg/mL), resulting in the strains JW0560-1DCR and DCSR-CRM32 (Supplementary Table 3). Deletions and mEos3.2 tagging were further confirmed by colony PCR using primers EcoRI-CueR-f and PstI-CueR-r for JW0560-1DCR, and EcoRI-CueR-f and SalI-mEos3.2-r for DCSR-CRM32 (Supplementary Table 1).

3. Functionality and intactness of mEos3.2-tagged proteins in *E. coli* cells

3.1 Ensemble fluorescence shows that the expressed mEos3.2-tagged proteins are fluorescent and photoconvertible

To test whether the expressed $CueR^{mE}$ and $ZntR^{mE}$ are actually fluorescent and photoconvertible inside cells, we performed fluorescence measurements of the cells grown in L-arabinose (inducer)-supplemented media. Strain CRM32-p, which has the *cueR-mEos3.2* gene encoded in both the chromosome and in pBAD24 plasmid, and strain ZRM32-p, which has the *zntR-mEos3.2* gene encoded in both the chromosome and pBAD24 plasmid, were cultured separately in 50 mL LB with chloramphenicol (25 µg/mL), ampicillin (100 µg/mL) and 10 mM L-arabinose in 37°C, shaking at 250 rpm, for 20 h. The cells were centrifuged and the pellet was re-suspended in PBS buffer (pH 7.4) for fluorescence measurements (Agilent Eclipse fluorometer). The emission spectrum of the green fluorescent form of mEos3.2 was collected using 465 nm excitation; its excitation spectrum was obtained by monitoring the emission at 556 nm. To photoconvert the green fluorescent form of mEos3.2 to its red fluorescent form, the cells in a 1 cm cuvette were exposed to 405 nm laser (10.5 W/cm^2) for 1 hour, then the emission spectrum of the cells was obtained using 525 nm excitation [4,8]; the excitation spectrum was obtained by monitoring emission at 635 nm.

Supplementary Figure 3 shows the emission and excitation spectra of $CueR^{mE}$ and $ZntR^{mE}$ in the cells before and after photoconversion. The spectra closely match those expected for the green and red forms of mEos3.2. Therefore, the mEos3.2 component of the fusion gene, once expressed in the cell, is fluorescent and can be photoconverted.

3.2 Cell growth assays under metal stress show that mEos3.2-tagged proteins are functional

The functionalities of the mEos3.2-tagged CueR and ZntR were tested using growth assays. The copper tolerance of the chromosomally engineered *E. coli* strain CRM32, which contains $CueR^{mE}$, was compared with those of the Δ*cueR* strain JW0476-1 and the wild type strain BW25113 (Supplementary Table 3). The zinc tolerance of the chromosomally engineered strain ZRM32, which contains $ZntR^{mE}$, was compared with those of the Δ*zntR* strain JW3254-5 and the wild type BW25113 (Supplementary Table 3). First, an overnight culture was prepared by inoculating a single cell-colony into 6 mL LB with

appropriate antibiotics, then the cells were allowed to grow for 18 h at 37ºC with shaking at 250 rpm. Next, the overnight cell cultures were each cultured in 10 mL LB (1:1,000 dilution of sample overnight culture, no antibiotics) in the presence of varying concentrations of $CuSO_4$ (for CRM32, JW0476-1, JW0560-1DCR, DCSR-CRM32, JW0560-1 and wild-type strain) and $ZnSO_4$ (for ZRM32, JW3254-5, and wild-type strain), incubated at 37°C aerobically with shaking at 250 rpm for 21 h. The final OD_{600} for each cell culture was then measured.

Supplementary Figure 4a shows that the cells whose chromosomal CueR is tagged with mEos3.2 show comparable tolerance to increasing copper concentrations in the growth media to the wild type strain (1 to 2 mM [$CuSO_4$]). In the same copper concentration range, the corresponding Δ*cueR* strain show consistently poorer tolerance (although the difference is small, i.e., about 10-15% lower; see Supplementary Figure 4a inset), supporting that mEos3.2-tagged CueR is functional. When copper concentration is larger than 2 mM, all three strains show poor tolerance.

To increase the copper sensitivity, we further used a Δ*cusR* base strain for growth assays. The copper tolerance of the chromosomally engineered *E. coli* strain DCSR-CRM32, which contains $CueR^{mE}$ in a *ΔcusR* background, was compared with those of the Δ*cusR* strain JW0560-1 and the Δ*cusR*Δ*cueR* double knockout strain JW0560-1DCR (Supplementary Table 3). *cusR* is another regulator in *E. coli* that controls the copper efflux pump *cusCBA*; knocking out *cusR* was used previously to incease copper sensititivy for growth assays[9]. Supplementary Figure 4b shows that the strain containing $CueR^{mE}$ and Δ*cusR* shows comparable tolerance to increasing copper concentration up to 3 mM to the Δ*cusR* base strain, while the Δ*cusR*Δ*cueR* double knockout strain is significantly poorer. These results further support that the mEos3.2-tagged CueR is functional.

For mEos3.2-tagged ZntR, Supplementary Figure 4c shows that the strain whose chromosomal ZntR is tagged with mEos3.2 shows comparable tolerance to increasing Zinc concentrations in the growth media to the wild type strain, whereas the corresponding Δ*zntR* strain shows significantly poorer tolerance, supporting that mEos3.2-tagged ZntR is functional.

3.3 *Protein gel analyses of cell lysates show that $CueR^{mE}$ stays intact in the cell, whereas $ZntR^{mE}$ is partially degraded; the latter is consistent with literature but does not affect our measurements of its unbinding kinetics from chromosomal recognition sites in vivo*

To our knowledge, there is no report so far on the degradation of CueR in *E. coli* cells and whether fusion proteins of CueR produce cleaved fragments in the cell. On the other hand, ZntR is a substrate of both ClpXP and Lon proteases and was reported to have a half-life of ~30 min in *E. coli*[10]. Because of this susceptibility to proteolytic cleavage, we expected ZntR and thus mEos3.2-tagged ZntR to be significantly degraded *in vivo*. Therefore, we performed protein gel electrophoresis analysis to: (1) check, and quantify if any, cleavage products of $CueR^{mE}$ and $ZntR^{mE}$ in the cell, and (2) check whether degradation, if applicable, continues to occur during our imaging experiment.

3.3.1 Coomassie-Blue stained SDS-PAGE shows that $CueR^{mE}$ is predominantly intact as a fusion protein in the cell and there is likely little change in cell physiology under induction conditions

SDS-PAGE was performed to verify the expression of $CueR^{mE}$ and $ZntR^{mE}$ as intact fusion proteins in cells. Two LB cultures of strain CRM32-p (for $CueR^{mE}$) or DZR-pBZRM32FLAG (for $ZntR^{mE}$) (Supplementary Table 3) were prepared: one culture containing no L-arabinose (inducer of fusion protein expression), and the other culture containing 1 mM or 10 mM L-arabinose. Both cultures were grown in the presence of chloramphenicol (25 μg/mL), and ampicillin (100 μg/mL). The cells were allowed to

grow at 37°C with shaking at 250 rpm for 18 h. A cell sample (1 mL) of each culture was centrifuged down (1300g, 5 min). The cell pellets from the cultures were lysed with 2X SDS-PAGE Laemmli buffer, heat-denatured at 95°C for 5 min before being loaded onto 12% SDS PAGE gel.

As shown in Supplementary Figure 5a (5th column) and Supplementary Figure 5b (2nd and 3rd columns), $CueR^{mE}$ (42 kDa) is overexpressed under induction. No clear band at ~26 kDa (the MW for mEos3.2) is observed, suggesting no significant cleavage of the mEos3.2 tag, which is confirmed in Western Blot analysis (see later). In addition, SDS-PAGE was also done on a negative control strain, CRM32-pBAD, which carries the original pBAD24 plasmid without inserts (Supplementary Table 3), and as expected, the SDS-PAGE gel does not show a discernable band of $CueR^{mE}$ (Supplementary Figure 5a, 3rd column).

Moreover, the overall cell protein expression profile of the induced CRM32-pBAD did not show any difference from that of the uninduced CRM32-pBAD sample (Supplementary Figure 5a, 3rd vs. 2nd column), whereas a marked differences can be observed only for the induced CRM32-p sample (Supplementary Figure 5a, 5th column vs. columns 2-4); therefore, the expression profile differences for the induced CRM32-p strain are predorminantly due to $CueR^{mE}$ overexpression and there is likely little change to the overall cell physiology under induction. In addition, inducing $CueR^{mE}$ expression at higher L-arabinose concentration also gives higher expression level of the protein (Supplementary Figure 5b), as expected.

We also performed similar SDS-PAGE analysis on the strain ZRM32-p (Supplementary Table 3) expressing $ZntR^{mE}$, but the expression level of this fusion protein and the changes to the overall cellular protein expression profile upon induction were not detectable using Coomassie Blue staining (Supplementary Figure 5c); we resorted to Western blotting, which is described in the next section.

On the other hand, regardless of being induced or not (Supplementary Figure 5c 3rd vs. 2nd column, and 6th vs. 4th column) and regardless whether the strain contains the $ZntR^{mE}$-expressing plasmid or the original plasmid without the $ZntR^{mE}$ insert (Supplementary Figure 5c, 6th vs. 3rd column and 4th vs. 2nd column), the SDS-PAGE protein expression profiles are similar, suggesting that there was little change in the cell physiology under induction conditions.

3.3.2 Western blot shows that $CueR^{mE}$ is essentially intact, whereas $ZntR^{mE}$ is significantly degraded in the cell (consistent with known cellular degradation of ZntR [10])

We further used Western blot to detect mEos3.2-tagged CueR and ZntR in the cell. Here the fusion proteins were further tagged with the FLAG epitope (RPDYKDDDDK) at the C-terminal of mEos3.2, i.e., $CueR^{mE}$-FLAG and $ZntR^{mE}$-FLAG, and anti-FLAG antibody was used for immuno-blotting.

$CueR^{mE}$-FLAG in cells. The SDS-PAGE in Section 3.3.1 has shown that mEos3.2-tagged CueR is predominantly intact in the cell and there is no discernable cleavage product. Here we evaluate it using Western blot, a more sensitive method than Coomassie-blue stained SDS-PAGE. We further evaluate the possible expression-level and time dependence of the intactness of mEos3.2-tagged CueR in the cell.

Procedures. Strain DCR-pBCRM32FLAG, which expresses $CueR^{mE}$-FLAG (Supplementary Table 3), was cultured in M9 medium without antibiotics at 30°C until OD_{600} = 0.3, then 1 mM L-arabinose (final solution concentration) was added and grown at 30°C for 30 min, or 20 h to have higher expression, with shaking at 200 rpm, in the same way as in the sample preparation for single-molecule imaging experiments (Section 4.1). Induction was then stopped by washing the cells in M9 medium (supplemented with 8% amino acids and 4% vitamins) containing 0.4% glucose.

From the cell culture induced for 30 min, the samples were immediately lysed after induction was stopped (i.e., t = 0 h, Supplementary Figure 6a). From the cell culture induced for 20 h, two sets of cell samples were prepared from the growth culture after stopping the induction. In one set indicated as t = 0 h in Supplementary Figure 6b, 1 mL of cell culture was centrifuged (1,300 g, 5 min) right after induction was stopped, and the pellet was immediately lysed with 100 μL of 2× SDS lysis buffer. In the other designated as t = 3 h in Supplementary Figure 6b, the cells were allowed to sit at room temperature (~22°C) for 3 h in M9 medium before centrifugation and lysis; this 3 hour period covers the overall period our imaging experiments were carried out.

The lysed samples were heat-denatured at 95°C for 5 min, and then were run in SDS PAGE, together with Amersham ECL Plex Fluorescent Rainbow protein molecular weight markers (GE Healthcare Life Sciences, Product code: RPN850E) in 1× MES buffer (pH = 7.3). Electrophoretic transfer of proteins from the SDS PAGE gel onto the Hybond-LFP PVDF membrane (GE Healthcare Life Sciences, Product code: 10600102) was performed for 70 min, 400 mA, 100 V (Transfer buffer: 25 mM Tris, 192 mM glycine, 20% methanol). The membrane was blocked with 4% Amersham ECL Prime blocking reagent (GE Healthcare Life Sciences, Product code: RPN418) in PBS-T (0.1% Tween-20, cat. #: P9416, Sigma-Aldrich) wash buffer, shaking at 25°C for 1 h. After blocking, the membrane was washed with PBS-T twice for 5 min each time. The membrane was incubated in a PBS-T solution containing mouse-derived DyLight549-conjugated FLAG-antibody (1:2,500 dilution, Rockland Immunochemical; cat. #: RL200-342-383) or with rabbit-derived anti-FLAG primary antibody (1:10,000 dilution, Rockland Immunochemical, cat. #: RL600-401-383S) for 2-4 h at 25°C, shaking at 190 rpm. The membrane was rinsed 4 x 5mins in PBS-T, then 3 x 5 mins in 10x PBS (pH = 7.4). For samples with lower protein expression that required a secondary antibody for detection, the goat-derived Horseradish Peroxidase-conjugated Fab fragment anti-rabbit antibody (1:5,000 dilution, Rockland Immunochemical; cat. #: RL811-1302) was used after primary antibody incubation/washing and probed with Pierce ECL 2 Western Blotting substrate (Fisher Scientific, cat. #: PI80196) as described by the manufacturer. The DyLight549 signal or peroxidase activity was detected using a fluorescent imager (Bio-Rad ChemiDoc MP Imaging System).

Results. For the 30 min induction and delay time t = 0 h sample, only one dominant band at MW ~42 KDa was observed (Supplementary Figure 6a), consistent with the expected MW of 42.2 kDa, indicating that CueR^{mE}-FLAG is essentially intact in the cell. At higher expression (i.e., 20 h induction and t = 0 h sample), the same dominant band was observed (Supplementary Figure 6b). On a closer look at the 20 h induction and t = 0 h sample, the gel also showed 3 additional ***barely visible*** bands at ~36 kDa, 29 kDa, and 26 kDa (the bands are almost invisible to human eyes on the image but can be seen by drawing line profiles across the image); the molecular weights here were determined using the calibration curve based on the molecular weight markers (Supplementary Figure 6e). Since the negative control (strain carrying pBAD24 without any insert and induced with L-arabinose) showed no detectable nonspecific bands (data not shown), these three bands are likely cleavage fragments that possibly contain the fluorescent mEos3.2 protein (the expected MW of the mEos3.2-FLAG part is 27 kDa). However, based on the relative intensities of the Western blot bands, these smaller fragments amount to < 8% of total detected protein on the Western blot, which is comparable to our measurement errors. *Therefore, CueR^{mE} is predominantly intact in the cell, and any possible cleavage fragments are insignificant for our measurements. And the intactness of CueR^{mE} is largely independent of its expression level.*

Moreover, the same results were observed for the t = 3 h sample, indicating that CueR^{mE}-FLAG has no time-dependent degradation within 3 hours of stopping induction. Therefore, *the protein concentration in the cell was at a steady state during the time when our imaging experiments were carried out*.

ZntR^{mE}-FLAG in cells. ZntR was reported to be a substrate of both ClpXP and Lon proteases and has a half-life of ~30 min in *E. coli* cell [10]. Because of this susceptibility to proteolytic cleavage, we expected ZntR and thus mEos3.2-tagged ZntR to be significantly degraded *in vivo*. To check and confirm the extent of this cleavage, we performed Western blot on cells expressing ZntR^{mE}-FLAG.

Procedure. Strain DZR-pBZRM32FLAG, which expresses ZntR^{mE}-FLAG, was cultured in M9 with 30 min induction as we did for the samples used for microscopy imaging, or in LB with 20 h induction to have higher expression for easier detection. Induction was stopped by washing the cells in M9 medium (supplemented with 8% amino acids and 4% vitamins) containing 0.4% glucose. The sample was immediately lysed after induction was stopped *(t* = 0 h*)* for gel analysis.

Results. For the sample grown in M9 (Supplementary Figure 6c), two major bands are observed at ~43 kDa and ~41 kDa with relative intensities of 1:3 (MW estimated using a MW calibration curve as done in Supplementary Figure 6e). The expected MW of ZntR^{mE}-FLAG is 43.2 kDa. Therefore, the band at ~43 kDa is assigned as the intact fusion protein, and the ~41 kDa band, based on its molecular weight, differs only in about a few to tens of amino acids from the intact ZntR^{mE}-FLAG, and the corresponding protein could still be a functional protein depending on the exact cleavage (see Section 17.2 on related discussions). Alternatively, as MW from comparing with markers is only approximate, the difference between the two bands could be small and both bands could be functional proteins. There is also a *barely visible* band at ~26 kDa, which is likely the cleaved mEos3.2 but amounts to <10% of the total detected protein on the gel image. *Taking into account that the ~41 kDa band may or may not be a functional protein, ~10 to ~70% ZntR^{mE}-FLAG is degraded in the cell.*

For the sample grown in LB with longer induction (Supplementary Figure 6d), the Western blot clearly showed all three bands at ~43 kDa, ~41 kDa, and ~26 kDa. Their relative intensities are ~35%, 33%, and 32%, respectively. Again, *taking into account that the ~41 kDa could still be a functional protein, ~32% to ~65% of ZntR^{mE}-FLAG is degraded in the cell.*

The cleavage of ZntR^{mE}-FLAG in the cell is consistent with the previous report that ZntR is prone to degradation in the cell and it has a cellular half-life of ~30 min [10]. In our single-molecule imaging experiments, *each* cell is imaged for a period of ~30 min. Based on ZntR's half-life, half of the mEos3.2-tagged ZntR molecules would be degraded during the imaging time, causing a time-dependent decrease of ZntR^{mE} concentration in the cell. Our measurement thus reflects a time-averaged protein unbinding kinetics during this period, during which the ZntR^{mE} concentration is changing.

3.3.3 Ensemble cell fluorescence measurements corroborate that CueR^{mE} stays intact in the cell and that ZntR^{mE}'s degradation is mostly confined to the ZntR part of the fusion protein

To probe potential protein degradation, we also measured the green fluorescence signal of mEos3.2 on the cells that express CueR^{mE} or ZntR^{mE} or, as a control, mEos3.2 (Supplementary Figure 7). Since the fluorescence signal is from mEos3.2, this measurement directly reports the status of the mEos3.2 part of the fusion protein.

CueR^{mE} in cells. After stopping the induction, the green fluorescence of the mEos3.2 from the cells decreases exponentially at a rate of ~0.84 ± 0.16 h^{-1} (Supplementary Figure 7a). Since Western blot analysis has already shown that CueR^{mE} stays intact in the cell (Supplementary Figure 6a and b), we attributed this fluorescence decay to the photobleaching of mEos3.2 from the excitation light. Consistently, when we performed the control fluorescence measurements on cells expressing the free mEos3.2, the cells here also showed the similar fluorescence decay over time, with the same decay rate of

0.81 ± 0.09 h^{-1} (Supplementary Figure 7c). *Therefore, the fluorescence measurements here further corroborate that CueR^{mE} stays intact in the cell over a period of hours after the stop of induction*.

ZntR^{mE} in cells. The cells expressing ZntR^{mE} also showed a decay of fluorescence signal after stopping the induction (Supplementary Figure 7b). Strikingly, the fluorescence decay rate here is only 0.70 ± 0.15 h^{-1}, the same rate within error as that of mEos3.2 photobleaching, even though we know from Western blot that ZntR^{mE} is degraded significantly in the cell (Supplementary Figure 6c and d). *This indicates that the mEos3.2 tag, once cleaved from ZntR, still stays functional (i.e., being a fluorescent protein), and the degradation of ZntR^{mE} is mainly limited to the ZntR part of the fusion protein*. This is also consistent that the two smaller fragments detected in Western blot have molecular weights greater or equal to that of mEos3.2 (Supplementary Figure 6c and d) and consistent with that ZntR's cellular half-life is ~30 min [10] and that mEos3.2 itself is stable over our fluorescence measurement time.

4. Microscopy cell sample preparation, cell viability under imaging, and relation among the cell cycle, cell size and the copy number of chromosome

4.1 Culturing cells for live cell imaging, and imaging sample preparation

E coli cells from a single colony were grown overnight (18 hours) in 6 mL LB medium containing the appropriate antibiotics. A sample from this overnight culture was diluted 1:100 in M9 medium supplemented with 8% MEM amino acids (GIBCO, cat. #: 11130051) and 4% MEM vitamins (GIBCO, cat. #: 11120052). The M9 medium used in the culture was prepared by mixing 200 mL of 5x M9 salts (0.1M Na_2HPO_4 (Mallinckrodt, Item code: 7917), 110 mM KH_2PO_4 (Mallinckrodt, Item code: 7100), 42.8 mM NaCl (Macron, 7581-12), 93.5 mM NH_4Cl (Mallinckrodt, Item code: 3384), all in 1 L sterilized nanopure H_2O), 2 mL of 1 M $MgSO_4$ (Fisher Scientific, M63-500), 20 mL of 20% glycerol, and 100 µL of 1 M $CaCl_2$ (Mallinckrodt, Item code: 4160), final volume adjusted to 1 L. When appropriate, the metals (e.g., Cu and Zn) in the M9 medium were removed by treating with Chelex 100 (Bio-rad Laboratories, cat. #: 1421253EDU) as described by the manufacturer, then filtered using Steriflip-GV membranes (0.22 µm, cat. #: SE1M179M6); we refer to this as the metal-depleted medium. The cells were incubated in 37°C until OD_{600} reaches 0.3. To induce the expression of genes encoded in pBAD24, L-arabinose (1 mM final concentration) was added to the cell culture. Induction times used in the experiments to vary intracellular protein concentration ranged from 5-30 min. The solution was pelleted by centrifugation at 1,300 g for 5 minutes, and then washed twice with M9 medium containing 0.4% glucose as a carbon source instead of glycerol at 1,300 g for 5 min each time (for experiments that involve metal stress, a final Cu^{2+} or Zn^{2+} concentration of 100 µM was added in this washing step and incubated for 1 h in the presence of the metal before imaging). Glucose is a catabolite repressor, inhibiting the expression of the fusion protein encoded in the pBAD24 plasmid under the control of the arabinose operon promoter. The final cell pellet to be used for imaging in the assembled sample cell below was re-suspended in 10 µL M9 medium containing 0.4% glucose, MEM amino acids (8%) and MEM vitamins (4%) (and Cu^{2+} or Zn^{2+} if applicable).

To prepare the imaging sample (Supplementary Figure 8), 30 µL of 100 nm gold nanoparticles (Ted Pella, Inc., Cat.#: 15708-9 **)** in 1:1 water-ethanol solution was first drop-casted onto a clean coverslip and allowed to dry at room temperature (~20 mins). These gold nanoparticles were used as position markers for drift correction. To serve as a gel pad for surface immobilization of *E. coli* cells, 20 µL of 3% agarose in M9 medium (supplemented with 0.4% glucose, 8% amino acids, and 4% vitamins) were placed onto a glass slide with parafilm spacers secured along the sides of the slide. Another glass slide was immediately pressed against the liquid agarose until it solidified to become a small gel pad. Double-sided tape was then used as a spacer in replacement of the parafilm lining the sides of the agarose gel pad. The cell sample (0.5µL) was added on top of the gel pad and then the coverslip with gold nanoparticles was

pressed against the gel pad, spreading and immobilizing the cells on the agarose gel. The coverslip edges were sealed with epoxy to prevent gel drying.

4.2 The E. coli cells are viable under our imaging conditions

To test if the *E. coli* cells were viable under our imaging conditions, we monitored the cell growth and division on the microscope at 20°C using cells immobilized on the agarose gel prepared with M9 medium containing glucose, amino acid, and vitamin (the typical condition during our imaging experiments, Section 5.2 and 5.3). The top row of Supplementary Figure 9 shows *E. coli* cells (the strain that expresses $\mathrm{CueR}^{\mathrm{mE}}_{\mathrm{apo}}$) growth and division without laser exposure, while the bottom row represents the same measurement but after performing laser photoconversion and imaging processes for single-molecule tracking. Similar growth rates in the two conditions suggest *that the cells were still viable after laser illumination and the laser exposure had insignificant influence on cell viability*. The cell doubling time here is about 250 min, consistent with the 235 min doubling time for *E. coli* in minimum medium at 21°C [11] and slower than the typical 30 min in LB medium or 120 min in minimum medium at 30°C [11,12], due to the lower temperature and minimal medium used here.

4.3 Hoescht dye staining to visualize chromosome organization

We also performed traditional fluorescence imaging to visualize the spatial spread of the chromosome by staining the chromosome with the Hoescht dye (the results are presented in Section 20.2). For this experiment, overnight cultures of *E. coli* cells were diluted 1,000× in M9, supplemented with 8% amino acids and vitamins (GIBCO). The cells were incubated in 37ºC, shaking at 250 rpm, until OD_{600} reached 0.3. (When applicable, chloramphenicol (100 µg/mL) is added to the cells to induce chromosome compaction and incubated for 30 min at 22ºC.) To this culture, Hoescht 33342 (BD Biosciences) solution was added to a final concentration of 12 µg/mL. The cells were incubated in 22ºC for 30 min before imaging. The cells were pelleted by centrifugation (1,300 g, 5 mins) then re-suspended in M9 medium (with amino acids and vitamins). The cell chamber set-up was assembled in the same way as Supplementary Figure 8. Imaging was done by exciting the DNA-bound Hoescht dye using a 405 nm laser (10-20 W/cm^2) and recording the fluorescence using an EMCCD camera. The filter used was AT460/50m (Chroma).

4.4 Relation between the cell cycle, cell size, and the copy number of chromosome in the cell

The cell cycle of slow-growing *E. coli* cells was extensively studied by flow cytometry and generally described to have B, C, and D three phases[13-17]. B phase is the period that newly formed daughter cells from division contain only one chromosome before they are ready (i.e., grown enough) to start chromosome replication. C phase is the period that the cell starts to replicate the chromosome and thus contains a partially replicated chromosome. D phase is the period that the cell has finished the replication and is ready to divide, and at this state, division septum appears and two chromosomes segregate into each daughter cell.

In our experiment, we cataloged the cells into non-dividing and dividing cells according to the absence and presence of the division septum. Therefore, the dividing cells we picked based on the appearance of the division septum were in this D phase, while the non-dividing ones (i.e., without a clear division septum) were either at the B or at the C phase.

To estimate the copy number of chromosome in each cell, we examined the integrated fluorescence intensity (corrected by the laser beam profile on the sample) from the Hoechst dye staining of the cell

chromosome; the integrated dye intensity should be proportional to the number of the chromosome inside a cell. Supplementary Figure 10a shows the distribution of the integrated Hoechst dye fluorescence intensity from dividing cells, using the cell strain expressing $\mathrm{CueR}_{\mathrm{apo}}^{\mathrm{mE}}$ as the example. The distribution is broad, but can be fitted by a single Gaussian-distributed population. The average integrated intensity here for a dividing cell is about 4.7 ± 1.8 a.u., which corresponds to 2 copies of the chromosome.

For non-dividing cells, the Hoechst dye intensity distribution is broader (Supplementary Figure 10b), reflecting the fact that these cells can contain one chromosome, or partially replicated chromosome for which the replication could be almost complete (i.e., almost two copies of chromosome). We Gaussian-resolved this distribution into two populations, in which one of the peak is shared in a global fit of the distribution of the dividing cells and represents the cell population containing 2 or close to 2 chromosomes. The resolved cell population with lower integrated intensity approximately represents those having 1 chromosome; their average fluorescence intensity is about 2.7 ± 1.5 a.u., which is about half of the value for the cells containing 2 chromosomes (4.7 ± 1.8), consistent with expectations. The relative areas of these two populations in (Supplementary Figure 10b) indicate that ~40% of the non-dividing cells have only one chromosome and ~60% of them have partially (or close to completely) replicated chromosome. This result agrees well with the flow cytometry results from Michelsen *et al.*[17] that *E. coli* K-12 cells under slow growth with a doubling time of ~240 min spend around 100, 100, and 40 min at the B, C, and D phases, respectively — this means that at any given time, ~50% of non-dividing cells contain only one chromosome.

5. Imaging setup for single-molecule tracking (SMT) via time-lapse stroboscopic imaging and for single-cell quantification of protein concentration (SCQPC) in live cells

5.1 Microscope configuration

The imaging was performed on an Olympus IX71 inverted microscope equipped with transmission optics and an electron-multiplying CCD camera (Andor Technology, DU-897E-CSO-#BV, pixel size 16×16 μm^2). A 60× TIRF oil immersion objective (Olympus PlanApo N 60× oil 1.45) together with a 1.6× magnification changer and a 1.2× C-mount adaptor (Spot Diagnostic Instruments, DD12BXC) collectively magnify the image 115.2×. The final image pixel size is 135.4 nm calibrated by using a high-precision Ronchi Ruling (Edmund Optics, 40 line-pairs/mm).

A schematic diagram of the optical setup is shown in Supplementary Figure 11. In the excitation path, an acousto-optic tunable filter, AOTF, (AA, AOTFnC-400.650-TN) is used to shutter 405 nm (CrystaLaser, DL405-100), 488 nm (CrystaLaser, DL488-050) and 561 nm lasers (Coherent, Sapphire 561-200CW). Three laser beams are spatially overlapped using dichroic filters (Chroma, T510lpxrxt and T425lpxr) and pass a quarter waveplate to change the polarizations to be circularly polarized. All laser lights were then expanded 4 times by an achromatic lens pair and focused (40 cm lens) at the back focal plane of the objective before being reflected toward the objective by a three-band dichroic filter (Chroma, Z408/488/561 rpc) inside the Olympus filter cube. The cells are then excited via epi-illumination with objective-collimated lasers whose beam size at the sample plane is 26 μm (FWHM). The epi-illumination is inclined approximately to be 60° from the optical axis of objective to ensure the illumination is through the cell and to decrease background from the medium above. In the detection path, the emission from mEos3.2 is passed through the three-band dichroic filter and the green (Chroma, ET525/50 M) or red band-pass filter (Semrock, FF01-617/73) before entering the EMCCD. A 200×200 pixel region of the EMCCD was used during data acquisition. The synchronization between camera and AOTF is through the Precision Control Unit (Andor Technology, ER_PCUT_101, PU-0614) and the imaging protocol is controlled via the Andor iQ 2.6 software.

5.2 Single-molecule tracking (SMT) via time-lapse stroboscopic imaging

SMT in live cells is achieved by combining a controlled photoconversion of mEos3.2 with the time-lapse stroboscopic imaging technique (Supplementary Figure 12a and b). Photoconversion of mEos3.2 is carried out with the 405 nm laser with a power density of 1-10 W/cm^2 to ensure less than one mEos3.2 per diffraction-limited area (or per cell) is converted on average. After photoconverting a single mEos3.2-tagged molecule, the camera-synchronized 561 nm laser is shuttered by the AOTF and creates pulse trains with short pulse width (T_{int}) and time lapse (T_{tl}) to probe and track the photoconverted molecule with power density of 21.7 kW/cm^2. The short duration of the 561 nm laser pulse is crucial for obtaining distinct fluorescent point spread function (PSF) from fast moving proteins in each imaging frame. Considering the typical diffusion constant of a cytosolic protein is around 3-10 μm^{2}s^{-1}, the minimum time resolution to generate a non-blurred PSF is about 1-5 ms estimated from the lateral resolution r of diffraction-limited resolution (i.e., r = 0.61λ/N.A.= 245 nm) and the two-dimensional diffusion mean square displacement (i.e., $\langle r^2 \rangle = 4Dt$). For example, for a molecule having a diffusion constant of 10 μm^{2}s^{-1}, it will only diffuse for about 200 nm in 1 ms, which is within the diffraction-limited PSF. The optimal time resolution (at which PSF shows the best signal to noise ratio without any smearing) for CueR^{mE} or ZntR^{mE} is tested out by examining the width of PSF at different T_{int}. We found 4 ms is sufficient for CueR^{mE} (84 kDa for a homodimer), which is similar to the reported T_{int} = 5 ms for tracking single RelA-Dendra2 (110 kDa) molecules in *E. coli* by English *et al.*[18]

Fitting the fluorescence PSF in each image with a two-dimensional Gaussian function gives the center location of the molecule in the image with tens of nanometer precision (down to a few nanometers at best, depending on the fluorescence photon counts). It is worth noting that this center location represents the average center localization of the molecule within T_{int}. By determining the center location of the molecule in each image frame across a time series, a position trajectory is thus obtained. Experimentally, the procedures described above is performed by an imaging cycle that includes a photoconversion of a CueR^{mE} or ZntR^{mE} molecule with 405 nm laser for 20 ms and subsequent 30 snapshots with camera-synchronized 561 nm laser with T_{int} = 4 ms and time lapse T_{tl} = 60 ms. The 60 ms was chosen after probing the dissociation kinetics with T_{tl} ranging from 20 to 200 ms to find the optimal resolution for probing protein unbinding kinetics across different protein concentrations — i.e., not too short to sample the residence times or too long so as to skip residence times. The number of imaging snapshots in each cycle was sufficient to eventually photobleach the molecule, before the next photoconversion/imaging cycle, which is repeated for 500 times for each living cell for later analysis.

5.3 Single-cell quantification of protein concentration (SCQPC)

In recent years, single-molecule fluorescence microscopy has been used to count mRNA or protein molecules in individual cells by dividing total fluorescence intensity of the whole cell by the single fluorophore intensity [19,20]. Even though this fluorescence based method is very powerful, two key aspects need to be handled carefully to obtain accurate copy numbers of proteins: the auto-fluorescence from the cell and the correct average fluorescence intensity of a single fluorophore. In our experiments, by using a photoconvertible fluorescent protein, we minimized the auto-florescence contribution by imaging the red fluorescence of mEos3.2. We photoconvert the fluorophore one at a time and perform SMT as described in Section 5.2 above. These photoconversion/imaging cycles simultaneously allow us to determine the average fluorescence intensity (i.e., $\langle I_{\mathrm{SMT}} \rangle$) of a single red mEos3.2 molecule in each image and the number of mEos3.2 proteins tracked (N_{SMT}). After the many photoconversion/imaging cycles, we photoconvert all the remaining green mEos3.2 proteins to their red forms and then measure the total red fluorescence intensity of the cell using the same imaging conditions. Since the average fluorescence

intensity $\langle I_{SMT} \rangle$ of a single mEos3.2 for each cell is determined from the same cell, it can be directly used to compare with the total red fluorescence intensity of the cell to calculate the copy number of the remaining proteins — note that the laser illumination intensity within the area of a cell (about 1×2.5 μm^2) is essentially homogeneous, even though the overall laser illumination profile is Gaussian-shaped over the entire illumination area of $\sim 26 \times 26$ μm^2.

Supplementary Figure 12 schematically shows the details of this assay. The first step is the same as described in the SMT section above (Supplementary Figure 12a and b, Section 5.2). In this SMT step, the information on the average intensity of single mEos3.2 ($\langle I_{SMT} \rangle$) is extracted from the number of proteins (N_{SMT}) inside the cell that were tracked and counted. Note that if an entire imaging cycle only has one single image containing fluorescence localization, this image cycle is discarded to minimize the contribution of spurious detection. The average count of molecules for a control cell without mEos3.2 gene is ~6 during the course of imaging, which is less than 10% of that for $CueR^{mE}$ and $ZntR^{mE}$ under basal expression from chromosome. In the second SCQPC step (Supplementary Figure 12a and c), the cells were then illuminated for 2 min with a 405 nm laser at 7 W/cm^2 to convert the rest of the proteins to red mEos3.2 followed by 561 nm laser imaging for 3000 frames at the same laser power density and laser exposure time as done in the SMT step. The second step can be repeated multiple times until all the proteins inside the cell are photoconverted. Under our experimental condition, two cycles will complete the photoconversion of all $CueR^{mE}$ and $ZntR^{mE}$ molecules. We then record the total red fluorescence intensity of the cell ($I_{SCQPC} = \Sigma I_i$) (Supplementary Figure 12c). Dividing the total fluorescence intensity by the average intensity of single fluorophore, $I_{SCQPC}/\langle I_{SMT} \rangle$, we obtain the copy number of the mEos3.2 proteins, N_{SCQPC}. Note that depending on the amount of remaining mEos3.2 molecules, the EM gain of the camera is adjusted accordingly to ensure the fluorescence signal is within the linear region of camera response. The fluorescence signal is then further corrected by the EM gain to obtain I_{SCQPC} and thus N_{SCQPC} for quantification. Considering the photoconversion efficiency of mEos3.2 ($PE_{mE} = 0.42$) [21,22] and the oligomeric state ($OS_{protein} = 2$ for homodimers CueR or ZntR), the total copy number of protein of interest in each cell can be calculated using Supplementary Equation 1:

$$N_{copy} = \frac{\left(N_{SMT} + N_{SCQPC}\right)}{PE_{mE} OS_{protein}} \qquad 1$$

To convert the single-cell protein copy number to the cellular protein concentration, we determined the cell volume from its transmission image. The cell boundary in the transmission image is fitted by the model of a cylinder with two hemispherical caps to get the quantitative information on the cell geometry as done by Itan *et al.* [23] (Supplementary Figure 13a). The width and length of the cell were defined as $2R$ and $2R+L$ and the volume of the cell is then calculated as $\pi R^2(4R/3+L)$ (Supplementary Figure 13a). Supplementary Figure 13b shows the histograms of cell geometries (width, length, aspect ratio (length/width), and cell volume of the *E. coli* cell strains under different growth conditions that were employed in our study. The average length, width, aspect ratio, and cell volume are about 2.5 µm, 1 µm, 2.5, and 2 fL, respectively.

6. Single-Molecule Imaging Data Analysis

6.1 Single-molecule localization and tracking

To obtain the localizations of individual mEos3.2-tagged proteins with nanometer precision, single-molecule fluorescence images were analyzed with the home written Matlab (R2012a, Math Works) program, iQPALM (image-based quantitative photo-activated localization microscopy). The major framework of iQPALM for image analysis is summarized in this section.

Identify the cell boundary and single-molecule fluorescence spots, and determine molecular localizations

The bright field transmission images were used to generate the cell boundary list where the cell boundary was mapped out by finding pixels around the cell that showed the largest pixel intensity contrast. The cell boundary list was then examined individually to exclude cell debris. Cells were then further classified into dividing and non-dividing ones according to whether a division septum is visible in the center region of the cell. Under our experimental condition, the cells were grown in M9 medium which results in a slow cell growing rate (doubling time ~250 min, Section 4.2).

For non-dividing cells, we further picked those with a length of ~ 2.7± 0.9 μm (Supplementary Figure 13b) to decrease the chance of selecting the cells that contain two complete chromosomes but do not yet show a visible division septum (Section 4.4). The cell boundary is then superimposed onto the corresponding fluorescence image and defines the region of interest (ROI) of each cell.

For the fluorescence images, each original image (e.g., Supplementary Figure 14a) was first convolved with a low-pass Gaussian kernel (13x13, σ = 1 pixel) to remove unreasonably small spots to generate a slightly smoothed image (e.g., Supplementary Figure 14b). This smoothed image is then applied by a boxcar kernel (15x15, pixel value = 1/225) to obtain the non-uniform background image (e.g., Supplementary Figure 14c). Subtracting Supplementary Figure 14c from Supplementary Figure 14b gives a final image (e.g., Supplementary Figure 14d) for spot localization as described by Henriques [24], Hess [25], and Weisshaar [26]. Pixels inside the ROI of the final image (e.g., Supplementary Figure 14d) were examined and those whose pixel intensities are above a user-defined threshold (typically the mean value plus 4 standard deviation of the whole image) were marked and stored in a candidate list. These candidates were further each fitted with a two-dimension (2D) Gaussian function using the original image corrected by the non-uniform background image (e.g., Supplementary Figure 14e generated by subtracting Supplementary Figure 14c from Supplementary Figure 14a) to obtain the information on the center position, intensity, spot size, and localization errors of each detected fluorescent molecule.

In detail, the centroid location of the candidate is extracted by fitting the filtered fluorescence image (a 13×13 image centered at candidate's coordinate) with a 2D Gaussian function:

$$I(x,y) = Aexp\left[-\frac{(x-x_0)^2}{2\sigma_x^2} - \frac{(y-y_0)^2}{2\sigma_y^2}\right] + B \qquad 2$$

Here $I(x,y)$ is the EMCCD fluorescence intensity counts of the candidate at position (x,y), A, B, (x_0,y_0), and (σ_x,σ_y) are the amplitude, background, centroid location, and standard deviation of the Gaussian function fit, respectively. The total EMCCD counts of the fitted spot (*cts*, the total volume under the fitted 2D Gaussian function) is then converted to the total number of fluorescence photons (N) via Supplementary Equation 3, provided by the camera supplier (Andor Technology).

$$N = \frac{(cts/g)\times(S/QE)\times 3.65}{E_{hv}} \qquad 3$$

Here g, S, and QE are the EM gain (unitless), sensitivity (electrons per count), and quantum yield (unitless) of the EMCCD camera in the spectral range of detected fluorescence respectively. The 3.65 is a physical constant for electron creation in silicon (eV per electron) and E_{hv} (in eV) is the energy of a single detected fluorescence photon where the center wavelength (584 nm, E_{hv}(584nm) = 2.12 eV) of mEos3.2 is used.

The localization error (Err_i, $i = x$ or y) of the centroid location was estimated according to [27,28]:

$$Err_i = \sqrt{\frac{\sigma_i^2}{N} + \frac{a^2}{12N} + \frac{8\pi\sigma_i^2 b^2}{a^2 N^2}} \quad 4$$

Here σ_i and N are the standard deviation of the 2D Gaussian fit and the total number of photons as described earlier; a is the pixel size; b is the standard deviation of the background obtained by subtracting the fitted 2D Gaussian function from the non-uniform background corrected fluorescence image (e.g., Supplementary Figure 14e).

Correct for sample drift and further filter the fitted fluorescence spots

The sample drift is acquired by registering the centroid drift of 100 nm Au nanoparticle markers relative to the first frame over the image stack. After fitting with 2D Gaussian functions, the centroids of candidates were corrected by the drift of Au nanoparticles in the same frame.

These drift-corrected candidates are then filtered based on σ_x and σ_y. If they are too small (i.e., too narrow for a reasonable single-molecule fluorescence PSF), they are rejected. If they are too big (i.e., too wide for a clean PSF image), they are also rejected. We used $80 < \sigma_i < 350$ nm ($i = x$ and y) as the thresholds based on the distribution of σ_x and σ_y of mEos3.2 molecules imaged in fixed *E. coli* cells as shown in Supplementary Figure 15. The average σ_x and σ_y is about 170 nm, which corresponds to a FWHM of ~ 401 nm for the fluorescence PSF, consistent with the (un-optimized) diffraction-limited resolution of our microscope. The candidates were then stored in the final localization list for SMT and subsequent analysis.

SMT trajectory

The final localizations are then grouped according to the imaging cycle (i.e., 30 frames) to generate the single-molecule tracking trajectories. Any imaging cycle that contains two or more spots in a single image frame is removed from diffusion and residence time analysis, as it is challenging to differentiate which is which in subsequent images for tracking. We note, however, that this situation occurs rarely (< 2%) under our photoconversion conditions, where we typically convert one or zero mEos3.2 per photoconversion/imaging cycle.

6.2 Thresholded microscopic residence times from the displacement trajectories of SMT

The SMT of a fluorescently tagged protein provides the information on protein locations and displacements over time. We can then generate the position trajectory (i.e., location vs. time) and the corresponding displacement trajectory (i.e., displacement r between consecutive image frames vs. time) as shown in Supplementary Figure 16 for CueR and ZntR. Once the protein binds to a recognition site on the chromosome, the movement of the protein then follows the chromosome movement, which has a very small effective diffusion constant (i.e., almost stationary) and thus on average has very small displacements. Therefore, we evaluate the residence time τ of CueR or ZntR on chromosome by defining the upper threshold of displacement r_0 (e.g., = 220 nm) to identify the protein that was bound to chromosomal recognition sites. The start of a τ is marked by a transition from high r to below r_0 and ends when r transitions to larger than r_0 (for example, τ_1 in Supplementary Figure 16a) or the fluorescence signal disappears due to photobleaching/blinking (for example, τ_2 in Supplementary Figure 16a). Section 14.1 and Section 8 below will justify the choice of r_0 value and show that our conclusions are independent of the threshold r_0 value.

7. Under chromosomal only expression or induced plasmid expression, there is always a huge cellular protein concentration heterogeneity, making it necessary to do single cell sorting into monodisperse populations based on SCQPC

To investigate protein-concentration-dependent processes in living *E. coli* cells, we adjust the cellular protein concentration by expressing it from an inducible plasmid (with or without the chromosomal copy) and changing the induction time. Ideally, by systematically varying the induction duration, one can directly probe the effect of protein concentration on the process of interest. Practically, this is difficult to achieve due to the huge protein concentration heterogeneity among *individual* cells. Supplementary Figure 17a shows the concentration distribution of ~100 cells that express $\mathrm{CueR}^{\mathrm{mE}}_{\mathrm{apo}}$ after 30-min induction from the same cell culture. Clearly, the protein concentrations of individual cells vary from tens of nM to a few μM, 2-3 orders of magnitude difference. The breadth of this distribution essentially covers the whole physiologically relevant concentration range and significantly obscures concentration-dependent processes if one compares populations of cells. Our single-cell protein quantitation method (i.e., SCQPC) overcomes this heterogeneity issue (Section 5.3): by quantifying the protein concentration of each cell, we can sort the individual cells accordingly into groups, each of which spans a narrow cellular protein concentration.

Even at expression from the chromosome only, cellular protein concentrations also vary significantly from cell to cell. Supplementary Figure 17b and c show the cellular $\mathrm{CueR}^{\mathrm{mE}}$ concentration distributions among cells grown without and with copper stress, respectively. Without copper stress, the average cellular protein $\mathrm{CueR}^{\mathrm{mE}}$ concentration is ~64 nM, similar to the reported number [29,30], but at the individual cell level, the concentration can range from minimally ~14 nM to maximally ~185 nM, a factor of 13 times in difference (Supplementary Figure 17b). Under copper stress that causes maximal *cueR* regulon induction, the average cellular concentration of CueR increases slightly to ~89 nM, but individual cells can have as low as ~17 nM or as high as ~240 nM, a factor of 14 times in difference (Supplementary Figure 17c) (Note why under copper stress the cellular concentration of CueR increases is not known; CueR does not regulate itself and what regulates *cueR* expression is unclear [29,31]). In both cases, the broad range again illustrates the protein expression heterogeneity problem among individual cells.

8. Regardless of the threshold r_0 value, $\langle\tau\rangle$ decreases with increasing cellular protein concentrations, and the trend persists after correcting for mEos3.2's photobleaching/blinking kinetics

To probe how the threshold r_0 might change the extracted microscopic residence times, we thresholded the displacement trajectories with r_0 = 170, 220, and 270 nm and determined the corresponding average τ for each cell across different protein concentrations. Supplementary Figure 18 shows the results for $\mathrm{CueR}^{\mathrm{mE}}_{\mathrm{apo}}$ as an example. We further average the $\langle\tau\rangle$'s over ~12,000 tracked protein molecules in ~450 cells with cellular protein concentration bin size ~200 nM. Regardless of r_0 value, the average residence time $\langle\tau\rangle$ shows the same trend: it is inversely proportional to the increasing cellular protein concentration. We thus fit the average residence time $\langle\tau\rangle$ with:

$$\langle\tau\rangle = \frac{1}{a[\mathrm{P}]_{\mathrm{cell}} + b} \qquad 5$$

where a and b are two parameters and $[\mathrm{P}]_{\mathrm{cell}}$ is the cellular protein concentration. Note Supplementary Equation 5 is equivalent to $\langle\tau\rangle^{-1} = a[\mathrm{P}]_{\mathrm{cell}} + b$. $\langle\tau\rangle^{-1}$ can be viewed as the overall rate that determines the average residence time $\langle\tau\rangle$, and it is determined by a $[\mathrm{P}]_{\mathrm{cell}}$-dependent term (i.e., $a[\mathrm{P}]_{\mathrm{cell}}$) and a protein-concentration-independent term (i.e., b). This form of Supplementary Equation 5 is related to the form of the apparent unbinding rate constant k_{-1}^{app} later in Supplementary Figure 29 and Section 14.

Moreover, in Section 14.1, we will show that the particular value of threshold r_0 can be included in the quantitative analysis of the distributions of τ, so that the r_0 value does not play a role in the extracted kinetic parameters of protein-DNA interactions.

This trend of $\langle\tau\rangle$ vs. $[P]_{cell}$ persists after further correcting the τ's for the contribution of mEos3.2 photobleaching/blinking kinetics whose rate constant k_{bl} was independently quantified from the length distribution of SMT trajectories (Section 10). Here we fit the distribution of residence time with a *single* exponential function, $y=\exp\left[-\left(\frac{1}{\tau^{pp}}+k_{bl}\frac{T_{int}}{T_{tl}}\right)t\right]$, which includes a rate constant $1/\tau^{pp}$ that accounts in general for kinetic processes that end the measured residence time except for the mEos3.2 photobleaching/blinking kinetics, for which the $k_{bl}\frac{T_{int}}{T_{tl}}$ term accounts for. We refer to this τ^{pp} as the photobleaching/blinking-corrected average residence time.

Supplementary Figure 19a and b show the concentration dependent average residence time (red dots) and corresponding τ^{pp} (blue markers) for $\mathrm{CueR}^{\mathrm{mE}}_{\mathrm{apo}}$ (or $\mathrm{ZntR}^{\mathrm{mE}}_{\mathrm{apo}}$) and $\mathrm{CueR}^{\mathrm{mE}}_{\mathrm{Cu}}$ (or $\mathrm{ZntR}^{\mathrm{mE}}_{\mathrm{Zn}}$), respectively. All of them show a clear trend that the average residence time decreases with increasing cellular protein concentrations.

9. Single-event level observation of the microscopic residence time τ allows extraction of kinetic processes faster than the experimental time resolution

In our measurements, the observable microscopic residence time τ is limited by our time resolution (here is 60 ms, from the time lapse T_{tl} = 60 ms), and a residence time that is shorter than 60 ms will not be measured. As the microscopic residence time effectively follows an exponential distribution (e.g., Supplementary Figure 20), the experimentally measured average τ, $\langle\tau\rangle$, thus represents an overestimate of the true average. However, as we measure the individual values of τ, the distribution of the measured τ allows us to extract the true average even though it is shorter than the experimental time resolution, as long as τ effectively follows an exponential distribution.

Supplementary Figure 20a shows a distribution of τ for $\mathrm{CueR}^{\mathrm{mE}}_{\mathrm{apo}}$. It follows effectively an exponential distribution. The straight average of the experimental τ's, $\langle\tau\rangle_{exp}$, is 76 ms. Fitting the distribution of τ with an exponential function gives the time constant $\tau_{exp,fit}$ of 59 ms, shorter than $\langle\tau\rangle_{exp}$ as expected. Assuming a single-step kinetic process for protein unbinding from DNA, the inverse of this time constant $\tau_{exp,fit}$ will give the rate constant for this unbinding step [32,33]. This $\tau_{exp,fit}$ is even shorter than the time resolution of 60 ms, reflecting that the distribution of the microscopic residence time τ allows for extraction of kinetics that are faster than the experimental time resolution. (Note 59 ms is not much shorter than 60 ms, just because our experimental system behaves this way.) This peculiar property stems from the property of single-exponental distribution of τ for (effective) single-setp kinetic processes: even though the distribution is partially measured due to time resolution limit, fitting the longer tail part of the distribution still gives the true time constant of the underlying exponential distribution.

Supplementary Figure 20b shows the simulation of the distribution of τ following an exponential function with a decay time constant $\tau_{0,sim}$ of 30 ms (plotted every 15 ms in blue open circles). We sample the simulated τ with the time resoluton of 60 ms (red open cricles), which is twice longer than the $\tau_{0,sim}$. We then perform the same analysis of the data sampled at 60 ms, as descibed in Supplementary Figure 20a. The straight average of the τ's ($\langle\tau\rangle_{sim}$) and single exponetial fitted $\tau_{fit,sim}$ is 69.4 and 30.2 ms,

respectively. Again the $\langle\tau\rangle_{sim}$ is longer than the time resolution of 60 ms, and the $\tau_{fit,sim}$ correctly extracted the true time constant of 30 ms in the simulation, which demonstrates the concept that single-event level observation of the microscopic residence time τ allows the extraction of kinetic processes faster than the experimental time resolution when τ effectively follows an exponential distribution.

10. Determination of photobleaching/blinking kinetics of mEos3.2 in *E. coli* cells

With stroboscopic SMT, we imaged photoconverted mEos3.2 at every single frame regardless of its being diffusing in the cytoplasm or bound to the chromosome until it photobleached (Supplementary Figure 21a). This allows us to directly examine the photoblinking and photobleaching kinetics of mEos3.2 quantitatively as described in previous literature [34-36]. In short, an emission intensity vs. time trajectory was generated by plotting out the fitted intensities of a tracked mEos3.2 in all images. The emission trace showed an on-off photoblinking behavior and eventually became permanently dark from photobleaching (Supplementary Figure 21b). The distribution of the on time, τ_{on}, reports the photobleaching/blinking rate constant, k_{bl}, which is the sum of blinking and bleaching rate constants. The distribution of the off time, τ_{off}, reports the recovery rate constants from the dark state, which contains two rate components k_{r1} and k_{r2}. These photophysics-related kinetic processes are schematically summarized in Supplementary Figure 21c, which is the same model as that used by Lee *et al.* [36] for mEos2, an earlier variant of mEos3.2 (i.e., mE).

Unlike the photophysics studies with continuous imaging scheme in the previous literature [34-36], our excitation laser pulse is merely on for T_{int} and stays off until the end of each T_{tl} in our time-lapse stroboscopic imaging. Therefore, the apparent photobleaching/blinking rate constant will equal to the intrinsic k_{bl} under continuous illumination condition corrected by a factor T_{int}/T_{tl} to account for the time-lapse imaging effect. The τ_{on} distribution can thus be fitted with Supplementary Equation 6 where C_{bl} is the normalization constant.

$$f_{bl}(t) = C_{bl}\exp\left(-k_{bl}\frac{T_{int}}{T_{tl}}t\right) \qquad 6$$

Supplementary Figure 21d shows the distribution of τ_{on} and the fitting to obtain k_{bl} of mEos3.2 under 561 nm laser illumination (power density of 21.7 kW/cm^2) with T_{int} = 4 ms and T_{tl} = 60 ms. We further determined k_{bl} at several different T_{tl} (i.e., 20, 60, 100, and 200 ms, Supplementary Figure 21e); the determined k_{bl} is independent of T_{tl}, as expected. The average k_{bl} is 257 ± 9 s^{-1}.

Lee et al reported that mEos2, a variant of mEos3.2, has photobleaching and blinking rate constants of 5 and 8 s^{-1} *in vitro*, respectively, at a lower laser power density (2 kW/cm^2)[36]. Similar values were reported by others[35]. Zhang *et al* did not provide quantitative rate constants but reported that mEos3.2 has a similar photobleaching rate to, but 1.8 times faster photoblinking than, mEos2[4]. Considering the 11 times higher laser power density we used, the expected k_{bl} for mEos3.2 based on Lee and Zhang would be (5+8×1.8)×11= 213 s^{-1}, within 17% of and thus consistent with our measured k_{bl} of 257 ± 9 s^{-1}.

Supplementary Figure 21f shows the corresponding distribution of τ_{off} and a double exponential fit to obtain k_{r1} and k_{r2}, the rate constants for fluorescence recovery from the dark state. The average k_{r1} and k_{r2} is 186 ± 22 and 31 ± 5 s^{-1}, respectively, over different time lapse T_{tl} (Supplementary Figure 21g). The recovery process is not relevant here for our study of protein residence time on chromosome in cells.

11. Determination of the number of diffusion states and their fractional populations

With displacement trajectories extracted from SMT data (e.g., Supplementary Figure 16), we further analyzed the displacement distributions. We found that *at least* three diffusion states were needed to

account for the SMT data of mEos3.2-tagged CueR or ZntR. In this section, we first introduce the basic diffusion model for analyzing the displacement distributions, determine and justify the minimum number of diffusion states needed to account for our data, as well as extract out the effective diffusion constants and fractional populations of the three diffusion states.

11.1 Probability density function (PDF) and cumulative distribution function (CDF) of displacement r per time lapse

In general, for a protein whose motions (fast or slow) follow Brownian diffusion effectively, let $P(\vec{r},t)\mathrm{d}\vec{r}$ denote the probability that a single fluorescently-tagged protein is detected within the region $[\vec{r},\vec{r}+\mathrm{d}\vec{r}]$ at the time *t*. $P(\vec{r},t)$ is the probability distribution function of the displacement vector $\vec{r}$, and it satisfies the following well-known 2-D Brownian diffusion equation [37]:

$$\frac{\partial P(\vec{r},t)}{\partial t} = D\nabla^2 P(\vec{r},t) \tag{7}$$

where *D* is the diffusion constant, and ∇^2 is the 2-D Laplacian operator. The solution to the above equation is:

$$P(\vec{r},t) = \frac{1}{4\pi Dt}\exp\left(-\frac{\vec{r}^2}{4Dt}\right) \tag{8a}$$

The probability distribution function of the scalar displacement *r*, PDF(*r*,*t*), in which all angular θ space in 2-D is included, satisfies $\mathrm{PDF}(r,t)\mathrm{d}r = \int_{\theta=0}^{2\pi} P(\vec{r},t)\mathrm{d}\vec{r}$. Subsituting $\mathrm{d}\vec{r} = r\mathrm{d}r\mathrm{d}\theta$ and $\vec{r}^2 = r^2$ gives:

$$\mathrm{PDF}(r,t) = \frac{r}{2Dt}\exp\left(-\frac{r^2}{4Dt}\right) \tag{8b}$$

In principle, if one measures the scalar displacement *r* over a time interval *t*, fitting the histogram of *r* with PDF(*r, t*) will give the corresponding diffusion constant *D*. In practice, this is complicated by the choice of bin size in generating the histogram of *r*, however. An optimal bin size is often challenging to define and the choice of bin size may affect the fitting results. To circumvent this issue, the cumulative distribution function (CDF) of scalar displacement *r* is used where the cumulative distribution can be explicitly determined. Integration of PDF(*r, t*) yields the cumulative distribution function:

$$\mathrm{CDF}\left(r_0,t\right) = \int_0^{r_0} \mathrm{PDF}(r,t)\mathrm{d}r = 1-\exp\left(-\frac{r_0^2}{4Dt}\right) \tag{9}$$

11.2 Number of effective diffusion states of CueR^{mE} and ZntR^{mE} in E. coli and their fractional populations

Our experimental CDF (and PDF) of the displacement *r* (e.g., Fig. 2a in the main text) indicates that more than one diffusion state are present for CueR^{mE} and ZntR^{mE} in living *E. coli* cells, even after considering the cell confinement effect on the CDF/PDF analysis of the diffusion behaviors of a molecule (see Section 15.2). To determine the number of diffusion states and the corresponding fractional populations, a linear combination of two or more PDFs or CDFs of the displacement *r* has been used in the literature to fit experimental SMT results and account for the multiple diffusion states of a protein in *E. coli* or mammalian cells [18,37-42]; this approach of using a linear combination of displacement PDFs/CDFs assumes a quasi-static system approximation, i.e., interconversion between the diffusion states is slower than the experimental time resolution (see justification regarding this approximation for the CueR^{mE} and ZntR^{mE} system in Section 18.4). We use a similar approach here, and further utilize the cellular protein concentration dependence of the diffusion behaviors to determine the minimal number of

diffusion states of CueR^{mE} and ZntR^{mE} in *E. coli* cells. Here we globally fit the CDF of displacement r over the entire accessible range of intracellular protein concentrations, in which the diffusion constants of respective diffusion states are shared but their fractional populations are allowed to vary. This protein-concentration-dependent CDF global fitting is useful to reveal all necessary diffusion states, as the protein concentration is expected to change the relative populations of the diffusion states but not their diffusion constants.

Supplementary Figure 22a shows exemplary concentration-dependent CDFs of r for CueR^{mE} (the CDFs are plotted against $r^2/4T_{tl}$ to factor out the time-lapse), where we sort the displacement trajectories of cells into five groups on the basis of each cell's protein concentration, and the five groups span a range of cellular protein concentrations from tens to hundreds of nM. Note that to obtain these experimental CDFs, only the first displacement of each single-molecule displacement trajectory was used to avoid the bias toward those molecules that have long trajectories. These CDFs clearly cannot be described by that of a single diffusion state as in Supplementary Equation 9, indicating that multiple diffusion states are present for CueR^{mE} and ZntR^{mE} moving inside cells.

To determine the minimum number of necessary diffusion states, we first fitted the CDFs assuming there are two diffusion states for CueR^{mE} and ZntR^{mE} in the cell. Accordingly, the CDFs across the different cellular protein concentrations are fitted with a linear combination of two terms, each of which follows Supplementary Equation 9 (Supplementary Figure 22a):

$$C_2(r) = A_1\left(1-\exp\left(-\frac{r^2}{4D_1T_{tl}}\right)\right) + A_2\left(1-\exp\left(-\frac{r^2}{4D_2T_{tl}}\right)\right) \qquad 10$$

Here A_1 and A_2 (= 1 − A_1) denote the relative amplitudes of the two diffusion states, and they correspond to their fractional populations in the cell. The two effective diffusion constants D_1 and D_2 are shared among all different cellular protein concentrations in the fitting. Supplementary Figure 22a shows the fitting results and the corresponding residuals across five different protein concentrations; the fitted D_1 and D_2 are 1.55 and 0.069 μm^2/s, respectively. It is clear that the two-diffusion-state model *cannot* fit the CDFs well: although the fits are reasonable for data at low cellular protein concentrations of 37-115 nM, the fits are clearly unsatisfactory for cellular concentrations of 592-789 nM. The difference between the fit and the data at larger cellular protein concentrations is mainly at the range toward larger $r^2/4T_{tl}$, i.e., larger displacement regions, which suggests that there is at least another diffusion state with a larger effective diffusion constant.

Therefore, we introduced a third diffusion state and fitted the CDFs using a three-state model:

$$C_3(r) = A_1\left(1-\exp\left(-\frac{r^2}{4D_1T_{tl}}\right)\right) + A_2\left(1-\exp\left(-\frac{r^2}{4D_2T_{tl}}\right)\right) + (1-A_1-A_2)\left(1-\exp\left(-\frac{r^2}{4D_3T_{tl}}\right)\right) \qquad 11$$

Here $1-A_1-A_2 = A_3$. The data are clearly satisfactorily fitted across all cellular protein concentrations (Supplementary Figure 22b), and the resulting three effective diffusion constants are D_1 = 4.2 ± 0.3, D_2 = 0.76 ± 0.04, and D_3 = 0.046 ± 0.015 μm^2/s. *It is worth noting that performing a global fit where the D's are shared across different cellular protein concentrations and A's are floated were critical in obtaining consistently-converged fitting result.*

The above analysis applies equally to the CDF analyses of all other CueR or ZntR variants across all different culture conditions. Three is always the minimal number of diffusion states to fit satisfactorily the CDFs across all cellular protein concentrations. The fitted D's and A's are summarized in Supplementary Table 4 and Supplementary Table 5.

12. Assignments and justifications of the three diffusion states of CueR or ZntR in live cells

We assigned the D_1, D_2, and D_3 states respectively to proteins freely diffusing (FD, D_{FD}) in the cytoplasm, nonspecifically bound to and moving on DNA (NB, D_{NB}), and specifically bound (SB, D_{SB}) to chromosomal recognition sites whose motions reflect the chromosome dynamics and uncertainties in molecular localization [18,44-48]. These assignments were made initially for the following reasons:

(1) These three states are expected to be present for CueR or ZntR in a bacterial cell: both CueR and ZntR can bind to DNA specifically to their respective recognition sequences [10,49-51]; CueR can bind DNA nonspecifically [50], and ZntR is expected to do so as well.
(2) The three effective D's are also consistent with reports [38,41,47,48] when the cell confinement effect [18] and the time-lapse effect of imaging [38] are both taken into account.
(3) When tracking CueR across different protein concentrations ranging from tens to hundreds of nM, the fractional populations A_{FD} and A_{NB} increase to 33% and 63%, whereas A_{SB} decreases to 4% as shown in Supplementary Figure 22c, consistent with that at higher protein concentrations, each regulator protein will spend more time freely diffusing or nonspecifically bound to DNA than specifically bound at the recognition sites. Similar results were observed for ZntR (Supplementary Table 5).

It is also important to note that both CueR and ZntR function as *stable* homodimers. The dissociation constants for their dimerization are not known, but our previous *in vitro* single-molecule studies on CueR have shown that down to nM range, its potential dissociation into monomers and the associated dimer-monomer equilibrium is insignificant for considering their interaction kinetics with DNA [50]. In the cell, the basal expressions of CueR and ZntR give their average cellular concentrations in the range of tens of nM (Section 7, and Supplementary Figure 17).

Besides the above three reasons, these assignments of the three states are further justified below:

12.1 Justifications for the assignment of the freely diffusing (FD) state (i.e., $D_1 \equiv D_{FD}$)

To support the assignment of the FD state of D_1, we use a cytosolic fluorescent protein, mEos3.2, to mimic the freely diffusing behavior of $CueR^{mE}$ and $ZntR^{mE}$ and perform the SMT and CDF analysis under the same T_{tl} of 60 ms. Fitting the CDF with one diffusion state shows a clear deviation from the Brownian motion (Supplementary Figure 23a, left). Two diffusion states thus are adopted to obtain satisfactory CDF fitting results where the fitted diffusion constants are 3.3 ± 0.2 and 0.51 ± 0.02 $\mu m^2/s$, with their fractional populations of 66% and 34%, respectively (Supplementary Figure 23a, right). The fast diffusion constant is the same within error bar as D_1 (3.7 ± 0.3 $\mu m^2/s$) extracted from CueR and ZntR, supporting its assignment of FD states.

The second, slow diffusion state (D_2) of mEos3.2 from fitting its CDF in Supplementary Figure 23a, right, is an artifact of cell confinement effect on the CDF analysis; this confinement effect is only significant for those with intrinsic diffusion constant $D > 1.88$ $\mu m^2/s$ (i.e., need more than one diffusion state model to fit the CDF), as shown by simulations (details of simulation, see Section 15.2).

We investigated the possible reason for the appearance of a second, minor and slower, artificial component in the CDF analysis of mEos3.2 diffusive motion using the diffusion simulation that contains only one fast diffusion state. We found that a contributor is the anisotropic shape of the cell, represented by its aspect ratio F (= cell-length/cell-width). Supplementary Figure 24 shows the dependence of the CDF of r (plotted against $r^2/4T_{tl}$; T_{tl} = 60 ms) from 1-diffusion-state simulations with cell aspect ratio being 2, 3, 4, and 5; the cell width was kept at 1.15 μm, and D_{input} = 11 $\mu m^2/s$, which is about the intrinsic

diffusion constant of free mEos3.2 in the bacterial cytoplasm [18]. At F =3 (close to the actual aspect ratio of ~2.5 of our BW25113 cell strain; Supplementary Figure 13), two diffusion components are needed to fit satisfactorily the CDF, even though the simulation only contained 1 diffusion state. With increasing aspect ratio, the second artificial component becomes more apparent. In general, the larger the aspect ratio, the larger (i.e., closer to the input) of the extracted effective diffusion constants, and the lower the amplitude of the major component, as shown in Supplementary Figure 24.

In our imaging experiments, the *E. coli* cells have a monodisperse distribution of aspect ratio centered at ~2.5 (Supplementary Figure 13). Therefore, for a CueR^{mE} or ZntR^{mE} molecule at the FD state with an intrinsic diffusion constant of about 11 $\mu m^2/s$, the CDF analysis will generate a second artificial slow component at ~1.1 $\mu m^2/s$, which is close to and thus contaminates the apparent diffusion constant of the NB state (D_2), as we will show in more detail later in Supplementary Figure 36 (Section 15.2). This artificial second component is, again, insignificant for states with intrinsic diffusion constants <1.88 $\mu m^2/s$ (i.e., apparent diffusion constants < 1.5 $\mu m^2/s$ at T_{tl} = 60 ms and cell aspect ratio ~ 2.5), for which a single component can fit its CDF satisfactorily.

In a confined geometry, the effective diffusion constant measured through time-lapse imaging is dependent on the value of time-lapse. To probe the time-lapse effect, we further performed SMT of free mEos3.2 with T_{tl} = 15 ms. Two diffusion states are still needed to fit the CDF (Supplementary Figure 23b), but their diffusion constants are now expectedly larger than those obtained at T_{tl} = 60 ms: 11.4 ± 0.3 and 0.70 ± 0.02 $\mu m^2 s^{-1}$ and fractional populations of 47% and 53%, respectively. The fast diffusion constant (11.4 $\mu m^2 s^{-1}$) at T_{tl} = 15 ms is similar to the literature reported value of freely diffusion mEos2 (a variant that differs from mEos3.2, i.e., mE, by 2 mutations [4]) estimated from the mean square displacement (MSD) analysis [18]. The slower one again is an artifact from the cell confinement effect on the CDF analysis. This again supports the assignment of the fast diffusion state D_1 at T_{tl} = 60 ms as being the freely diffusion state.

We also examine the diffusion behaviors of CueR^{mE} and ZntR^{mE} at different time-lapses T_{tl} from 30 to 400 ms. CDFs at different T_{tl} are globally fitted with shared fractional populations of three states while allowing the diffusion constants to float. The time-lapse dependent diffusion constants are summarized in Supplementary Figure 25. With increasing time-lapse, the three effective diffusion constants decrease, as expected.

It is well-known that the cytoplasm is a crowded soft environment and the diffusion is not directly proportional to T_{tl}.[52] This is indeed what we observed and thus we further fit the diffusion constants at different time-lapse for each state with

$$D = D_{\alpha} T_{tl}^{\alpha-1} \quad 12$$

where D_α and α characterize the amplitude and the degree of sub-diffusivity of motion [53]. With D_α and α extracted from CueR^{mE} or ZntR^{mE}, we can extrapolate D's to T_{tl} = 15 ms and get the diffusion constant D_1 for the FD state of 10.1 ± 3.1 $\mu m^2 s^{-1}$; this value is consistent with the diffusion constant D_1 of free mEos3.2 at T_{tl} = 15 ms described above (Supplementary Figure 23b) and again supports its assignment as the freely diffusing state (FD) of CueR^{mE} or ZntR^{mE}.

12.2 Justifications for the assignment of the specifically bound (SB) state (i.e., $D_3 \equiv D_{SB}$)

The assignment of D_3 to the CueR^{mE} and ZntR^{mE} specifically bound to chromosomal recognition sites was based on the following:

(1) The value (~0.04 μm^{2}s^{-1}; Supplementary Table 4) of D_3 (measured at time lapse T_{tl} = 60 ms) is consistent with the reported diffusion constant D = 0.075 μm^{2}s^{-1} with T_{tl} = 30 ms for DNA polymerase bound to DNA in *E. coli* by Uphoff *et al.* [41] Note for diffusion constants this small (i.e., D < 0.1 μm^{2}s^{-1}), its measured value has very little dependence on the time lapse in the imaging between T_{tl} = 30 ms and 60 ms (Supplementary Figure 25; note its y-axis is in log scale).

(2) We further perform the short-time movement analysis, as previously done by Javer *et al.,* who used it to determine the motion of *E. coli* chromosomal loci[44]. In this analysis, one analyzes selected segments of the SMT trajectories, where the displacements are below the threshold r_0, that is, the segments of the tracking trajectories that give rise to the residence time τ as in Supplementary Figure 16. These segments (and associated residence times) comprise proteins at specific recognition sites on the chromosome (i.e., specifically bound (SB) states), but also have contributions from proteins that are nonspecifically bound to DNA (NB) or freely diffusing (FD), both of which have finite possibility of having displacements smaller than the threshold r_0.

After thresholding to obtain them, these segments are further grouped according to their lengths (i.e., the length of their corresponding residence times in the units of image frame number N; N = 4 to 8). Mean square displacement is then calculated for each group as a function of time lapse as shown in Supplementary Figure 26a. The initial slope of MSD vs. time lapse, determined from the first five data points, is used to estimate the diffusion constant. With increasing N, i.e., with increasing length of the microscopic residence time, the contribution of the SB state to these segments increases, while those of the NB and FD states decreases. Consequently, the determined diffusion constant from the MSD-vs.-time-lapse should decrease with increasing N, and eventually flatten, as observed (Supplementary Figure 26b); the limiting value of the diffusion constant at large N should then be that for the SB state. This limiting value of diffusion constant is determined to be ~0.035 μm^{2}s^{-1} (Supplementary Figure 26b), in excellent agreement with the value of D_3 (~0.04 μm^{2}s^{-1}) extracted from the CDF analysis. This agreement again supports the assignment of the D_3 state as proteins specifically bound to chromosomal recognition sites.

(3) Moreover, we determined the CDF of displacement using various time-lapse T_{tl} and extracted the dependence of D_3 on the time-lapse (Supplementary Figure 25). Fitting the data with Supplementary Equation 12 gives α = 0.45 ± 0.12 , consistent with the reported α (= 0.39 ± 0.04) for bacterial chromosomal loci movements by Weber *et al.* [54] This agreement again supports that the D_3 state is the specifically bound state of protein at chromosomal recognition sites.

12.3 Justifications for the assignment of the nonspecifically bound (NB) state (i.e., $D_2 \equiv D_{NB}$)

The assignment of D_2 to the CueR^{mE} and ZntR^{mE} nonspecifically bound to DNA was based on the following:

(1) The value (~0.7 μm^{2}s^{-1}; Supplementary Table 4) of D_2 (measured at time lapse T_{tl} = 60 ms) is consistent with the reported effective diffusion constant D_{eff} (including the one-dimensional diffusion along DNA segments and 3D translocation among segments) of 0.4 ± 0.02 μm^{2}s^{-1} for nonspecifically bound transcription factor LacI on DNA in *E. coli* by Elf *et al.* [48] from MSD analysis.

(2) The major concern about the assignment of the NB state originates from that the CDF analysis of a single free-diffusing state with an intrinsic diffusion constant > 1.88 μm^{2}s^{-1} requires two diffusion exponential components to fit (Supplementary Figure 23 above) and therefore the NB state may be just an artifact of the slower component of the FD state. To clarify this issue, we further examined the fractional

populations of CueR or ZntR diffusion states across all concentrations and compared them with free mEos3.2 case. From the CDF analysis of mEos3.2 described above, at T_{tl} = 60 ms, the fractional populations of the fast (3.3 ± 0.2 $\mu m^2 s^{-1}$) and slow (0.51 ± 0.02 $\mu m^2 s^{-1}$) components are 66% and 34% respectively and are independent on the protein concentration. This means that for $CueR^{mE}$ and $ZntR^{mE}$, if the D_2 state is purely an artifact from the FD state, the fractional population of D_2 will always be lower than that of the D_1 state, which contradicts with our observation that A_{NB} is always larger than A_{FD} and increases with increasing cellular protein concentration (Supplementary Figure 22c and Supplementary Table 5). This suggests that even though the NB state may be contaminated by the free-diffusing D_1 state, it cannot be wholly an artifact from the CDF analysis of the FD state, which supports at least three diffusion states are present in our SMT data of CueR and ZntR in live *E. coli* cells.

Moreover, we evaluated the limiting case assuming that the observed D_2 state *were* to be entirely an artifact from the FD state (Section 14.6). It does not affect our conclusion that the unbinding kinetics of CueR or ZntR from recognition sites increases with increasing cellular protein concentrations in *E. coli* cells.

13. mEos3.2-tagged CueR and ZntR distribute over the cell homogeneously, except when binding to DNA, and they do not aggregate

This section addresses the potential concern that mEos3.2-tagged CueR or ZntR might exist in different subcellular locations, thus experiencing different microenvironments and leading to different effective diffusion constants. By examining the spatial distribution of $CueR^{mE}$ and $ZntR^{mE}$ in the cell and the cell morphology in transmission images, we alleviate the concern as described below.

13.1 Spatial distribution of localizations of tracked CueR or ZntR molecules is homogeneous throughout the cell

To examine whether the mEos3.2-tagged CueR or ZntR might localize in different subcellular locations or just homogeneously distribute over the cell, we first overlaid the *first* localizations of all trajectories of tracked molecules on the cell bright field transmission images. The top row of Supplementary Figure 27 shows such overlays using $CueR^{mE}_{apo}$ as the example. The spatial distribution of localizations suggests that $CueR^{mE}_{apo}$ can sample the entire cell volume. In these overlaid localizations, sometimes we observe some clusters of the localizations colored in green as shown in the top right two panels in Supplementary Figure 27, which comes from protein binding to DNA in the cell (i.e., residence sites on DNA). Using the r_0-thresholding of the displacement trajectories (e.g., Supplementary Figure 16), we removed these residence sites associated with $r < r_0$, which are dominated by proteins either specifically or nonspecifically bound to DNA. The rest of locations homogeneously distribute over the cell, indicating that there are no discernable subcellular spatial localization of the proteins. This conclusion holds for all CueR or ZntR variants we studied.

13.2 No aggregation or inclusion bodies are observed in cells expressing mEos3.2-tagged proteins

When inducing protein expression from a plasmid, one potential problem is the formation of inclusion bodies (i.e., insoluble aggregates of misfolded proteins) at high cellular protein concentrations. These inclusion bodies can often be seen from the transmission image, as reported in literatures [55-57]. Similar inclusion bodies are also observed in the transmission images by us for cells that overexpress CueR that was tagged with a cleaved mEos3.2 *fragment* (as done in bimolecular fluorescence complementation assay [58]), which is an aggregation-prone fusion protein (Supplementary Figure 28a). These inclusion

bodies appear as bright localized spots in the cell (marked by black arrows in Supplementary Figure 28a). In contrast, transmission images of the cells whose expression of $\mathrm{CueR}_{\mathrm{apo}}^{\mathrm{mE}}$ were induced for 30 min, the longest induction for our experiments, show a homogeneous morphology and no aggregation or inclusion bodies were observed (Supplementary Figure 28b). The same conclusion applies to cells that express other tagged CueR or ZntR variants studied here.

14. Kinetic model of regulator–DNA interactions and the derivations of the corresponding microscopic residence time distribution

Here we use a kinetic model for CueR or ZntR-DNA interactions in the cell to derive the equations to analyze the microscopic residence time τ thresholded from the displacement trajectories as in Supplementary Figure 16. This kinetic model includes three different states for a regulator in the cell (Supplementary Figure 29): freely diffusing (FD), nonspecifically bound to DNA (NB), and specifically bound to recognition sites on the chromosome (SB). These three states were resolved and assigned based on our experimental results and literature, as described in detail in Section 12 earlier. Among the three states, this model includes the following kinetic processes:

(1) A FD regulator can convert to a SB regulator reversibly via binding to specific recognition sites on the chromosome. The apparent rate constant for binding here is $k_1[\mathrm{D}]_{\mathrm{SB}}$, where k_1 is a rate constant and $[\mathrm{D}]_{\mathrm{SB}}$ is the *effective* concentration of vacant regulator specific recognition sites in the cell. The apparent unbinding rate constant k_{-1}^{app} ($k_{-1}^{\mathrm{app}} = k_{-1} + k_{\mathrm{f}}\left[\mathrm{P}\right]_{\mathrm{FD}}$) contains two terms: the spontaneous unbinding term (i.e., k_{-1}) and the facilitated unbinding term (k_{f}; $[\mathrm{P}]_{\mathrm{FD}}$ is the concentration of FD regulator).
(2) A FD regulator can convert to a NB regulator reversibly via binding nonspecifically to DNA. The apparent rate constant for binding here is $k_2[\mathrm{D}]_{\mathrm{NB}}$, where k_2 is a rate constant and $[\mathrm{D}]_{\mathrm{NB}}$ is the *effective* concentration of vacant nonspecific DNA binding sites in the cell. The unbinding rate constant here is k_{-2}.
(3) The direct interconversion for a regulator between the NB and SB states is assumed to be sufficiently slow to be negligible for the following reasons: (i) The rate constant for the direct transition for a regulator at the NB state to the SB state should be very small, because it corresponds to the process for a protein to go from a NB site directly to a SB site without going through a dissociated state. The number of nonspecific binding sites is very large and the specific recognition sites are merely at discrete locations. On average, when a regulator is at the NB site, a specific recognition site is far away, making this direct transition from NB to SB site low probability. (ii) From the population analysis of FD, NB, and SB states (Section 14.5), we also know that the rate constant for direct transition from SB to NB state is on the same order of magnitude (i.e., equally small) as that from NB to SB state. Therefore, the direct interconversion between NB and SB states is neglected as an approximation in the kinetic model.
(4) On top of all the kinetic processes between the three states, the photobleaching/blinking process, with the rate constant k_{bl} of mEos3.2, limits the observation time of each photoconverted protein.

14.1 Derivation of the distribution function of the r_0 thresholded microscopic residence time τ

In this section, we will derive the theoretical distribution of the microscopic residence time τ, which was obtained by thresholding the displacement r versus time trajectory at a threshold value r_0, as in Supplementary Figure 16. For a moving molecule with an effective diffusion constant D, its probability

density function PDF(r,t) of displacement r at time t is described by Supplementary Equation 8. Integrating PDF(r,t) to a certain radius r_0:

$$S(r_0,t)=\int_0^{r_0}\mathrm{d}r\mathrm{PDF}(r,t)=1-\exp\left(-\frac{{r_0}^2}{4Dt}\right) \qquad 13$$

$S(r_0,t)$ is the probability for a molecule that was initially at the origin to survive (i.e., stay) within a circle of radius r_0 during the time interval (0, t). In other words, $S(r_0,t)$ is the probability for the displacement of this molecule to be ≤ r_0 within the time interval (0, t). The function form of $S(r_0,t)$ is the same as the cumulative distribution function (CDF) of r (Supplementary Equation 9), which was used to extract the diffusion constant (D) and fractional population (A) of each state in CDF analysis (Supplementary Figure 22).

For a protein molecule at the SB state, its motion, which stems from the chromosomal diffusion in the cell, is slow (i.e., having a very small effective diffusion coefficient D) and its associated displacement r is small on average. Therefore, in the r-vs.-t trajectory (Supplementary Figure 16), by setting a displacement threshold r_0 of a reasonably large value (e.g., 220 nm), we can include all displacements belonging to a protein molecule that is at the SB state and consequently, extract its microscopic residence time τ at the SB state.

Experimentally, τ starts with a transition from large r to below r_0, and it ends either when r transitions to above r_0 or when the fluorescence signal disappears due to the photobleaching/photoblinking of the mEos3.2 tag. To connect the experimentally thresholded τ to the distribution of the microscopic residence time τ at the SB state, we thus need to correct the survival probability $S(r_0,t)$ so that it should include not only the slow diffusive motions of the chromosome but also the dissociation of the protein from the recognition site and the photobleaching/blinking kinetics of mEos3.2. Since single-step kinetics such as protein dissociation from DNA and photobleaching/photoblinking causes an exponential decay in survival probability of the starting state, the *corrected* survival probability for $r \le r_0$ is

$$S`(r_0,t)=S(r_0,t)\exp\left(-k_{\mathrm{eff}}t\right)=\left(1-\exp\left(-\frac{{r_0}^2}{4Dt}\right)\right)\exp\left(-k_{\mathrm{eff}}t\right) \qquad 14$$

where the exponential term containing k_{eff} corrects for the decrease of the survival probability by protein dissociation from the recognition site and by the photobleaching/blinking of mEos3.2. $k_{\mathrm{eff}} = k_{-1}^{\mathrm{app}} + k_{\mathrm{bl}}\frac{T_{\mathrm{int}}}{T_{\mathrm{tl}}}$, i.e., a sum of k_{-1}^{app}, the apparent unbinding rate constant from recognition sites, and $k_{\mathrm{bl}}\frac{T_{\mathrm{int}}}{T_{\mathrm{tl}}}$, where k_{bl} is the combined photobleaching/blinking rate constant and is corrected by the ratio of the experimental image integration time T_{int} and time-lapse T_{tl}. Once the protein dissociates from the DNA, it will undergo free diffusion; and with an effective diffusion constant $D_{\mathrm{FD}} \sim 3.7\ \mu\mathrm{m}^2/\mathrm{s}$ and T_{tl} = 60 ms, it will diffuse to about 942 nm away, way beyond the r_0 threshold (= 220 nm here).

A good choice of the displacement threshold value r_0 is to ensure that all data points belonging to the SB state in the r-vs.-t trajectories are included; at this condition Supplementary Equation 14 can be further simplified. Specifically, for a protein at the SB state, its effective diffusion constant D_{SB} is much smaller than $r_0^2/4\mathrm{t}$ when $t = T_{\mathrm{tl}}$, and the $\exp(-r_0^2/4D\mathrm{t})$ term in the corrected survival probability $S^{'}(r_0,t)$ is negligible (i.e., for r_0 = 220 nm, T_{tl} = 60 ms, and $D_{\mathrm{SB}} = 0.036\ \mu\mathrm{m}^2/\mathrm{s}$, $1-\exp(-r_0^2/4D\mathrm{t}) = 0.9963 \approx 1$), and only the exponential term survives:

$$S_{\mathrm{SB}}^{'}\left(r_0,t\right)=\exp\left(-k_{\mathrm{eff}}^{\mathrm{SB}}t\right),\ \text{where } k_{\mathrm{eff}}^{\mathrm{SB}}=k_{\mathrm{bl}}\frac{T_{\mathrm{int}}}{T_{\mathrm{tl}}}+k_{-1}^{\mathrm{app}} \qquad 15$$

At the same time, however, there are still finite probabilities for a protein at the FD or NB state to not have moved beyond r_0 for a certain period of time, and they thus also contribute to the experimentally extracted residence time distribution and need to be considered.

The function form of the corrected survival probability for a protein at the NB or FD state is the same as Supplementary Equation 14 (the value of the diffusion constants would be different, of course). However, the corresponding rate parameters in the k_{eff} term are different. For a protein molecule at the FD state, since FD states do not have a protein dissociation event, k_{eff} is simply $k_{\text{bl}}\frac{T_{\text{int}}}{T_{\text{tl}}}$. For a protein molecule at the NB state, k_{eff} is a sum of $k_{\text{bl}}\frac{T_{\text{int}}}{T_{\text{tl}}}$ and k_{-2}, which is the unbinding rate constant from nonspecific sites. Therefore, we have:

$$S'_{\text{FD}}(r_0,t)=\left(1-\exp\left(-\frac{r_0^2}{4D_{\text{FD}}t}\right)\right)\exp\left(-k_{\text{eff}}^{\text{FD}}t\right),\ k_{\text{eff}}^{\text{FD}}=k_{\text{bl}}\frac{T_{\text{int}}}{T_{\text{tl}}} \quad 16$$

$$S'_{\text{NB}}(r_0,t)=\left(1-\exp\left(-\frac{r_0^2}{4D_{\text{NB}}t}\right)\right)\exp\left(-k_{\text{eff}}^{\text{NB}}t\right),\ k_{\text{eff}}^{\text{NB}}=k_{\text{bl}}\frac{T_{\text{int}}}{T_{\text{tl}}}+k_{-2} \quad 17$$

With the above corrected survival probability functions, we can now evaluate their corresponding residence time distribution functions $\varphi(\tau)$. $\varphi_{\text{I}}(\tau)\Delta\tau$ is the probability that the single fluorescently-tagged protein disappeared to beyond a circle of radius r_0 in the time interval (τ, $\tau+\Delta\tau$), where $\text{I}\in[\text{FD,NB,SB}]$. Therefore, $\varphi_{\text{I}}(\tau)\Delta\tau$ is equal to the survival probability decrement in time interval (τ, $\tau+\Delta\tau$), i.e., $S'_{\text{I}}(\tau)-S'_{\text{I}}(\tau+\Delta\tau)$. In the limit of infinitesimal $\Delta\tau$, $\varphi_{\text{I}}(\tau)$ is equal to $-\left.\frac{\partial S'_{\text{I}}(t)}{\partial t}\right|_{t=\tau}$. Then we obtain:

$$\varphi_{\text{SB}}(\tau)=k_{\text{eff}}^{\text{SB}}\exp\left(-k_{\text{eff}}^{\text{SB}}\tau\right) \quad 18$$

$$\varphi_{\text{FD}}(\tau)=\left(\frac{r_0^2}{4D_{\text{FD}}\tau^2}\exp\left(-\frac{r_0^2}{4D_{\text{FD}}\tau}\right)+k_{\text{eff}}^{\text{FD}}\left(1-\exp\left(-\frac{r_0^2}{4D_{\text{FD}}\tau}\right)\right)\right)\exp\left(-k_{\text{eff}}^{\text{FD}}\tau\right) \quad 19$$

$$\varphi_{\text{NB}}(\tau)=\left(\frac{r_0^2}{4D_{\text{NB}}\tau^2}\exp\left(-\frac{r_0^2}{4D_{\text{NB}}\tau}\right)+k_{\text{eff}}^{\text{NB}}\left(1-\exp\left(-\frac{r_0^2}{4D_{\text{NB}}\tau}\right)\right)\right)\exp\left(-k_{\text{eff}}^{\text{NB}}\tau\right) \quad 20$$

We fit the experimental distribution of residence time τ to a linear combination of the above three equations:

$$\varphi(\tau)_{\text{all}}=A_{\text{SB}}\varphi_{\text{SB}}(\tau)+A_{\text{NB}}\varphi_{\text{NB}}(\tau)+A_{\text{FD}}\varphi_{\text{FD}}(\tau) \quad 21$$

The effective diffusion constants (D's) and their corresponding fractional populations (A's) are from analyzing the CDF/PDF of the displacement r (Section 11). k_{bl} is determined independently (Section 10). The unbinding rate constant from nonspecific sites k_{-2} is determined by analyzing the distribution of τ at

the highest cellular concentration, where $A_{SB} \approx 0$ (Section 14.2). The only floating parameter in fitting the distribution of τ across a range of cellular concentrations is the apparent unbinding rate constant from the specific recognition sites, k_{-1}^{app}.

It is important to note that in Supplementary Equation 21, the $\varphi_{SB}(\tau)$ could use the full derivative of Supplementary Equation 14), without using the previously justified approximation of $\exp(-r_0^2/4D_{SB}t) \approx 1$ (see above), so that r_0 is explicitly included as well. Additionally, using a linear combination of residence time distributions in Supplementary Equation 21 also assumes a quasi-state system, i.e., interconversion between different states is slower than the experimental time resolution (60 ms for T_{tl} in our experiments). This is the same approximation for using a linear combination of PDFs/CDFs of r (Section 11.2), and this approximation is validated in Section 18.4.

14.2 Extraction of kinetic parameters for protein unbinding from DNA via the residence time distributions

In this section we describe how we extract the kinetic parameters of protein unbinding from DNA using the formulations derived in Section 14.1 above. This procedure involves analyzing the SMT data to determine the contributions of FD, NB, and SB states to the overall residence time distribution $\varphi(\tau)_{all}$ as in Supplementary Equation 21. We then apply the analysis to CueR and ZntR and extract their kinetic parameters.

Diffusion constants (D_{FD}, D_{NB}, and D_{SB}) and fractional populations (A_{FD}, A_{NB}, and A_{SB}) of the three states are obtained from a global fit to the CDFs of r across different cellular protein concentrations (e.g., Supplementary Figure 22b and c). Note that the fraction population of each state changes across different cellular protein concentrations, while the diffusion constants stay the same.

<u>Contribution of the FD state to $\varphi(\tau)_{all}$, i.e., $A_{FD}\varphi_{FD}(\tau)$ in Supplementary Equation 21</u>

To estimate the contribution of the FD state to $\varphi(\tau)_{all}$ at a cellular protein concentration, we need to determine the A_{FD} at that cellular protein concentration, D_{FD} (i.e., D_1) and k_{bl} as shown in Supplementary Equation 16 and 19. A_{FD} and D_{FD} are obtained from global fits to CDFs of r, as described above. k_{bl} is determined independently from the length distribution of the SMT trajectories as described in Section 10.

<u>Contribution of the NB state to $\varphi(\tau)_{all}$, i.e., $A_{NB}\varphi_{NB}(\tau)$ in Supplementary Equation 21</u>

To estimate the contribution of the NB state to $\varphi(\tau)_{all}$ at a cellular protein concentration, we need to determine A_{NB}, D_{NB}, k_{-2} and k_{bl} as shown in Supplementary Equation 17 and 20. A_{NB}, D_{NB}, and k_{bl} can be obtained in the same way as described above. k_{-2} is the only parameter left to determine. From the cellular protein concentration dependence of CDFs (Supplementary Figure 22b and c), it is clear that with increasing cellular protein concentrations, each protein molecule will spend more time freely diffusing or nonspecifically bound to the DNA, and less time specifically bound at recognition sites. At very high protein concentrations (i.e., $[P]_{cell}$ = 789 nM, Supplementary Figure 22c), the fractional population A_{SB} of the SB state becomes negligible (i.e., less than 5%). Consequently, the contribution of the SB state to the residence time distribution at high protein concentration is insignificant and $\varphi(\tau)_{all}$ is simplified to:

$$\varphi'(\tau)_{all} = A_{NB}\varphi_{NB}(\tau) + A_{FD}\varphi_{FD}(\tau) \qquad 22$$

With the known $A_{FD}\varphi_{FD}(\tau)$, fitting the residence time distribution at the highest cellular protein concentration gives k_{-2}.

Contribution of the SB state to $\varphi(\tau)_{all}$, i.e., $A_{SB}\varphi_{SB}(\tau)$ in Supplementary Equation 21

With known $A_{NB}\varphi_{NB}(\tau)$ and $A_{FD}\varphi_{FD}(\tau)$, fitting the residence time distribution with Supplementary Equation 21 at all other protein concentrations where A_{SB} is higher than 5% gives us k_{-1}^{app}, the concentration-dependent apparent unbinding rate constant from the recognition sites (Supplementary Figure 29). The extracted k_{-1}^{app} can be plotted against free protein concentration, $[P]_{FD}$, which is determined as $[P]_{FD} = [P]_{cell} \times A_{FD}$. Linear fit of this concentration dependent k_{-1}^{app} thus gives the intercept k_{-1}, the 1st-order spontaneous unbinding rate constant from the recognition sites in s^{-1}, and the slope k_f, the 2nd-order facilitated unbinding rate constant from the recognition sites in $\mu M^{-1}s^{-1}$.

14.3 Analysis of relative populations of the FD, NB, and SB states using quasi-equilibrium model

In this section we will present the analysis of the relative populations of the FD, NB, SB states of a tracked regulator in the cell to extract additional kinetic and thermodynamic constants about its interactions with DNA. This analysis is also based on the kinetic model in Supplementary Figure 29, where a regulator protein can be freely diffusing (FD), nonspecifically bound to DNA (NB), or specifically bound at chromosomal recognition sites (SB) in the cell. The kinetic processes that lead to interconversions between these states are indicated on Supplementary Figure 29. Note that k_{-1}^{app}, the apparent rate constant for unbinding from recognition sites, comprises k_{-1} and $k_f[P]_{FD}$. $[P]_{FD}$ represents the concentration of freely diffusing proteins in the cell, and $[PD]_{NB}$ and $[PD]_{SB}$ represent the concentrations of nonspecific bound proteins and specific bound proteins, respectively. We also intentionally restored the interconversion process between the NB and SB states, with the corresponding two apparent rate constants $k_3[D]_{SB}$ and $k_{-3}[D]_{NB}$, where $[D]_{SB}$ and $[D]_{NB}$ are the effective cellular concentrations of vacant specific recognition sites and nonspecific binding sites, respectively. Later on in this section, we will show that these interconversions between the NB and SB states can be neglected, as we approximated in analyzing the residence time distributions earlier in Section 14.1 and 14.2.

We assume that CueR or ZntR can sample the FD, NB, and SB states rapidly in the cell and establishes a quasi DNA binding/unbinding equilibrium, so that equilibrium relationship between concentrations and kinetic parameters can be used to analyze the relative populations of proteins at these three states. This approximation is reasonable for the following reasons:

(1) The rate constants we extracted from the residence time analysis show that the CueR or ZntR binding/unbinding to DNA occur on a timescale of tens to a few hundreds of ms, which is much faster than the protein lifetime in the cell — CueR is stable in the cell over a period of many hours, and ZntR's half-life in the cell is ~30 min[10] (Section 3.3.2). Therefore, a CueR or ZntR molecule should be able to sample a large number of times each of these three states during its lifetime to reach a dynamic equilibrium.
(2) During our experimental imaging time (~30 min), for each cell, the total cellular concentration of CueR does not change much (see results in Section 3.3) and is thus at steady state. The total concentration of ZntR can change (half-life ~ 30 min; Section 3.3), for which our results would represent a time average over the imaging time.

Based on the above quasi-equilibrium approximation, the following relations hold between the concentrations of proteins at different states and the kinetic rate constants:

$$\frac{[PD]_{SB}}{[P]_{FD}} = \frac{k_1[D]_{SB}}{k_{-1}^{app}} = \frac{k_1[D]_{SB}}{k_{-1} + k_f[P]_{FD}} \qquad 23$$

$$\frac{[\mathrm{PD}]_{\mathrm{NB}}}{[\mathrm{P}]_{\mathrm{FD}}} = \frac{k_2[\mathrm{D}]_{\mathrm{NB}}}{k_{-2}} \qquad 24$$

$$\frac{[\mathrm{PD}]_{\mathrm{SB}}}{[\mathrm{PD}]_{\mathrm{NB}}} = \frac{k_3[\mathrm{D}]_{\mathrm{SB}}}{k_{-3}[\mathrm{D}]_{\mathrm{NB}}} \qquad 25$$

Note the $[\mathrm{D}]_{\mathrm{SB}}$ and $[\mathrm{D}]_{\mathrm{NB}}$ are the effective concentrations of vacant specific DNA recognition sites and vacant nonspecific binding sites in the cell, respectively. And $[\mathrm{D}]_{\mathrm{SB}} = [\mathrm{D}_0]_{\mathrm{SB}}-[\mathrm{PD}]_{\mathrm{SB}}$, and $[\mathrm{D}]_{\mathrm{NB}} = [\mathrm{D}_0]_{\mathrm{NB}}-[\mathrm{PD}]_{\mathrm{NB}}$, where $[\mathrm{D}_0]_{\mathrm{SB}}$ and $[\mathrm{D}_0]_{\mathrm{NB}}$ are the effective concentrations of total specific recognition sites and nonspecific binding sites in the cell, respectively.

By substituting $[\mathrm{D}]_{\mathrm{SB}}$ and $[\mathrm{D}]_{\mathrm{NB}}$ with $[\mathrm{D}_0]_{\mathrm{SB}}-[\mathrm{PD}]_{\mathrm{SB}}$ and $[\mathrm{D}_0]_{\mathrm{NB}}-[\mathrm{PD}]_{\mathrm{NB}}$ in Supplementary Equation 23-25 and rearranging the equations results in Supplementary Equation 26-28.

$$\frac{[\mathrm{PD}]_{\mathrm{SB}}}{[\mathrm{P}]_{\mathrm{FD}}} = \frac{A_{\mathrm{SB}}}{A_{\mathrm{FD}}} = \frac{1}{K_{\mathrm{D1}}[\mathrm{D}_0]_{\mathrm{SB}}^{-1} + \left(1+\frac{k_{\mathrm{f}}}{k_1}\right)[\mathrm{D}_0]_{\mathrm{SB}}^{-1}[\mathrm{P}]_{\mathrm{FD}}} = \frac{1}{\mathrm{R}_{\mathrm{FD}}^{\mathrm{SB}} + \mathrm{S}_{\mathrm{FD}}^{\mathrm{SB}}[\mathrm{P}]_{\mathrm{FD}}}; K_{\mathrm{D1}} = \frac{k_{-1}}{k_1} \qquad 26$$

$$\frac{[\mathrm{PD}]_{\mathrm{NB}}}{[\mathrm{P}]_{\mathrm{FD}}} = \frac{A_{\mathrm{NB}}}{A_{\mathrm{FD}}} = \frac{1}{K_{\mathrm{D2}}[\mathrm{D}_0]_{\mathrm{NB}}^{-1} + [\mathrm{D}_0]_{\mathrm{NB}}^{-1}[\mathrm{P}]_{\mathrm{FD}}} = \frac{1}{\mathrm{R}_{\mathrm{FD}}^{\mathrm{NB}} + \mathrm{S}_{\mathrm{FD}}^{\mathrm{NB}}[\mathrm{P}]_{\mathrm{FD}}}; K_{\mathrm{D2}} = \frac{k_{-2}}{k_2} \qquad 27$$

$$\frac{[\mathrm{PD}]_{\mathrm{SB}}}{[\mathrm{PD}]_{\mathrm{NB}}} = \frac{A_{\mathrm{SB}}}{A_{\mathrm{NB}}} = \frac{1}{K_{\mathrm{D3}}\frac{[\mathrm{D}_0]_{\mathrm{NB}}}{[\mathrm{D}_0]_{\mathrm{SB}}} + \frac{(1-K_{\mathrm{D3}})}{[\mathrm{D}_0]_{\mathrm{SB}}}[\mathrm{PD}]_{\mathrm{NB}}} = \frac{1}{\mathrm{R}_{\mathrm{NB}}^{\mathrm{SB}} + \mathrm{S}_{\mathrm{NB}}^{\mathrm{SB}}[\mathrm{PD}]_{\mathrm{NB}}}; K_{\mathrm{D3}} = \frac{k_{-3}}{k_3} \qquad 28$$

For a given cellular protein concentration $[\mathrm{P}]_{\mathrm{cell}}$, which we experimentally measure, we can obtain $[\mathrm{P}]_{\mathrm{FD}}$, $[\mathrm{PD}]_{\mathrm{NB}}$, and $[\mathrm{PD}]_{\mathrm{SB}}$ via $[\mathrm{P}]_{\mathrm{cell}} \times A_{\mathrm{FD}}$, $[\mathrm{P}]_{\mathrm{cell}} \times A_{\mathrm{NB}}$, and $[\mathrm{P}]_{\mathrm{cell}} \times A_{\mathrm{SB}}$, respectively, where A_{FD}, A_{NB}, and A_{SB} are the fractional populations of FD, NB, and SB states as determined from the CDFs in Supplementary Figure 22c. We can then plot out the experimental $[\mathrm{PD}]_{\mathrm{SB}}/[\mathrm{P}]_{\mathrm{FD}}$ vs. $[\mathrm{P}]_{\mathrm{FD}}$, $[\mathrm{PD}]_{\mathrm{NB}}/[\mathrm{P}]_{\mathrm{FD}}$ vs. $[\mathrm{P}]_{\mathrm{FD}}$, and $[\mathrm{PD}]_{\mathrm{SB}}/[\mathrm{PD}]_{\mathrm{NB}}$ vs. $[\mathrm{PD}]_{\mathrm{NB}}$ (Supplementary Figure 30) whose relationships are given in Supplementary Equation 26-28. Using k_{-2}, k_{-1}, and k_{f} from the residence time analysis in Sections 14.1 and 14.2 earlier, we can fit such data in Supplementary Figure 30 with Supplementary Equation 26-28 to obtain $\mathrm{R}_{\mathrm{FD}}^{\mathrm{SB}} = K_{\mathrm{D1}}[\mathrm{D}_0]_{\mathrm{SB}}^{-1}$, $\mathrm{S}_{\mathrm{FD}}^{\mathrm{SB}} = \left(1+\frac{k_{\mathrm{f}}}{k_1}\right)[\mathrm{D}_0]_{\mathrm{SB}}^{-1}$, $\mathrm{R}_{\mathrm{FD}}^{\mathrm{NB}} = K_{\mathrm{D2}}[\mathrm{D}_0]_{\mathrm{NB}}^{-1}$, $\mathrm{S}_{\mathrm{FD}}^{\mathrm{NB}} = [\mathrm{D}_0]_{\mathrm{NB}}^{-1}$, $\mathrm{R}_{\mathrm{NB}}^{\mathrm{SB}} = K_{\mathrm{D3}}[\mathrm{D}_0]_{\mathrm{NB}}[\mathrm{D}_0]_{\mathrm{SB}}^{-1}$, and $\mathrm{S}_{\mathrm{NB}}^{\mathrm{SB}} = (1-K_{\mathrm{D3}})[\mathrm{D}_0]_{\mathrm{SB}}^{-1}$, from which we can obtain k_2, $[\mathrm{D}_0]_{\mathrm{NB}}$, k_1, and $[\mathrm{D}_0]_{\mathrm{SB}}$. Because of the cyclic nature of the mechanistic model in Supplementary Figure 29, these kinetic and thermodynamic parameters can be calculated from two pathways as described below.

Pathway 1 for determining the kinetic parameters

Pathway 1 includes the following steps:

(*i*) Determine K_{D2} ($=k_{-2}/k_2$) and $[\mathrm{D}_0]_{\mathrm{NB}}$ from $[\mathrm{PD}]_{\mathrm{NB}}/[\mathrm{P}]_{\mathrm{FD}}$ fit. With known k_{-2}, we get k_2.

(*ii*) Determine K_{D3} ($=k_{-3}/k_3$) and $[\mathrm{D}_0]_{\mathrm{SB}}$ from $[\mathrm{PD}]_{\mathrm{SB}}/[\mathrm{PD}]_{\mathrm{NB}}$ fit.

(*iii*) Determine K_{D1} ($=k_{-1}/k_1$) from $[\mathrm{PD}]_{\mathrm{SB}}/[\mathrm{P}]_{\mathrm{FD}}$ fit. With known k_{-1} and k_{f}, we get k_1.

In Step (*i*), we fit $[\mathrm{PD}]_{\mathrm{NB}}/[\mathrm{P}]_{\mathrm{FD}}$ data with Supplementary Equation 27 and $1/\mathrm{S}_{\mathrm{FD}}^{\mathrm{NB}}$ directly gives $[\mathrm{D}_0]_{\mathrm{NB}}$. Using determined k_{-2} and $[\mathrm{D}_0]_{\mathrm{NB}}$, k_2 can be obtained from Supplementary Equation 29.

$$k_2 = \frac{k_{-2}}{\mathrm{R}_{\mathrm{FD}}^{\mathrm{NB}}[\mathrm{D}_0]_{\mathrm{NB}}} \qquad 29$$

In Step (*ii*), we fit $[\mathrm{PD}]_{\mathrm{SB}}/[\mathrm{PD}]_{\mathrm{NB}}$ data with Supplementary Equation 28. With known $[\mathrm{D}_0]_{\mathrm{NB}}$, k_{-3}/k_3 can be extracted from Supplementary Equation 30.

$$\frac{k_{-3}}{k_3} = 1 - \frac{\mathrm{S}_{\mathrm{NB}}^{\mathrm{SB}}}{\mathrm{R}_{\mathrm{NB}}^{\mathrm{SB}}}[\mathrm{D}_0]_{\mathrm{NB}} \tag{30}$$

Using k_{-3}/k_3 and $[\mathrm{D}_0]_{\mathrm{NB}}$, $[\mathrm{D}_0]_{\mathrm{SB}}$ can be obtained from Supplementary Equation 31.

$$[\mathrm{D}_0]_{\mathrm{SB}} = \frac{k_{-3}}{k_3}\frac{[\mathrm{D}_0]_{\mathrm{NB}}}{\mathrm{R}_{\mathrm{NB}}^{\mathrm{SB}}} \text{ or } [\mathrm{D}_0]_{\mathrm{SB}} = \left(1 - \frac{k_{-3}}{k_3}\right)\frac{1}{\mathrm{S}_{\mathrm{NB}}^{\mathrm{SB}}} \tag{31}$$

In Step (*iii*), we fit $[\mathrm{PD}]_{\mathrm{SB}}/[\mathrm{P}]_{\mathrm{FD}}$ with Supplementary Equation 26. With known k_{-1}, k_{f}, $[\mathrm{D}_0]_{\mathrm{SB}}$, and $[\mathrm{D}_0]_{\mathrm{NB}}$, k_1 can be obtained from Supplementary Equation 32.

$$k_1 = \frac{k_{-1}}{\mathrm{R}_{\mathrm{FD}}^{\mathrm{SB}}[\mathrm{D}_0]_{\mathrm{SB}}} \text{ or } k_1 = \frac{k_1 + k_{\mathrm{f}}}{\mathrm{S}_{\mathrm{FD}}^{\mathrm{SB}}[\mathrm{D}_0]_{\mathrm{SB}}} \tag{32}$$

Pathway 2 for determining the kinetic parameters

Pathway 2 includes the following steps:

(*i*) Determine K_{D2} ($=k_{-2}/k_2$) and $[\mathrm{D}_0]_{\mathrm{NB}}$ from $[\mathrm{PD}]_{\mathrm{NB}}/[\mathrm{P}]_{\mathrm{FD}}$ fit. With known k_{-2}, we get k_2.
(*ii*) Determine K_{D1} ($=k_{-1}/k_1$) and $[\mathrm{D}_0]_{\mathrm{SB}}$ from $[\mathrm{PD}]_{\mathrm{SB}}/[\mathrm{P}]_{\mathrm{FD}}$ fit. With known k_{-1} and k_{f}, we get k_1.
(*iii*) Determine K_{D3} ($=k_{-3}/k_3$) from $[\mathrm{PD}]_{\mathrm{SB}}/[\mathrm{PD}]_{\mathrm{NB}}$ fit with known $[\mathrm{D}_0]_{\mathrm{SB}}$ and $[\mathrm{D}_0]_{\mathrm{NB}}$.

Step (*i*) is exactly the same as described in Pathway 1 to extract $[\mathrm{D}_0]_{\mathrm{NB}}$ and k_2.

In Step (*ii*), we fit $[\mathrm{PD}]_{\mathrm{SB}}/[\mathrm{P}]_{\mathrm{FD}}$ with Supplementary Equation 26. With known k_{-1}, k_{f}, and $[\mathrm{D}_0]_{\mathrm{NB}}$, k_1 can be obtained from Supplementary Equation 33. $[\mathrm{D}_0]_{\mathrm{SB}}$ can then be obtained via Supplementary Equation 34.

$$k_1 = k_{-1}\frac{\mathrm{S}_{\mathrm{FD}}^{\mathrm{SB}}}{\mathrm{R}_{\mathrm{FD}}^{\mathrm{SB}}} - k_{\mathrm{f}} \tag{33}$$

$$[\mathrm{D}_0]_{\mathrm{SB}} = \frac{k_{-1}}{k_1\mathrm{R}_{\mathrm{FD}}^{\mathrm{SB}}} \text{ or } [\mathrm{D}_0]_{\mathrm{SB}} = \frac{k_1 + k_{\mathrm{f}}}{k_1\mathrm{S}_{\mathrm{FD}}^{\mathrm{SB}}} \tag{34}$$

In step (*iii*), we fit $[\mathrm{PD}]_{\mathrm{SB}}/[\mathrm{PD}]_{\mathrm{NB}}$ data with Supplementary Equation 28. With known $[\mathrm{D}_0]_{\mathrm{SB}}$ and $[\mathrm{D}_0]_{\mathrm{NB}}$, k_{-3}/k_3 can be extracted from Supplementary Equation 35.

$$\frac{k_{-3}}{k_3} = \mathrm{R}_{\mathrm{NB}}^{\mathrm{SB}}\frac{[\mathrm{D}_0]_{\mathrm{SB}}}{[\mathrm{D}_0]_{\mathrm{NB}}} \text{ or } \frac{k_{-3}}{k_3} = 1 - \mathrm{S}_{\mathrm{NB}}^{\mathrm{SB}}[\mathrm{D}_0]_{\mathrm{SB}} \tag{35}$$

From $[\mathrm{D}_0]_{\mathrm{SB}}$ and $[\mathrm{D}_0]_{\mathrm{NB}}$, we can obtain the effective numbers of recognition sites (N_{SB}) and nonspecific binding sites (N_{NB}) in the cell using the cell volume determined from cell's transmission image (Supplementary Figure 13a).

We obtained the kinetic and thermodynamic parameters using both pathways and the resulting values are all within error bar, the values with smallest error bars are summarized in Supplementary Table 7 and Supplementary Table 8.

14.4 Example: residence time and population analysis of $\mathrm{CueR}_{\mathrm{apo}}^{\mathrm{mE}}$

Here we use $\text{CueR}^{\text{mE}}_{\text{apo}}$ as an example to demonstrate the analysis procedure described above to extract its unbinding/binding kinetic parameters in interactions with DNA in the cell. The procedure includes following steps:

(1) Sort cells into groups of similar cellular protein concentrations, and for each group, compile the SMT data to generate the corresponding CDF of *r*. Globally fit the CDFs across the cellular protein concentrations, which gives the diffusion constants and fractional populations of the three diffusive states at each concentration as shown in Supplementary Figure 31a and b.
(2) Determine the photobleaching/blinking rate constant of mEos3.2 by fitting the τ_{on} distribution of SMT trajectories within *each* concentration group to prove/check that k_{bl} shows no dependence on cellular protein concentration. k_{bl} is then re-determined by globally fitting the data across all cellular protein concentrations (Supplementary Figure 31c); this value of k_{bl} is used for residence time analysis.
(3) Fit the residence time distribution at the highest protein concentration where A_{SB} is < 5% with Supplementary Equation 22 to get k_{-2} as shown in Supplementary Figure 31d.
(4) Fit the residence time distributions from groups where the A_{SB} is > 5% with Supplementary Equation 21 using diffusion constants, fractional populations, k_{bl}, and k_{-2} determined in steps 1-3 to get k_{-1}^{app} for each group. Linearly fit the k_{-1}^{app} vs. $[\text{P}]_{\text{FD}}$ plot to get k_{-1} and k_{f} as shown in Supplementary Figure 31e and f.
(5) Using k_{-2}, k_{-1}, and k_{f} from step 1-4, we can fit $[\text{PD}]_{\text{SB}}/[\text{P}]_{\text{FD}}$ vs. $[\text{P}]_{\text{FD}}$, $[\text{PD}]_{\text{NB}}/[\text{P}]_{\text{FD}}$ vs. $[\text{P}]_{\text{FD}}$, and $[\text{PD}]_{\text{SB}}/[\text{PD}]_{\text{NB}}$ vs. $[\text{PD}]_{\text{NB}}$ with Supplementary Equation 26-28 to further obtain k_2, $[\text{D}_0]_{\text{NB}}$, k_1, and $[\text{D}_0]_{\text{SB}}$ as shown in Supplementary Figure 31g-i.

Same analysis is performed for other CueR or ZntR variants and growth conditions and the k_{-1}^{app} vs. $[\text{P}]_{\text{FD}}$ results are summarized in Supplementary Figure 32.

14.5 Validation that direct interconversions between NB and SB states are negligible

We examined the validity of our approximation that direct interconversion between NB and SB states are negligible kinetically in our mechanistic model (Supplementary Figure 29). The time of direct transition from NB to SB can be approximated by the average searching time along DNA for a transcription factor, which has been measured to be ~ 3.5 minutes by Hammar and Li *et al.* for the transcription factor LacI [59,60], corresponding to a rate r_{NS} of 0.0048 s^{-1}.

From our model, the transition rates from NB to SB (r_{NS}) and from SB to NB (r_{SN}) will equal to $k_3[\text{D}]_{\text{SB}}$ and $k_{-3}[\text{D}]_{\text{NB}}$, respectively. The ratio of the two transition rates (i.e., $r_{\text{SN}}/r_{\text{NS}}$) is then calculated from the ratio of $k_{-3}[\text{D}]_{\text{NB}}$ and $k_3[\text{D}]_{\text{SB}}$. Using r_{NS} of 0.0048 s^{-1} from the literature and the ratio of $k_{-3}[\text{D}]_{\text{NB}}$ and $k_3[\text{D}]_{\text{SB}}$ (i.e., $\frac{k_{-3}[\text{D}]_{\text{NB}}}{k_3[\text{D}]_{\text{SB}}}$) from our fitting result, we can then calculate the r_{SN} via

$$r_{SN} = \frac{k_{-3}[\text{D}]_{\text{NB}}}{k_3[\text{D}]_{\text{SB}}} \times r_{NS} \tag{36}$$

Note that $\frac{k_{-3}[\text{D}]_{\text{NB}}}{k_3[\text{D}]_{\text{SB}}}$ is equal to $\frac{[\text{PD}]_{\text{NB}}}{[\text{PD}]_{\text{SB}}}$ according to Supplementary Equation 25) where it varies from 1.6 to 5.9 according to the result shown in Supplementary Figure 31I (note this figure plots $[\text{PD}]_{\text{SB}}/[\text{PD}]_{\text{NB}}$). This gives the r_{SN} a highest rate of 0.028 s^{-1}, corresponding to a timescale of ~35 s. Comparing with the slowest interconversion time (i.e., hundreds of ms) between NB and FD and between

SB and FD states (Supplementary Table 7), interconversion between SB and NB would be two to three orders of magnitude slower, which validates our approximation that interconversion between NB and SB states are kinetically negligible in our analysis of the CueR or ZntR unbinding from DNA.

14.6 The limiting case that the D_{NB} state were to be entirely an artifact from cell confinement effect on the free diffusion state: Conclusion of concentration-dependent protein unbinding stays the same

In analyzing the CDF of r of the SMT trajectories of free mEos3.2, we needed to use two diffusion states to fit the data satisfactorily, even though mEos3.2 has only one free diffusion state in *E. coli*. This discrepancy comes from the effect of the cell confinement in combination with the time-lapse imaging mode. The faster component of the two fitted diffusion states reflects the effective diffusion constant at the respective time-lapse, whereas the slower component is an artifact, as confirmed by our simulations (Section 15.2). This confinement effect is more significant for faster-diffusing proteins and it becomes insignificant and unobservable when the protein's intrinsic diffusion constant is smaller than 1.88 $\mu m^2/s$ (which corresponds to an apparent diffusion constant of ~1.5 $\mu m^2/s$ from CDF analysis at T_{tl} = 60 ms). As a result, the middle diffusion state D_{NB} in our analysis of the CDFs of CueR or ZntR contains some contribution from the fast diffusion state D_{FD}. In other words, A_{NB} could potentially be an overestimate of the fractional population of the NB state while A_{FD} is an underestimate.

To probe how this complication may affect our residence time analysis, here we treat the limiting case that the entire D_2 state is a contamination from the D_{FD} state, i.e., this NB state is not present and D_{NB} is just another free-diffusing state with a smaller effective diffusion constant. For treating the residence time distribution, this limiting case is equivalent to setting k_{-2} = 0 in $\varphi(\tau)_{\text{all}}$ (reference Supplementary Equation 20 and 21). The resulting k_{-1}^{app} vs. $[P]_{FD}$ plots of all CueR or ZntR variants are summarized in Supplementary Figure 33 for this limiting case. Although the slopes and intercepts are different from Supplementary Figure 32, k_{-1}^{app} for all variants still show a clear linear dependence on the cellular protein concentration. *Therefore, whether or not the cell confinement affects the quantitative extraction of the kinetic parameters, our conclusions remain the same: both CueR and ZntR have concentration-dependent unbinding from their chromosomal recognition sites in a cell, suggesting that the facilitated unbinding mechanism (e.g., assisted dissociation or direct substitution) also operate in vivo.*

15. Validation of data analysis using simulations of 1-state and multi-state diffusions in a cell

15.1 Simulation algorithm for molecular diffusion in an E. coli cell with one or multiple interconverting diffusion states

Two-dimensional simulated diffusion trajectories were generated by a 2-D stochastic random walk process with an appropriate cell shape in MATLAB to approximate the real diffusion for the fluorescently tagged proteins in *E. coli* cells, and the simulated results were used to check and validate our data analysis procedures described in Section 14.

The algorithm of our stochastic 2-D diffusion simulation method follows the procedures below:

(1) Input cell geometry (length and width; the 2-D cell geometry is modeled as a rectangle capped by two hemi-circles), the number of diffusion states (e.g., FD, NB, and SB states), their associated diffusion constants D_I (which could come from experimental results; $I \in [\text{FD,NB,SB}]$), and the rate constants for interconversions between these states (e.g., rate constant $\gamma_{J,I}$ for interconversion

from state I to J ($J \neq I$; and $I, J \in$ [FD,NB,SB]), which could also be taken from experimental results).

(2) Build a sequence of residence time t_I, for example $t_{\mathrm{FD}} \rightarrow t_{\mathrm{SB}} \rightarrow t_{\mathrm{FD}} \rightarrow \cdots$, where each residence time t_I samples the residence time distribution $\exp(-\sum_J \gamma_{J,I} t)$, where Σ_J is a sum of all competing processes, leaving from state I to state J $(J \neq I)$, each with a rate constant $\gamma_{J,I}$. The transition from state I to a particular state J follows the relative probability $\frac{\gamma_{J,I}}{\sum_J \gamma_{J,I}}$, and the residence time sequence is terminated by t_{bl}, which equals the sum of residence time t_I in the sequence and samples the distribution $\exp(-k_{\mathrm{bl}} \frac{T_{\mathrm{int}}}{T_{\mathrm{tl}}} t)$ limited by photobleaching/blinking.

(3) Sample the initial position (x,y) randomly within the cell geometry.

(4) During each state, generate the displacements of x and y (i.e., dx and dy) within the laser integration time (= 4 ms) following the solution of the diffusion equation using that state's diffusion constant D_I (for example, dx is a random displacement and it follows the distribution $\frac{1}{\sqrt{4\pi Dt}} e^{-\frac{x^2}{4Dt}}$ from solving the 1-D diffusion equation), such that (n)'th position will be the sum of $(n-1)$'s and the displacement dx and dy.

(5) Apply reflecting boundary if (n)'th position falls outside of the cell boundary.

(6) The primary simulated trajectory is at the resolution of laser integration time (= 4 ms), and this simulated trajectory will be sampled in a time lapse fashion at every lapse time T_{tl} (= 60 ms) to give the eventual simulated diffusion trajectory, which is analyzed.

From the above simulation, we obtained a sequence of positions inside the cell. Supplementary Figure 34d shows an example of simulated diffusion position trajectory, which includes 3 interconverting states with diffusion constants D_{input} = 11, 0.7, and 0.036 μm^2/s, respectively.

15.2 Simulation with a single diffusion state and the corresponding analysis of CDF of r

For slow diffusing molecules (intrinsic *D* < 1.88 μm^2/s), CDF analysis reliably determines the number of the diffusion state, but for relatively fast diffusing molecules (intrinsic *D* > 1.88 μm^2/s), it leads to an artificial slow minor diffusing component.

From the experimental SMT data, we analyzed the CDF of r (plotted against $r^2/4T_{\mathrm{tl}}$) to extract diffusion constants and amplitudes of the diffusion states. Here we use simulations to check and validate this analysis of diffusion trajectories. We first performed the CDF analysis on the simulated results that contained only one diffusion state, and fitted the CDF with a single diffusion component following the equation: $CDF(r) = 1 - \exp(-\frac{r^2}{4DT_{\mathrm{tl}}})$. Only the first displacement in each simulated trajectory was used, as we did in analyzing the experimental SMT data.

Supplementary Figure 35 shows the CDF analysis of the simulated results, where the simulation contains a single diffusion state with the input diffusion constant of 11, 0.7, or 0.036 um^2/s. The apparent D extracted from the CDF gets closer to the input D value as the input D becomes smaller. For example, for D_{input} = 11 μm^2/s, the extracted D from the CDF fitting is 3.5 μm^2/s; but for D_{input} = 0.036 μm^2/s, the extracted one from CDF analysis is virtually identical at 0.034 μm^2/s. The significant difference between the input D and the extracted apparent D for larger input D values is due to the confinement effect of the cell. As D becomes greater, the molecule could travel further and is thus more affected by the cell

boundary. Therefore, a greater boundary effect applies to states with larger D values, resulting in a more decreased apparent D extracted from CDF analysis.

Moreover, for large D_{input} (e.g., Supplementary Figure 35a), using one single diffusion component cannot sufficiently fit well the CDF, especially at the regime of large displacements (i.e., large $r^2/4T_{\text{tl}}$); this again comes from the cell confinement effect and calls for an additional diffusion component to account for the data, which would be artificial.

To probe how the confinement effect would influence the results in CDF fitting using multiple diffusion components, we tried fitting the CDF using a combination of two components (i.e., with two diffusion constants D_{major} and D_{minor}, where D_{major} is the faster component; $CDF(r) = A\left[1 - \exp\left(-\frac{r^2}{4D_{major}T_{tl}}\right)\right] + (1-A)\left[1 - \exp\left(-\frac{r^2}{4D_{minor}T_{tl}}\right)\right]$, even though the simulation only contained one diffusion state. Supplementary Figure 36a shows how the amplitude of the major, faster component D_{major} from the 2-component CDF fitting depends on the input diffusion constant (D_{input}) of the 1-state simulations. In the range of $D_{\text{input}} \leq 1.88$ μm^2/s, the amplitude is ≥ 95%, which means for a relatively slow diffusing molecule in a cell, its CDF of r can be sufficiently fitted with a single diffusion component, and a 2nd component is negligible and unnecessary.

However, in the range of $D_{\text{input}} > 1.88$ μm^2/s and up to 12 μm^2/s (the upper limit here is larger than the typical intrinsic diffusion constant for a free diffusing small protein in bacterial cytoplasm, which is around 10-11 μm^2/s) [18] (Supplementary Figure 36a), the amplitude of the major, faster component is <95%, and the second, minor, slower component is non-negligible. This means that for a relatively fast diffusing molecule, even though it only has one diffusion state, its CDF needs two components to fit satisfactorily, giving rise to an artificial minor component with a smaller diffusion constant.

Taking into account all above, we decided to use the diffusion constant of 1.88 μm^2/s as a divide: when $D_{\text{input}} \leq 1.88$ μm^2/s, its CDF is satisfactorily fitted with a single component with the extracted apparent diffusion constant ($D_{\text{extracted}}$) slightly smaller (Supplementary Figure 36b); and at $D_{\text{input}} = 1.88$ μm^2/s, the extracted apparent D from CDF analysis is 1.5 μm^2/s. When $D_{\text{input}} > 1.88$ μm^2/s, its CDF is fitted with two components, the faster component is the major one, whose diffusion constant ($D_{\text{major}} > 1.5$ μm^2/s) reflects the apparent diffusive behavior of the molecule in the cell, whereas the slower, minor component, is artificial from the CDF analysis. The extracted diffusion constants of the CDF analysis of 1-diffusion-state simulation are plotted together in Supplementary Figure 36b.

These results indicate that for a molecule that has multiple diffusion states in the cell, if one of the states has a fast intrinsic diffusion constant (i.e., > 1.88 μm^2/s), this fast diffusion state will contaminate slower diffusion states in the CDF analysis of its displacements. Specifically, for CueR or ZntR's free diffusion (FD) state with an apparent diffusion constant ~3.7 ± 0.3 μm^2/s, it has an accompanying artificial slower component at ~1.1 μm^2/s, which would be unresolvable from the nonspecific bound (NB) state that has an apparent diffusion constant of ~0.7 μm^2/s. To probe the possible consequence of this complication on our data analysis, we have considered two limiting cases of our analysis of the CDF of CueR or ZntR diffusion in the cells (Section 14): in one limiting case, the medium diffusive state D_2 is considered as a clean NB state without any contamination (this is the default case in our data analysis); in the other limiting case, the medium diffusive state D_2 is considered as a second FD state, i.e., completely coming from the contamination. In both cases, the extracted unbinding rate constant from the recognition sites show consistent increasing trends with increasing cellular protein concentrations. *Therefore, our conclusion of concentration-dependent protein unbinding kinetics from recognition sites is always valid,*

regardless of the imperfection of CDF analysis in quantifying the underlying diffusion states and their diffusion constants.

15.3 Simulation with three interconverting diffusion states, the corresponding analysis of CDF of r, and the analysis of residence time distributions to extract unbinding kinetics

15.3.1 CDF analysis of simulations containing three interconverting diffusion states provides the amplitude of each state and the corresponding apparent diffusion constant

We performed simulations that contain three diffusion states, representing the FD, NB, and SB states we observed experimentally, with variable interconversion rate constants between them. The input rate parameters for the simulations were taken from the experimental results on $\mathrm{CueR}_{\mathrm{apo}}^{\mathrm{mE}}$, and the input diffusion constants were the expected intrinsic diffusion constants (Supplementary Figure 36b) for these three states on the basis of their apparent diffusion constants determined experimentally (Supplementary Table 6). No interconversion was allowed between the NB and SB states, as in our model in analyzing the experimental data (Supplementary Figure 29).

Three separate simulations, each containing three diffusion states, were performed, in which the interconversion rate constants correspond to those observed at the three cellular $\mathrm{CueR}_{\mathrm{apo}}^{\mathrm{mE}}$ concentrations at 180 nM, 338 nM, and 926 nM, respectively. The input diffusion constants and rate parameters are summarized in Supplementary Table 6. The input interconversion rate constants determine the input fractional populations of the three diffusion states in each of the simulations.

Then, as we did in analyzing the experimental data of $\mathrm{CueR}_{\mathrm{apo}}^{\mathrm{mE}}$, we used three-components to globally fit the CDFs of r (plotted against $r^2/4T_{\mathrm{tl}}$) from the simulations to extract the apparent diffusion constants (which were shared in the global fitting) and their amplitudes in each simulation. Supplementary Figure 37a-c show the CDFs of displacement r from the three simulations and the fitting results.

The extracted three apparent diffusion constants from the CDF analysis are D_1 = 3.55 μm^2/s, D_2 = 0.74 μm^2/s, and D_3 = 0.037 μm^2/s, nicely matched the apparent diffusion constants at 3.7 ± 0.2, 0.70 ± 0.03, and 0.036 ± 0.009 μm^2/s expected from the input intrinsic diffusion constants, respectively (Supplementary Table 6). The extracted amplitudes (i.e., fractional population) of these three effective diffusive states also show the same trend as the input with increasing protein concentrations (Supplementary Figure 37e). With respect to the exact values of their amplitudes, although A_2 is underestimated and A_1 is overestimated, A_3 is quantitatively reproduced in the CDF analysis and it is the most important component for our analysis of the SB state. *Therefore, the CDF analysis can resolve the three diffusion states and their apparent diffusion constants, and the extracted fractional populations of these states show a correct trend, with quantitative determination of the fractional population of the SB state.*

15.3.2 Simulation and the subsequent analysis reproduces the photoblinking/bleaching kinetics of the mEos3.2 tag

In our simulations, the photobleach/blinking kinetics was included as an input parameter (see Section 15.1 on simulation algorithm). The temporal length distribution of the simulated trajectories, each of which ends at a photobleaching/blinking event, provides a direct check. Supplementary Figure 38a shows

the distribution of the length of the simulated trajectories. A fit with $\exp(-k_{\mathrm{bl}}\frac{T_{\mathrm{int}}}{T_{\mathrm{tl}}}t)$ gives k_{bl} = 269 ± 1 s^{-1} with T_{int} = 4 ms and T_{tl} = 60 ms, within ~7% of the input value of k_{bl} = 252 s^{-1}.

15.3.3 Two-diffusion-state simulation (i.e., FD and NB states) allows for the determination of the nonspecific unbinding rate constant k_{-2}.

Experimentally, γ_{21} (i.e., k_{-2}) was extracted from the fit of residence time distribution at the highest protein concentration where the fractional population of the SB state is <5% (Section 14). We used simulation to validate this approach, and performed a simulation with two diffusion states (i.e., corresponding to the FD and NB states), where the input interconversion rate constants were taken from those of $\mathrm{CueR}_{\mathrm{apo}}^{\mathrm{mE}}$ at the highest cellular protein concentration (~1375 nM). The input parameters are also summarized in Supplementary Table 6. Supplementary Figure 37d shows the 3-component CDF fitting of this two-state simulation (as part of the global fit together with Supplementary Figure 37a-c) — indeed, the fitted 3rd component (A_3 ~ 2%, Supplementary Figure 37e) is negligible (i.e., < 5%).

Using the simulated position trajectories, we then obtained the simulated displacement vs. time trajectories, to which we applied the r_0 = 220 nm thresholding to obtain the individual residence time τ. Supplementary Figure 38b shows the distribution of the residence time τ and the fitting with two components using Supplementary Equation 22. The fitting gives γ_{21} (i.e., k_{-2}) = 12.4 ± 2.3 s^{-1}, which is higher than the input of simulation (2.6 s^{-1}), indicating that in our analysis of experimental data, γ_{21} (i.e., k_{-2}) is likely overestimated. Nevertheless, this overestimation of γ_{21} (i.e., k_{-2}) does not affect the trend that the extracted γ_{31} (i.e., k_{-1}^{app}) increases with increasing cellular protein concentration (see below).

15.3.4 Three-diffusion-state simulation (i.e., FD, NB, and SB states) allow for the determination of the apparent unbinding rate constant from recognition sites, k_{-1}^{app}

From the 3-state diffusion simulations, we also extracted the residence times by thresholding the displacement vs. time trajectory at r_0 = 220 nm. Fitting the residence time distribution with Supplementary Equation 21, including contributions from FD, NB, and SB states, gives γ_{31} (i.e., k_{-1}^{app}), the apparent unbinding rate constant from the SB state (i.e., from recognition sites) (Supplementary Figure 38c). γ_{31} (i.e., k_{-1}^{app}) is the only floating parameter in the fit here, whereas the apparent diffusion constants and their fractional populations are from the results in fitting the CDFs of the simulations, k_{bl} is from Supplementary Figure 38a, and γ_{21} (i.e., k_{-2}) from Supplementary Figure 38b. For the simulation corresponding to the cellular protein concentration of ~926 nM, the extracted γ_{31} (i.e., k_{-1}^{app}) from fitting the residence time distribution is 14.4 ± 1.0 s^{-1}, in agreement with the simulation input value of 16.8 s^{-1} (Supplementary Figure 38c).

We analyzed the residence time distributions from the three separate 3-state simulations of different interconversion rates, corresponding to the cellular protein concentrations of 180, 338 and 926 nM (for $\mathrm{CueR}_{\mathrm{apo}}^{\mathrm{mE}}$, as in Supplementary Figure 37a-c). The extracted γ_{31} (i.e., k_{-1}^{app}) are plotted together with the input γ_{31} values of the simulations across the corresponding cellular protein concentrations in Supplementary Figure 39 (also see Supplementary Table 6). It is clear that, even though the extracted γ_{31} (i.e., k_{-1}^{app}) and the input γ_{31} are not identical, they are close in values and the trend that γ_{31} (i.e., k_{-1}^{app}) increases with cellular protein concentration is reliably reproduced. *Therefore, our analysis procedure to extract the protein unbinding kinetics from recognition sites is valid and reliable, in particular in reproducing the trend of its dependence on the cellular protein concentration.*

16. Further validation of data analysis using hidden Markov model analysis of single-particle tracking trajectories

In addition to using 3-state-simulations to validate our data analysis procedures (Section 15.3), we further examined our data with vbSPT (variational Bayes Single Particle Tracking), a software package for hidden Markov Model analysis of single particle tracking data published by Elf and coworkers [61].

We initially allowed maximally $N = 8$ states in vbSPT analysis. The outputs gave the optimal number of states as $N = 3$, 4, 5, or 6, having similar ranking scores (~ −100; the closer to zero the better), all better than $N = 1$ or 2 (score ~ −2000). To have the simplest kinetic model, we used the model with 3 hidden states (input setting: time step = 60 ms and limits of diffusion constant = 0.001 to 10 μm^2/s), and analyzed our experimental position trajectories from cells expressing $\mathrm{CueR}_{\mathrm{apo}}^{\mathrm{mE}}$ with vbSPT across a range of cellular protein concentrations (99-926 nM). Supplementary Figure 40a shows the output results from vbSPT, including diffusion constants (D_1, D_2, and D_3), their fractional populations (area of the blue, green, and red circles for FD, NB, and SB states, respectively), and the interconversion rate constants.

In general, the vbSPT analysis further supports our analysis in Section 14 and conclusions drawn there:

1) The extracted diffusion constants (D_1 ~ 5 μm^2/s, D_2~ 0.9 μm^2/s, and D_3 ~0.04 μm^2/s) and the dependence of fractional populations on protein concentration (i.e., A_{SB} decreases while A_{FD} and A_{NB} increase with protein concentrations, Supplementary Figure 40b) are in general agreement with those of our CDF analysis.

2) The SB → FD transition rate constant $k_{\mathrm{SB}\rightarrow\mathrm{FD}}$ from vbSPT corresponds to k_{-1}^{app}, the apparent unbinding rate constant from recognition sites in our residence time analysis. Although $k_{\mathrm{SB}\rightarrow\mathrm{FD}}$ have different values from k_{-1}^{app} obtained from residence time analysis (Supplementary Figure 40c), it shows the same trend: it increases with increasing cellular concentrations of free-diffusing proteins, thus validating our conclusion of concentration-dependent unbinding kinetics from recognition sites.

3) Transitions between SB and NB states (blue solid and dash curves in Supplementary Figure 40d) have the slowest, which is consistent with our approximation that interconversion between NB and SB states are negligible kinetically in the residence time analysis in Section 14 (reference Supplementary Figure 29).

There are also problems with the vbSPT analysis results. (1) The 3 diffusion constants from vbSPT vary across different protein concentrations (Supplementary Figure 40a), even though they are not expected to depend on the cellular protein concentrations. The software, to our knowledge, does not allow globally sharing the diffusion constants across multiple data sets. (2) For the apparent binding rates (i.e., for FD→SB and FD→NB transitions; red and green dash curves), they do not show clear dependence on the free-diffusing protein concentration (Supplementary Figure 40d), even though they are expected to be so. This discrepancy is probably related to that vbSPT does not produce consistent diffusion constants across different data sets at different cellular protein concentrations.

17. Correcting for the effects of cellular degradation of $\mathrm{ZntR}^{\mathrm{mE}}$

The mEos3.2-tagged ZntR shows significant degradation in the cell (Supplementary Figure 6 and Section 3.3.2), as expected from previous studies of ZntR degradation in *E. coli* [10]. In this section, we first discuss the possible consequences of this cellular degradation and the corresponding corrections that

could be taken in the data analysis for the extraction of correct protein concentrations in the cell (i.e., $[P]_{cell}$, $[P]_{FD}$, $[P]_{NB}$, and $[P]_{SB}$). We then discuss how this complication may affect the extraction of ZntR's kinetic parameters for unbinding from its recognition sites. The conclusion here is that for ZntR, the apparent unbinding rate constant k_{-1}^{app} from recognition sites is unaffected by this degradation, but k_f, the facilitated unbinding rate constant from recognition sites, is likely underestimated and k_{-1}, the spontaneous unbinding rate constant from recognition sites, is likely overestimated.

17.1 Possible consequences of ZntR degradation/cleavage

To successfully extract k_{-1} and k_f in our data analysis (Section 14), we need to know the fractional populations of the FD, NB, and SB states, and the cellular protein concentration. Here we discuss how the $ZntR^{mE}$ degradation in the cell may affect the quantification of these two factors and the corrections.

According to our Western blot results (Supplementary Figure 6 and Section 3.3.2), the degradation of $ZntR^{mE}$ involves cleavage at the linker region between ZntR and mEos3.2, and the Western blot shows the intact $ZntR^{mE}$ and the cleaved mEos3.2, both of which were detected by the C-terminal FLAG tag. For the cleaved mEos3.2 tag, the corresponding N-terminal ZntR portion is not detectable in the blot, and could be fully degraded and nonfunctional or stay as a whole functional ZntR protein; the former is more likely, as Pruteanu *et al.* showed that ZntR itself is significantly degraded in the cell [10]. The degradation/cleavage of $ZntR^{mE}$ can have a few possible consequences:

(1) *The cleaved N-terminal ZntR portion is degraded and nonfunctional, and the cleaved mEos3.2 tag is also nonfluorescent.*

Under this possibility, any fragments coming from $ZntR^{mE}$ do not interact with DNA, nor contribute to our measured tracking results. No corrections are needed in analyzing our measured tracking results.

(2) *The cleaved N-terminal ZntR portion is still a functional protein, and the cleaved mEos3.2 tag is also fluorescent.*

Under this possibility, the cleaved ZntR can still interact with DNA, just like the way a non-photoconverted $ZntR^{mE}$ interacting with DNA. The corresponding cleaved mEos3.2 tag is still fluorescent (mE^{FL}) and observable and contributes to the measurements of fractional population and cellular protein concentration. Since the cleaved mEos3.2 and ZntR have one to one correspondence, the measurement of cellular protein concentration is still correct (i.e., true cellular concentration $[\mathrm{ZntR}]_{cell}^{T}$ is equal to the concentration sum of observed $ZntR^{mE}$ and cleaved mE^{FL}, $[\mathrm{ZntR}]_{cell}^{T} = [\mathrm{ZntR}^{mE}] + [\mathrm{mE}^{FL}]$), but the freely diffusing cleaved mEos3.2 tag will result in an overestimated fractional population A_{FD} of freely-diffusing ZntR. If the percent cleavage is p (i.e., $\frac{A_{FD}^{c}}{A_{FD}^{T} + A_{FD}^{c} + A_{NB} + A_{SB}} = p$, where A_{FD}^{c} is the fractional population of cleaved ZntR, A_{FD}^{T} is the true fractional population of freely diffusing ZntR; and $A_{FD}^{T} + A_{FD}^{c} = A_{FD}$, and $A_{FD}^{T} + A_{FD}^{c} + A_{NB} + A_{SB} = 1$), the true fractional population A_{FD}^{T} of freely diffusing $ZntR^{mE}$ can be obtained via Supplementary Equation 37.

$$A_{FD}^{T} = 1 - p - A_{NB} - A_{SB} \tag{37}$$

We can then calculate the correct $[P]_{FD}$, $[P]_{NB}$, and $[P]_{SB}$ via Supplementary Equation 38 to 40 respectively for kinetic and thermodynamic parameter analysis.

$$\left[\text{ZntR}\right]_{\text{FD}} = \left[\text{ZntR}\right]_{\text{cell}}^{\text{T}} \times A_{\text{FD}}^{\text{T}} = \left(\left[\text{ZntR}^{\text{mE}}\right] + \left[\text{mE}^{\text{FL}}\right]\right) \times A_{\text{FD}}^{\text{T}} \quad 38$$

$$\left[\text{ZntR}\right]_{\text{NB}} = \left[\text{ZntR}\right]_{\text{cell}}^{\text{T}} \times A_{\text{NB}} = \left(\left[\text{ZntR}^{\text{mE}}\right] + \left[\text{mE}^{\text{FL}}\right]\right) \times A_{\text{NB}} \quad 39$$

$$\left[\text{ZntR}\right]_{\text{SB}} = \left[\text{ZntR}\right]_{\text{cell}}^{\text{T}} \times A_{\text{SB}} = \left(\left[\text{ZntR}^{\text{mE}}\right] + \left[\text{mE}^{\text{FL}}\right]\right) \times A_{\text{SB}} \quad 40$$

(3) *Only the cleaved N-terminal ZntR is functional, whereas the cleaved mEos3.2 tag is not fluorescent.*

Similar to possibility #2 above, the cleaved ZntR still contributes to DNA binding while the cleaved mEos3.2 is not fluorescent or detectable. This means that the estimation of extracted fractional population does represent the protein-DNA interaction status of ZntR (i.e., A_{FD}^{T} = A_{FD}), but the overall cellular concentration is underestimated. The true protein concentration will be the concentrations sum of observed ZntR^{mE} and non-fluorescent mEos3.2, mE^{NF}:

$$\left[\text{ZntR}\right]_{\text{cell}}^{\text{T}} = \left[\text{ZntR}^{\text{mE}}\right] + \left[\text{mE}^{\text{NF}}\right] \quad 41$$

Note that $[\text{mE}^{\text{NF}}]$ can be expressed in terms of p and $\left[\text{ZntR}\right]_{\text{cell}}^{\text{T}}$.

$$p = \frac{\left[\text{mE}^{\text{NF}}\right]}{\left[\text{ZntR}\right]_{\text{cell}}^{\text{T}}} \Rightarrow \left[\text{mE}^{\text{NF}}\right] = p\left[\text{ZntR}\right]_{\text{cell}}^{\text{T}} \quad 42$$

Substitute $\left[\text{mE}^{\text{NF}}\right] = p\left[\text{ZntR}\right]_{\text{cell}}^{\text{T}}$ in Supplementary Equation 41 and rearrange the equation we get

$$\left[\text{ZntR}\right]_{\text{cell}}^{\text{T}} = \left(\frac{1}{1-p}\right)\left[\text{ZntR}^{\text{mE}}\right] \quad 43$$

We can then calculate the correct $[\text{P}]_{\text{FD}}$, $[\text{P}]_{\text{NB}}$, and $[\text{P}]_{\text{SB}}$ via Supplementary Equation 44 to 46 respectively for kinetic and thermodynamic parameter analysis

$$\left[\text{ZntR}\right]_{\text{FD}} = \left[\text{ZntR}\right]_{\text{cell}}^{\text{T}} \times A_{\text{FD}} = \left[\text{ZntR}\right]_{\text{cell}}^{\text{T}} \times A_{\text{FD}}^{\text{T}} \quad 44$$

$$\left[\text{ZntR}\right]_{\text{NB}} = \left[\text{ZntR}\right]_{\text{cell}}^{\text{T}} \times A_{\text{NB}} \quad 45$$

$$\left[\text{ZntR}\right]_{\text{SB}} = \left[\text{ZntR}\right]_{\text{cell}}^{\text{T}} \times A_{\text{SB}} \quad 46$$

.

(4) *Only mEos3.2 is fluorescent, whereas the cleaved N-terminal ZntR portion is degraded and nonfunctional.*

Under this possibility, the cleaved ZntR does not contribute to the protein-DNA interactions, and the detectable cleaved mEos3.2 results in an overestimated cellular ZntR concentration and the fractional population of FD proteins. The true protein concentration will be the concentration of ZntR^{mE}, which is equal to the overall observable fluorescence (from both ZntR^{mE} and the cleaved mEos3.2, mE^{FL}) minus the fluorescence of mE^{FL}.

$$\left[\text{ZntR}\right]_{\text{cell}}^{\text{T}} = \left(\left[\text{ZntR}^{\text{mE}}\right] + \left[\text{mE}^{\text{FL}}\right]\right) - \left[\text{mE}^{\text{FL}}\right] \quad 47$$

Note that $[\text{mE}^{\text{FL}}]$ can be expressed in terms of p and $[\text{ZntR}^{\text{mE}}]$.

$$p = \frac{\left[\text{mE}^{\text{FL}}\right]}{\left[\text{ZntR}^{\text{mE}}\right] + \left[\text{mE}^{\text{FL}}\right]} \Rightarrow \left[\text{mE}^{\text{FL}}\right] = p\left(\left[\text{ZntR}^{\text{mE}}\right] + \left[\text{mE}^{\text{FL}}\right]\right) \quad 48$$

Substitute $[\text{mE}^{\text{FL}}] = p([\text{ZntR}^{\text{mE}}]+[\text{mE}^{\text{FL}}])$ in Supplementary Equation 47, rearrange the equation we got

$$[\mathrm{ZntR}]^{\mathrm{T}}_{\mathrm{cell}} = (1 - p)\left(\left[\mathrm{ZntR}^{\mathrm{mE}}\right] + [\mathrm{mE}^{\mathrm{FL}}]\right) \quad 49$$

With the percent cleavage is p, we can estimate the $A^{\mathrm{T}}_{\mathrm{FD}}$ via Supplementary Equation 37) and estimate the correct cellular ZntR concentration with Supplementary Equation 49).

We can then calculate the correct $[\mathrm{P}]_{\mathrm{FD}}$, $[\mathrm{P}]_{\mathrm{NB}}$, and $[\mathrm{P}]_{\mathrm{SB}}$ via Supplementary Equation 50-52 respectively for kinetic and thermodynamic parameter analysis

$$[\mathrm{ZntR}]_{\mathrm{FD}} = [\mathrm{ZntR}]^{\mathrm{T}}_{\mathrm{cell}} \times \frac{A^{\mathrm{T}}_{\mathrm{FD}}}{A^{\mathrm{T}}_{\mathrm{FD}} + A_{\mathrm{NB}} + A_{\mathrm{SB}}} \quad 50$$

$$[\mathrm{ZntR}]_{\mathrm{NB}} = [\mathrm{ZntR}]^{\mathrm{T}}_{\mathrm{cell}} \times \frac{A_{\mathrm{NB}}}{A^{\mathrm{T}}_{\mathrm{FD}} + A_{\mathrm{NB}} + A_{\mathrm{SB}}} \quad 51$$

$$[\mathrm{ZntR}]_{\mathrm{SB}} = [\mathrm{ZntR}]^{\mathrm{T}}_{\mathrm{cell}} \times \frac{A_{\mathrm{SB}}}{A^{\mathrm{T}}_{\mathrm{FD}} + A_{\mathrm{NB}} + A_{\mathrm{SB}}} \quad 52$$

17.2 Western blot results likely provide an upper limit for ZntR^{mE}'s percent degradation in the cells used in single-molecule imaging

The faster degradation rate of ZntR than mEos3.2 (Section 3.3.3) suggests that scenario #4 in Section 17.1 is the most likely case. The Western blot of ZntR^{mE}-FLAG under the similar growth and induction conditions (i.e., 30 min in M9 medium is the longest induction time for imaging condition) suggests 10-70% of ZntR is degraded in the cell (Section 3.3.2). When using $p > 12.1\%$ (which is the minimum value of A_{FD} of ZntR^{mE} variants, Supplementary Table 5) to calculate $A^{\mathrm{T}}_{\mathrm{FD}}$ via Supplementary Equation 37 at low concentration, we found the $A^{\mathrm{T}}_{\mathrm{FD}}$ becomes negative, which is unrealistic. This suggests that the 41 kDa fragment is most likely still functional. Alternatively, due to the lengthy processing time of Western blot, ZntR^{mE} may be further degraded during the procedures, resulting in a larger $A^{\mathrm{c}}_{\mathrm{FD}}$ (= p). Therefore, Western blot results likely represent an upper limit for the percent degradation of ZntR^{mE}.

17.3 Degradation of ZntR^{mE} will not affect k^{app}_{-1}, but k_f is likely underestimated while k_{-1} is overestimated

Here we use $\mathrm{ZntR}^{\mathrm{mE}}_{\mathrm{apo}}$ as an example to qualitatively consider the effect of its cellular cleavage on the extracted kinetic and thermodynamic parameters about its interactions with DNA in the cell. Again, we assume the possibility #4 above, which is the most likely case.

(1) *k^{app}_{-1} from analyzing the residence time distribution would stay the same.*

With the cleaved-off mEos3.2 tag in the cell, it will be included in the FD state, as a freely diffusing mEos3.2 is not differentiable from a freely diffusing ZntR^{mE} under our imaging conditions at time lapse T_{tl} = 60 ms. The A_{FD} from the CDF fitting thus includes this population of cleaved mEos3.2. This presence of mEos3.2 will not affect our extraction of k^{app}_{-1} from analyzing the residence time distribution, however, because this cleaved mEos3.2 behaves the same way as the freely diffusing ZntR^{mE} and its contribution to the residence time distribution is deconvoluted out by the FD term in Supplementary Equation 19) and 21) as described in Section 14.1 and 14.2.

(2) *k_f from analyzing k^{app}_{-1} vs. $[P]_{FD}$ is likely an underestimate while k_{-1} is likely an overestimate, considering the presence of free mEos3.2 from ZntR^{mE} degradation.*

To determine k_{-1} and k_f, we need to plot k_{-1}^{app} against $[P]_{FD}$. Here, because of the cleaved mEos3.2 tag, the uncorrected $[P]_{FD}$ is an overestimate of the actual $[P]_{FD}$ in the cell. In other words, without correction, for the linear dependence of k_{-1}^{app} vs. $[P]_{FD}$, the slope k_f is likely an underestimate of the actual value and k_{-1} is likely overestimated.

18. Additional results and discussions of extracted kinetic and thermodynamics parameters, and functional implications of the facilitated unbinding of CueR or ZntR in cells

Here we summarize all the kinetic and thermodynamic parameters about CueR or ZntR interactions with DNA *in vivo*, extracted from the kinetic model, as well as those of corresponding control experiments. We also discuss the potential functional implications of this quantitative information.

18.1 Summary of extracted kinetic and thermodynamic parameters

Following analysis described in Section 14, we extracted the kinetic and thermodynamic parameters for all $CueR^{mE}$ and $ZntR^{mE}$ variants and summarized in Supplementary Table 7 and Supplementary Table 8. The kinetic parameters are defined in Supplementary Figure 29. *It should be noted that both CueR and ZntR have many recognition sites in the E. coli genome (Section 22), so the kinetic parameters measured here represent averaged properties over all these possible sites.*

For $CueR^{mE}$, the variants include:

1) Strains expressing $CueR^{mE}_{apo}$ imaged in metal-depleted or regular M9 medium; referred to as $CueR^{mE}_{apo}$ in the table below.

2) Strains expressing $CueR^{mE}$, imaged in the presence of 100 μM Cu^{2+} in the M9 medium, where the cellular $CueR^{mE}$ is significantly metallated; referred to as $CueR^{mE}_{Cu}$ in the table below.

3) Strains expressing $CueR^{mE}$, imaged in the metal-depleted medium; referred to as $CueR^{mE}$ in the table below.

4) Strain expressing $CueR^{mE}$ and contains the promoter knockout ΔP_{cueO}; imaged in metal-depleted medium; referred to as $CueR^{mE} + \Delta P_{cueO}$ in the table below.

For $ZntR^{mE}$, the variants parallel those of $CueR^{mE}$, except that the promoter knockout is ΔP_{zntA} and the metal stress is with 100 μM Zn^{2+}. Also, the kinetic parameters here were obtained without correcting for the $ZntR^{mE}$ degradation in the cell, and thus they are presented here as just estimates and for qualitative discussions.

18.2 Comparisons of kinetic/thermodynamic parameters of $CueR^{mE}_{apo}$, $CueR^{mE}_{Cu}$, and $CueR^{mE}$, and of $ZntR^{mE}_{apo}$, $ZntR^{mE}_{Zn}$, and $ZntR^{mE}$ for interactions with DNA in vivo, and their functional significance

First, it is worth noting that for $CueR^{mE}_{apo}$ and $ZntR^{mE}_{apo}$, their Cu/Zn-binding cysteines were mutated to serines to make them permanently apo and constitutive repressors. For $CueR^{mE}_{Cu}$ and $ZntR^{mE}_{Zn}$, the cells were stressed with metal concentrations that cause maximal regulon induction [7,9], where the cellular CueR or ZntR are significantly metallated (i.e., in holo, the activator, form,) but may still contain a fraction of un-metallated apo forms. For $CueR^{mE}$ and $ZntR^{mE}$, they most likely exist in a mixture of apo and holo forms; the relative populations of apo vs. holo forms depend on the (residual) cellular Cu/Zn levels and their respective affinity to Cu/Zn, even though the cells were grown in Cu/Zn-depleted media.

On the functional side, CueR senses cytoplasmic Cu^{+} (down to 10^{-21} M) [6] to activate Cu efflux genes like *copA*, an inner-membrane efflux pump, and *cueO*, a periplasmic multicopper oxidase [9,49,62,63]. ZntR responds to Zn^{2+} to activate the transcription of the Zn-efflux ATPase ZntA, the only identified gene regulated by ZntR in *E. coli* [64,65] prior to our study (see Section 22.1).

(1) Binding affinities, binding rate constants, and spontaneous unbinding rate constants

For all variants of both CueR and ZntR, their determined binding affinities to the specific recognition sites (K_{D1}, tens of nM) are tens-to-hundreds times stronger than those to the nonspecific sites (K_{D2}, hundreds of nM), as expected; their values are also all consistent with literature values determined *in vitro* [10,49,50], and they do not differ much in the presence or absence of metal stress, which is also consistent with reported or estimated DNA binding affinities for apo and holo CueR as described in Section 19.

For CueR, the spontaneous unbinding rate constants from the specific (k_{-1}, 5.7-12.3 s^{-1}) and nonspecific sites (k_{-2}, 6.0-11.5 s^{-1}) are similar or slightly larger compared with reported values (specific: 0.4-1.1 s^{-1}; nonspecific sites: 5.9 s^{-1}) from our *in vitro* study [50]. The difference could come from cellular environments vs. *in vitro* solution conditions. Interestingly, kinetic basis for the affinity differences between specific and nonspecific sites mainly come from differences in binding (i.e., k_1 vs. k_2) rather than unbinding (i.e., k_{-1} vs. k_{-2}) — CueR and ZntR bind to their respective recognition sites much faster than to nonspecific sites.

(2) Facilitated unbinding rate constants from recognition sites, and their functional significance for more facile switching between transcription deactivation and activation

For all variants of CueR and ZntR, their apparent unbinding rate constant from specific recognition sites k_{-1}^{app} show a clear dependence on the cellular free protein concentration. This concentration-dependent unbinding kinetics was also observed in our *in vitro* experiments on CueR [50] where it was determined to result from two kinetic pathways: an assisted dissociation pathway (i.e., k_{ad}) and a direct substitution pathway (i.e., k_{ds}) as shown in Supplementary Figure 41a. In the assisted dissociation process, which is more dominant for apo-CueR, a protein from solution helps carry away the incumbent one bound at the recognition site on DNA; in the direct substitution process, which is more dominant for holo-CueR, a protein coming from the surrounding solution directly replaces the incumbent protein on DNA. *Both of the two processes only occur to CueR bound at specific sites, but not to nonspecifically bound CueR* [50].

We previously proposed a unified mechanism for these two pathways, evoking a $protein_2$−DNA ternary complex (i.e. $CueR_2$−DNA) as a common intermediate[66] (here CueR refers to the functional homodimer; Supplementary Figure 41b), which was supported experimentally by a related ternary complex in CueR interactions with an engineered DNA Holliday junction [66,67]. In this mechanism, CueR is a homodimer and binds to one-half of the dyad symmetric sequence with each DNA-binding domain. Under thermal fluctuation, one of the DNA-binding domains could detach from DNA, allowing another CueR molecule from the solution to bind to the vacant half dyad sequence and leading to a $CueR_2$−DNA ternary complex. The ternary complex could then proceed in either of the two pathways: (1) one of the two CueR falls off, where 50% of the chance would result in a direct substitution; (2) both proteins fall off, resulting in an assisted dissociation. Similar mechanisms were also recently proposed by Marko and coworkers in rationalizing their *in vitro* observation of concentration-dependent unbinding rate of non-specific DNA binding proteins from *E. coli* (Fis and HU) and yeast (NHP6A) [68-70], and by Greene in rationalizing the *in vitro* concentration-dependent exchange of replication protein A (RPA) on single-stranded DNA [71]. An essential common feature of all these mechanisms are the multivalent contacts between the protein and DNA, which allows for two protein molecules to each have partial contact with DNA in forming ternary protein-DNA complexes as intermediates.

In our measurements of CueR or ZntR concentration-dependent unbinding from specific recognition sites in cells, we could not differentiate the assisted dissociation and direct substitution processes, so our extracted rate constant k_f includes both of them (i.e., $k_f = k_{ad} + k_{ds}$). But based on our *in vitro* work of CueR, k_f (~32 $\mu M^{-1}s^{-1}$) for $CueR^{mE}_{apo}$ should be dominated by k_{ad}. On the other hand, k_f of $CueR^{mE}_{Cu}$ has significant contribution from k_{ds}; its value (~55 $\mu M^{-1}s^{-1}$) is significantly different from and ~2 times larger than that of $CueR^{mE}_{apo}$, indicating we are indeed measuring the kinetics of the holo-form of CueR in the cell. Moreover, the k_f for $CueR^{mE}$, where the cells were grown in Cu-depleted medium, is comparable to that for $CueR^{mE}_{Cu}$, suggesting that even in Cu-depleted medium, the cellular CueR is already significantly metallated, likely due to the residual Cu in the cell and the extremely strong affinity of CueR to Cu^+ (10^{-21} M affinity) [6]. Therefore, under Cu-stress conditions, k_f of $CueR^{mE}_{Cu}$ reflects dominantly the holo-form and, based on our *in vitro* work, is dominated by the k_{ds}, the direct substitution process. The 2× larger k_f for $CueR^{mE}_{Cu}$ than for k_f of $CueR^{mE}_{apo}$, therefore, not only serves as a strong evidence that we are indeed measuring the contribution of the direct substitution pathway but also implies the direct substitution is even more efficient compared with the assisted dissociation pathway in removing an incumbent protein at a recognition site. Similar conclusion could be drawn from the results of $ZntR^{mE}_{apo}$, $ZntR^{mE}$ and $ZntR^{mE}_{Zn}$, although the differences in their k_f's are not as large as those for CueR variants.

The concentration-dependent unbinding kinetics of CueR and ZntR can help them deactivate or activate transcription more facilely in the cell. CueR and ZntR, as well as other MerR-family regulators, recognize specific dyad-symmetric sequences located within σ^{70}-dependent suboptimal promoters [72-76]; these promoters have elongated ~19 base pair spacers between the −10 and −35 elements as compared with the optimal 17±1 base pair spacer in optimal promoters. As a result, these suboptimal promoters represent a weakly repressed state for transcription; the apo-regulator (e.g., apo-CueR) binds strongly and acts as a repressor to strongly repress the transcription, whereas the holo-regulator (e.g., holo-CueR) binds also strongly and acts as an activator, both via distorting the DNA structure at the promoter region [77-83].

For transcription deactivation at these suboptimal promoters, the promoter-bound holo-regulator, which is the activator, needs to be replaced by an apo-regulator, which is the repressor. This could be via either metal dissociation from the promoter-bound holo-regulator or holo-regulator protein unbinding, which leads to a weakly repressed state and subsequent apo-regulator rebinding leading to strongly repressed state. Metal dissociation, although plausible in principle, is the least likely pathway because both CueR and ZntR bind their respective metals extremely tightly (CueR's Cu^{1+} affinity is 10^{-21} M [6], while ZntR's Zn^{2+} affinity is about 10^{-15} M [65]). Although thiol ligand exchange can possibly facilitate metal removal from the binding sites as observed for copper chaperones [84], no evidence exists that CueR or ZntR can undergo similarly facile metal ligand exchange reactions. Therefore, transcription deactivation likely will involve unbinding of a promoter-bound holo-activator, instead of waiting for metal dissociation from the holo-protein. With the k_{-1} and k_f from Supplementary Table 7 and Supplementary Table 8, we can calculate the overall unbinding rate constant with facilitated pathways (i.e., $k_{-1}+k_f[P]_{FD}$) across different protein concentrations in the cell. For example, for holo-CueR under just chromosomal expression, the cellular protein concentration $[CueR]_{cell}$ varies from about 17 to 240 nM (Supplementary Figure 17c). Using A_{FD} information from CDF analysis (Section 11 and Supplementary Table 5) at similar cellular protein concentrations (<15% and ~25%, respectively), we can estimate that the free protein concentration $[CueR]_{FD}$ ranges from < 2.5 to ~60 nM. With k_f = 55 $\mu M^{-1}s^{-1}$, k_{-1} = 4.6 s^{-1}, and $[CueR]_{FD}$ ranging from 2.5 to 60 nM with *just chromosomal expression*, the overall apparent unbinding rate constant (i.e., $k_{-1}+k_f[P]_{FD}$) ranges from ~4.7 to ~7.9 s^{-1}, a ~70% difference. *Therefore, the facilitated unbinding mechanism can help deactivate transcription of metal resistance genes more facilely.*

On the other hand, activating transcription will also likely involve unbinding of a promoter-bound apo-repressor, rather than waiting for the promoter-bound apo-repressor to get metallated. For a single apo-regulator bound at a recognition site within a specific promoter, in order to get metallated, it faces the competition of tens to hundreds of copies of all other apo–regulators in the cytopalsm and at other binding sites [85,86], which all have comparable affinities to the metal (Section 19). Rather than waiting for all other apo-regulators to get metallated before this particular apo-regulator gets the metal, the promoter-bound apo-regulator can unbind, vacating the promoter site for binding by a holo-regulator from the cytoplasm. Here the facilitated unbinding of apo-regulator would be helpful. For example, for apo-CueR, with its $k_f = 32\ \mu M^{-1}s^{-1}$, $k_{-1} = 8.2\ s^{-1}$, and $[CueR]_{FD}$ ranging from about 2 to 50 nM *with just chromosomal expression* ($[CueR]_{cell}$ ~ 14 to 185 nM, with A_{FD} of <15% and ~25%, Supplementary Figure 17b, Section 7), its overall unbinding rate constant from the recognition sites can be up to ~9.8 s^{-1}, ~20% faster than its spontaneous unbinding at 8.2 s^{-1}. *Therefore, the facilitated unbinding mechanism can help activate transcription of metal resistance genes more facilely.*

(3) Effective number of specific recognition sites (N_{SB}) and nonspecific binding sites (N_{NB})

Our analysis also gives the *effective* number of specific DNA recognition sites (N_{SB}) and nonspecific DNA binding sites (N_{NB}) in the cell for CueR and ZntR (Supplementary Table 7 and Supplementary Table 8). For both proteins, N_{SB} is much smaller than N_{NB}, consistent with expectation. For CueR, its N_{SB} is in the same magnitude (~10^2) as what was reported (~200 CueR boxes) in the literature [30]. For ZntR, its N_{SB} is around 10^1-10^2, although there was merely one identified recognition site in the promoter of the *zntA* gene (i.e., P_{zntA}) [64,65,81]. This finding prompted to us to search the *E. coli* genome for possible ZntR recognition sites and we indeed find many candidates (see Section 22).

18.3 Results from promoter knock out controls: ΔPcueO and ΔPzntA

As further controls, we knocked out the *cueO* promoter (ΔP_{cueO}), one of the many sites CueR recognizes and the *zntA* promoter (ΔP_{zntA}), the only site that ZntR was known to recognize in *E. coli*. We expected to see little change in the fractional population of the SB state in the ΔP_{cueO} strain for CueR studies, but significant change in the fractional population of the SB state in the ΔP_{zntA} strain for ZntR studies if *zntA* promoter region were the only ZntR recognition site.

After extracting the fractional populations from CDF analysis, we found, at a same intracellular regulator concentration (e.g., ~100 nM), the A_{SB} for CueR^{mE} in the ΔP_{cueO} strain did not show observable decrease compared with the wild type strain, consistent with expectation (Fig. 2c in the main text). The A_{SB} for ZntR^{mE} in the ΔP_{zntA} strain decreased by ~34% compared with that in the wild-type strain (Fig. 2c in the main text). The larger decrease of A_{SB} from ΔP_{zntA} indicates that fewer effective ZntR recognition sites exist in the chromosome, which is consistent with the smaller N_{SB} for ZntR compared with the N_{SB} for CueR (Supplementary Table 7 and Supplementary Table 8). Furthermore, even though a significant decrease of A_{SB} for ZntR was observed in the ΔP_{zntA} strain, A_{SB} was not abolished, suggesting there are other ZntR recognition sites besides that in P_{zntA} in the *E. coli* genome.

To further support there are other recognition sites for ZntR in the ΔP_{zntA} strain, short time chromosome MSD analysis was applied to ZntR^{mE} in the ΔP_{zntA} strain (see Section 12.2). The results gave the same chromosome diffusion constant (~0.037 $\mu m^2 s^{-1}$) as that ZntR^{mE} in the wild-type strain (Supplementary Figure 26, Section 12.2), which further supports the existence of other ZntR recognition sites.

This interesting finding prompted us to search for other ZntR recognition sites in the *E. coli* genome. Searching with the consensus sequence, ACTNTGNAGTCGACTCCANAGT, indeed generated many other hits (see details in Section 22).

18.4 Validation of quasi-static system approximation for using a linear combination of displacement CDFs/PDFs and a linear combination of residence time distributions

In analyzing the experimental distribution of displacement *r*, we used a linear combination of CDFs (and PDFs) to determine the number of diffusion states and their fractional populations (Section 11.2), as similarly done in the literature [18,37-42]. We also used a linear combination of terms of different states to analyze the residence time distribution (Section 14.1, Supplementary Equation 21). This linear combination approach assumes a quasi-static system, i.e., interconversion between the different states is slower than the experimental time resolution (60 ms for T_{tl} in our experiments).

With extracted kinetic parameter, we can validate this approximation of quasi-static system. Using $\mathrm{CueR}^{\mathrm{mE}}_{\mathrm{apo}}$ as example, at the highest cellular protein concentration $[\mathrm{CueR}^{\mathrm{mE}}_{\mathrm{apo}}]_{\mathrm{cell}}$ of ~900 nM (corresponding to $[\mathrm{CueR}^{\mathrm{mE}}_{\mathrm{apo}}]_{\mathrm{FD}}$ ~260 nM) where we performed 3-state analysis and where all binding/unbinding steps are fastest, we can estimate the apparent interconversion rates between the states. The computed apparent unbinding rates of SB (k_{-1}^{app}) and NB (k_{-2}) are 16.8 and 2.5 s^{-1} (corresponding to the timescales of ~60 and 400 ms). The apparent binding rates of SB ($k_1[\mathrm{D}]_{\mathrm{SB}}$) and NB ($k_2[\mathrm{D}]_{\mathrm{NB}}$) are ~5.3 and 5.4 s^{-1} (corresponding to the timescales of 189 and 185 ms). Comparing with these timescales, our imaging time lapse of 60 ms is comparable or significantly faster, which validates the quasi-static system approximation.

19. Metal-binding affinities of DNA-bound and free CueR or ZntR are comparable

The metal binding affinities of DNA-bound and free ZntR have been reported by Hitomi *et al*. to be 0.63 and 1.58 fM respectively (no error bars were reported there) [65]. Therefore, regardless of ZntR being free (e.g., in the cytoplasm) or bound to a recognition site, its affinity to Zn^{2+} is comparable or maximally differ by a factor of ~2.

However, the parallel information for CueR is lacking. Here we estimate the relative metal binding affinities of DNA-bound and free CueR via a thermodynamic analysis using the equilibrium scheme as shown in Supplementary Figure 42, which was used to estimate the allosteric coupling free energy of Zn^{2+} to the metalloregulator CzrA by Giedroc *et al*. [87,88] In short, to form a metal-bound protein-DNA complex, it can go through the following two pathways:

(1) An apo protein ($\mathrm{P}^{\mathrm{apo}}$) binds first to the metal with the dissociation constant $K_{\mathrm{D,M}}^{\mathrm{P}}$ and forms the holo protein ($\mathrm{P}^{\mathrm{holo}}$), which then binds to DNA to from $\mathrm{P}^{\mathrm{holo}}\mathrm{D}$ with the dissociation constant $K_{\mathrm{D,PD}}^{\mathrm{holo}}$.

(2) A $\mathrm{P}^{\mathrm{apo}}$ binds to DNA with the dissociation constant $K_{\mathrm{D,PD}}^{\mathrm{apo}}$ and forms the $\mathrm{P}^{\mathrm{apo}}\mathrm{D}$ complex, which then gets metallated to form $\mathrm{P}^{\mathrm{holo}}\mathrm{D}$ with the dissociation constant $K_{\mathrm{D,M}}^{\mathrm{PD}}$.

With known DNA binding affinities of apo- and holo-proteins obtained from the literature, we can then estimate the relative metal binding affinities of DNA-bound and free regulator via:

$$\frac{K_{\mathrm{D,M}}^{\mathrm{PD}}}{K_{\mathrm{D,M}}^{\mathrm{P}}}=\frac{K_{\mathrm{D,PD}}^{\mathrm{holo}}}{K_{\mathrm{D,PD}}^{\mathrm{apo}}} \tag{S53}$$

The calculated results are summarized in Supplementary Table 9 and do not show clear differences in the metal binding affinity between P^{holo} and $P^{holo}D$, suggesting that the DNA binding does not change the metal binding affinity of regulator significantly. In other words, the DNA-bound CueR and free CueR bind Cu^+ approximately equally well.

Therefore, for either apo CueR or apo ZntR, whether present in the cytoplasm or bound to a DNA site, it binds its cognate metal ion comparably well. And for a particular apo CueR or apo ZntR molecule bound at a promoter site, in order to get metallated, it faces the competition for the metal by the equally capable, but much larger in number, apo-regulators in the cytoplasm and bound at other DNA sites. This concept of competition for metal has been discussed previously by Robinson *et al.*[85,86]

20. Quantifying the extent of chromosome condensation using the average pairwise distance between residence sites

One common way to visualize the spatial distribution of the chromosome in a living *E. coli* cell is by monitoring the spatial distribution of fluorescent-protein-tagged nucleoid-associated proteins such as HU and Fis [12,89,90], which nonspecifically decorate the entire chromosome, or by mapping out the binding sites of chromosome-interacting proteins [91]. In a similar manner, by monitoring the spatial distribution of $\mathrm{CueR^{mE}}$ and $\mathrm{ZntR^{mE}}$ molecules that are bound to chromosome nonspecifically or specifically, we can directly map out the spatial distribution of the chromosome in a cell. Here, in particular, for both CueR and ZntR, their specific recognition sites are scattered across the chromosome randomly (Section 22.3), making the specific binding events also amenable for mapping the spatial distribution of the chromosome in the cell. In this section, we will discuss how we extract the locations of $\mathrm{CueR^{mE}}$ and $\mathrm{ZntR^{mE}}$ molecules that are nonspecifically bound (NB) to chromosome and specifically bound (SB) to recognition sites on the chromosome, so as to quantify the extent of chromosome condensation by using the r_0-thresholded bound state (e.g., Supplementary Figure 16 and Fig.1a and b in the main text) and the average pairwise distance between the protein residence sites on the chromosome.

20.1 Quantify the chromosome condensation using the average pairwise distance $\langle d_{ij}\rangle$ between the residence sites from the r_0-thresholded bound state

Supplementary Figure 43a and b show the fitted relative contributions of FD, NB, and SB states to the r_0-thresholded residence time distribution of $\mathrm{CueR_{apo}^{mE}}$ at low (99 nM) and high (926 nM) cellular protein concentrations. Note each data point within a residence time extracted here also corresponds to a particular spatial location in the cell as demonstrated in Fig.1a and b in the main text.

Based on our kinetic model in Section 14, with r_0 = 220 nm and diffusion constants and fractional populations in Supplementary Table 5for $\mathrm{CueR_{apo}^{mE}}$, we can then estimate the contribution of FD state to the residence time distribution. For protein concentrations probed in this study, when the residence time τ is > 60 ms (i.e., longer than a single imaging frame at T_{tl} = 60 ms), the contribution of FD state is always less than 10% (e.g., 3% and 8.5% for the lowest and highest cellular protein concentrations, respectively; Supplementary Figure 43c and d). In other words, the locations of the r_0 thresholded residence times from the displacement-vs.-time trajectory dominantly report the locations of protein molecules either bound to chromosome non-specifically (i.e., NB state) or specifically (i.e., SB state). Note the contribution from plasmids to the nonspecific binding is negligible (detail see Section 20.5). Using the first locations within

all thresholded residence times, i.e., the residence sites, we can map the spatial distribution of the chromosome within a living *E. coli* cell.

Supplementary Figure 44a and b show examples of such maps for a number of nondividing cells expressing $\mathrm{CueR}^{\mathrm{mE}}_{\mathrm{apo}}$ where the map of each example is presented as scatter (left panel) and 2D histogram (right panel) plots. In the scatter plot, each data point is a residence site determined from *the first location* of one residence time. Two extreme types of spatial distributions of the residence sites are immediately clear among these non-dividing cells: in some cells, the residence sites are spatially confined to a small region in the cell, i.e., clustered together (e.g., Supplementary Figure 44a); in some other cells, the residence sites are almost spread over the entire cell volume (e.g., Supplementary Figure 44b); the latter behavior is more general among all the cells here. The former type suggests that in these cells, the chromosome is spatially confined within a small region in the cell and thus highly condensed in organization. The latter type suggests that in these cells the chromosome is less condensed in organization and spatially it spreads over the cell. In Section 20.2 below, we will use direct fluorescence imaging of dye-stained chromosome to determine the spatial spread of chromosome and validate that the spatial distribution of residence sites can indeed reflect the chromosome organization in the cell.

Supplementary Figure 44c shows the residence site maps and corresponding 2-D histograms for a number of dividing cells expressing $\mathrm{CueR}^{\mathrm{mE}}_{\mathrm{apo}}$. Their residence sites are mostly spatially confined to two distinct regions, each region in one half of the dividing cell. This spatial confinement of the regulator residence sites is consistent with that the dividing cells have two copies of the chromosome and tend to have highly condensed chromosomes, ready for division [92-94].

To quantify the extent of chromosome condensation of each cell, we computed the average pairwise distance (i.e., $\langle d_{ij} \rangle$) of residence sites. d_{ij} is the Euclidean distance between residence sites i and j, as indicated schematically by each point in Supplementary Figure 45a. Cells with more condensed chromosome have more spatially clustered residence sites and are thus expected to have shorter $\langle d_{ij} \rangle$.

Supplementary Figure 45b shows the two-dimensional histograms of cell length (L) and $\langle d_{ij} \rangle$ from many nondividing cells expressing $\mathrm{CueR}^{\mathrm{mE}}_{\mathrm{apo}}$, $\mathrm{CueR}^{\mathrm{mE}}_{\mathrm{Cu}}$, or from dividing cells expressing $\mathrm{CueR}^{\mathrm{mE}}_{\mathrm{apo}}$, as examples. For nondividing cells expressing $\mathrm{CueR}^{\mathrm{mE}}_{\mathrm{apo}}$, $\langle d_{ij} \rangle$ shows a broad distribution from ~0.5 to 1.1 μm with two resolved subpopulations, centered at $\langle d_{ij} \rangle$ ~0.68 and ~0.88 μm; these two subpopulations represent cells with more condensed chromosomes and less condensed chromosomes, respectively (Supplementary Figure 45b upper). And the subpopulation with smaller $\langle d_{ij} \rangle$ tends to be slightly smaller in cell length than those with larger $\langle d_{ij} \rangle$, as expected[95]. For nondividing cells expressing $\mathrm{CueR}^{\mathrm{mE}}_{\mathrm{Cu}}$, $\langle d_{ij} \rangle$ also shows a broad distribution although no clear subpopulations were resolved (Supplementary Figure 45b middle).

For the dividing cells that have two copies of chromosome, each chromosome is highly condensed, as reflected by the spatial maps of residence sites in Supplementary Figure 44c and quantified by the significantly smaller $\langle d_{ij} \rangle$ as compared with those of the nondividing ones (Supplementary Figure 45b lower). (Note that for dividing cells, each cell has two chromosomes located in each half cell envelop with the division septum in-between. We thus calculated $\langle d_{ij} \rangle$ after grouping the residence sites into two separate chromosomes using the division septum as the dividing line.) The highly condensed chromosomes observed here for dividing cells are consistent with well-known knowledge about chromosome organization in dividing cells[92,93,95-98].

20.2 Average pairwise distance ⟨d_{ij}⟩ of residence sites is a proper measure to quantify chromosome condensation, as supported by comparison with direct imaging of dye-stained chromosome

Typically, the extent of chromosome condensation, or compactness, in living *E. coli* cells is measured by staining the chromosome with a dye (e.g., the blue fluorescent dye Hoechst [99,100]) and imaging the dye fluorescence. We therefore performed the chromsome staining and imaging, and compared the results to ⟨d_{ij}⟩, so as to justify that ⟨d_{ij}⟩ is a proper measure to reflect the extent of the chromosome condensation. Nondividing and dividing cells expressing $\mathrm{CueR}_{\mathrm{apo}}^{\mathrm{mE}}$ were again used as examples for this purpose.

Supplementary Figure 46a and b show the fluorescence images of Hoechst-dye-stained nondividing cells. Again, some cells have the fluorescence signal of the Hoechst stain more localized spatially in the cell, reflecting the highly condensed chromosome organization (Supplementary Figure 46a), while others have less condensed chromosome organization, for which the Hoechst-dye fluorescence is spread over the entire cell (Supplementary Figure 46b). In contrast, for dividing cells (Supplementary Figure 46c), the Hoechst-dye fluorescence signal is always spatially confined, reflecting the highly condensed chromosomes, and each cell clearly shows its two copies of chromosome, each on one side of the division septum.

To quantify the chromosome condensation of each cell in the Hoechst-dye-stained fluorescence image, we measured the area that the chromosome occupies in the cell by the area that the fluorescence signal occupies in each cell. For each dividing cell where two chromosomes are clearly observed, we measured the average area of the two chromosomes occupy by dividing the total fluorescence area of the cell by two.

Supplementary Figure 47a shows distribution of the chromosome-occopied area in the cell from many nondividing cells and that from dividing cells, again using cells that express $\mathrm{CueR}_{\mathrm{apo}}^{\mathrm{mE}}$ as examples. On average, the chromosomes in dividing cells clearly occupy smaller areas than those in the nondividing cells, as expected and consistent with the distributions of their ⟨d_{ij}⟩ (Supplementary Figure 45b). Moreover, the nondividing cells show a broader distribution, consistent with that some of them have highly condensed chromosomes while some others have less condensed chromosomes as we observed in the spatial distributions of residence sites (Supplementary Figure 44a and b).

Supplementary Figure 47b shows the 1-D distributions of ⟨d_{ij}⟩ from many nondividing cells and the dividing cells that express $\mathrm{CueR}_{\mathrm{apo}}^{\mathrm{mE}}$ from the top and bottom panels in Supplementary Figure 45b. For the nondividing cells, the two subpopulations are again resolved here: a major peak centered at ⟨d_{ij}⟩ ~0.88 μm and a small side shoulder at ⟨d_{ij}⟩ ~0.68 μm.

We further fitted the distributions of chromosome-occupied area from Hoechst-dye-staining results for the nondividing and dividing cells with two Gaussian distributions (G_{nc} and G_{dc}, respectively, Supplementary Equation 54; Supplementary Figure 47a). These fitted two Gaussian functions were then used to fit the corresponding ⟨d_{ij}⟩ distributions simultaneously with the same set of scaling factors S_x and S_y as shown in Supplementary Equation 55, which scale the distributions along *x*, *y* axes after a *x*-offset (S_{xh}). Strikingly, by using this simple scaling, we could use the same two Gaussian distributions of chromosome-occupied area to describe well the distributions of ⟨d_{ij}⟩ of nondividing cells and dividing cells (Supplementary Figure 47b). *Therefore, ⟨d_{ij}⟩, the average pair-wise distance between the regulator residence sites on chromosome, is a proper measure of the chromosome condensation*. We will further rationalize the validity of ⟨d_{ij}⟩ as a proper measure of chromosome condensation in Section 20.4 and 22.3.

$$G_i = a_i \exp(-\frac{(x-b_i)^2}{2c_i^2}),\ i = \text{nc or dc} \qquad 54$$

$$G_i = S_y a_i \exp(-\frac{(x-(S_{xh}b_i))^2}{2(S_x c_i)^2}),\ i = \text{nc or dc} \qquad 55$$

It is worth noting that the distribution chromosome-occupied area for nondividing cells from Hoechst dye staining did not resolve clearly the cell subpopulation with highly condensed chromosome (Supplementary Figure 47a), which was resolved as a side peak at ~ 0.68 μm in the distribution of $\langle d_{\text{ij}} \rangle$ for nondividing cells (Supplementary Figure 47b). This suggests that $\langle d_{\text{ij}} \rangle$ is a more resolving measure of chromosome condensation than the fluorescence imaging of Hoechst dye staining of the chromosome. This higher resolution of $\langle d_{\text{ij}} \rangle$ is expected, however, as the residence sites were localized to tens of nanometer resolution from SMT, whereas the fluorescence imaging of Hoechst dye staining of the chromosome is only at diffraction-limited resolution (~400 nm for our microscope).

20.3 The subpopulation with highly-condensed chromosome in the $\langle d_{ij} \rangle$ distribution among nondividing cells is still resolved when cell size is considered

Even though the distribution of $\langle d_{\text{ij}} \rangle$ of nondividing cells shows clearly two populations, which represent cells with highly condensed chromosome and less condensed chromosomes (Supplementary Figure 47a), one might be concerned that the subpopulation with highly condensed chromosome may be just an artifact from cells having smaller sizes. We thus further examined the correlation between the cell length and $\langle d_{\text{ij}} \rangle$ for the nondividing cells to address this concern, again using cells that express $\text{CueR}^{\text{mE}}_{\text{apo}}$ as examples. (Note the cell sizes mainly vary in length; their widths are narrowly distributed (Supplementary Figure 13).

Supplementary Figure 48 shows the correlation between each cell's length L and its $\langle d_{\text{ij}} \rangle$ for nondividing cells, in scatter plot and in 2-D histogram format. Two distinct subpopulations were clearly resolved: one with smaller $\langle d_{\text{ij}} \rangle$ (centered at ~0.68 μm) and the other with larger $\langle d_{\text{ij}} \rangle$ (centered at ~0.88 μm), which we have already described in presenting Supplementary Figure 45b. For the cells with less condensed chromosomes (i.e., $\langle d_{\text{ij}} \rangle > 0.77$ μm), the Pearson's correlation coefficient ρ between L and $\langle d_{\text{ij}} \rangle$ is 0.36, indicating a clear positive correlation. This is expected, as when the chromosome is less condensed and spread over the cell, the cell size should constrain the spatial spread of chromosome, resulting in a correlation. In contrast, the correlation between L and $\langle d_{\text{ij}} \rangle$ for cells with highly condensed chromosome (i.e., $\langle d_{\text{ij}} \rangle < 0.77$ μm) is much weaker, $\rho = 0.13$. This supports that the subpopulation with highly condensed chromosomes resolved from $\langle d_{\text{ij}} \rangle$ distribution is not a direct result of the cell size.

20.4 The spatial distribution of residence sites from both nonspecific binding (NB) and specific binding (SB) can reflect the overall chromosome organization in the cell

It is known that chromosome organization can be reflected by mapping the spatial distribution of nonspecific DNA binding sites but not always expected for specific chromosomal recognition sites in the cell. The residence sites (Supplementary Figure 44), which were determined by thresholding the displacement versus time trajectories, include both specific binding sites (i.e., CueR or ZntR recognition sites) and nonspecific binding sites on chromosome. As we have shown in short-time diffusion analysis in Section 12.2, the r_0-thresholded bound state with residence time $\tau \geq 5T_{\text{tl}}$ (T_{tl} = 60 ms) is dominated by the SB state. For the r_0-thresholded bound state with residence time $\tau < 5T_{\text{tl}}$, a significant portion is originated from the NB state. Here we examine the correlation between $\langle d_{\text{ij}} \rangle$ from residence sites associated with

residence time $\tau \geq 5T_{\text{tl}}$ and < $5T_{\text{tl}}$ to test the feasibility of correlating the distribution of SB sites to the overall chromosome compactness.

Supplementary Figure 49 shows the correlation plot between $\langle d_{\text{ij}} \rangle$ for $\tau \geq 5T_{\text{tl}}$ and $< 5T_{\text{tl}}$ for nondividing and dividing cells that express $\text{CueR}_{\text{apo}}^{\text{mE}}$. The correlation coefficients ρ are 0.25 and 0.32 respectively, which suggests that the distribution of SB residence sites is correlated with that NB sites and thus also reflects the overall chromosome organization. This implies that the locations of CueR recognition sites should be scattered somewhat randomly along the chromosome (i.e., not restricted to a certain base range of the chromosome). Using the possible CueR recognition sites identified from sequence analysis (Section 22), we analyzed their locations on the genome map (Section 22.3, Supplementary Figure 56a and c); indeed these recognition sites are located randomly across the chromosome. Same was observed for all the possible ZntR recognition sites (Section 22.3, Supplementary Figure 56b and d). The random locations of CueR or ZntR recognition sites along the chromosome, therefore, rationalize our observation that along with their NB sites, the spatial distribution of their SB sites can also reflect the overall chromosome conformation in the cell.

20.5 Residence sites from nonspecific binding to plasmids are negligible (<7%)

With the pBAD24 plasmid transformed into the cell to achieve higher and variable protein expression, the mEos3.2-tagged CueR or ZntR can interact with both the chromosome and the plasmid in the cell, both of which would contribute to the r_0-thresholded residence times/sites. Since CueR or ZntR can interact specifically and nonspecifically with the chromosome but only nonspecifically with the plasmid (the pBAD24 plasmid does not contain CueR or ZntR recognition sites), residence sites from the NB state should have contributions from both chromosome and plasmids, whereas the residence sites of the SB state are from chromosome only.

Here we quantify the relative contribution of NB residence sites originated from the plasmids to the chromosome by comparing the overall size of each of them as described in the following. Considering the average pBAD24 copy number in a cell is around 55 [101], and with a plasmid size of 5.6 kb, the overall DNA base pairs of these plasmids is ~0.31 Mb. The *E. coli* BW25113 strain we use does not contain other plasmids [1,102]. Compared with the *E. coli* genome size of ~4.6 Mb, the overall size of plasmids is ~ 6.7% of the chromosome. Therefore, the residence sites, and thus the residence times, from the nonspecifically bound (NB) state in our analysis results dominantly from protein interactions with the chromosome, and the contribution from the plasmids is negligible.

20.6 Additional support that the nonspecific binding sites are likely randomly scattered along the chromosome

To support that the nonspecific binding sites of CueR and ZntR are also randomly scattered along the chromosome, we compared $\langle d_{\text{ij}} \rangle$ of cells with highest protein concentrations to that of all cells combined. For cells with highest protein concentrations, their residence sites are dominated by the NB state. If the distribution of $\langle d_{\text{ij}} \rangle$ of this subset of cells is similar to that of all cells combined, for which a significant fraction of the residence sites are from SB sites, which we showed to be randomly scattered along the chromosome (Section 22.3), it will support that the nonspecific sites are also randomly-scattered on the chromosome. We used the cells that express $\text{CueR}_{\text{apo}}^{\text{mE}}$ as an example here. Supplementary Figure 45c, bottom, shows the distribution of $\langle d_{\text{ij}} \rangle$ for those cells with $[\text{P}]_{\text{cell}}$ = 1150 nM, for which A_{NB} = 63.7% and A_{SB} = 6.4%, (i.e., ~90% of the residence sites are NB), in comparison with the distribution of $\langle d_{\text{ij}} \rangle$ among all cells (Supplementary Figure 45b, top). The two distributions are similar, thus supporting that nonspecific protein binding sites also sample the chromosome randomly.

20.7 *Metal stress conditions do not __directly__ affect the extent of chromosome condensation*

In vitro, metal ions can change the condensation, or conformation in general, of DNA directly, for example, via charging screening or direct binding to DNA[103]. Our *in vivo* experiments are different from those *in vitro* studies. In our experiments, we use metal stress conditions (high Cu and Zn concentrations in the cell growth medium) to change the metallation state of CueR or ZntR to be dominantly holo (see Section 4.1 on growth conditions). These metal stress conditions mainly change the fractions of metallated-vs-unmetallated forms of Cu/Zn-binding proteins in the cell, but do not change much the intracellular concentrations of *free* Cu^{+} or Zn^{2+} ions, which are regulated tightly by the Cu and Zn homeostatic machineries. For example, for *E. coli*, its cellular *free* Zn concentration is maintained between 2.0×10^{-16} and 11.5×10^{-16} M;[64] and its free Cu concentration is close to zero in the cell[6]. Therefore, Cu and Zn stress conditions should not *directly* affect DNA condensation *in the cell*, which is supported by that cells under metal-depleted and metal-stressed conditions show similar $\langle d_{ij}\rangle$ distributions (Supplementary Figure 45b).

21. Chromosome-organization-dependent protein unbinding kinetics from recognition sites

With $\langle d_{ij}\rangle$ as a quantitative indication of the extent of chromosome condensation for a cell, we can then relate the chromosome organization to the protein unbinding kinetics from recognition sites. In this section, using the cell strains that express $\mathrm{CueR}^{\mathrm{mE}}_{\mathrm{apo}}$, $\mathrm{ZntR}^{\mathrm{mE}}_{\mathrm{apo}}$, $\mathrm{CueR}^{\mathrm{mE}}_{\mathrm{Cu}}$ and $\mathrm{ZntR}^{\mathrm{mE}}_{\mathrm{Zn}}$ as examples, we will discuss the protein unbinding kinetics as a function of chromosome organization for nondividing cells (i.e., cells that do not show a division septum), dividing cells (i.e., cells that show a clear division septum), and chloramphenicol treated nondividing cells.

21.1 $\mathrm{CueR}^{\mathrm{mE}}_{\mathrm{apo}}$ *and* $\mathrm{ZntR}^{\mathrm{mE}}_{\mathrm{apo}}$*, or* $\mathrm{CueR}^{\mathrm{mE}}_{\mathrm{Cu}}$ *and* $\mathrm{ZntR}^{\mathrm{mE}}_{\mathrm{Zn}}$ *in nondividing cells*

From the $\langle d_{ij}\rangle$ histogram (Fig.3g-h in the main text and Supplementary Figure 45b), we used each cell's $\langle d_{ij}\rangle$ value and grouped nondividing cells into three groups with $\langle d_{ij}\rangle \le 0.68$ µm, $0.68 < \langle d_{ij}\rangle \le 0.88$ µm and $\langle d_{ij}\rangle > 0.88$ µm. This sorting of individual cells into these three subpopulations was done for nondividing cells that express $\mathrm{CueR}^{\mathrm{mE}}_{\mathrm{apo}}$, $\mathrm{ZntR}^{\mathrm{mE}}_{\mathrm{apo}}$, $\mathrm{CueR}^{\mathrm{mE}}_{\mathrm{Cu}}$ or $\mathrm{ZntR}^{\mathrm{mE}}_{\mathrm{Zn}}$. Within each group, we analyzed the residence times and determined k_{-1}^{app} as a function of cellular protein concentration as described in Section 14.2.

Supplementary Figure 50a shows the average residence time $\langle\tau\rangle$ of $\mathrm{CueR}^{\mathrm{mE}}_{\mathrm{apo}}$ as a function of cellular protein concentration for the three groups of cells with different extents of chromosome condensations (the cell-averaged $\langle d_{ij}\rangle$, $\overline{\langle d_{ij}\rangle}$, are 0.56, 0.78, 1.06 µm, respectively). Within each group, their $\langle\tau\rangle$ decreases with increasing cellular protein concentration, and their k_{-1}^{app} shows a consistent linear dependence with increasing cellular concentration of freely diffusing proteins (Supplementary Figure 50b), both consistent with those where we did not sub-divide the cells based on their chromosome organization. This indicates that the concentration-dependent protein unbinding kinetics from recognition sites is operative, regardless of the chromosome organization. But, at any cellular protein concentration, k_{-1}^{app} for cells with more condensed chromosomes (i.e., smaller $\langle d_{ij}\rangle$) is always smaller than those with less condensed chromosomes (i.e., larger $\langle d_{ij}\rangle$), indicating that $\mathrm{CueR}^{\mathrm{mE}}_{\mathrm{apo}}$ unbinding from recognition sites is slower when the chromosome is more condensed. The same trend is observed for $\mathrm{ZntR}^{\mathrm{mE}}_{\mathrm{apo}}$ (Supplementary Figure 50c).

The same analysis is performed as described above for nondividing cells expressing $\mathrm{CueR_{Cu}^{mE}}$ or $\mathrm{ZntR_{Zn}^{mE}}$ and the results on $\langle\tau\rangle$ and k_{-1}^{app} are shown in Supplementary Figure 50d-f. First, their k_{-1}^{app} consistently show the linear dependence with increasing cellular protein concentration, regardless of the chromosome organization. But, surprisingly, a trend opposite to those of $\mathrm{CueR_{apo}^{mE}}$/$\mathrm{ZntR_{apo}^{mE}}$ is observed in comparing k_{-1}^{app} for cells with different chromosome organizations — at any cellular protein concentration, k_{-1}^{app} for cells with more condensed chromosome is larger, indicating that $\mathrm{CueR_{Cu}^{mE}}$ / $\mathrm{ZntR_{Zn}^{mE}}$ unbinding from recognition sites is faster when the chromosome is more condensed.

21.2 Summary of chromosome-organization-dependent kinetic parameters for nondividing cells

Supplementary Table 10 and Supplementary Table 11 summarize the kinetic and thermodynamic parameters for CueR or ZntR–DNA interactions in nondividing living cells as a function of chromosome organization (i.e., for cells with different $\overline{\langle d_{\mathrm{ij}}\rangle}$, the average $\langle d_{\mathrm{ij}}\rangle$ among individual cells in a group). Diffusion constants (i.e., D_{FD}, D_{NB}, and D_{SB}) and unbinding rate constant from nonspecific sites (i.e., k_{-2}) determined from all nondividing cells were shared across different $\overline{\langle d_{\mathrm{ij}}\rangle}$ conditions to extract the corresponding fractional populations at different protein concentrations and k_{-1}^{app}. This was necessary because the simultaneous sorting of cells into different protein concentrations and $\overline{\langle d_{\mathrm{ij}}\rangle}$ significantly lowers the statistics per group and it becomes impractical to determine the diffusion constants and k_{-2} for each group separately. This sharing assumes that diffusion constants and k_{-2} are independent of the extent of chromosome condensation, which is validated by that diffusion constants and k_{-2} for dividing cells and for nondividing cells are the same (i.e., D_{FD}, D_{NB}, and D_{SB} are 4.1 ± 0.2, 0.71 ± 0.03, and 0.044 ± 0.004 $\mu\mathrm{m}^2\mathrm{s}^{-1}$ for dividing cells and 3.7 ± 0.2, 0.70 ± 0.03, and 0.036 ± 0.009 $\mu\mathrm{m}^2\mathrm{s}^{-1}$ for nondividing cells, and k_{-2} = 2.6 ± 0.1 and 2.5 ± 0.1 s^{-1} for dividing and nondividing cells respectively; Supplementary Table 4, Supplementary Table 7, and Section 21.4).

For $\mathrm{CueR_{apo}^{mE}}$and $\mathrm{CueR_{Cu}^{mE}}$, note the error bars for some of the parameters are large because of the limited data points when sub-dividing the individual cells based on both their cellular protein concentrations and their chromosome organizations (i.e., the extent of condensation).

For $\mathrm{ZntR_{apo}^{mE}}$ and $\mathrm{ZntR_{Zn}^{mE}}$ cells, we also collected data from ~200 cells (similar to the CueR case). However, due to lower protein concentrations and thus fewer tracking trajectories, analysis on the concentration dependent k_{-1}^{app} is not always feasible at all $\overline{\langle d_{\mathrm{ij}}\rangle}$ (see Supplementary Figure 50c, f), leading to missing error bars or missing values in Supplementary Table 11.

21.3 Comparisons of chromosome-organization-dependent kinetic and thermodynamic parameters for regulator-DNA interactions in nondividing cells, and their functional significance

Here we compare the kinetic and thermodynamic parameters for cells with different $\overline{\langle d_{\mathrm{ij}}\rangle}$ using $\mathrm{CueR_{apo}^{mE}}$/$\mathrm{CueR_{Cu}^{mE}}$ as examples. The chromosome-organization-dependences of the relevant kinetic and thermodynamic parameters are plotted against $\overline{\langle d_{\mathrm{ij}}\rangle}$ in Supplementary Figure 51.

(1) Binding affinities and binding rate constants

For both $\text{CueR}^{\text{mE}}_{\text{apo}}$ and $\text{CueR}^{\text{mE}}_{\text{Cu}}$, their determined binding affinities (K_{D1}) and binding rate constants (k_1) to the specific recognition sites do not show a clear $\overline{\langle d_{\text{ij}} \rangle}$ dependence within experimental errors (Supplementary Figure 51a, b). In contrast, their binding affinities (K_{D2}) and binding rate constants (k_2) to the non-specific sites show some dependence on the $\overline{\langle d_{\text{ij}} \rangle}$ (Supplementary Figure 51c, d), although the uncertainty is large because of large error bars: for cells with less condensed chromosome, k_2 is smaller (i.e., slower binding) and consistently K_{D2} is larger (i.e., weaker affinity). Therefore, regarding *binding*, its kinetics is perhaps more sensitive to the chromosome organization for the nonspecific sites than for the recognition sites.

(2) Facilitated unbinding rate constants and spontaneous unbinding rate constants from recognition sites

Supplementary Figure 51e shows the k_{f} of $\text{CueR}^{\text{mE}}_{\text{apo}}$ as a function of $\overline{\langle d_{\text{ij}} \rangle}$ (blue lines). The k_{f} increases with increasing $\overline{\langle d_{\text{ij}} \rangle}$. Fitting the data with a linear function $y = S_{k_{\text{f}}}x + I_{k_{\text{f}}}$ gives the slope $S_{k_{\text{f}}}$ and intercept $I_{k_{\text{f}}}$ of 71.3 ± 234.8 $\mu\text{M}^{-1}\ \text{s}^{-1}\mu\text{m}^{-1}$ and −29.4 ± 168.3 $\text{s}^{-1}\mu\text{m}^{-1}$ (i.e., y = 71.3$\overline{\langle d_{\text{ij}} \rangle}$ −29.4), respectively. The same analysis for k_{-1} of $\text{CueR}^{\text{mE}}_{\text{apo}}$ is shown in Supplementary Figure 51f; fitting with $y = S_{k_{-1}}x + I_{k_{-1}}$ gives $S_{k_{-1}}$ and $I_{k_{-1}}$ of 4.8 ± 3.2 $\text{s}^{-1}\mu\text{m}^{-1}$ and 4.7 ± 2.1 s^{-1} (i.e., y = 4.8$\overline{\langle d_{\text{ij}} \rangle}$ + 4.7), respectively. The faster k_{f} and k_{-1} with larger $\overline{\langle d_{\text{ij}} \rangle}$ indicate that for $\text{CueR}^{\text{mE}}_{\text{apo}}$, cells with less condensed chromosome have faster unbinding rate from recognition sites; in other words, cells with more condensed chromosome have slower unbinding from recognition sites.

For $\text{CueR}^{\text{mE}}_{\text{Cu}}$, fitting k_{f} and k_{-1} vs. $\overline{\langle d_{\text{ij}} \rangle}$ (red lines) with linear functions gives the corresponding slopes and intercepts for k_{f} ($S_{k_{\text{f}}}$ of −46.7 ± 99.6 $\mu\text{M}^{-1}\text{s}^{-1}\mu\text{m}^{-1}$, $I_{k_{\text{f}}}$ of 98.6 ± 76.9 $\mu\text{M}^{-1}\text{s}^{-1}$, and y = −46.7$\overline{\langle d_{\text{ij}} \rangle}$ + 98.6) and k_{-1} ($S_{k_{-1}}$of −7.8 ± 7.6 $\text{s}^{-1}\mu\text{m}^{-1}$, $I_{k_{-1}}$of 12.4 ± 5.5 s^{-1}, and y = −7.8$\overline{\langle d_{\text{ij}} \rangle}$ + 12.4). The negative slopes here indicate that for $\text{CueR}^{\text{mE}}_{\text{Cu}}$, cells with less condensed chromosome have slower unbinding rate from recognition sites, opposite to the trend for $\text{CueR}^{\text{mE}}_{\text{apo}}$.

The chromosome-organization dependence of k_{f} and k_{-1} for $\text{CueR}^{\text{mE}}_{\text{apo}}$ and $\text{CueR}^{\text{mE}}_{\text{Cu}}$ are summarized through Supplementary Equation 56)-59).

For $\text{CueR}^{\text{mE}}_{\text{apo}}$:

$$k_{\text{f}}:\quad y = 71.3\overline{\langle d_{\text{ij}} \rangle} - 29.4 \qquad (56)$$

$$k_{-1}:\quad y = 4.8\overline{\langle d_{\text{ij}} \rangle} + 4.7 \qquad (57)$$

$\text{CueR}^{\text{mE}}_{\text{Cu}}$:

$$k_{\text{f}}:\quad y = -46.7\overline{\langle d_{\text{ij}} \rangle} + 98.6 \qquad (58)$$

$$k_{-1}:\quad y = -7.8\overline{\langle d_{\text{ij}} \rangle} + 12.4 \qquad (59)$$

Using the above equations we can estimate how k_{f} and k_{-1} would change with $\langle d_{\text{ij}} \rangle$ ranging from ~0.5 to 1.2 μm for a nondividing cell (Fig. 4d, e in the main text and Supplementary Figure 47b). For $\text{CueR}^{\text{mE}}_{\text{apo}}$, its k_{f} can change from 6.3 to 56.2 $\mu\text{M}^{-1}\text{s}^{-1}$, a factor of ~9 times, and its k_{-1} can change from 7.1 to 10.5 s^{-1},

a factor of ~1.5 times. For $\mathrm{CueR}_{\mathrm{Cu}}^{\mathrm{mE}}$, its k_f can change from 75.3 to 42.6 $\mu\mathrm{M}^{-1}\mathrm{s}^{-1}$, a factor of ~1.8 times, and its k_{-1} can change from 8.5 to 3.0 s^{-1}, a factor of ~2.8 times.

With the chromosome-organization-dependence of k_f and k_{-1}, we also calculated the overall apparent unbinding rate constant (i.e., $k_{-1}+k_\mathrm{f}[\mathrm{P}]_\mathrm{FD}$) across different $\overline{\langle d_\mathrm{ij}\rangle}$ at a known $[\mathrm{P}]_\mathrm{FD}$. For example, for holo-CueR under merely chromosomal expression with $[\mathrm{CueR}]_\mathrm{FD}$ of 50 nM, at $\overline{\langle d_\mathrm{ij}\rangle}$ = 0.5 μm, k_f = 75.3 $\mu\mathrm{M}^{-1}\mathrm{s}^{-1}$ (via Supplementary Equation 58)) and k_{-1} = 8.5 s^{-1} (via Supplementary Equation 59) and thus the overall unbinding rate constant is 12.3 s^{-1}. When $\overline{\langle d_\mathrm{ij}\rangle}$ varies from 0.5 to 1.2 μm, the k_f, and k_{-1} changes to 42.6 $\mu\mathrm{M}^{-1}\mathrm{s}^{-1}$ and 3.0 s^{-1}, respectively. This results in an overall unbinding rate constant to decrease to 5.2 s^{-1}, compared with which, 12.3 s^{-1} is 237% larger. Therefore, having the chromosome in a less condensed state can help holo-CueR unbind slower, which would keep activating transcription of metal resistance genes longer.

For apo-CueR, with the same $[\mathrm{CueR}]_\mathrm{FD}$ of 50 nM and with $\overline{\langle d_\mathrm{ij}\rangle}$ varies from 0.5 to 1.2 μm, we can also estimate the k_f (via Supplementary Equation 56), k_{-1} (via Supplementary Equation 57), and the overall unbinding rate constant (via $k_{-1}+k_\mathrm{f}[\mathrm{P}]_\mathrm{FD}$) at each $\overline{\langle d_\mathrm{ij}\rangle}$. The calculated unbinding rate constant changes from 7.4 to 13.3 s^{-1}, a ~80% increase. Therefore, having the chromosome in the less condensed state can help apo-CueR unbind faster, leading to more facile transcription activation of metal resistance genes. On the other hand, having the chromosome in the more condensed state can help apo-CueR unbind slower, which would give longer repression and less activation of metal resistance genes.

21.4 For dividing cells, $\mathrm{CueR}_{\mathrm{apo}}^{\mathrm{mE}}$ *and* $\mathrm{ZntR}_{\mathrm{apo}}^{\mathrm{mE}}$ *show slower unbinding kinetics from recognition sites*

Literature has reported that the compaction of *E. coli* chromosome segments during the bulk chromosome segregation process [92] serves as the principal driving force for drawing replicated sisters away from each other [93]. Therefore, compared with nondividing cells, the dividing cells should have more condensed/compact chromosome in general. This led us to examine the unbinding kinetics of $\mathrm{CueR}_{\mathrm{apo}}^{\mathrm{mE}}$ and $\mathrm{ZntR}_{\mathrm{apo}}^{\mathrm{mE}}$ in dividing cells (i.e., cells that show a clear division septum) versus those in nondividing cells. According to our results from Section 21.1, k_{-1}^{app} of $\mathrm{CueR}_{\mathrm{apo}}^{\mathrm{mE}}$ and $\mathrm{ZntR}_{\mathrm{apo}}^{\mathrm{mE}}$ should be slower in dividing cells than in nondividing cells.

We first used the same approach of calculating $\langle d_\mathrm{ij}\rangle$ as described in Section 20.1 to quantify the chromosome condensation of each dividing cell. A special note here: for dividing cells, each cell has two copies of chromosome and the distribution of the residence sites shows clearly two chromosomes located on each side of the division septum (Supplementary Figure 44c). We thus calculate d_ij with residence sites on each side of the division septum and quantified the condensation of the dividing cell (i.e., $\langle d_\mathrm{ij}\rangle$) by averaging d_ij obtained from both chromosomes. Supplementary Figure 47b, as well as Supplementary Figure 45b lower, shows the distribution of $\langle d_\mathrm{ij}\rangle$ among dividing cells, which could be fitted with a Gaussian distribution. Consistent with expectation that dividing cells have highly condensed chromosomes, the $\langle d_\mathrm{ij}\rangle$ of the dividing cells is clearly shorter than that of the nondividing cells.

With the confirmation of that dividing cells have highly condensed chromosomes and that $\langle d_\mathrm{ij}\rangle$ indeed is a good metric for chromosome condensation, analysis described in Section 14.2 is performed to extract the fractional populations of diffusion states, their respective effective diffusion constants, k_{-2}, and k_{-1}^{app}. Regardless of being in a dividing or non-dividing cells, the three diffusion states of $\mathrm{CueR}_{\mathrm{apo}}^{\mathrm{mE}}$ show similar diffusion constants (i.e., D_FD, D_NB, and D_SB are 4.1 ± 0.2, 0.71 ± 0.03, and 0.044 ± 0.004 $\mu\mathrm{m}^2\mathrm{s}^{-1}$ for

dividing cells and 3.7 ± 0.2, 0.70 ± 0.03, and 0.036 ± 0.009 $\mu m^2 s^{-1}$ for nondividing cells) and k_{-2} (= 2.6 ± 0.1 and 2.5 ± 0.1 s^{-1} for both dividing and nondividing cells) (Supplementary Table 4 and Supplementary Table 7). Supplementary Figure 52b shows k_{-1}^{app} for $CueR_{apo}^{mE}$ in dividing and non-dividing cells across different cellular protein concentrations. Again, both show larger k_{-1}^{app} at higher cellular protein concentrations. More important, at any cellular protein concentration measured here, the dividing cells have smaller k_{-1}^{app} than the non-dividing cells, consistent with our observation earlier that cells with more condensed chromosome have slower $CueR_{apo}^{mE}$ unbinding kinetics from recognition sites. And this trend was already observable in $\langle \tau \rangle$ vs. cellular protein concentration for dividing and nondividing cells (Supplementary Figure 52a).

We repeated the same experiments for cells expressing $ZntR_{apo}^{mE}$. The slower unbinding rate from recognition sites is also observed for dividing cells as compared with nondividing cells (Supplementary Figure 52c). We could not examine the dividing cells for those that express $CueR^{mE}$ and $ZntR^{mE}$ in the presence of high metal concentrations, because under metal stress, these cells are essentially not growing nor dividing, which is expected.

21.5 Chloramphenicol-treated nondividing cells have slower regulator unbinding kinetics from recognition sites

In *E. coli*, it has been reported that the chromosome can undergo progressive changes in shape and compaction in the presence of drugs: for example chloramphenicol binds to the ribosome and stops peptide synthesis, leading to more condensation of DNA [8,104]. We thus performed experiments with chloramphenicol treated nondividing cells that express $CueR_{apo}^{mE}$ and $ZntR_{apo}^{mE}$. Based on our observation that cells have more condensed chromosomes have slower $CueR_{apo}^{mE}$ and $ZntR_{apo}^{mE}$ unbinding kinetics from recognition sites, we expect to observe slower average unbinding rates when the cells are treated by chloramphenicol. Note we did not perform chloramphenicol treatment on metal-stressed cells (i.e., cells having $CueR_{Cu}^{mE}$ or $ZntR_{Zn}^{mE}$), because the respective strains are chloramphenicol resistant (Strains CRM32, ZRM32, CRM32-p, and ZRM32-p in Supplementary Table 3). Without this resistance, we also expect that the combined drug and metal stress would be lethal for the cells.

After growing the cells to OD = 0.3, we treated them with 100 μg/mL chloramphenicol for 30 minutes to induce chromosome compaction, as reported[104,105]. The chloramphenicol-treated cells are smaller than the untreated ones and have the average cell width, length, and aspect ratio of 1.14 ± 0.13 μm, 2.54 ± 0.40 μm and 2.23 ± 0.47 respectively. These cells were then used to perform SMT and kinetic analysis to extract the k_{-1}^{app} .

Supplementary Figure 53a shows the protein-concentration-dependent k_{-1}^{app} for nondividing cells that express $CueR_{apo}^{mE}$ and have been treated with chloramphenicol. Compared with those cells without chloramphenicol treatment, they indeed show slower unbinding rates. The same trend is also observed for chloramphenicol-treated cells expressing $ZntR_{apo}^{mE}$, as shown in Supplementary Figure 53b. Note that for these chloramphenicol-treated cells, there lacked cells having high-enough $ZntR_{apo}^{mE}$ concentrations. As a result, we could not determine k_{-2} and thus k_{-1}^{app} using the procedure in Section 14.2. Instead, to demonstrate the effect of chloramphenicol treatment, we report the inverse of the photobleaching/blinking corrected average residence time, $1/\tau^{pp}$, as described in Section 8. In short, results from chloramphenicol-treated cells support our conclusion that more condensed chromosome is associated with slower unbinding kinetics of $CueR_{apo}^{mE}$ from recognition sites.

We also observed that for these cells, the effective diffusion constants and the k_{-2} also changed (Supplementary Table 4 and Supplementary Table 7). For example, for $\text{CueR}^{\text{mE}}_{\text{apo}}$, the D_{FD}, D_{NB}, and D_{SB} dropped from 3.7 ± 0.2, 0.7 ± 0.03, and 0.036 ± 0.009 to 2.9 ± 0.2, 0.57 ± 0.02, and 0.019 ± 0.004 $\mu m^2 s^{-1}$, respectively, and the k_{-2} dropped from 2.5 ± 0.1 to 1.8± 0.1 s^{-1}. D_{FD}, the apparent diffusion constant of freely diffusing proteins, becomes smaller for chloramphenicol-treated cells, and it is likely a direct consequence of the smaller aspect ratio of these cell (i.e., aspect ratios are 2.23 and 2.48 for chloramphenicol-treated and untreated cells respectively), as predicted in Supplementary Figure 24. The changes in D_{NB}, D_{SB}, and the nonspecific unbinding rate constant k_{-2} under chloramphenicol treatment are not surprising, because these parameters are all associated with protein-chromosome interactions. As chloramphenicol induces significant compaction in chromosome organization, it can affect the motions of nonspecifically bound protein on chromosome (i.e., D_{NB}), the dynamics of chromosome itself (i.e., D_{SB}), and the unbinding rate constant of nonspecifically bound proteins (i.e., k_{-2}).

21.6 Possible mechanism for chromosome-organization-dependent unbinding kinetics of CueR and ZntR from recognition sites: protein-imposed DNA structural distortions

The phenomenon of chromosome-organization-dependent unbinding kinetics of CueR or ZntR from recognition sites immediately raises the question: why and how does this occur mechanistically? Inside cells, the chromosome is always condensed, but more or less so depending on the cell cycle, through DNA supercoiling, organization by nucleoid associated proteins, etc.[92] Depending on the organization state of the chromosome, a variable mechanical tension might exist around the CueR or ZntR recognition sites.

CueR and ZntR, like other MerR-family regulators, distort the DNA structure upon binding to their recognition sites. For example, the holo-regulator bends and unwinds DNA slightly, as shown by crystal structures of related MerR-family regulators in complex with DNA [72,77,78,81,106]. These protein-imposed DNA distortions should lead to susceptibility of the protein-DNA complex to mechanical tensions in DNA (which can also change the DNA structure), giving rise to a possible mechanism for the dependence of CueR or ZntR's unbinding on the chromosome organization. Previous studies have shown that mechanical tensions in DNA can indeed affect protein unbinding kinetics from DNA [107,108].

It is also known that apo and holo forms of MerR-family regulators distort DNA structure differently [72,77,78,81,106], although the detailed structural differences are not yet defined. These differences could be the reason that apo- and holo-CueR or ZntR respond to chromosome organization in opposite trends.

22. Sequence analysis of the *E. coli* genome reveals many potential CueR and ZntR recognition sites scattered throughout the chromosome

22.1 Potential ZntR binding sites other than P_{zntA} in the E. coli genome

Our SMT studies of ZntR^{mE} in the ΔP_{zntA} promoter knockout strain suggest that ZntR has additional recognition site(s) in the *E. coli* genome apart from the well-known recognition site within the promoter of the *zntA* gene. To test this suggestion, we performed a genome search for possible ZntR recognition sites in the *E. coli* chromosome.

We first generated a consensus recognition sequence for ZntR using the known or putative promoter sequences recognized by ZntR from different organisms: *E. coli* P_{zntA} (known) [109], *Photorhabdus luminescens* $P_{PLU4679}$ (putative) [110], and *Salmonella aureus* P_{znt} (known) [51]. Based on the sequence

alignment (Supplementary Figure 54a) and the general observation that MerR-family regulators recognize dyad-symmetric (i.e., palindromic) sequences spaced by a central variable region [72,74], the resulting consensus recognition sequence was divided into three regions (Supplementary Figure 54b): regions 1 and 3 are the two halves of the dyad-symmetric sequence; region 2 is the central variable region.

Next, to find candidate ZntR recognition sites in the *E. coli* chromosome, we searched, using Lasergene DNAStar software, for ZntR box-like sequences on the *E. coli* K-12 MG1655 genome using the consensus sequence (ACTNTGNAGTCGACTCCANAGT) that we generated (Supplementary Figure 54b, lower). For simplicity, we used the consensus ACTNTGNANNNNNNTNCANAGT, whereby region 2, the variable region, has been replaced with N's, representing any base. By allowing total two mismatches in either region 1 or region 3, a total of 88 hits were found, in which 3 hits that contain a single mismatch are given in Table S12 along with P_{zntA}. The obvious hit is the recognition sequence within the promoter of *zntA* gene (entry #1 in Table S12). Three additional hits that are highly similar to the consensus binding sequence are sites within the genes of *yaiT, ato operon,* and *nsrR*. YaiT is a predicted member of the outer membrane porin family [112]. The *ato operon* forms an enzyme complex that functions as an acetyl CoA-transferase in *E. coli* [113]. Lastly, NsrR is a nitrite-sensitive repressor that regulates genes involved in protecting the cell against nitric oxide [114,115].

22.2 Potential CueR binding sites in the E. coli genome

We performed similar searches in the *E. coli* K-12 (MG1655) genome for potential CueR recognition sequences. Supplementary Figure 55a gives the sequence alignments of six known CueR recognition sequences within different promoters from different organisms [29,30,116], as well as the corresponding consensus sequence CCTTNCCCTTACGGGAAGG. Again, the consensus sequence is divided into three regions: regions 1 and 3 are the two halves of the dyad-symmetric sequence; region 2 is the variable region in the center. Selected sequence hits are given in Supplementary Table 13. For simplicity, we used the sequence CCTTCCNNNNNNNGGAAGG as the consensus when searching the genome for CueR box-like sequences.

We found a total of 197 CueR box-like recognition sequences including the promoters of *copA* and *cueO* genes, which are known to be recognized by CueR (entry #1 and #2 in Supplementary Table 13). This number of 197 is the same as the results reported by Yamamoto *et al*. [30] Also included in the list is the sequence within the promoter of *moaABCDE* operon, a set of genes required for molybdopterin biosynthesis, and it was previously identified also by Yamamoto *et al*. [30] through sequence analysis. The CueR binding site at the *moaABCDE* operon contains 3 mismatches to the consensus sequence. We identified a total of 12 sequences, which we consider highly similar to the consensus sequence because they contain only one mismatch to the dyad symmetric regions as shown in Supplementary Table 13, including the one at the *moaABCDE* operon promoter.

22.3 CueR or ZntR recognition sites are distributed randomly across the genome map

To determine the locations of the identified CueR or ZntR recognition sites in the chromosome, we generated histograms of CueR and ZntR recognition sites (Supplementary Figure 56a and b). Based on these histograms, it is evident that for CueR or ZntR, its recognition sites are not clustered in any particular region in the chromosome (i.e., the sites are randomly distributed across the chromosome). This supports the observation that CueR and ZntR specific binding events could sample the entire chromosome equally well and the spatial distribution of their specific binding sites can thus reflect the chromosome organization in the cell.

To further visualize the distribution of the recognition sites, Supplementary Figure 56c and d show the locations of the identified recognition sites of CueR and ZntR, respectively, on the circular genome map. The black ticks outside each circle correspond to the locations of potential recognition sites on the map. Similarly, the sites indeed encompass the whole perimeter of the chromosome. Therefore, the specific binding events of CueR or ZntR can serve to reflect chromosome organization.

Supplementary references

1 Datsenko, K. A. & Wanner, B. L. One-step inactivation of chromosomal genes in *escherichia coli* k-12 using pcr products. *Proc. Nat. Acad. Sci. USA* **97**, 6640-6645 (2000).

2 Hatano, T., Yamaichi, Y. & Niki, H. Oscillating focus of sopa associated with filamentous structure guides partitioning of f plasmid. *Mol. Microbiol.* **64**, 1198-1213 (2007).

3 Yu, D. *et al.* An efficient recombination system for chromosome engineering in *escherichia coli Proc. Natl. Acad. Sci. USA* **97**, 5978–5983 (2000).

4 Zhang, M. *et al.* Rational design of true monomeric and bright photoactivatable fluorescent proteins. *Nat. Meth.* **9**, 727-729 (2012).

5 Guzman, L. M., Belin, D., Carson, M. J. & Beckwith, J. Tight regulation, modulation, and high-level expression by vectors containing the arabinose pbad promoter. *J. Bacteriol.* **177**, 4121-4130 (1995).

6 Changela, A. *et al.* Molecular basis of metal-ion selectivity and zeptomolar sensitivity by cuer. *Science* **301**, 1383-1387 (2003).

7 Khan, S., Brocklehurst, K. R., Jones, G. W. & Morby, A. P. The functional analysis of directed amino-acid alterations in zntr from escherichia coli. *Biochem. Biophys. Res. Comm.* **299**, 438–445 (2002).

8 McKinney, S. A., Murphy, C. S., Hazelwood, K. L., Davidson, M. W. & Looger, L. L. A bright and photostable photoconvertible fluorescent protein. *Nat. Meth.* **6**, 131-133 (2009).

9 Outten, F. W., Huffman, D. L., Hale, J. A. & O'Halloran, T. V. The independent *cue* and *cus* systems confer copper tolerance during aerobic and anaerobic growth in *escherichia coli*. *J. Biol. Chem.* **276**, 30670-30677 (2001).

10 Pruteanu, M., Neher, S. B. & Baker, T. A. Ligand-controlled proteolysis of the escherichia coli transcriptional regulator zntr. *J. Bacteriol.* **189**, 3017-3025 (2007).

11 Pierucci, O. Chromosome replication and cell division in escherichia coli at various temperatures of growth. *J. Bacteriol.* **109**, 848-854 (1972).

12 Hadizadeh Yazdi, N., Guet, C. C., Johnson, R. C. & Marko, J. F. Variation of the folding and dynamics of the escherichia coli chromosome with growth conditions. *Mol. Microbiol.* **86**, 1318-1333 (2012).

13 Skarstad, K., Steen, H. B. & Boye, E. Cell cycle parameters of slowly growing escherichia coli b/r studied by flow cytometry. *J. Bacteriol.* **154**, 656-662 (1983).

14 Akerlund, T., Nordström, K. & Bernander, R. Analysis of cell size and DNA content in exponentially growing and stationary-phase batch cultures of escherichia coli. *J. Bacteriol.* **177**, 6791-6797 (1995).

15 Nielsen, H. J., Li, Y., Youngren, B., Hansen, F. G. & Austin, S. Progressive segregation of the escherichia coli chromosome. *Mol. Microbiol.* **61**, 383-393 (2006).

16 Youngren, B., Nielsen, H. J., Jun, S. & Austin, S. The multifork escherichia coli chromosome is a self-duplicating and self-segregating thermodynamic ring polymer. *Genes Dev.* **28**, 71-84 (2014).

17 Michelsen, O., Teixeira de Mattos, M. J., Jensen, P. R. & Hansen, F. G. Precise determinations of c and d periods by flow cytometry in escherichia coli k-12 and b/r. *Microbiology* **149**, 1001-1010 (2003).

18 English, B. P. *et al.* Single-molecule investigations of the stringent response machinery in living bacterial cells. *Proc. Natl. Acad. Sci. USA* **108**, E365–E373 (2011).

19 Levsky, J. M., Shenoy, S. M., Pezo, R. C. & Singer, R. H. Single-cell gene expression profiling. *Science* **297**, 836-840 (2002).

20 Taniguchi, Y. *et al.* Quantifying e. Coli proteome and transcriptome with single-molecule sensitivity in single cells. *Science* **329**, 533-538 (2010).

21 Durisic, N., Laparra-Cuervo, L., Sandoval-Alvarez, A., Borbely, J. S. & Lakadamyali, M. Single-molecule evaluation of fluorescent protein photoactivation efficiency using an in vivo nanotemplate. *Nat. Meth.* **11**, 156-162 (2014).

22 Annibale, P., Scarselli, M., Greco, M. & Radenovic, A. Identification of the factors affecting co-localization precision for quantitative multicolor localization microscopy. *Opt. Nano.*, 10.1186/2192-2853-1181-1189 (2012).
23 Itan, E., Carmon, G., Rabinovitch, A., Fishov, I. & Feingold, M. Shape of nonseptated escherichia coli is asymmetric. *Phys. Rev. E* **77**, 061902 (2008).
24 Henriques, R. *et al.* Quickpalm: 3d real-time photoactivation nanoscopy image processing in imagej. *Nat. Meth.* **7**, 339-340 (2010).
25 Gould, T. J., Verkhusha, V. V. & Hess, S. T. Imaging biological structures with fluorescence photoactivation localization microscopy. *Nat. Protoc.* **4**, 291-308 (2009).
26 Bakshi, S., Bratton, Benjamin P. & Weisshaar, James C. Subdiffraction-limit study of kaede diffusion and spatial distribution in live escherichia coli. *Biophys. J.* **101**, 2535-2544 (2011).
27 Thompson, R. E., Larson, D. R. & Webb, W. W. Precise nanometer localization analysis for individual fluorescent probes. *Biophys. J.* **82**, 2775-2783 (2002).
28 Yildiz, A. *et al.* Myosin v walks hand-over-hand: Single fluorophore imaging with 1.5-nm localization. *Science* **300**, 2061-2065 (2003).
29 Pérez Audero, M. E. *et al.* Target transcription binding sites differentiate two groups of merr-monovalent metal ion sensors. *Mol. Microbiol.* **78**, 853-865 (2010).
30 Yamamoto, K. & Ishihama, A. Transcriptional response of *escherichia coli* to external copper. *Mol. Microbiol.* **56**, 215-227 (2005).
31 Egler, M., Grosse, C., Grass, G. & Nies, D. H. Role of the extracytoplasmic function protein family sigma factor rpoe in metal resistance of escherichia coli. *J. Bacteriol.* **187**, 2297-2307 (2005).
32 Lu, H. P., Xun, L. & Xie, X. S. Single-molecule enzymatic dynamics. *Science* **282**, 1877-1882 (1998).
33 Zhuang, X. *et al.* A single-molecule study of rna catalysis and folding. *Science* **288**, 2048-2051 (2000).
34 Annibale, P., Scarselli, M., Kodiyan, A. & Radenovic, A. Photoactivatable fluorescent protein meos2 displays repeated photoactivation after a long-lived dark state in the red photoconverted form. *J. Phys. Chem. Lett.* **1**, 1506-1510 (2010).
35 Annibale, P., Vanni, S., Scarselli, M., Rothlisberger, U. & Radenovic, A. Quantitative photo activated localization microscopy: Unraveling the effects of photoblinking. *PLoS ONE* **6**, e22678 (2011).
36 Lee, S.-H., Shin, J. Y., Lee, A. & Bustamante, C. Counting single photoactivatable fluorescent molecules by photoactivated localization microscopy (palm). *Proc. Natl. Acad. Sci. USA* **109**, 17436-17441 (2012).
37 Schütz, G. J., Schindler, H. & Schmidt, T. Single-molecule microscopy on model membranes reveals anomalous diffusion. *Biophys. J.* **73**, 1073-1080 (1997).
38 Gebhardt, J. C. M. *et al.* Single-molecule imaging of transcription factor binding to DNA in live mammalian cells. *Nat. Meth.* **10**, 421-426 (2013).
39 Mazza, D., Abernathy, A., Golob, N., Morisaki, T. & McNally, J. G. A benchmark for chromatin binding measurements in live cells. *Nuc. Acid. Res.* **40**, e119 (2012).
40 Niu, L. & Yu, J. Investigating intracellular dynamics of ftsz cytoskeleton with photoactivation single-molecule tracking. *Biophys. J.* **95**, 2009-2016 (2008).
41 Uphoff, S., Reyes-Lamothe, R., Garza de Leon, F., Sherratt, D. J. & Kapanidis, A. N. Single-molecule DNA repair in live bacteria. *Proc. Natl. Acad. Sci. USA* **110**, 8063-8068 (2013).
42 Mueller, F., Stasevich, T. J., Mazza, D. & McNally, J. G. Quantifying transcription factor kinetics: At work or at play? *Crit. Rev. Biochem. Mol. Biol.* **48**, 492-514 (2013).
43 Lawless, J. F. *Statistical models and methods for lifetime data*. (John Wiley & Sons, 2011).
44 Javer, A. *et al.* Short-time movement of e. Coli chromosomal loci depends on coordinate and subcellular localization. *Nat. Commun.*, 10.1038/ncomms3003 (2013).

45 Cunha, S., Woldringh, C. L. & Odijk, T. Restricted diffusion of DNA segments within the isolated escherichia coli nucleoid. *J. Struct. Biol.* **150**, 226-232 (2005).
46 Mazza, D., Ganguly, S. & McNally, J. G. Monitoring dynamic binding of chromatin proteins in vivo by single-molecule tracking. *Meth. Mol. Biol.* **1042**, 117-137 (2013).
47 Mehta, P. *et al.* Dynamics and stoichiometry of a regulated enhancer-binding protein in live escherichia coli cells. *Nat. Commun.*, 10.1038/ncomms2997 (2013).
48 Elf, J., Li, G.-W. & Xie, X. S. Probing transcription factor dynamics at the single-molecule level in a living cell. *Science* **316**, 1191-1194 (2007).
49 Stoyanov, J. V., Hobman, J. L. & Brown, N. L. Cuer (ybbi) of *escherichia coli* is a merr family regulator controlling expression of the copper exporter copa. *Mol. Microbiol.* **39**, 502-511 (2001).
50 Joshi, C. P. *et al.* Direct substitution and assisted dissociation pathways for turning off transcription by a merr-family metalloregulator. *Proc. Nat. Acad. Sci. USA* **109**, 15121-15126 (2012).
51 Singh, V. K. *et al.* Zntr is an autoregulatory protein and negatively regulates the chromosomal zinc resistance operon znt of staphylococcus aureus. *Mol. Microbiol.* **33**, 200-207 (1999).
52 Golding, I. & Cox, E. C. Physical nature of bacterial cytoplasm. *Phys. Rev. Lett.* **96**, 098102 (2006).
53 Feder, T. J., Brust-Mascher, I., Slattery, J. P., Baird, B. & Webb, W. W. Constrained diffusion or immobile fraction on cell surfaces: A new interpretation. *Biophys. J.* **70**, 2767-2773 (1996).
54 Weber, S. C., Spakowitz, A. J. & Theriot, J. A. Bacterial chromosomal loci move subdiffusively through a viscoelastic cytoplasm. *Phys. Rev. Lett.* **104**, 238102 (2010).
55 Martinez, K. A. *et al.* Cytoplasmic ph response to acid stress in individual cells of escherichia coli and bacillus subtilis observed by fluorescence ratio imaging microscopy. *Appl. Environ. Microbiol.* **78**, 3706-3714 (2012).
56 Iafolla, M. A. J. *et al.* Dark proteins: Effect of inclusion body formation on quantification of protein expression. *Proteins: Structure, Function, and Bioinformatics* **72**, 1233-1242 (2008).
57 Huang, Z., Zhang, C., Chen, S., Ye, F. & Xing, X.-H. Active inclusion bodies of acid phosphatase phoc: Aggregation induced by gfp fusion and activities modulated by linker flexibility. *Microb. Cell Fact.* **12**, 25 (2013).
58 Liu, Z. *et al.* Super-resolution imaging and tracking of protein–protein interactions in sub-diffraction cellular space. *Nat. Commun.* **5**, 4443 (2014).
59 Hammar, P. *et al.* The *lac* repressor displays facilitated diffusion in living cells. *Science* **336**, 1595-1598 (2012).
60 Li, G.-W., Berg, O. G. & Elf, J. Effects of macromolecular crowding and DNA looping on gene regulation kinetics. *Nat. Phys.* **5**, 294-297 (2009).
61 Persson, F., Linden, M., Unoson, C. & Elf, J. Extracting intracellular diffusive states and transition rates from single-molecule tracking data. *Nat. Meth.* **10**, 265-269 (2013).
62 Outten, F. W., Outten, C. E., Hale, J. & O'Halloran, T. V. Transcriptional activation of an *escherichia coli* copper efflux regulation by the chromosomal merr homologue, cuer. *J. Biol. Chem.* **275**, 31024-31029 (2000).
63 Grass, G. & Rensing, C. Genes involved in copper homeostasis in *escherichia coli*. *J. Bacteriol.* **133**, 2145–2147 (2001).
64 Outten, C. E. & O'Halloran, T. V. Femtomolar sensitivity of metalloregulatory proteins controlling zinc homeostasis. *Science* **292**, 2488-2492 (2001).
65 Hitomi, Y., Outten, C. E. & O'Halloran, T. V. Extreme zinc-binding thermodynamics of the metal sensor/regulator protein, zntr. *J. Am. Chem. Soc.* **123**, 8614-8615 (2001).
66 Chen, P. *et al.* Single-molecule dynamics and mechanisms of metalloregulators and metallochaperones. *Biochemistry* **52**, 7170-7183 (2013).
67 Andoy, N. M. *et al.* Single-molecule study of metalloregulator cuer-DNA interactions using engineered holliday junctions. *Biophys. J.* **97**, 844-852 (2009).

68 Graham, J. S., Johnson, R. C. & Marko, J. F. Concentration-dependent exchange accelerates turnover of proteins bound to double-stranded DNA. *Nuc. Acid. Res.* **39**, 2249-2259 (2011).
69 Sing, C. E., Olvera de la Cruz, M. & Marko, J. F. Multiple-binding-site mechanism explains concentration-dependent unbinding rates of DNA-binding proteins. *Nuc. Acid. Res.* **42**, 3783-3791 (2014).
70 Cocco, S., Marko, J. F. & Monasson, R. Stochastic ratchet mechanisms for replacement of proteins bound to DNA. *Phys. Rev. Lett.* **112**, 238101 (2014).
71 Gibb, B. *et al.* Concentration-dependent exchange of replication protein a on single-stranded DNA revealed by single-molecule imaging. *PloS One*, 10.1371/journal.pone.0087922 (2014).
72 Brown, N. L., Stoyanov, J. V., Kidd, S. P. & Hobman, J. L. The merr family of transcripitional regulators. *FEMS Microbiol. Rev.* **27**, 145-163 (2003).
73 Helmann, J. D., Shewchuk, L. M. & Walsh, C. T. Regulation of gene expression by mercury. *Adv Inorg Biochem* **8**, 33-61 (1990).
74 Hobman, J. L., Wilkie, J. & Brown, N. L. A design for life: Prokaryotic metal-bindng merr family regulators. *BioMetals* **18**, 429-436 (2005).
75 Barkay, T., Miler, S. M. & Summers, A. O. Bacterial mercury resistance from atoms to ecosystems. *FEMS Microbiol. Rev.* **27**, 355-384 (2003).
76 Summers, A. O. Damage control: Regulating defenses against toxic metals and metalloids. *Curr Opin Microbiol* **12**, 138-144 (2009).
77 O'Halloran, T. V., Frantz, B., Shin, M. K., Ralston, D. M. & Wright, J. G. The merr heavy metal receptor mediates positive activation in a topologically novel transcription complex. *Cell* **56**, 119-129 (1989).
78 Frantz, B. & O'Halloran, T. V. DNA distortion accompanies transcriptional activation by the metal-responsive gene-regulatory protein merr. *Biochemistry* **29**, 4747-4751 (1990).
79 Ralston, D. M. & O'Halloran, T. V. Ultrasensitivity and heavy-metal selectivity of the allosterically modulated merr transcription complex. *Proc. Natl. Acad. Sci. USA* **87**, 3846-3850 (1990).
80 Summers, A. O. Untwist and shout: A heavy metal-responsive transcriptional regulator. *J. Bacteriol.* **174**, 3097-3101 (1992).
81 Outten, C. E., Outten, F. W. & O'Halloran, T. V. DNA distortion mechanism for transcriptional activation by zntr, a zn(ii)-responsive merr homologue in *escherichia coli*. *J. Biol. Chem.* **274**, 37517-37524 (1999).
82 Zheleznova, E. E., Markham, P. N., Neyfakh, A. A. & Brennan, R. G. Structural basis of multidrug recognition by bmrr, a transcription activator of a multidrug transporter. *Cell* **96**, 353–362 (1999).
83 Zheleznova, E. E. & Brennan, R. G. Crystal structure of the transcription activator bmrr bound to DNA and a drug. *Nature* **409**, 378-382 (2001).
84 Rosenzweig, A. C. & O'Halloran, T. V. Structure and chemistry of the copper chaperone proteins. *Curr. Opin. Chem. Biol.* **4**, 140-147 (2000).
85 Waldron, K. J., Rutherford, J. C., Ford, D. & Robinson, N. J. Metalloproteins and metal sensing. *Nature* **460**, 823-830 (2009).
86 Waldron, K. J. & Robinson, N. J. How do bacterial cells ensure that metalloproteins get the correct metal? *Nat. Rev. Microbiol.* **7**, 25-35 (2009).
87 Grossoehme, N. E. & Giedroc, D. P. Energetics of allosteric negative coupling in the zinc sensor s. Aureus czra. *J. Am. Chem. Soc.* **131**, 17860-17870 (2009).
88 Giedroc, D. P. & Arunkumar, A. I. Metal sensor proteins: Nature's metalloregulated allosteric switch. *Dalton Trans.*, 3107-3120 (2007).
89 Pelletier, J. *et al.* Physical manipulation of the escherichia coli chromosome reveals its soft nature. *Proc. Natl. Acad. Sci. USA* **109**, E2649-E2656 (2012).
90 Wang, W., Li, G.-W., Chen, C., Xie, X. S. & Zhuang, X. Chromosome organization by a nucleoid-associated protein in live bacteria. *Science* **333**, 1445-1449 (2011).

91 Sanamrad, A. *et al.* Single-particle tracking reveals that free ribosomal subunits are not excluded from the escherichia coli nucleoid. *Proc. Natl. Acad. Sci. USA* **111**, 11413-11418 (2014).

92 Wang, X., Llopis, P. M. & Rudner, D. Z. Organization and segregation of bacterial chromosomes. *Nat. Rev. Gen.* **14**, 191-203 (2013).

93 Marko, J. F. Linking topology of tethered polymer rings with applications to chromosome segregation and estimation of the knotting length. *Phys. Rev. E* **79**, 051905 (2009).

94 Wang, X., Liu, X., Possoz, C. & Sherratt, D. J. The two escherichia coli chromosome arms locate to separate cell halves. *Genes Dev.* **20**, 1727-1731 (2006).

95 Kuhlman, T. E. & Cox, E. C. Gene location and DNA density determine transcription factor distributions in escherichia coli. *Mol. Syst. Biol.* **8**, 610 (2012).

96 Benza, V. G. *et al.* Physical descriptions of the bacterial nucleoid at large scales, and their biological implications. *Rep. Prog. Phys.* **75**, 076602 (2012).

97 Dillon, S. C. & Dorman, C. J. Bacterial nucleoid-associated proteins, nucleoid structure and gene expression. *Nat. Rev. Microbiol.* **8**, 185-195 (2010).

98 Toro, E. & Shapiro, L. Bacterial chromosome organization and segregation. *Cold Spring Harb. Perspect. Biol.* **2**, a000349 (2010).

99 Wang, Q., Mordukhova, E. A., Edwards, A. L. & Rybenkov, V. V. Chromosome condensation in the absence of the non-smc subunits of mukbef. *J. Bacteriol.* **188**, 4431-4441 (2006).

100 She, W., Wang, Q., Mordukhova, E. A. & Rybenkov, V. V. Mukef is required for stable association of mukb with the chromosome. *J. Bacteriol.* **189**, 7062-7068 (2007).

101 Megerle, J. A., Fritz, G., Gerland, U., Jung, K. & Rädler, J. O. Timing and dynamics of single cell gene expression in the arabinose utilization system. *Biophys. J.* **95**, 2103-2115 (2008).

102 Baba, T. *et al. Construction of escherichia coli k-12 in-frame, single-gene knockout mutants: The keio collection*. Vol. 2 (2006).

103 Teif, V. B. & Bohinc, K. Condensed DNA: Condensing the concepts. *Prog. Biophys. Mol. Biol.* **105**, 208-222 (2011).

104 Zimmerman, S. B. Shape and compaction of escherichia coli nucleoids. *J Struct Biol* **156**, 255-261 (2006).

105 Zimmerman, S. B. Toroidal nucleoids in escherichia coli exposed to chloramphenicol. *J Struct Biol* **138**, 199-206 (2002).

106 Kliegman, J. I., Griner, S. L., Helmann, J. D., Brennan, R. G. & Glasfeld, A. Structural basis for the metal-selective activation of the manganese transport regulator of *bacillus subtilis*. *Biochemistry* **45**, 3493-3505 (2006).

107 Xiao, B., Johnson, R. C. & Marko, J. F. Modulation of hu-DNA interactions by salt concentration and applied force. *Nuc. Acid. Res.* **38**, 6176-6185 (2010).

108 Marko, J. F. & Siggia, E. D. Driving proteins off DNA using applied tension. *Biophys. J.* **73**, 2173-2178 (1997).

109 Brocklehurst, K. R. *et al.* Zntr is a zn(ii)-responsive merr-like transcriptional regulator of znta in escherichia coli. *Mol. Microbiol.* **31**, 893-902 (1999).

110 Permina, E., Kazakov, A., Kalinina, O. & Gelfand, M. Comparative genomics of regulation of heavy metal resistance in eubacteria. *BMC Microbiol.* **6**, 49 (2006).

111 Crooks, G. E., Hon, G., Chandonia, J.-M. & Brenner, S. E. Weblogo: A sequence logo generator. *Gen. Res.* **14**, 1188-1190 (2004).

112 Zhai, Y. & Saier, M. H. The β-barrel finder (bbf) program, allowing identification of outer membrane β-barrel proteins encoded within prokaryotic genomes. *Prot. Sci.* **11**, 2196-2207 (2002).

113 Sramek, S. J. & Frerman, F. E. Purification and properties of escherichia coli coenzyme a-transferase. *Arch. Biochem. Biophys.* **171**, 14-26 (1975).

114 Bodenmiller, D. M. & Spiro, S. The yjeb (nsrr) gene of escherichia coli encodes a nitric oxide-sensitive transcriptional regulator. *J. Bacteriol.* **188**, 874-881 (2006).

115 Rankin, L. D. *et al.* Escherichia coli nsrr regulates a pathway for the oxidation of 3-nitrotyramine to 4-hydroxy-3-nitrophenylacetate. *J. Bacteriol.* **190**, 6170-6177 (2008).
116 Adaikkalam, V. & Swarup, S. Molecular characterization of an operon, cuear, encoding a putative p1-type atpase and a merr-type regulatory protein involved in copper homeostasis in pseudomonas putida. *Microbiology* **148**, 2857-2867 (2002).
